# Enhanced bovine genome annotation through integration of transcriptomics and epi-transcriptomics datasets facilitates genomic biology

**DOI:** 10.1093/gigascience/giae019

**Published:** 2024-04-16

**Authors:** Hamid Beiki, Brenda M Murdoch, Carissa A Park, Chandlar Kern, Denise Kontechy, Gabrielle Becker, Gonzalo Rincon, Honglin Jiang, Huaijun Zhou, Jacob Thorne, James E Koltes, Jennifer J Michal, Kimberly Davenport, Monique Rijnkels, Pablo J Ross, Rui Hu, Sarah Corum, Stephanie McKay, Timothy P L Smith, Wansheng Liu, Wenzhi Ma, Xiaohui Zhang, Xiaoqing Xu, Xuelei Han, Zhihua Jiang, Zhi-Liang Hu, James M Reecy

**Affiliations:** Department of Animal Science, Iowa State University, Ames, IA 50011, USA; Department of Animal and Veterinary and Food Science, University of Idaho, ID 83844, USA; Department of Animal Science, Iowa State University, Ames, IA 50011, USA; Department of Animal Science, Pennsylvania State University, PA 16802, USA; Department of Animal and Veterinary and Food Science, University of Idaho, ID 83844, USA; Department of Animal and Veterinary and Food Science, University of Idaho, ID 83844, USA; Zoetis, Parsippany-Troy Hills, NJ 07054, USA; Department of Animal and Poultry Sciences, Virginia Tech, VA 24060, USA; Department of Animal Science, University of California, Davis, CA 95616, USA; Department of Animal and Veterinary and Food Science, University of Idaho, ID 83844, USA; Department of Animal Science, Iowa State University, Ames, IA 50011, USA; Department of Animal Science, Washington State University, WA 99164, USA; Department of Animal and Veterinary and Food Science, University of Idaho, ID 83844, USA; Department of Veterinary Integrative Biosciences, Texas A&M University, TX 77843, USA; Department of Animal Science, University of California, Davis, CA 95616, USA; Department of Animal and Poultry Sciences, Virginia Tech, VA 24060, USA; Zoetis, Parsippany-Troy Hills, NJ 07054, USA; University of Missouri, Columbia, MO 65211, USA; USDA, ARS, USMARC, 68933, USA; Department of Animal Science, Pennsylvania State University, PA 16802, USA; Department of Animal Science, Pennsylvania State University, PA 16802, USA; Department of Animal Science, Washington State University, WA 99164, USA; Department of Animal Science, University of California, Davis, CA 95616, USA; Department of Animal Science, Washington State University, WA 99164, USA; Department of Animal Science, Washington State University, WA 99164, USA; Department of Animal Science, Iowa State University, Ames, IA 50011, USA; Department of Animal Science, Iowa State University, Ames, IA 50011, USA

**Keywords:** functional genomics, transcriptomics, epi-genetics, multi-omics integration, trait-similarity network, QTL

## Abstract

**Background:**

The accurate identification of the functional elements in the bovine genome is a fundamental requirement for high-quality analysis of data informing both genome biology and genomic selection. Functional annotation of the bovine genome was performed to identify a more complete catalog of transcript isoforms across bovine tissues.

**Results:**

A total of 160,820 unique transcripts (50% protein coding) representing 34,882 unique genes (60% protein coding) were identified across tissues. Among them, 118,563 transcripts (73% of the total) were structurally validated by independent datasets (PacBio isoform sequencing data, Oxford Nanopore Technologies sequencing data, *de novo* assembled transcripts from RNA sequencing data) and comparison with Ensembl and NCBI gene sets. In addition, all transcripts were supported by extensive data from different technologies such as whole transcriptome termini site sequencing, RNA Annotation and Mapping of Promoters for the Analysis of Gene Expression, chromatin immunoprecipitation sequencing, and assay for transposase-accessible chromatin using sequencing. A large proportion of identified transcripts (69%) were unannotated, of which 86% were produced by annotated genes and 14% by unannotated genes. A median of two 5′ untranslated regions were expressed per gene. Around 50% of protein-coding genes in each tissue were bifunctional and transcribed both coding and noncoding isoforms. Furthermore, we identified 3,744 genes that functioned as noncoding genes in fetal tissues but as protein-coding genes in adult tissues. Our new bovine genome annotation extended more than 11,000 annotated gene borders compared to Ensembl or NCBI annotations. The resulting bovine transcriptome was integrated with publicly available quantitative trait loci data to study tissue–tissue interconnection involved in different traits and construct the first bovine trait similarity network.

**Conclusions:**

These validated results show significant improvement over current bovine genome annotations.

## Introduction

Domestic bovine (*Bos taurus*) provide a valuable source of nutrition and an important disease model for humans [1]. Furthermore, cattle have the greatest number of genotype associations and genetic correlations of the domesticated livestock species, which means they provide an excellent model to close the genotype-to-phenotype gap. Furthermore, the functional elements of the genome provide a means whereby complex biological pathways responsible for variation in a particular phenotype can be identified. Therefore, the accurate identification of these elements in the bovine genome is a fundamental requirement for high-quality analysis of data from which both genome biology and genomic selection can be better understood.

Current annotations of farm animal genomes largely focus on the protein-coding regions [2] and fall short of explaining the biology of many important traits that are controlled at the transcriptional level [3–5]. In humans, 93% of trait-associated single nucleotide polymorphisms (SNPs) identified by genome-wide association studies (GWASs) are found in noncoding regions [6]. Therefore, elucidating noncoding functional elements of the genome is essential for understanding the mechanisms that control complex biological processes.

Untranslated regions play critical roles in the regulation of messenger RNA (mRNA) stability, translation, and localization [7], but these regions have been poorly annotated in farm animals [2, 8]. A recent study of the pig transcriptome using single-molecule long-read isoform sequencing technology resulted in the extension of more than 6,000 annotated gene borders compared to Ensembl or NCBI annotations [2].

Small noncoding RNAs, such as microRNAs (miRNA), are known to be involved in gene regulation through posttranscriptional regulation of expression via silencing, degradation, or sequestering to inhibit translation [9–11]. The number of annotated miRNAs in the current bovine genome annotation (Ensembl release 2018–11; 951 miRNAs) is much lower than the number reported in the highly annotated human genome (Ensembl release 2021–03; 1,877 miRNAs).

This study used a comprehensive set of transcriptome and chromatin state data from 50 cattle tissues and cell types to (i) increase the complexity of the bovine transcriptome, comparable to that reported for the highly annotated human genome; (ii) improve the annotation of protein-coding, noncoding, and miRNA genes; (iii) integrate transcriptome data with publicly available quantitative trait loci (QTL) and gene association data to study tissue–tissue interconnection involved in different traits; and (iv) construct the first bovine trait similarity network that recapitulates published genetic correlations.

## Results

The diversity of RNA and miRNA transcripts among 50 different bovine tissues, developmental stages, and cell types was assessed using polyadenylation (poly(A)) selected Illumina high-throughput RNA sequencing (RNA-seq) data (47) and/or miRNA-seq (46) and data (Supplemental File 1). Most of the tissues studied were from Hereford cattle closely related to L1 Dominette 01449, the individual from which the bovine reference genome (ARS-UCD1.2) was sequenced. The 50 tissues and cell samples included follicular cells, myoblasts, 14 mammary gland samples from various stages of mammary gland development and lactation, 8 fetal tissues (78 days of gestation), 8 tissues from adult digestive tract, and 16 other adult organs (Supplemental File 1). A total of approximately 4.1 trillion RNA-seq reads and 1.2 billion miRNA-seq reads were collected, with a minimum of 27.5 million RNA-seq and 9.3 million miRNA-seq reads from each tissue/cell type (average 87.8 ± 49.7 million and 27.6 ± 12.9 million, respectively) (Supplemental File 2: Fig. S1 and Supplemental File 3).

### Transcript-based analyses

The summary of predicted transcript/genes is presented in Table 1. All of the predicted splice junctions across tissues were supported by RNA-seq reads that spanned the splice junction, substantiating the accuracy of the transcript definition from RNA-seq reads.

**Table 1:** Summary of expressed transcripts/genes

Feature	Annotation*		
	Current project	Ensembl (release 2021–03)	NCBI (release 106)
Number of genes	34,882 (21,116)	27,607 (21,880)	35,143 (21,355)
Number of transcripts	160,820 (79,957)	43,984 (37,538)	83,195 (47,280)
Number of spliced transcripts	130,531	37,299	73,423
Number of transcripts per gene	4.9	1.5	2.3
Median number of 5′ UTRs per gene	2	1	1
Median number of 3′ UTRs per gene	1	1	1

* Numbers in parentheses indicate the number of protein-coding genes/transcripts.

**Figure 1:**
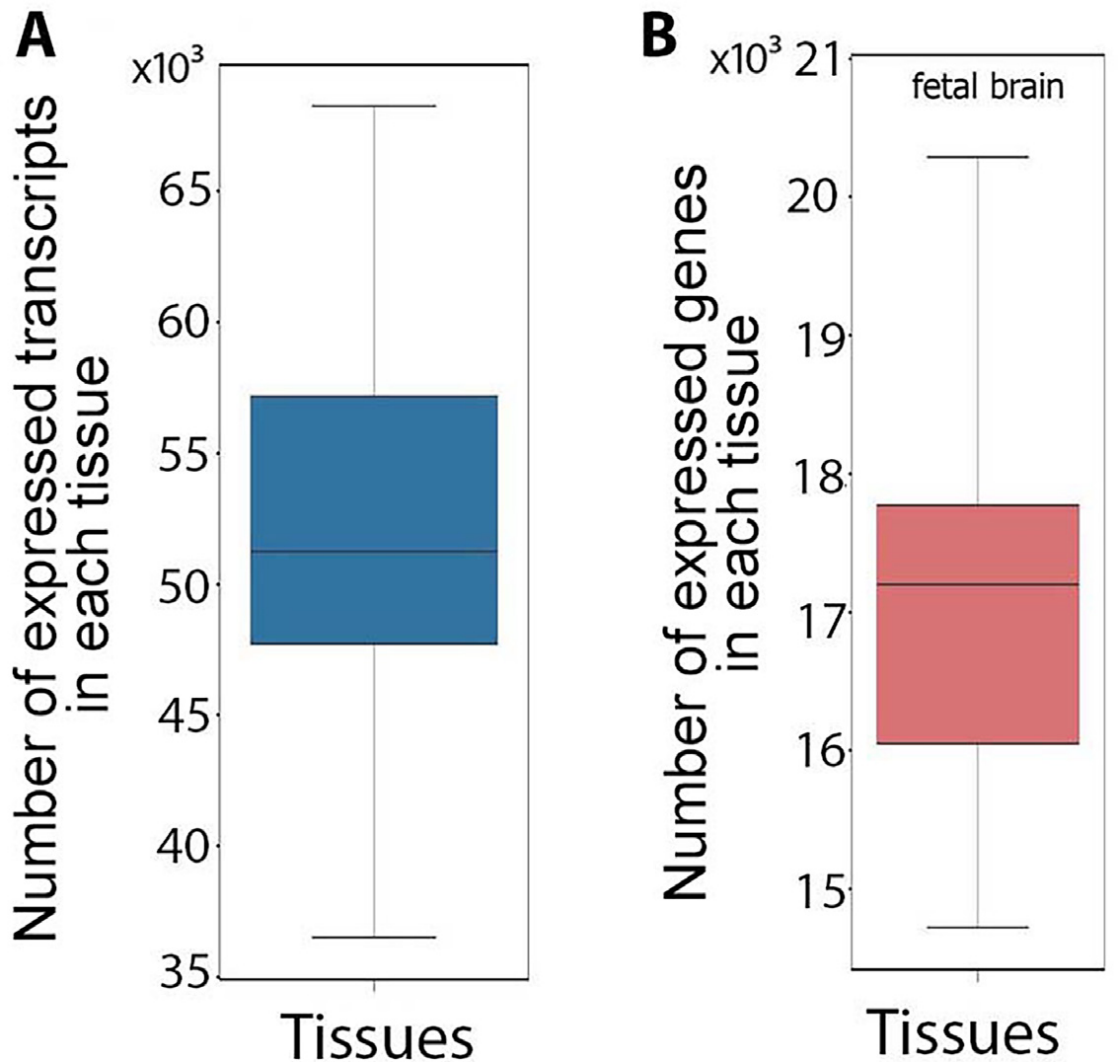
Distribution of the number of expressed transcripts (A) and genes (B) across tissues.

A total of 31,476 transcripts appeared tissue specific by virtue of being assembled from RNA-seq reads in just a single tissue, but 20,100 of those transcripts (64%) were actually expressed in multiple tissues. Thus, reliance solely on assembled transcripts in a given tissue to predict a tissue transcript atlas may overestimate tissue specificity due to a high false-negative rate for transcript detection. To solve this problem of overprediction of tissue specificity, we marked a transcript as “expressed” in a given tissue only if (i) it had been assembled from RNA-seq data in that tissue or (ii) its expression and all of its splice junctions had been quantified using RNA-seq reads in the tissue of interest with an expression level more than 1 read per kilobase of transcript per million reads mapped (RPKM) (see Methods section). This resulted in 145,258 transcripts (90%) expressed in more than 1 tissue (Fig. 1), among which 9,024 transcripts (5%) were found in all 47 tissues examined.

**Figure 2:**
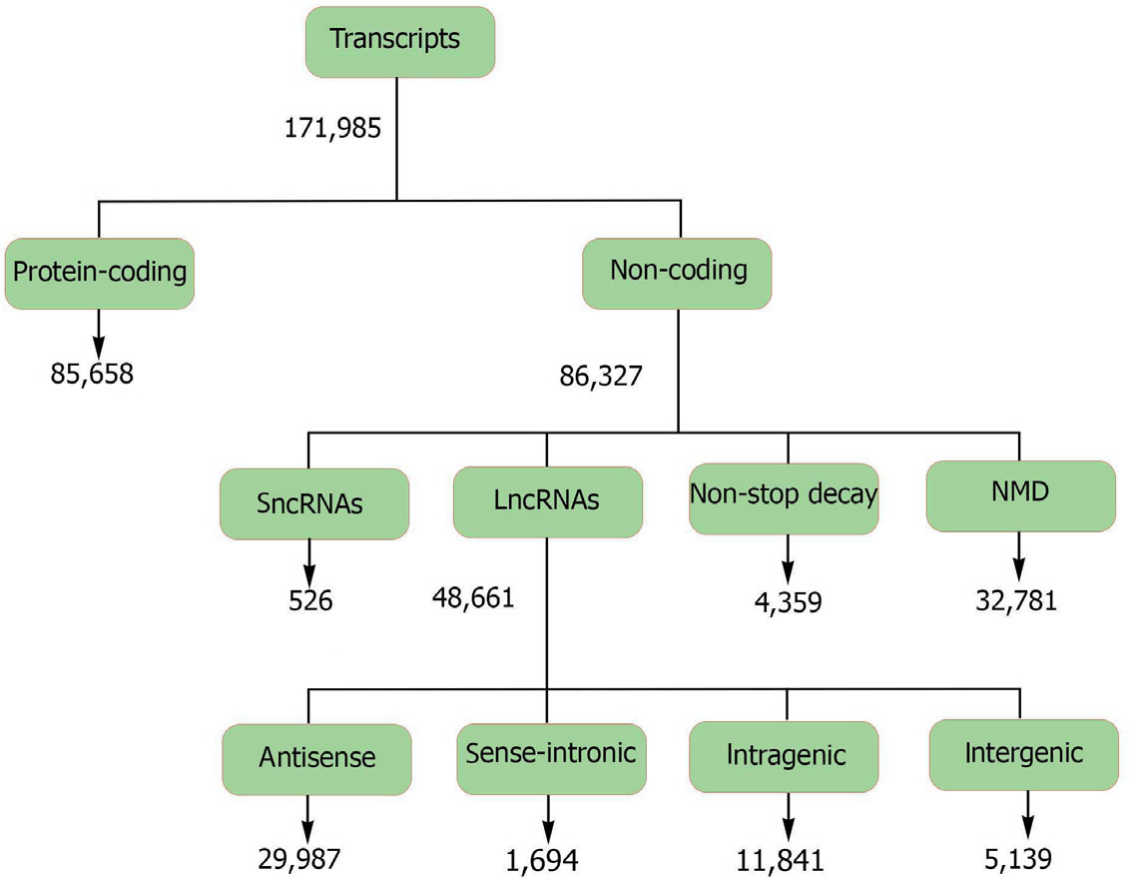
Classification of the predicted transcripts into different biotypes.

The unique transcripts identified were equally distributed between protein-coding transcripts and noncoding transcripts (ncRNAs) (Fig. 2). Noncoding transcripts were further classified as long noncoding RNAs (lncRNAs), nonsense-mediated decay (NMD) transcripts, nonstop decay (NSD) transcripts, and small noncoding RNAs (sncRNAs). While the majority of expressed transcripts in each tissue were protein coding (median of 62% of tissue transcripts), NMD transcripts and antisense lncRNAs each made up more than 10% of the transcripts (Supplemental File 2: Fig. S2A and B, Supplemental Files 4 and 5). Fetal muscle and fetal gonad tissues showed the highest proportion of antisense lncRNAs compared to that observed in other tissues, and around 60% of antisense lncRNAs were expressed from these 2 tissues (Supplemental File 2: Fig. S2B). Compared to noncoding transcripts, protein-coding transcripts were more likely to have spliced exons (*P* < 2.2e-16) and were expressed in a higher number of tissues (*P* < 2.2e-16; Supplemental File 2: Fig. S2C).

There were no significant correlations between the number of RNA-seq reads for a given tissue and the number of transcripts identified, except for a modest correlation for the antisense lncRNA class (Supplemental File 2: Fig. S3A). There was a significant positive correlation (*P =* 1.3e-04) between the number of NMD transcripts in a tissue and the number of protein-coding transcripts, and the NMD transcript class showed the lowest median expression level across tissues compared to other transcript biotypes (Supplemental File 2: Fig. S2D and Fig. S3B).

#### Transcript similarity to other species

Protein/peptide homology analysis of transcripts with an open reading frame (protein-coding transcripts, lncRNAs, and sncRNAs) revealed a higher conservation of protein-coding transcripts compared to lncRNA and sncRNA transcripts (*P* < 2.2e-16) (Table 2). Bovine noncoding transcripts had significantly (*P* < 2.2e-16) less similarity to other species than protein-coding transcripts (Table 2 and Table 3). Within noncoding transcripts, sense intronic lncRNAs showed the highest conservation rate (Table 4).

**Table 2:** Protein/peptide homology of transcripts with coding potential

Transcript biotype	Number of transcripts	Transcripts with protein/peptide homology to other species*
Protein-coding transcripts	85,658	73,268 (86%)
sncRNAs and lncRNAs that encode short peptides^†^	48,425	4,054 (8%)

* Number in parentheses indicates the percentage of each transcript biotype.

† Open reading frame of 9 to 43 amino acids.

**Table 3:** Sequence homology of noncoding transcripts

Transcript biotype	Number of transcripts	Transcripts with sequence homology to ncRNAs in other species*
Long noncoding RNAs	48,661	23,707 (49%)
Small noncoding RNAs	526	194 (37%)
Nonstop decay RNAs	4,359	1,551 (35%)
Nonsense-mediated decay RNAs	32,781	18,195 (55%)

* Number in parentheses indicates the percentage of each transcript biotype.

**Table 4:** Sequence homology of different types of lncRNAs

lncRNA biotype	Number of transcripts	Transcripts with sequence homology to ncRNAs in other species*
Antisense lncRNAs	29,987	13,793 (46%)
Sense-intronic lncRNAs	1,694	1,029 (60%)
Intragenic lncRNAs	5,569	2,314 (41%)
Intergenic lncRNAs	11,841	5,820 (49%)

* Number in parentheses indicates the percentage of each transcript biotype.

#### Transcript expression diversity across tissues

A median of 70% of protein-coding transcripts were shared between pairs of tissues (Supplemental File 2: Fig. S4A), which was significantly higher than that observed for noncoding transcripts (53%; *P* < 2.2e-16; Supplemental File 2: Fig. S5). Clustering of tissues based on protein-coding transcripts was different from that observed based on noncoding transcripts (Supplemental File 2: Fig. S4B and Fig. S5B, Fig. S35F). The fetal tissues clustered together and were generally more similar to one another than to the corresponding adult tissue in both dendrograms. In addition, fetal tissues had significantly higher proportions of noncoding transcripts compared to protein-coding transcripts (*P* < 2.2e-16; Supplemental File 6).

#### Transcript validation

**Figure 3:**
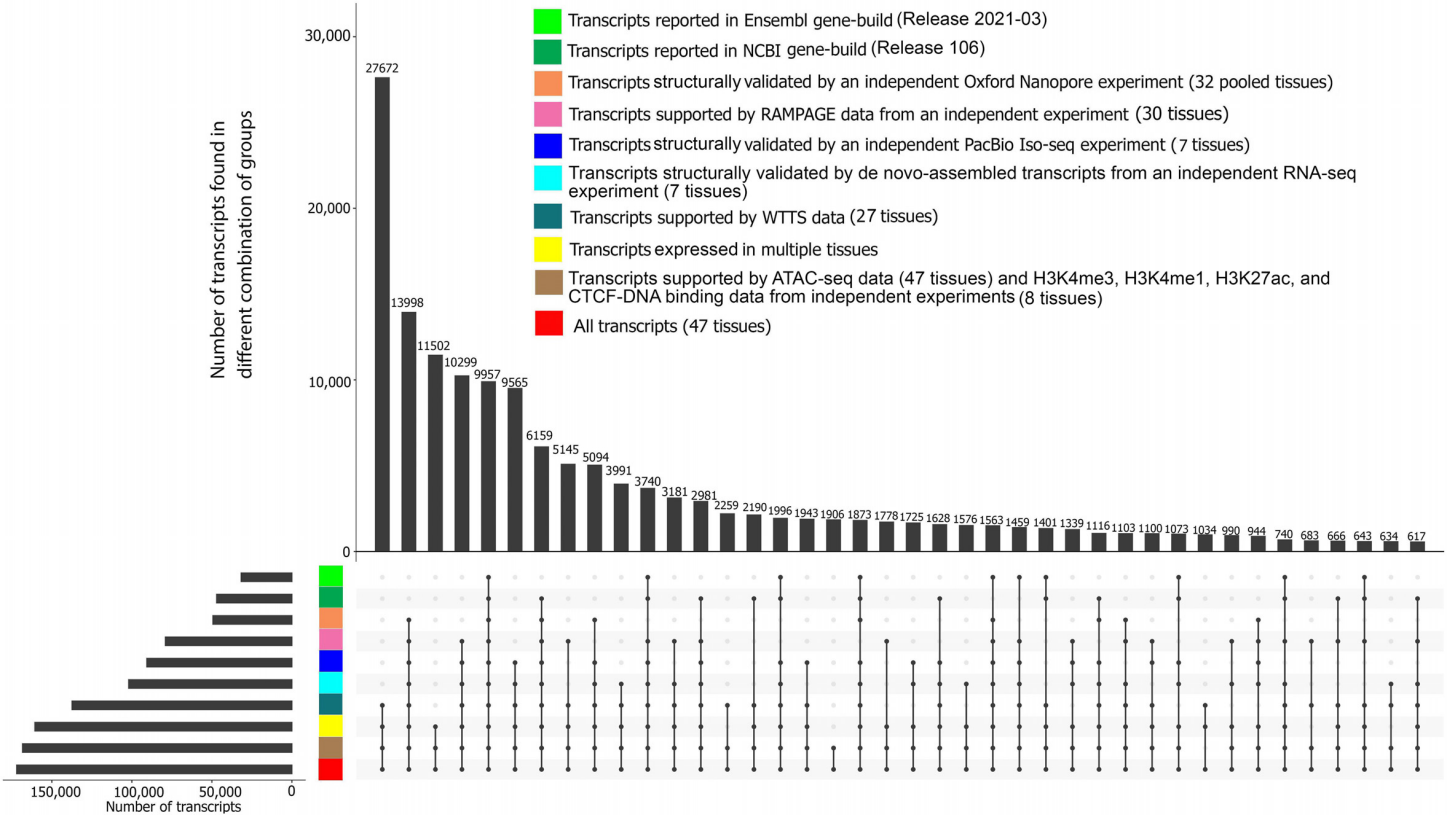
Support of predicted transcripts using data from different technologies and datasets.

Prediction of transcripts and isoforms from RNA-seq data may produce erroneous predicted isoforms. The validity of transcripts was therefore examined by comparison to a library of isoforms taken from Ensembl (release 2021–03) and NCBI gene sets (release 106), as well as isoforms identified through complete isoform sequencing with Pacific Biosciences, a *de novo* assembly produced from its matched RNA-seq reads, and isoforms identified from Oxford Nanopore platforms (see Methods section). A total of 118,563 transcripts (73% of predicted transcripts) were structurally validated by independent datasets (Pacific Biosciences single-molecule long-read isoform sequencing [PacBio Iso-seq], Oxford Nanopore Technologies sequencing [ONT-seq] data, *de novo* assembled transcripts from RNA-seq data) and comparison with Ensembl and NCBI gene sets. A total of 145,258 transcripts were expressed in multiple tissues (90% of predicted transcripts), providing further support for their validity (Fig. 3). All transcripts were also extensively supported by data from different technologies such as whole transcriptome termini site sequencing (WTTS-seq), RNA Annotation and Mapping of Promoters for the Analysis of Gene Expression (RAMPAGE), histone modification (H3K4me3, H3K4me1, H3K27ac), CTCF-DNA binding, and assay for transposase-accessible chromatin using sequencing (ATAC-seq) (Fig. 3).

Comparison of predicted transcript structures with annotated transcripts in the current bovine genome annotations (Ensembl release 2021–03 and NCBI release 106) resulted in a total of 48,906 annotated transcripts that exactly matched previously annotated transcripts (30% of all transcripts), including 44,097 annotated NCBI transcripts, 29,179 annotated Ensembl transcripts, and 24,370 transcripts that were common to both annotated gene sets (Fig. 3). The median expression level of annotated transcripts in their expressed tissues was similar to that observed for unannotated transcripts (Supplemental File 2: Fig. S6). Annotated transcripts were expressed in a higher number of tissues than that observed for unannotated transcripts (*P* = 7.4e-03; Supplemental File 2: Fig. S6). In addition, compared to unannotated transcripts, annotated transcripts were enriched with protein-coding (*P* = 1.37e-02) and spliced transcripts (*P* = 3.76e-02).

The median length of the coding sequence (CDS) of annotated transcripts was significantly longer than that observed in unannotated transcripts (*P* = 0.0) (Supplemental File 2: Fig. S7A). In addition, unannotated transcripts had longer 5′ untranslated regions (UTRs) compared to annotated transcripts (*P* = 2.631E-06; Supplemental File 2: Fig. S7A). Annotated protein-coding transcripts showed a higher GC content in their 5′ UTRs than unannotated transcripts (*P =* 5.562E-18), but both classes of transcripts showed similar GC content within their CDS (Supplemental File 2: Fig. S7B).

### Gene-based analyses

**Figure 4:**
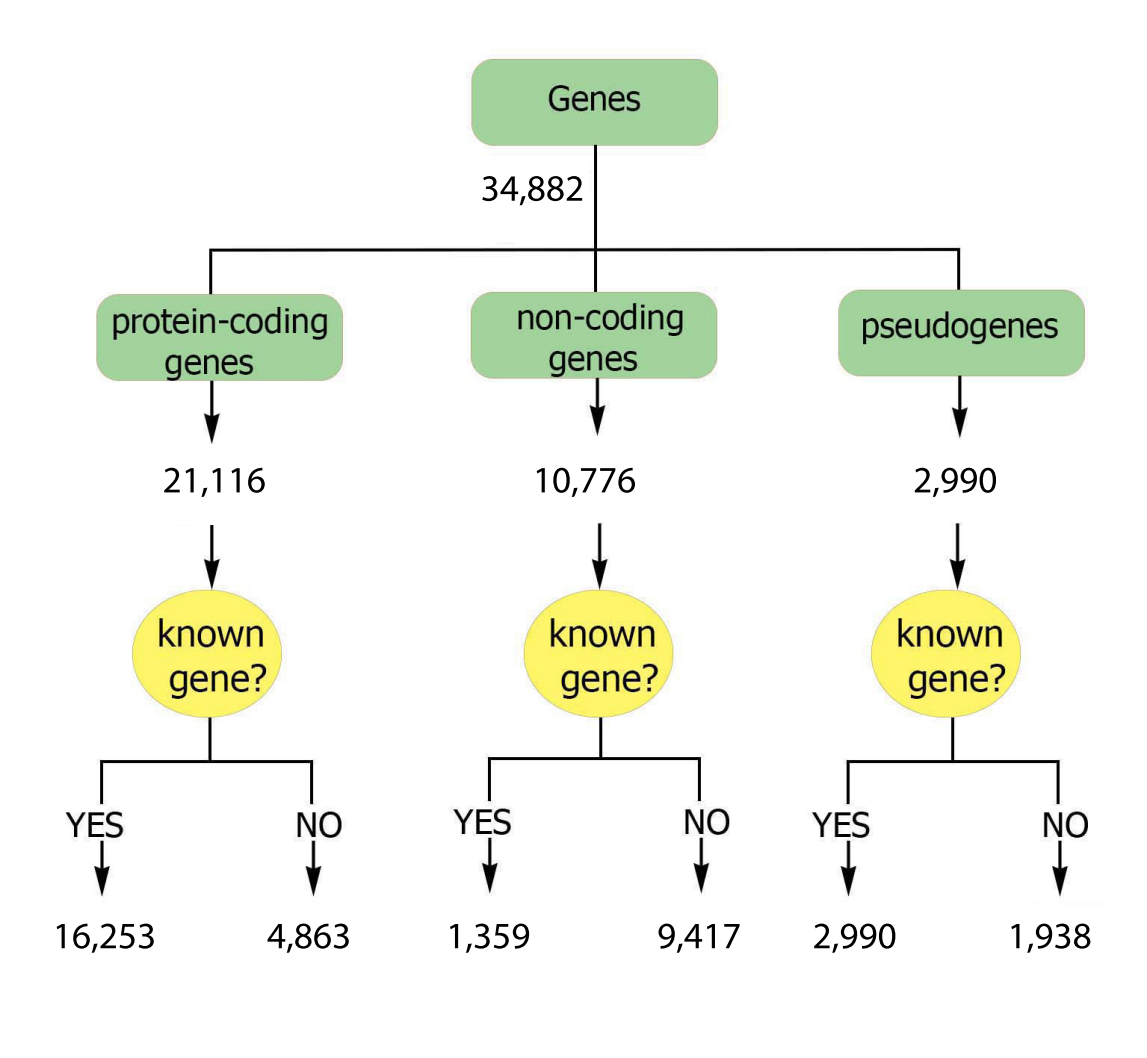
Classification of the predicted genes into different biotypes.

The transcripts correspond to a total of 34,882 genes, which were classified into protein-coding, noncoding, and pseudogenes (Supplemental Files 4 and 5, and Fig. 4). Genes that transcribed at least a single “expressed” transcript (see Transcript-level analysis section) in a given tissue were marked as an “expressed gene” in that tissue. Most genes expressed in each tissue were protein-coding genes, followed by noncoding and pseudogenes (Supplemental File 2: Fig. S8). Testis showed the highest number of expressed genes compared to other tissues (Supplemental File 2: Fig. S8). In addition, the proportion and number of transcribed pseudogenes was higher in the testis than in other tissues (Supplemental File 2: Fig. S8). Fetal brain and fetal muscle tissues showed the highest number and percentage of noncoding genes compared to that observed in other tissues (Supplemental File 2: Fig. S8). There was no significant correlation between the number of input reads and the number of expressed genes across tissues, but the numbers of genes from different coding potential classes were significantly correlated across tissues (Supplemental File 2: Fig. S9).

Transcripts corresponding to the predicted genes that had at least 1 exon overlapping an Ensembl- or NCBI-annotated gene were considered to belong to an annotated gene. This supports an intersection analysis of predicted and previously annotated genes that indicated 22,452 (64%) of our predicted genes correspond to previously annotated genes. Approximately 86% of unannotated transcripts (96,412) were associated with this set of annotated genes. The remaining 12,430 genes (36% of predicted genes) represent unannotated genes, that is, genes not found on Ensembl (release 2021–03) or NCBI (release 106), with which 14% of unannotated transcripts (15,502 transcripts) were associated. The median number of unique transcripts per annotated gene (tpg) was 4, which was higher than that observed in either the Ensembl (1.5 tpg) or NCBI (2.3 tpg) annotated gene sets, while the median number of transcripts per unannotated gene was 1, with an average of 1.31 and standard deviation of 1.36. Most of the transcripts identified were transcribed from annotated genes, including 95% of protein-coding transcripts (76,492), 79% of lncRNA transcripts (37,683), 80% of sncRNA transcripts (281), and more than 95% of NMD transcripts (27,511). Annotated genes were enriched with protein-coding genes (*P* < 2.2e-16). The median transcript abundance from annotated genes in their expressed tissues was significantly higher than that observed for unannotated genes (*P* < 2.2e-16; Supplemental File 2: Fig. S10A). The median number of tissues in which annotated genes were expressed was also significantly higher than that observed for unannotated genes (*P* < 2.2e-16; Supplemental File 2: Fig. S10B).

**Figure 5:**
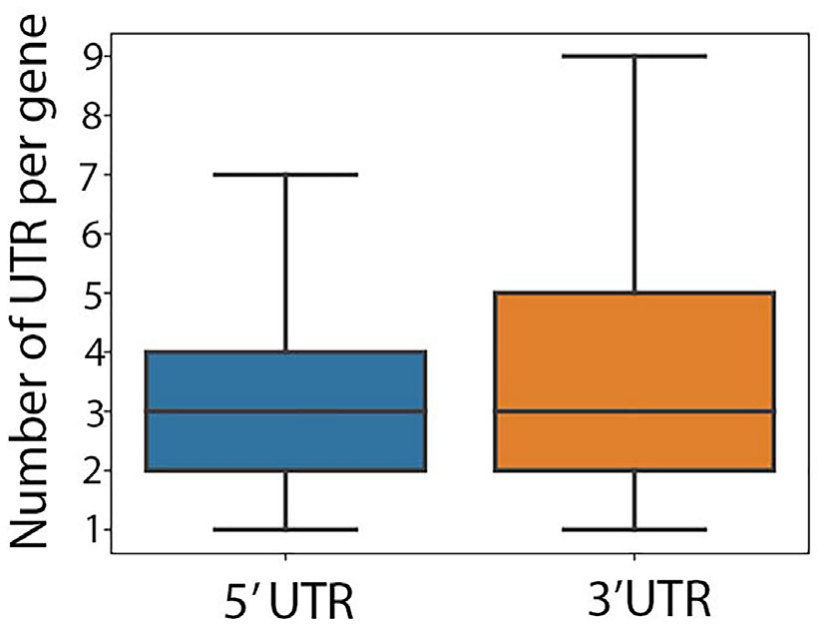
Distribution of the number of 5′ UTRs and 3′ UTRs per gene in genes with multiple UTRs.

More than a third (37%) of genes with at least 1 predicted protein-coding transcript displayed either multiple 5′ UTRs or multiple 3′ UTRs among associated transcript isoforms (Fig. 5). The 496 genes with the highest number of UTRs (the top 5% in this metric) were highly enriched (*q =* 1.7E-7) for the “response to protozoan” Biological Process (BP) Gene Ontology (GO) term (Supplemental File 2: Fig. S11 and Supplemental File 7).

**Figure 6:**
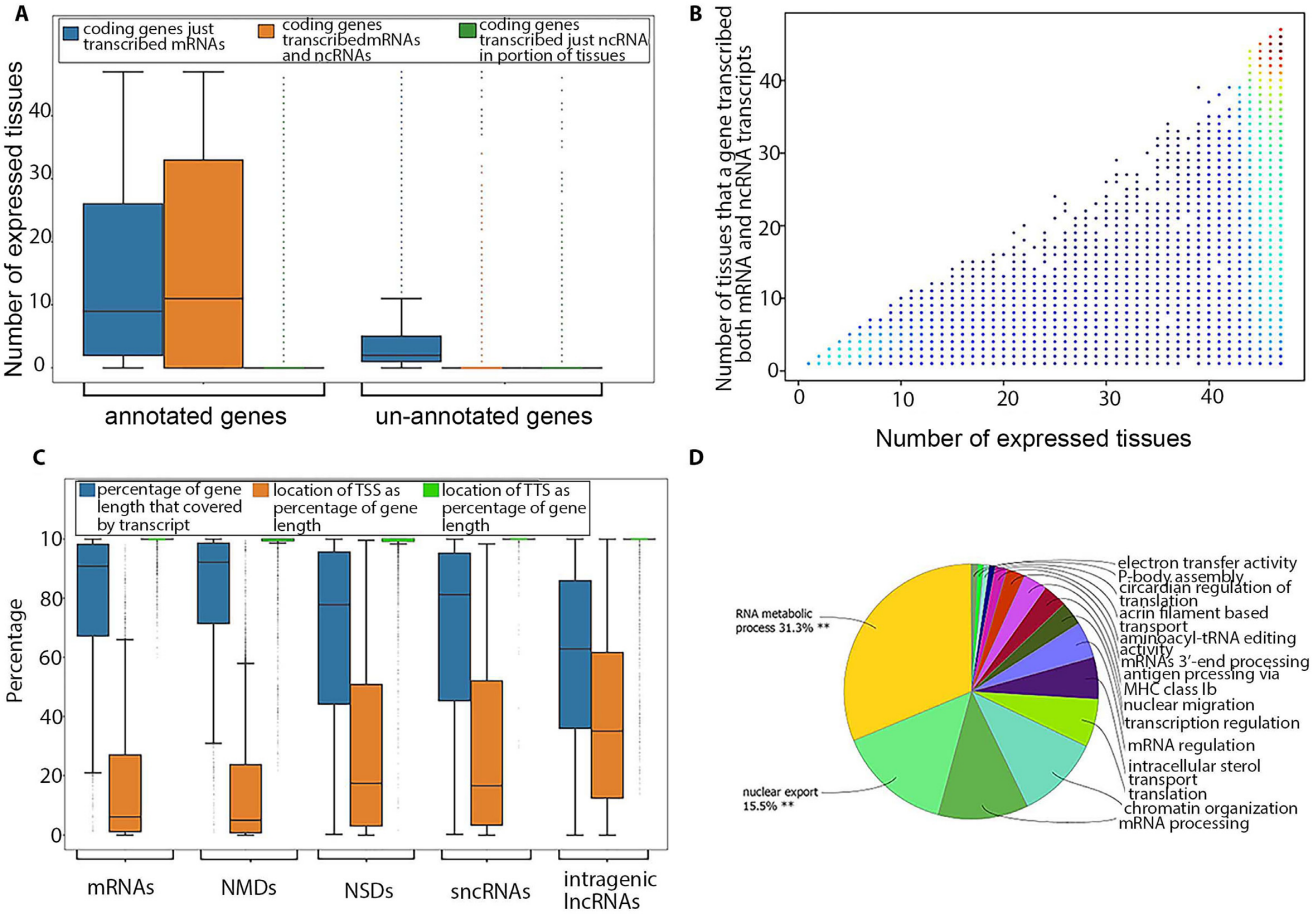
(A) Classification of protein-coding genes based on their novelty and types of encoded transcripts. (B) Number of expressed tissues for bifunctional genes. Dots have been color coded based on their density. (C) Location of different transcript biotypes on bifunctional genes. (D) Functional enrichment analysis of genes that remained bifunctional in all of their expressed tissues.

A median of 51% of the expressed protein-coding genes in each tissue transcribed both protein-coding and noncoding transcripts and were denoted as bifunctional genes. These genes were mostly previously annotated (95%) and had both coding and noncoding transcripts in a median of 21 tissues, representing 57% of their expressed tissues (Fig. 6A, B). Protein-coding transcripts and NMD transcripts covered more than 90% of the exonic length in bifunctional genes (Fig. 6C). This percentage was significantly lower for other types of noncoding transcripts transcribed from bifunctional genes (Fig. 6C). Although transcript terminal sites (TTSs) of transcripts encoded by bifunctional genes were centralized around these genes’ 3′ ends, transcript start sites (TSSs) varied greatly among transcript biotypes (Fig. 6C). The TTSs of NSD transcripts, sncRNAs, and intragenic lncRNAs were shifted from their protein-coding genes’ start sites (Fig. 6C). Genes that transcribed both protein-coding and noncoding transcripts in all of their expressed tissues were highly enriched for “mRNA processing” (*q =* 6.08E-16) and “RNA splicing” (*q =* 1.35E-14) BP GO terms that were mostly (65%) related to different aspects of transcription and translation (Fig. 6D and Supplemental File 8).

A total of 3,744 genes were acting as noncoding in a median of 2 tissues (equivalent to 15% of their expressed tissues) and were switched to protein coding in the remaining expressed tissues. Detailed investigation of these bifunctional genes in tissues from both adult and fetal samples (brain, kidney, muscle, and spleen) revealed the total of 106 noncoding genes (90% annotated) in fetal tissues that were switched to protein-coding genes with only protein-coding transcripts in their matched adult tissues (Supplemental File 2: Fig. S12). Functional enrichment analysis of these genes resulted in the identification of enriched BP GO terms related to “humoral immune response,” “sphingolipid biosynthetic process,” “negative regulation of wound healing,” “cellular senescence,” “symporter activity,” “regulation of lipid biosynthetic process,” and “filopodium assembly” (Supplemental File 2: Fig. S12, Supplemental File 9).

A median of 32% of protein-coding genes in each tissue expressed at least a single potentially aberrant transcript (PAT), that is, NMDs and NSDs. In this group of genes, the number of PATs was strongly correlated with the total number of transcripts (median correlation of 0.61 across all tissues). The median expression level of these genes in their expressed tissues (11.52 RPKM) was significantly higher (*P* < 2.2e-16) than for protein-coding genes with no PATs (4.48 RPKM). In each tissue, protein-coding genes with PATs showed a significantly higher number of introns (*P* < 2.2e-16; median of 65 introns per gene) than that observed in the remainder of protein-coding genes (median of 15 introns per gene). In addition, genes from this group were expressed in a median of 47 tissues, significantly higher (*P* < 2.2e-16) than that observed for the other group of genes (Supplemental File 2: Fig. S13A, B). These genes transcribed a median of 2 PATs in half of their expressed tissues, equivalent to a median of 22% of all their transcripts in each tissue. Protein-coding genes that transcribed PATs as their main transcripts (PATs comprised >50% of their transcripts) in all of their expressed tissues were highly enriched with RNA splicing–related BP GO terms (Supplemental File 10).

### Gene similarity to other species

Eighty-five percent of protein-coding genes (18,087) encoded either homologous proteins or homologous ncRNAs (Supplemental File 2: Fig. S14A). Nineteen percent of protein-coding genes (4,043) encoded cattle-specific proteins (Supplemental File 2: Fig. S14A). Most of these genes (68%) were either annotated genes or genes with homology to another cattle gene(s) that has established homology to genes in other species (Supplemental File 2: Fig. S14C). The remaining 32% of cattle-specific, protein-coding genes (1,293) were denoted as protein-coding orphan genes (Supplemental File 2: Fig. S14C). A median of 70 protein-coding orphan genes were expressed in each tissue. The expression level of these genes was significantly lower than other types of protein-coding genes (Supplemental File 2: Fig. S15A, B). The median number of expressed tissues for protein-coding orphan genes was lower than for other types of protein-coding genes (Supplemental File 2: Fig. S15C). In addition, protein-coding orphan genes only transcribed protein-coding transcripts in their expressed tissue(s).

Fifty percent of noncoding genes (5,559) encoded either homologous short peptides (9–43 amino acids) or homologous ncRNAs (Supplemental File 2: Fig. S14B). There were 5,546 noncoding genes (51% of noncoding genes) that encoded cattle-specific ncRNAs (Supplemental File 2: Fig. S14B). Ninety-nine percent of these genes were either annotated genes or genes with homology to another cattle gene(s) that has established homology to genes in other species (Supplemental File 2: Fig. S14C). The remaining 1% (9 noncoding genes) were denoted as noncoding orphan genes (Supplemental File 2: Fig. S14C). The median number of expressed tissues for noncoding orphan genes was was higher (*P* < 2.2e-16) than for homologous noncoding genes and protein-coding orphan genes (Supplemental File 2: Fig. S15C).

A total of 2,990 pseudogenes were expressed. The median expression level of these genes in their expressed tissues was lower than that observed for protein-coding genes and similar to that observed for noncoding genes (Supplemental File 2: Fig. S16A). Pseudogenes were expressed in a median of 4 tissues (Supplemental File 2: Fig. S16B). In addition, a total of 1,002 pseudogene-derived lncRNAs were expressed. The median expression of pseudogene-derived lncRNAs was similar to that observed for other lncRNAs (Supplemental File 2: Fig. S17A). In addition, pseudogene-derived lncRNAs were expressed in fewer tissues than observed for other lncRNAs (Supplemental File 2: Fig. S17B).

Testis had the highest number of expressed pseudogene-derived lncRNAs compared to other tissues (Supplemental File 2: Fig. S8A, B). The correlation between the number of input reads and the number of pseudogene-derived lncRNAs was not significant (0.25, *P =* 0.09).

#### Gene expression diversity across tissues

Tissue similarities increased dramatically from transcript level to gene level (Supplemental File 2: Fig. S4A, Fig. S5A, Fig. S18A, Fig. S19A). The median percentage of shared genes between pairs of tissues was significantly higher in protein-coding genes compared to noncoding genes (*P* < 2.2e-16; Supplemental File 2: Fig. S18A, Fig. S19A). Clustering of tissues based on protein-coding genes was similar to that observed based on protein-coding transcripts (Supplemental File 2: Fig. S18B, Fig. S19B). The same result was observed in noncoding genes and transcripts. In addition, clustering of tissues based on protein-coding genes was different from that of noncoding genes (Supplemental File 2: Fig. S4B, Fig. S5B, Fig. S18B, Fig. S19B, Fig. S35F).

Tissues with both fetal and adult samples (brain, kidney, muscle, and spleen) were used to investigate gene biotype differences between these developmental stages. Similar to what was observed at the transcript level, fetal tissues were significantly enriched for noncoding genes and pseudogenes and were depleted for protein-coding genes (*P* < 2.2e-16; Supplemental File 10). These results were consistent across all tissues with both adult and fetal samples (Supplemental File 11).

#### Gene validation

**Figure 7:**
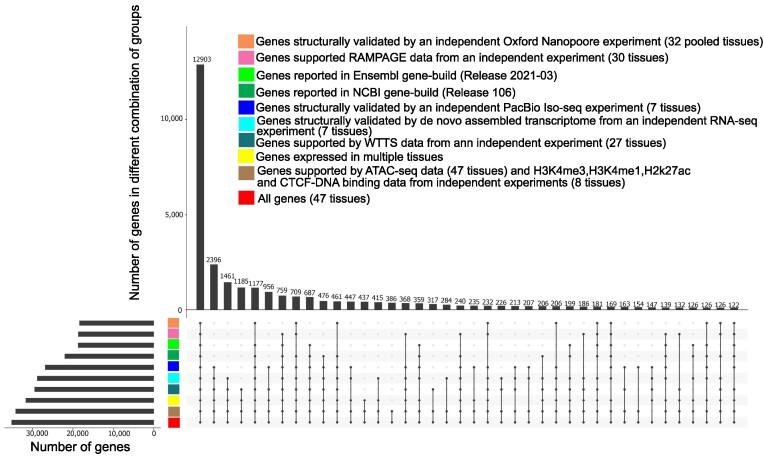
Support of predicted genes using data from different technologies and datasets.

A total of 32,460 genes (93% of predicted genes) were structurally validated by independent datasets (PacBio Iso-seq data, ONT-seq data, *de novo* assembled transcripts from RNA-seq data) and comparison with Ensembl and NCBI gene sets (see Methods section). In addition, a total of 31,635 genes (90% of predicted genes) were expressed in multiple tissues (31,635 genes or 90%) (Fig. 7). All genes were extensively supported by data from different technologies such as WTTS-seq, RAMPAGE, histone modification (H3K4me3, H3K4me1, H3K27ac) and CTCF-DNA binding, and ATAC-seq data generated from the samples (Fig. 7).

#### Identification and validation of annotated gene border extensions

**Figure 8:**
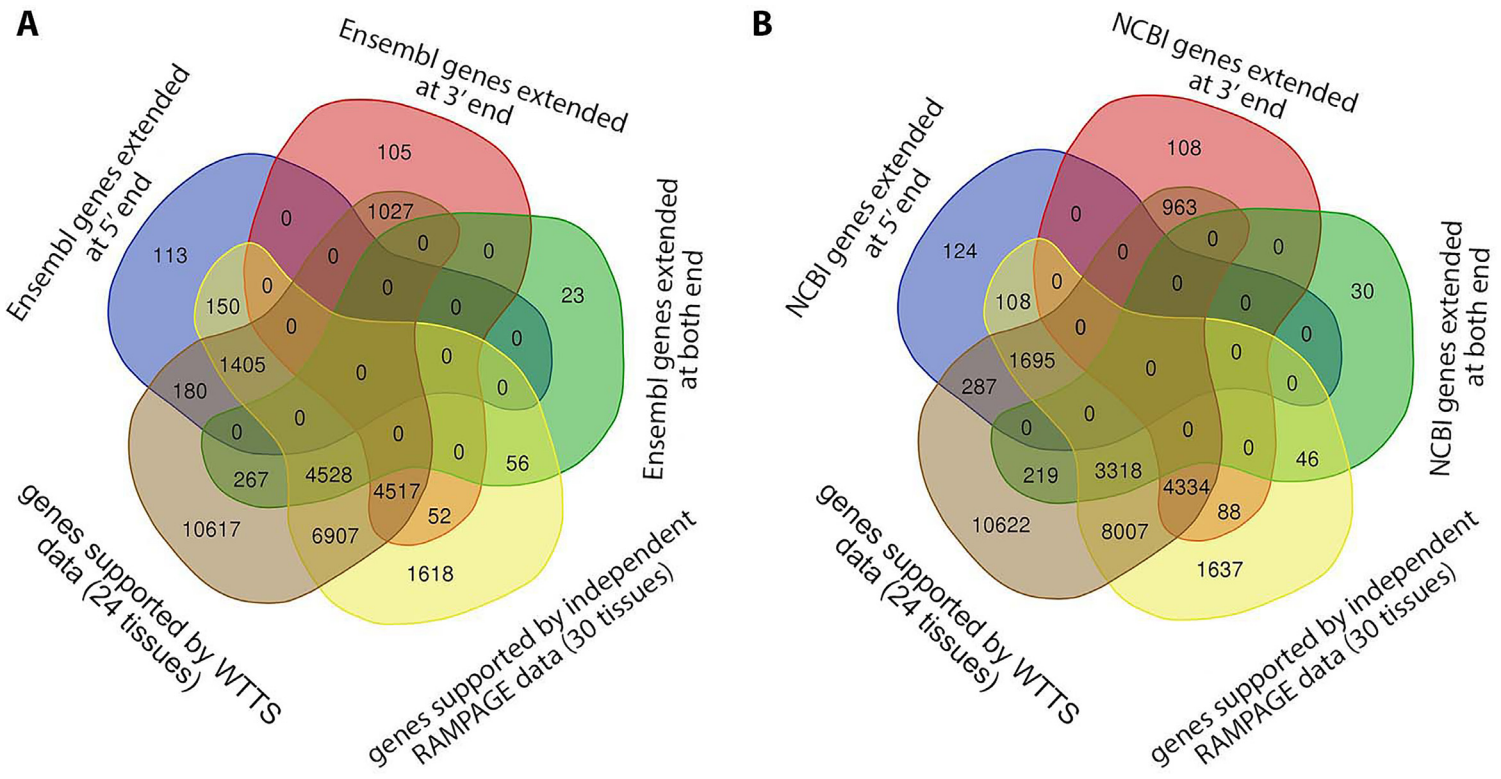
Functional enrichment analysis of noncoding genes in fetal tissues that were switched to protein coding with only coding transcripts in their matched adult tissue.

This new bovine gene set annotation extended (5′ end extension, 3′ end extension, or both) more than 11,000 annotated Ensembl or NCBI gene borders. Extensions were longer on the 3′ side, but the median increase was 104 nt for the 5′ end (Table 5). To validate gene border extensions, independent WTTS-seq and RAMPAGE datasets were utilized. More than 80% of annotated gene border extensions were validated by independent data (Fig. 8). The extension of annotated gene borders on both ends resulted in an approximate 9-fold expression increase of these genes in the new bovine gene set annotation compared to their matched Ensembl and NCBI genes (Table 6).

**Table 5:** Gene border extensions in current ARS-UCD1.2 genome annotations by *de novo* assembled transcriptome from short-read RNA-seq data

Annotation	Type of gene extension	Number of genes	Median extension (nucleotides)
Ensembl (release 2021–03)	5′ extension only	1,848	128
	3′ extension only	5,701	422
	Both ends extended	4,874	122, 5′
			439, 3′
NCBI (release 106)	5′ extension only	2,214	80
	3′ extension only	5,496	126
	Both ends extended	3,613	66, 5′
			210, 3′

**Table 6:** Median number of reads mapped to the extended region of annotated genes*

Annotation	5′ end extension	3′ end extension	Both ends extension
Ensembl (release 2021–03)	92 (1.10)	220 (1.24)	1,766 (8.90)
NCBI (release 106)	72 (1.05)	95 (1.10)	2,009 (9.05)

* Numbers in parentheses indicate the median fold change in expression level resulting from gene extensions.

### Alternative splicing events

A total of 102,502 transcripts (85% of spliced transcripts) were involved in different types of alternative splicing (AS) events (see Methods section and Supplemental File 1: Fig. S20A), a large increase over Ensembl (63% of spliced transcripts) and NCBI (75% of spliced transcripts) annotations (Supplemental File 2: Fig. S20B). Skipped exons were observed in a greater number of transcripts compared to other types of AS events (Supplemental File 2: Fig. S21).

A median of 60% of tissue transcripts showed at least 1 type of AS event (Supplemental File 1: Fig. S22A). There was no significant correlation between the number of input reads and the number of AS event transcripts across tissues (Supplemental File 2: Fig. S22B).

The median expression level of AS transcripts (111,366) was similar to that observed for other types of transcripts (Supplemental File 2: Fig. S23A). In addition, AS transcripts were expressed in a higher number of tissues compared to the other transcript types (Supplemental File 2: Fig. S23B). Alternatively spliced transcripts were enriched with protein-coding transcripts (*P* < 2.2e-16). A switch from protein-coding to ncRNAs was the main biotype change resulting from AS events (Supplemental File 2: Fig. S24).

A median of 4 AS events were expressed in alternatively spliced genes (14,260 genes) (Supplemental File 2: Fig. S25). The top 5% of genes with the highest number of AS events were highly enriched for several BP GO terms related to different aspects of RNA splicing (Supplemental File 2: Fig. S26B, Supplemental File 12).

Comparison of tissues with both fetal and adult samples (brain, kidney, longissimus dorsi [LD] muscle, and spleen) revealed a significantly higher rate of AS events in fetal tissues (only genes expressed in both fetal and adult samples were included in this analysis) (Supplemental File 2: Fig. S27).

### Tissue specificity

Nine percent of all genes and transcripts were only expressed in a single tissue and were denoted as tissue specific (Supplemental File 2: Fig. S28A). Most tissue-specific genes (75%) and transcripts (84%) were unannotated. Forty-nine percent of tissue-specific transcripts (11,748) were produced by annotated genes. Most tissue-specific genes and transcripts were protein coding (Supplemental File 2: Fig. S28A, B). In addition, more than 70% of tissue-specific transcripts (11,222) were transcribed from non-tissue-specific genes. Compared to other tissues, testis and thymus had the highest number of tissue-specific genes and transcripts (Supplemental File 2: Fig. S28C, Supplemental File 12). The expression level of tissue-specific genes and transcripts was significantly lower than that of their non-tissue-specific counterparts (*P* < 2.2e-16; Supplemental File 2: Fig. S28D). A median of 71% of tissue-specific transcripts showed any type of AS event in their expressed tissues (Supplemental File 2: Fig. S29). This was only 3.9% for tissue-specific genes (Supplemental File 2: Fig. S29). Testis, myoblasts, mammary gland, and thymus had the highest proportion of tissue-specific genes displaying any type of AS event (Supplemental File 2: Fig. S29).

A total of 6,744 multitissue expressed genes (21% of all multitissue expressed genes) and 71,662 multitissue expressed transcripts (49% of all multitissue expressed transcripts) showed Tissue Specificity Index (TSI) scores greater than 0.9 and were expressed in a tissue-specific manner (Supplemental File 14). These genes and transcripts were expressed in a median of 6 tissues and 4 tissues, respectively (Supplemental File 2: Fig. S30A, B). Functional enrichment analysis of the top 5% of genes with the highest TSI score resulted in the identification of “sexual reproduction” (*P =* 3.06e-24) and “fertilization” (*P =* 1.04e-8) as their top enriched BP GO terms (Supplemental File 2: Fig. S30C–E, Supplemental File 15).

### Tying genes to phenotypes

There was a median of 7,263 predicted genes identified as the closest expressed gene to an existing QTL (QTL-associated genes) per tissue (Supplemental File 16). These genes had either QTLs located inside (median of 4,563 genes) or outside (median of 4,678 genes) their genomic borders (either from their 5′ end or 3′ end) with a median distance of 51.9 kilobases (KB) and a maximum distance of 2.6 million bases (MB) (Supplemental File 2: Fig. S31). Most QTL-associated genes were annotated genes (8,130 genes or 83%). In addition, the median number of AS events in these genes (8) was significantly higher than that observed in other genes (median of 7 AS events; *P* = 5.69e-09).

### Potential testis–pituitary axis

**Figure 9:**
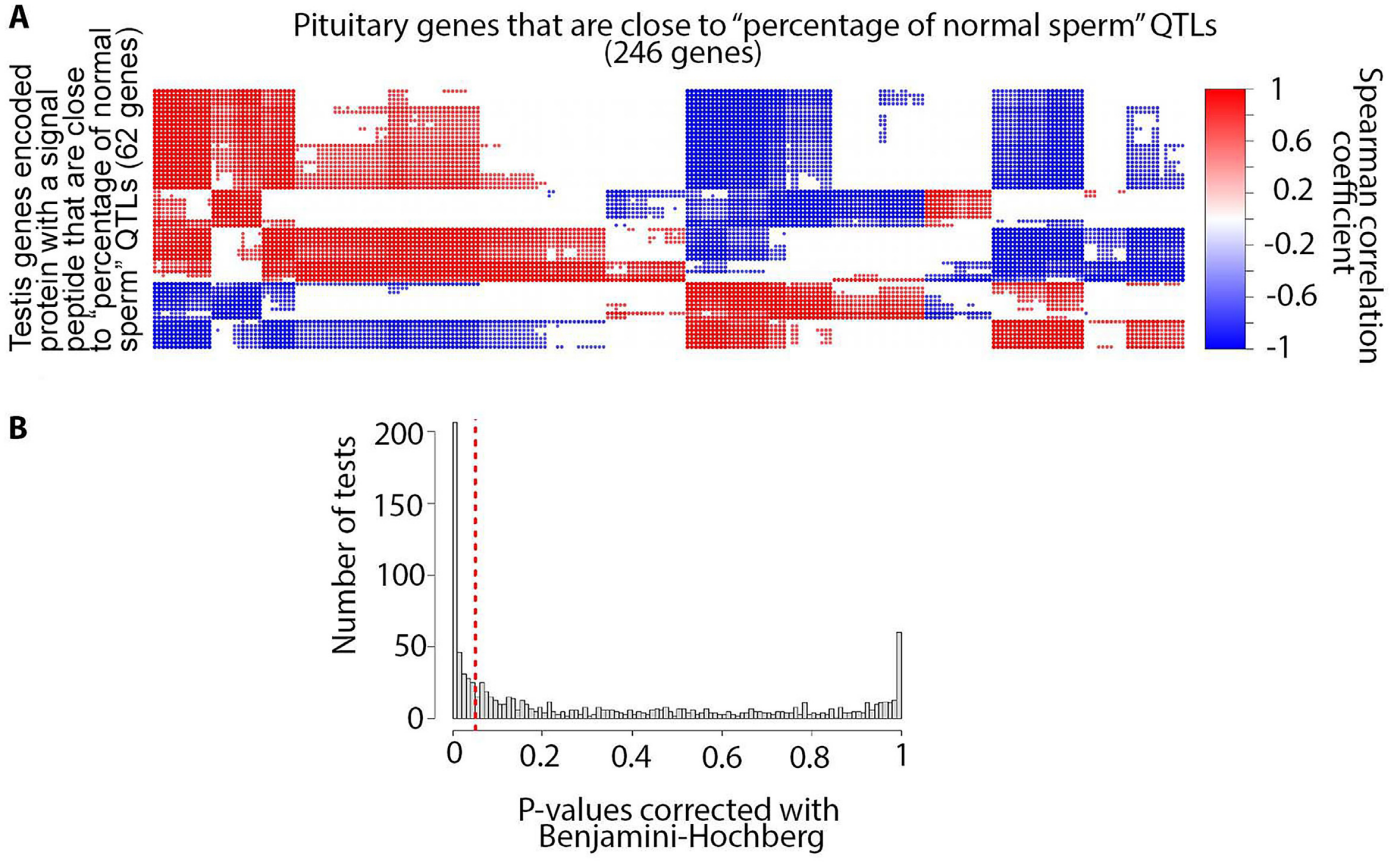
(A) Correlation between testis genes encoded protein with a signal peptide that were close to the “percentage of normal sperm” QTL and pituitary expressed genes closest to this trait (reference correlations). (B) Distribution of *P* values resulting from a right-sided *t*-test between reference correlation coefficients and correlation coefficients derived from random chance (see Methods for details).

Testis tissue was not clustered with any other tissues and had the highest number of tissue-specific genes compared to the rest of the tissues (Supplemental File 2: Fig. S4, Fig. S5, Fig. S18, and Fig. S19). Testis-specific genes were highly enriched with different traits related to fertility (e.g., percentage of normal sperm and scrotal circumference), body weight (e.g., body weight gain and carcass weight), and feed efficiency (e.g., residual feed intake) (Supplemental File 17). The extent of testis–pituitary axis involvement in the “percentage of normal sperm” was investigated using animals with both testis and pituitary samples (3 samples per tissue). The *SPACA5* gene was the only testis-specific gene encoded protein with a signal peptide (SP) that was close to the “percentage of normal sperm” QTLs. The expression of this gene in testis samples showed significant positive correlation with 70 pituitary expressed genes that were closest to the “percentage of normal sperm” QTLs (Supplemental File 2: Fig. S32, Supplemental File 18). These pituitary genes were enriched with the “signal transduction in response to DNA damage” BP GO term (Supplemental File 2: Fig. S32). In addition, the expression of testis genes that encoded protein with a signal peptide that were close to the “percentage of normal sperm” QTLs was significantly correlated with expression of pituitary genes close to this trait (Fig. 9, Supplemental File 19). The same result was observed for the pituitary–testis tissue axis (Supplemental File 2: Fig. S33, Supplemental File 20).

### Trait similarity network

The extent of genetic similarity between different bovine traits was investigated using their associated QTLs. A total of 1,857 significantly similar trait pairs (184 different traits) were identified and used to create a bovine trait similarity network (Supplemental File 21).

## miRNAs

**Figure 10:**
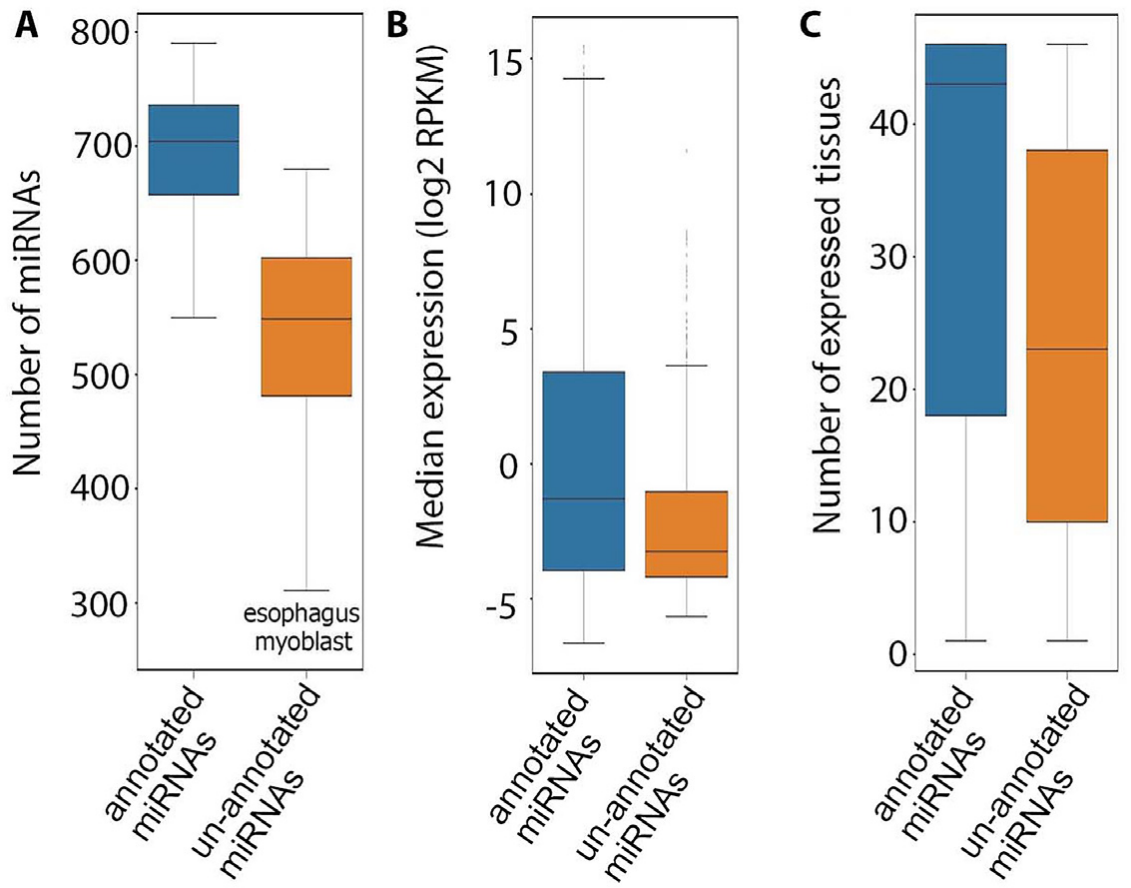
(A) Distribution of the number of expressed annotated and unannotated miRNAs across tissues. (B) Expression of annotated and unannotated miRNAs across their expressed tissues. (C) Number of expressed tissues for annotated and unannotated miRNAs.

A total of 2,007 miRNAs (at least 10 mapped reads in each tissue) comprising 973 annotated and 1,034 unannotated miRNAs were expressed (Supplemental File 22). In each tissue, a median of 704 annotated miRNAs and 549 unannotated miRNAs were expressed (Fig. 10A). The median expression of unannotated miRNAs was significantly lower than that observed for annotated miRNAs (*P =* 3.25e-25; Fig. 10B). In addition, unannotated miRNAs were expressed in a significantly lower number of tissues than for annotated miRNAs (*P =* 1.00e-45; Fig. 10C). A median of 84.53% of miRNAs were shared between pairs of tissues (Supplemental File 2: Fig. S34). Clustering of tissues based on miRNAs was similar to what was observed based on noncoding genes (Supplemental File 2: Fig. S35).

A total of 113 miRNAs (5.6%) were expressed in a single tissue and were denoted as tissue specific (Supplemental File 2: Fig. S36A). The proportion of tissue-specific miRNAs was higher for unannotated miRNAs, such that 75% of the tissue-specific miRNAs were unannotated. The number of unannotated miRNAs was higher in preadipocytes compared to other tissues, followed by fetal gonad and testis (Supplemental File 2: Fig. S36B). Unannotated miRNAs showed a significantly lower expression level compared to annotated miRNAs (*P =* 1.4e-19; Supplemental File 2: Fig. S36C). In addition, a total of 1,047 multitissue expressed miRNAs were expressed in a tissue-specific manner (Supplemental File 2: Fig. S36D). These miRNAs were expressed in a median of 19 tissues (Supplemental File 2: Fig. S36E).

**Figure 11:**
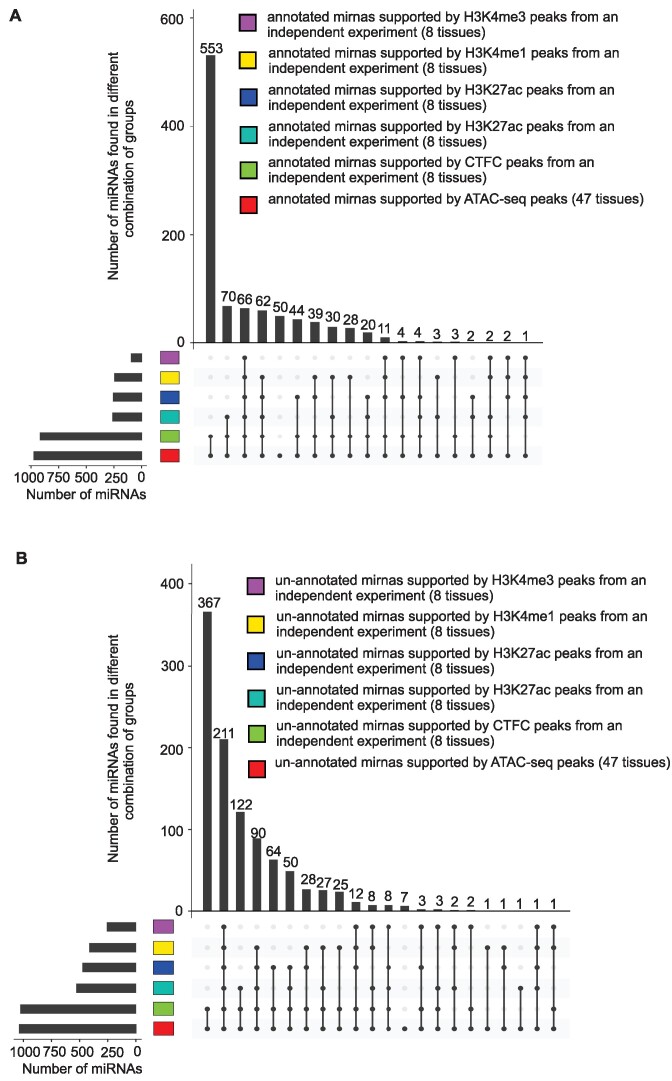
Support of annotated (A) and unannotated (B) miRNAs using different histone marks and CTCF-DNA binding data.

Chromatin features across 500-bp windows surrounding upstream of miRNA precursors’ start sites or downstream of miRNA precursors’ terminal sites from independent cattle experiments were used to investigate the relationship between miRNAs and chromatin accessibility. More than 99% of unannotated miRNAs and 94% of annotated miRNAs were supported by at least one of the H3K4me3, H3K4me1, H3K27ac, CTCF-DNA binding, or ATAC-seq peaks (Fig. 11).

### Summary of expressed transcripts, genes, and miRNAs

The number of expressed transcripts, genes, and miRNAs in different tissues is summarized in Supplemental File 2: Fig. S37. In addition, the number of annotated and unannotated genes, transcripts, and miRNAs in different tissues is summarized in Supplemental File 2: Fig. S38.

## Discussion

Despite many improvements in the current bovine genome annotation ARS-UCD1.2 assembly (Ensembl release 2021–03 and NCBI release 106) compared to the previous genome assembly (UMD3.1), these annotations are still far from complete [12, 13]. In this study, using RNA-seq and miRNA-seq data from 50 different bovine tissues, developmental stages, and cell types, 12,444 unannotated genes and 1,034 unannotated miRNAs were identified that have not been reported in current bovine genome annotations (Ensembl release 2021–03, NCBI release 106, and miRbase [14]). In addition, we identified protein-coding transcripts with a median open reading frame (ORF) length of 270 nt for 822 annotated bovine genes that have been annotated as noncoding in current bovine genome annotations (Supplemental File 2: Fig. S14C). The high frequency of validation of these unannotated genes and unannotated miRNAs using multiple independent datasets from different technologies verifies the improvement in terms of the number of genes and miRNAs using our methods.

The 5′ and 3′ UTR length plays a critical role in regulation of mRNA stability, translation, and localization [7]. However, only a single 5′ UTR and 3′ UTR per gene is annotated in current bovine genome annotations (Ensembl release 2021–03 and NCBI release 106), and variations in UTR length are not available. In this study, 7,909 genes (22% of predicted genes) with multiple UTRs were identified. Genes with multiple 5′ UTRs are common, primarily due to the presence of multiple promoters [15] or alternative splicing mechanisms within 5′ UTRs [15]. Fifty-four percent of human genes have multiple transcription start sites [15]. In addition, the length of 3′ UTRs often varies within a given gene, due to the use of different poly(A) sites [7, 16].

In this study, around 50% of expressed protein-coding genes in each tissue transcribed both coding and noncoding transcript isoforms. Several studies have shown evidence of the existence of bifunctional genes with coding and noncoding potential using RNA-seq and ribosome footprinting followed by sequencing (Ribo-seq) [17–19]. For example, steroid receptor RNA activator (SRA), a known bifunctional gene, acts as a lncRNA while also encoding a conserved protein SRAP, both of which contribute to the development and progression of prostate and breast cancers [20]. More than 20% of human protein-coding genes have been reported to transcribe noncoding isoforms, often generated by alternative splicing [21] and recurrently expressed across tissues and cell lines [19]. A considerable number of noncoding isoform variants of protein-coding genes appear to be sufficiently stable to have functional roles in cells [22]. It has been shown that the proportion of noncoding isoforms from protein-coding genes dramatically increases during myogenic differentiation of primary human satellite cells and decreases in myotonic dystrophy muscles [23]. In this study, 106 noncoding genes were identified in fetal tissues that switched to protein-coding genes in their matched adult tissues. Taken together, this supports the notion that protein-coding/noncoding transcript switching plays an important role in tissue development in cattle as well.

Nonsense-mediated RNA decay is an evolutionarily conserved process involved in RNA quality control and gene regulatory mechanisms [24]. For instance, the RNA-binding protein polypyrimidine tract binding protein 1 (*PTBP1*) can promote the transcription of NMD transcripts via alternative splicing, which negatively regulates its own expression [25]. In this study, NMD transcripts comprised 18% of bovine transcripts that were transcribed from 30% of bovine genes (10,380). In humans, NMD-mediated degradation can affect up to 25% of transcripts [26] and 53% of genes [27]. As expected, in this study, most genes that transcribed NMD transcripts were protein coding (83% or 8,610 genes), while a considerable portion (17%) were pseudogenes. Many pseudogenes are annotated to give rise to NMD transcripts [28, 29]. Bioinformatic study of the human transcriptome revealed that 78% of NMD transcript–producing genes were protein coding, followed by pseudogenes (9%), long intergenic noncoding RNAs (6%), and antisense transcripts (4%) [29].

Despite the important regulatory function of lncRNAs and miRNAs, very low numbers of these elements have been annotated in the current bovine genome annotations (Table 7). In this study, a total of 10,689 lncRNA genes and 2,007 miRNA genes were expressed in the bovine transcriptome, which is similar to what has been reported for the human transcriptome (Table 7), while a total of 3,770 human miRNAs and 1,203 cattle miRNAs have been reported in miRbase [14].

**Table 7:** Comparison of different gene builds based on gene biotypes

Species	Gene build	Protein-coding genes	lncRNA genes	miRNA genes	Other types of small noncoding genes*	Pseudogenes
Bovine (ARS-UCD1.2)	Ensembl (release 2021–03)	21,880	1,480	951	2,209	492
	NCBI (release 106)	21,039	5,179	797	3,249	4,569
	Current project	21,116	10,689	2,007	87	3,029
Human (GRCh38.104)	Ensembl (release 2021–03)	20,442	16,876	1,877	2,930	15,266

* Small nucleolar RNAs, small noncoding RNAs, small Cajal body–specific RNAs, small conditional RNAs, and transfer RNAs.

In this study, 1,002 pseudogene-derived lncRNAs were identified that were recurrently expressed across tissues and cell types. Ever-increasing evidence from different studies suggests pseudogene-derived RNAs are key components of lncRNAs [30–32]. lncRNAs expressed from pseudogenes have been shown to regulate genes with which they have sequence homology [30, 31] or to coordinate development and disease in metazoan systems [30].

Correct annotation of gene borders has an important role in defining promoter and regulatory regions. Our novel transcriptome analysis extended (5′-end extension, 3′-end extension, or both) more than 11,000 annotated Ensembl or NCBI gene borders. Extensions were longer on the 3′ side, which was relatively similar to that we observed in the pig transcriptome using PacBio Iso-seq data [2].

A growing body of evidence indicates that a considerably large portion of lncRNAs encode microproteins that are less conserved than canonical ORFs [33–37]. In this study, most (98%) of the predicted lncRNAs had short ORFs (<44 amino acids) that were less conserved than canonical ORFs (Table 2).

Alternative splicing is the key mechanism to increase the diversity of the mRNA expressed from the genome and is therefore essential for response to diverse environments. In this study, skipped exons and retained introns were the most prevalent AS events identified in the bovine transcriptome, similar to what has been observed in other vertebrates and invertebrates [38]. A higher rate of AS events was observed in fetal tissues compared to their adult tissue counterparts. The same result has been observed in a recently published study in humans [39].

We hypothesized that the integration of the gene/transcript data with previously published QTL/gene association data would allow for the identification of potential molecular mechanisms responsible for (i) tissue–tissue communication as well as (ii) genetic correlations between traits. To test the first hypothesis, we developed a novel approach to study the involvement of tissue–tissue interconnection in different traits based on the integration of the transcriptome with publicly available QTL data. In particular, the interconnection between testis and pituitary tissues with respect to the “percentage of normal sperm” trait was investigated in more detail. This resulted in the identification of the regulation of ubiquitin-dependent protein catabolic process, the regulation of nuclear factor–κB (NF-κB) transcription factor activity, and Rab protein signal transduction as key components of this tissue–tissue interaction (Supplemental Files 19 and 20). Interestingly, expressed genes that were closest to “percentage of normal sperm” QTLs, and also encoded protein with a signal peptide (short peptide present at the N-terminus of proteins that are destined toward the secretory pathway [40]) in both testis and pituitary tissues, were highly enriched for the BP GO term “regulation of ubiquitin-dependent protein catabolic process” (Supplemental Files 18 and 19). The expression of these genes in testis tissue was significantly correlated with expression levels of pituitary expressed genes closest to “percentage of normal sperm” QTLs that were highly enriched for the “positive regulation of NF-kappaB transcription factor activity” BP GO term (Supplemental File 2: Fig. S32 and Supplemental File 19). Activation of NF-κB requires ubiquitination, and this modification is highly conserved across different species [41]. NF-κB induces secretion of adrenocorticotropic hormone from the pituitary [42], which directly stimulates testosterone production by the testis [43]. In addition, ubiquitinated proteins in testis cells are required for the progression of mature spermatozoa [44]. The expression levels of pituitary expressed genes closest to “percentage of normal sperm” QTLs that also encoded signal peptides were significantly correlated with expression levels of testis expressed genes closest to “percentage of normal sperm” QTLs (Supplemental File 2: Fig. S33). These testis genes were highly enriched for the “Rab protein signal transduction” BP GO term (Supplemental File 20). Rab proteins have been reported to be involved in male germ cell development [45]. Thus, it appears that integration of gene data with QTL/association data can be used to identify putative molecular pathways underlying tissue–tissue communication mechanisms.

To test the second hypothesis, we also developed a novel approach to study trait similarities based on the integration of the transcriptome with publicly available QTL data. Using this approach, we could identify significant similarity between 184 different bovine traits. For example, clinical mastitis showed significant similarity with 23 different cattle traits that were greatly supported by published studies, such as milk yield [46], milk composition traits [47], somatic cell score [48], foot traits [49], udder traits [50], daughter pregnancy rate [51], length of productive life [52], and net merit [53]. Similar results were observed for residual feed intake, which showed significant similarity with 14 different traits such as average daily feed intake [54], average daily gain [55], carcass weight [56], feed conversion ratio [57], metabolic body weight [58], subcutaneous fat [59], and dry matter intake [60].

Taken together, these results identify a list of candidate genes that might be controlled by genetic variation responsible for the genetic mechanisms underlying genetic correlations (Supplemental Files 19 and 20). If this is the case, in the future, these novel methods should be able to predict the impact of a given set of genetic variants that are associated with a trait of interest on other traits that were not measured in a given study. This might then lead to the optimization of variants used (or not used) in genomic selection to minimize any nonbeneficial effect of selection on selected traits. However, it is important to acknowledge that (i) the nearest neighbor gene to a genotype association may not necessarily be the causal gene, (ii) the breed/gender differences between this study and the data from Animal QTLdb may impact the results, and (iii) due to experimental limitations, the genetic and phenotypic association data were not used in this study. Nonetheless, these results are intriguing in that meaningful genetic correlation can be recapitulated. Furthermore, these results indicate the potential for gene mechanisms whereby traits that have genetic correlations to be identified.

## Conclusions

In-depth analysis of multiomics data from 50 different bovine tissues, developmental stages, and cell types provided evidence to improve the annotation of thousands of protein-coding, lncRNA, and miRNA genes. These validated results increase the complexity of the bovine transcriptome (number of transcripts per gene, number of UTRs per gene, lncRNA transcripts, AS events, and miRNAs), comparable to that reported for the highly annotated human genome. The predicted unannotated transcripts extend existing annotated gene models, by verifying such extensions using independent WTTS-seq and RAMPAGE data. The integrated transcriptome data with publicly available QTL data revealed putative molecular pathways that may underlie tissue–tissue communication mechanisms and candidate genes responsible for the genetic mechanisms that may underlie genetic correlations between traits. This integrative approach is particularly important in the selection of indicator traits for breeding purposes, study of artificial selection side effects in livestock species, and functional annotation of poorly annotated livestock genomes.

## Methods

Tissue sample collection and sequencing library preparation methods are summarized in Supplemental File 23. The overview of the bioinformatics analysis steps is presented in Supplemental File 2: Fig. S39.

### RNA-seq data analysis and transcriptome assembly

Single-end Illumina RNA-seq reads (75 bp) from each tissue sample were trimmed to remove the adaptor sequences and low-quality bases using Trim Galore (RRID:SCR_011847) (version 0.6.4) [61] with –quality 20 and –length 20 option settings. The resulting reads were aligned against ARS-UCD1.2 bovine genome using STAR (RRID:SCR_004463) (version 020201) [62] with a cutoff of 95% identity and 90% coverage. FeatureCounts (RRID:SCR_012919) (version 2.0.2) [63] was used to quantify genes reported in the NCBI gene build (version 1.21) with -Q 255 -s 2 –ignoreDup –minOverlap 5 option settings. The resulting gene counts were adjusted for library size and converted to counts per million (CPM) values using SVA R package (version 3.30.0) [64]. In each tissue, sample similarities were checked using hierarchical clustering and regression analysis of gene expression values (log_2_-based CPM), and outlier samples were removed from downstream analysis. Samples from each tissue were combined to get the most comprehensive set of data in each tissue. To reduce the processing time due to huge sequencing depth, the trimmed reads were *in silico* normalized using insilico_read_normalization.pl from the Trinity package (RRID:SCR_013048) (version 2.6.6) [65] with –JM 350 G and –max_cov 50 option settings. Normalized RNA-seq reads were aligned against the ARS-UCD1.2 bovine genome using STAR (version 020201) [62] with a cutoff of 95% identity and 90% coverage. The normalized reads were assembled using *de novo* Trinity software (version 2.6.6) [65] combined with massively parallelized computing using HPCgridRunner (v1.0.1) [66] and GNU parallel software [67]. The resulted transcript reads were mapped against the ARS-UCD1.2 bovine genome using GMAP (RRID:SCR_008992) [68] with a cutoff of 95% identity and 90% coverage. In the next step, transcript reads were collapsed and grouped into putative gene models (clustering transcripts that had at least a 1-nucleotide overlap) by the pbtranscript-ToFU from SMRT Analysis software (v2.3.0) [69] with min-identity = 95%, min-coverage = 90% and max_fuzzy_junction = 15 nt, whereas the 5′-end and 3′-end differences were not considered when collapsing the reads. Base coverage of the resulting transcripts was calculated using mosdepth (RRID:SCR_018929) (version 0.2.5) [70]. Predicted transcripts were required to have a minimum of 3 times base coverage in their assembled tissues. The predicted acceptor and donor splice sites were required to be canonical and supported by Illumina-seq reads that spanned the splice junction with a 5-nt overhang. Spliced transcripts with the exact same splice junctions as their reference transcripts but that contained retained introns were removed from analysis, as they were likely pre-RNA sequences. Unspliced transcripts with a stretch of at least 20 As (allowing 1 mismatch) in a genomic window covering 30 bp downstream of their putative terminal site were removed from analysis, as they were likely genomic DNA contaminations. To decrease the false-positive rate, unspliced transcripts that were only expressed in a single tissue were removed from downstream analysis. In addition, single-exon genes without histone mark (H3K4me3, H3K4me1, H3K27ac) or ATAC-seq peaks mapped to their promoter (see Relating transcripts and genes to epigenetic data section) were removed from downstream analysis as they were likely transcriptional noise. The resulting transcripts from each tissue were regrouped into gene models using an in-house Python script. Structurally similar transcripts from the different tissues (see Comparison of transcript structures across datasets/tissues section) were collapsed using an in-house Python script to create the RNA-seq–based bovine transcriptome.

The resulting transcripts and genes were quantified using align_and_estimate_abundance.pl from the Trinity package (version 2.6.6) [65] with –aln_method bowtie –est_method RSEM –SS_lib_type R option settings. The quantified counts were normalized for sequencing depth using the RPKM method.

“Isoform” and “transcript” terms are used interchangeably throughout the article.

### PacBio Iso-seq data analysis

Publicly available PacBio Iso-seq reads and matched RNA-seq reads (PRJNA386670) were used in this study. In brief, a total of 6 tissues from L1 Dominette 01449 (aged 11 years) and testis from SuperBull 99375 (aged 9 years) were used in this experiment (Supplemental File 24). RNA was extracted using TRIzol reagent as directed by the manufacturer (Invitrogen) with integrity examined using a BioAnalyzer (Agilent). Libraries for RNA-seq short-read sequencing were prepared using the TruSeq RNA Kit following the “TruSeq RNA Sample Preparation v2 Guide” as recommended by the manufacturer (Illumina). RNA-seq libraries were sequenced on a NextSeq500 instrument. Iso-seq libraries for long-read sequencing were prepared using the SMRTbell Template Prep Kit 1.0. Complementary DNA (cDNA) was converted to the SMRTbell template library following the “Iso-Seq using Clontech cDNA Synthesis and BluePippin Size Selection” protocol as directed by the manufacturer (Pacific Biosciences). The sequences were processed into HQ isoforms using SMRT Analysis v6.0 for each tissue independently but with all size fractions within tissue included in the analysis.

PacBio Iso-seq data have been processed as described for the pig transcriptome [2] with the following exceptions. Errors in the full-length, nonchimeric (FLNC) cDNA reads were corrected with the preprocessed RNA-seq reads from the same tissue samples using the combination of proovread (RRID:SCR_017331) (v2.12) [71] and FMLRC (v1.0.0) [72] software packages. Error rates were computed as the sum of the number of bases of insertions, deletions, and substitutions in the aligned FLCN error-corrected reads divided by the length of aligned regions for each read (Table 8).

**Table 8:** Summary of error-corrected, FLNC Iso-seq reads and their matched RNA-seq reads

Tissue	Error-corrected FLNC Iso-seq reads*	Median error rate in error-corrected FLNC Iso-seq reads	Normalized RNA-seq reads used for error correction^†^
Thalamus	664,900 (90%)	0.21%	32,452,612
Testes	711,821 (86%)	1.43%	31,939,024
Liver	1,064,146 (84%)	1.84%	13,657,156
Medulla	380,531 (86%)	0.43%	48,256,918
Subcutaneous fat	215,759 (93%)	0.45%	42,043,313
Cerebral cortex	440,797 (87%)	1.01%	21,285,864
Jejunum	604,436 (90%)	2.331%	34,457,447

* Number in parentheses indicates mapping rate (90% coverage and 95% identity).

† *In silico* normalized using insilico_read_normalization.pl from Trinity (version 2.6.6) with the following settings: –max_cov 50 –max_pct_stdev 100 –single.

The RNA-seq–based transcriptome was assembled as described in the previous section.

### Oxford Nanopore data analysis

Assembled isoforms from a previously published Oxford Nanopore experiment were used in this study [12]. In brief, a total of 32 tissues (Supplemental File 24) from 2 male and 2 female Line 1 Hereford cattle, aged 14 months, were used in this experiment. Barcoded cDNAs extracted from frozen tissues (−80°C) were pooled at the University of California, Davis and sequenced using the Oxford Nanopore Technologies SQK-DCS109 kit according to the manufacturer’s protocol [12].

### Comparison of transcript structures across datasets/tissues

The structure of transcripts predicted from RNA-seq data was compared across tissues and independent datasets, including a library of annotated isoforms (Ensembl release 2021–03 and NCBI Release 106), as well as isoforms identified through complete isoform sequencing with Pacific Biosciences, a *de nov*o assembly produced from its matched RNA-seq reads, and isoforms identified from Oxford Nanopore platforms. Transcripts whose 5′ and 3′ borders were supported by RAMPAGE and/or WTTS data (see Transcript and gene border validation section) and whose splice junctions were identical (maximum fuzzy junction was set to 15 bp) were considered “structurally equivalent transcripts.” The maximum of 100 nt fuzzy 5′ and 3′ transcript borders were applied when comparing transcripts was not supported by RAMPAGE and/or WTTS data. Other transcripts that did not met these criteria were considered “structurally different transcripts.”

A pair of genes was considered structurally equivalent across datasets if they transcribed at least a single “structurally equivalent transcript.”

### Prediction of transcript and gene biotypes

Transcripts’ ORFs were predicted using the stand-alone version of ORFfinder [73] with “ATG and alternative initiation codons” as the ORF start codon. The longest 3 ORFs were matched to the Uniprot (RRID:SCR_002380) vertebrate database using Blastp (RRID:SCR_001010) [73] with an E-value cutoff of 10^− 6^, min coverage of 60%, and min identity of 95%. The ORFs with the lowest E-value to a protein were used as the representative, or if no matches were found, the longest ORF was used. Putative transcripts that had representative ORFs longer than 44 amino acids were labeled as protein-coding transcripts. If the representative ORF had a stop codon that was more than 50 bp upstream of the final splice junction, it was labeled as a nonsense-mediated decay transcript [74]. Transcripts with a start codon but no stop codon before their poly(A) site were labeled nonstop decay RNAs. Putative noncoding transcripts (ORFs shorter than 44 amino acids and lack of coding potential predicted by CPC2 [75]) with lengths less than 200 bp that did not overlap with annotated or unannotated miRNA precursors (see miRNA-seq data analysis section) were labeled as small noncoding RNAs [74]. Putative noncoding transcripts with lengths greater than 200 bp were labeled as long noncoding RNAs [74]. Long noncoding RNAs overlapping 1 or more coding loci on the opposite strand were labeled as antisense lncRNAs. Long noncoding RNAs located in introns of coding genes on the same strand were labeled as sense-intronic lncRNAs. Long noncoding RNAs that had an exon(s) that overlapped with a protein-coding gene were labeled as intragenic lncRNAs. Long noncoding RNAs located in intergenic regions of the genome were labeled as intergenic lncRNAs.

Putative genes that transcribed at least a single protein-coding transcript were labeled as protein-coding genes. Putative genes with homology to existing vertebrate protein-coding genes (Blastx [73], E-value cutoff 10^−6^, min coverage of 90%, and min identity of 95%) but containing a disrupted coding sequence (i.e., transcribe only nonsense-mediated decay or nonstop decay transcripts in all of their expressed tissues) were labeled as pseudogenes. The rest of the putative genes were labeled as noncoding.

### ncRNA homology analysis

Putative noncoding transcripts were matched to NCBI and Ensembl vertebrate ncRNA databases using Blastn (RRID:SCR_001598) [73] with an E-value cutoff of 10^− 6^, min coverage of 90%, and min identity of 95%. Transcripts with at least 1 hit were considered homologous ncRNAs.

### Transcriptome termini site sequencing data analysis

T-rich stretches located at the 5′ end of each WTTS-seq raw read were removed using an in-house Perl script, as described previously [76]. T-trimmed reads were error-corrected using Coral (version 1.4.1) [77] with -v -Y -u -a 3 option settings. The resulting reads with length greater than 300 nt were quality trimmed using the FASTX Toolkit (RRID:SCR_005534) (version 0.0.14) [78] with -q 20 and -p 50 option settings. High-quality, error-corrected WTTS-seq reads were aligned against the ARS-UCD1.2 bovine genome using STAR (version 020201) [62] with a cutoff of 95% identity and 90% coverage.

### Chromatin immunoprecipitation sequencing data analysis

Regions of signal enrichment (“peaks”) from a previously published chromatin immunoprecipitation sequencing (ChIP-seq) experiment were used in this study [79]. In brief, total 8 tissue (Supplemental File 24) from 2 male Line 1 Hereford cattle, aged 14 months, were used in this experiment. ChIP-seq experiments were performed on frozen tissue (−80°C) using the iDeal ChIP-seq kit for Histones (Diagenode, cat. C01010059) based on a protocol described in [79]. The following antibodies used were from Diagenode: H3K4me3 (in kit), H3K27me3 (#C15410069), H3K27ac (#C15410174), H3K4me1 (#C15410037), and CTCF (#15410210).

### ATAC-seq data analysis

The UC Davis FAANG Functional Annotation Pipeline was applied to process the ATAC-seq data, as previously described [79]. Briefly, the ARS-UCD1.2 genome assembly and Ensembl genome annotation (v100) were used as references for cattle. Sequencing reads were trimmed with Trim Galore! [61] (v.0.6.5) and aligned BWA [80]) (v0.7.17) to the ARS-UCD1.2 genome assembly with the –fr option. Alignments with mapping quality score (MAPQ) scores <30 were filtered using Samtools (RRID:SCR_005227) (v.1.9). Duplicate reads were marked and removed using Picard (RRID:SCR_006525) (v.2.18.7). Regions of signal enrichment were called by MACS2 (RRID:SCR_013291) (v.2.1.1).

### Relating transcripts and genes to epigenetic data

The promoter was defined as the genomic region that spans from 500 bp 5′ to 100 bp 3′ of the gene/transcript start site. Histone mark (H3K4me3, H3K4me1, H3K27ac), CTCF-DNA binding, or ATAC-seq peaks mapped to the promoter of a given gene/transcript were related to that gene/transcript.

### Transcript and gene border validation

RAMPAGE peaks from a previously published experiment [13] were used to validate gene/transcript start site (Supplemental File 24). Peaks within the genomic region spanning from 30 bp 5′ to 10 bp 3′ of a gene/transcript start site were assigned to that gene/transcript. WTTS-seq reads (median length of 161 bp) within the genomic region spanning from 10 bp 5′ to 165 bp 3′ of a gene/transcript terminal site were assigned to that gene/transcript.

### Functional enrichment analysis

The potential mechanism of action of a group of genes was deciphered using ClueGO (RRID:SCR_005748) [81]. The latest update (May 2021) of the Gene Ontology Annotation database (GOA) [82] was used in the analysis. The list of genes with at least 1 transcript expressed in a given tissue was used as background for that tissue. The GO tree interval ranged from 3 to 20, with the minimum number of genes per cluster set to 3. Term enrichment was tested with a right-sided hyper-geometric test that was corrected for multiple testing using the Benjamini–Hochberg procedure [83]. The adjusted *P* value threshold of 0.05 was used to filter enriched GO terms. Enriched GO terms were grouped based on kappa statistics [84].

### Alternative splicing analysis

Alternative splicing (AS) events (Supplemental File 2: Fig. S20A) are commonly distinguished in terms of whether RNA transcripts differ by inclusion or exclusion of an exon, in which case the exon involved is referred to as a “skipped exon” (SE) or “cassette exon,” “alternative first exon,” or “alternative last exon.” Alternatively, spliced transcripts may also differ in the usage of a 5′ splice site or 3′ splice site, giving rise to alternative 5′ splice site exons (A5Es) or alternative 3′ splice site exons (A3Es), respectively. A sixth type of alternative splicing is referred to as “mutually exclusive exons” (MXEs), in which 1 of 2 exons is retained in RNA but not both. However, these types are not necessarily mutually exclusive; for example, an exon can have both an alternative 5′ splice site and an alternative 3′ splice site, or have an alternative 5′ splice site or 3′ splice site, but be skipped in other transcripts. A seventh type of alternative splicing is “intron retention,” in which 2 transcripts differ by the presence of an unspliced intron in one transcript that is absent in the other. An eighth type of alternative splicing is “unique splice site exons” (USEs), in which 2 exons overlap with no shared splice junction. Alternative splicing events, except unique splice site exons, were detected using generateEvents from SUPPA (version 2.3) [85] with default settings. Unique splice site exons were detected using an in-house Python script.

### miRNA-seq data analysis

Single-end Qiagen miRNA-seq reads (50 bp) from each tissue sample were trimmed to remove the adaptor sequences and low-quality bases using Trim Galore (version 0.6.4) [61] with –quality 20, –length 16, –max_length 30 -a AACTGTAGGCACCATCAAT option settings. miRNA reads were aligned against the ARS-UCD1.2 bovine genome using mapper.pl from mirDeep2 (RRID:SCR_010829) (version 0.1.3) [86] with -e -h -q -j -l 16 -o 40 -r 1 -m -v -n option settings. miRNA mature sequences along with their hairpin sequences for *B. taurus* species were downloaded from miRbase [14]. These sequences, along with the aligned miRNA reads, were used to quantify annotated miRNAs in each sample using miRDeep2.pl from mirDeep2 (version 0.1.3) [86] with -t bta -c -v 2 setting options. miRNA normalized reads per million (RPM) were used to check sample similarities using hierarchical clustering and regression analysis of gene expression values (log_2_-based CPM). Outlier samples, which did not cluster together, indicating the potential for tissue mislabeling, were detected and removed from downstream analysis. In order to predict the most comprehensive set of unannotated miRNAs, samples from different tissues were concatenated into a single file that were aligned against the ARS-UCD1.2 bovine genome using mapper.pl from mirDeep2 (version 0.1.3) [86] with the aforementioned settings. Aligned reads from the previous step were used, along with annotated miRNAs’ mature sequences and their hairpins, to predict unannotated miRNAs using miRDeep2.pl from mirDeep2 (version 0.1.3) [86] with the aforementioned settings. Samples from each tissue were combined to get the most comprehensive set of data for that tissue. Mature miRNA sequences and their hairpins for both annotated and predicted unannotated miRNAs’ sequences along with the aligned miRNA reads from each tissue were used to quantify annotated and unannotated miRNAs in each tissue using mirDeep2 (version 0.1.3) [86] with the aforementioned settings.

### Tissue Specificity Index

Tissue Specificity Index (TSI) calculations were utilized to present more comprehensive information on transcript/gene/miRNA expression patterns across tissues. This index has a range of zero to 1 with a score of zero corresponding to ubiquitously expressed transcripts/genes/miRNAs (i.e., “housekeepers”) and a score of 1 for transcripts/genes/miRNAs that are expressed in a single tissue (i.e., “tissue specific”) [87]. The TSI for a transcript/gene/miRNA j was calculated as [87]


\begin{eqnarray*}
TS{I}_j = \frac{{\mathop \sum \nolimits_{i = 1}^N \left( {1 - {x}_{j,i}} \right)}}{{N - 1}}
\end{eqnarray*}

where *N* corresponds to the total number of tissues measured, and ${x}_{j,i}$ is the expression intensity of tissue *i* normalized by the maximal expression of any tissue for transcript/gene/miRNA *j*.

### QTL enrichment analysis

Publicly available bovine QTLs were retrieved from Animal QTLdb (RRID:SCR_001748) [88]. Closest expressed genes to a given trait’s QTLs were denoted as QTL-associated genes for that trait. The median distance of QTLs located outside gene borders to the closest expressed gene was 51.9 kilobases and the maximum distance was 2.6 million bases. QTL enrichment was tested with a right-sided Fisher exact test using an in-house Python script. The resulting *P* values were corrected for multiple testing by the Benjamini–Hochberg procedure [83]. The adjusted *P* value threshold of 0.05 was used to filter QTLs.

### Trait similarity network

For a given pair of traits, trait A was denoted as “similar” to trait B if a significant portion of trait A’s QTL-associated genes were also the closest expressed genes to trait B’s QTLs based on 1,000 permutation tests. The resulting *P* values were corrected for multiple testing using the Benjamini–Hochberg procedure [83]. The same procedure was used to test trait B’s similarity to trait A. The adjusted *P* value threshold of 0.05 was used to filter significant trait similarities. A graphical presentation of the method used to construct the tissue similarity network is presented in Supplemental File 2: Fig. S40. The resulting network was visualized using Cystoscape software [89].

### Testis–pituitary axis correlation significance test

The presence of signal peptides on representative ORFs of protein-coding transcripts was predicted using SignalP-5.0 [90]. Spearman correlation coefficients were used to study expression similarity between testis genes encoding signal peptides that were closest to the “percentage of normal sperm” QTLs (62 genes) and pituitary expressed genes closest to the “percentage of normal sperm” QTLs (246 genes). To test the statistical difference between these correlation coefficients (reference correlations) and random chance, 1,000 random sets of 246 pituitary genes were selected, and their correlation coefficients with 62 previously described testis genes were calculated (random correlations). The reference correlations were compared with 1,000 sets of random correlations using a right-sided *t*-test. The resulting *P* values were corrected for multiple testing by the Benjamini–Hochberg procedure [83]. The distribution-adjusted *P* values were used to determine the significance level of expression similarities for genes involved in the testis–pituitary axis related to “percentage of normal sperm.” The same analysis was conducted to determine the significance of pituitary–testis axis involvement in this trait.

### Tissue dendrogram comparison across different transcript and gene biotypes

Tissues were clustered based on the percentage of their transcripts/genes that were shared between tissue pairs using the hclust function in R. Cophenetic distances for tissue dendrograms were calculated using the cophenetic R function. The degree of similarity between dendrograms constructed based on different gene/transcript biotypes was obtained using the Spearman correlation coefficient between the dendrograms’ Cophenetic distances.

## Supplementary Material

giae019_GIGA-D-23-00037_Original_Submission

giae019_GIGA-D-23-00037_Revision_1

giae019_GIGA-D-23-00037_Revision_2

giae019_Response_to_Reviewer_Comments_Original_Submission

giae019_Response_to_Reviewer_Comments_Revision_1

giae019_Reviewer_1_Report_Original_Submission Yang Zhou -- 4/12/2023 Reviewed

giae019_Reviewer_2_Report_Original_Submission Pablo Augusto de Souza Fonseca -- 4/20/2023 Reviewed

giae019_Reviewer_2_Report_Revision_1 Pablo Augusto de Souza Fonseca -- 9/5/2023 Reviewed

giae019_Reviewer_3_Report_Original_Submission Christopher Hunter, Ph.D. -- 5/2/2023 Reviewed

giae019_Reviewer_3_Report_Revision_1 Christopher Hunter, Ph.D. -- 9/6/2023 Reviewed

giae019_Supplemental_Files

## Data Availability

RNA-seq and miRNA-seq, ATAC-seq, and WTTS-seq datasets generated in this study have been submitted to the ArrayExpress database [92] under accession numbers E-MTAB-11699, E-MTAB-11815, and E-MTAB-12052, respectively. The constructed bovine trait similarity network is publicly available through the Animal Genome database [93]. The constructed cattle transcriptome and related sequences are publicly available in the Open Science Framework database [94]. Bioinformatics work-follow and custom codes used are available in the GitHub repository [95]. In addition, bioinformatics_workfloow.sh contains all bioinformatics work-follow used in this project. All additional supporting data are available in the *GigaScience* repository, GigaDB [96].

## Additional Files

**Supplemental File 1**. List of different datasets generated in the experiment.

**Supplemental File 2**. Fig. S1. Distribution of the number of RNA-seq reads across tissues. Fig. S2. (A) Comparison of tissues based on number of transcript biotypes and (B) percentage of transcript biotypes. (C) Comparison of transcript biotypes based on their number of expressed tissues and (D) their expression level across expressed tissues. Fig. S3. (A) Relation between the number of input reads and the number of transcript biotypes. (B) Comparison of expression level between different transcript biotypes. Fig. S4. Tissue similarities (A) and clustering (B) based on the percentage of protein-coding transcripts shared between pairs of tissues. Fig. S5. Tissue similarities (A) and clustering (B) based on the percentage of noncoding transcripts shared between pairs of tissues. Fig. S6. Comparison of annotated and unannotated transcripts based on their expression (A) and number of expressed tissues (B). Fig. S7. Comparison of annotated and unannotated protein-coding transcripts based on the length (A) and GC content (B) of their 5′ UTR, CDS, and 3′ UTR. Fig. S8. (A) Comparison of tissues based on number of gene biotypes and (B) percentage of gene biotypes. Fig. S9. Relation between the number of input reads and the number of gene biotypes. Fig. S10. Comparison of annotated and unannotated genes based on their expression (A) and number of expressed tissues (B). Fig. S11. Functional enrichment analysis of the top 5% of genes with the highest number of UTRs. Fig. S12. Similarity of tissues based on the number of noncoding genes in their fetal samples that switched to protein-coding genes with only coding transcripts in their adult samples. Fig. S13. (A) Distribution of genes that transcribed PATs, based on their number of expressed tissues, percentage of genes’ transcripts that are PATs, and percentage of genes’ expressed tissues in which PATs were transcribed. (B) Comparison of genes that transcribed PATs with other gene biotypes. Fig. S14. (A) Homology analysis of protein-coding genes. (B) Homology analysis of noncoding genes. (C) Detection of orphan genes based on homology classification of cattle-specific protein-coding genes and noncoding genes. Fig. S15. Comparison of the expression level of homologous and orphan genes across (A) and within (B) their expressed tissues. (C) Comparison of homologous and orphan genes based on the number of expressed tissues. Fig. S16. Comparison of different gene biotypes based on the expression (A) and the number of expressed tissues (B). Fig. S17. Comparison of different pseudogene-derived lncRNAs and non-pseudogene-derived lncRNAs based on the expression level (A) and the number of expressed tissues (B). Fig. S18. Tissue similarities (A) and clustering (B) based on the percentage of protein-coding genes shared between pairs of tissues. Fig. S19. Tissue similarities (A) and clustering (B) based on the percentage of noncoding genes shared between pairs of tissues. Fig. S20. (A) Different types of alternative splicing events. (B) Comparison of bovine genome builds based on the number of transcripts that showed any type of alternative splicing (AS) events. Fig. S21. Comparison of tissues based on the number (A) and the percentage (B) of transcripts that showed different types of alternative splicing events. Comparison of tissues based on the number (C) and the percentage (D) of alternative splicing events. Fig. S22. (A) Comparison of tissues based on the percentage of transcripts that showed any type of alternative splicing events, spliced transcripts from single-transcript genes, and unspliced transcripts and (B) the relation between the number of input reads and the number of these transcripts across tissues. Fig. S23. Comparison of transcripts that showed different types of alternative splicing events based on (A) the expression level in the expressed tissues and (B) the number of expressed tissues. Fig. S24. Transcript biotype switching due to alternative splicing events. Fig. S25. Comparison of tissues based on the number of alternative splicing events per alternatively spliced gene. Fig. S26. (A) Distribution of the number of alternative splicing events per alternatively spliced gene. The 5% quantile is shown using a dashed red line. (B) Functional enrichment analysis of the top 5% of genes with the highest number of alternative splicing events. Fig. S27. Comparison of the alternative splicing rate between adult and fetal tissues. Fig. S28. (A) Distribution of a gene’s number of expressed tissues. Tissue-specific gene biotypes are shown in the pie chart. (B) Distribution of a transcript’s number of expressed tissues. Tissue-specific transcript biotypes are shown in the pie chart. (C) Comparison of tissues based on the number of tissue-specific genes and transcripts. (D) Comparison of the expression level of tissue-specific genes and transcripts versus their non-tissue-specific counterparts. Fig. S29. Relationship between tissue specificity and alternative splicing events. Fig. S30. Relationship between Tissue Specificity Index and the number of multitissue expressed genes (A) and transcripts (B). Distribution of Tissue Specificity Iindexes in multitissue expressed genes (C) and transcripts (D). The 5% quantile is shown using dashed red lines. (E) Functional enrichment analysis of the top 5% of multitissue expressed genes with the highest Tissue Specificity Indexes. Fig. S31. Distribution of QTLs located outside gene borders in relation to the closest expressed gene. Fig. S32. (A) Distribution of correlation coefficients between *SPACA5* gene expression and pituitary expressed genes closest to “percentage of normal sperm” QTLs. Dashed lines show the minimum significant positive and negative correlation (*P* < 0.05). (B) Expression atlas of *SPACA5* gene in human tissues from the Human Protein Atlas [91]. Fig. S33. (A) Correlation between pituitary genes with signal peptides that were close to the “percentage of normal sperm” QTL and testis expressed genes closest to this trait’s QTL (reference correlations). (B) Distribution of *P* values resulting from right-sided *t*-test between reference correlation coefficients and correlation coefficients derived from random chance (see Methods for details). Fig. S34. Tissue similarities (A) and clustering (B) based on the percentage of miRNAs shared between pairs of tissues. Fig. S35. Clustering of tissues based on protein-coding genes (A), protein-coding transcripts (B), noncoding genes (C), noncoding transcripts (D), and miRNAs (E). (F) Comparison of tissue dendrograms based on the correlation between their Cophenetic distances. Fig. S36. (A) Distribution of the number of expressed tissues for annotated and unannotated miRNAs. Classification of miRNAs as annotated or unannotated is presented in the pie chart. (B) Comparison of tissues based on their number of tissue-specific miRNAs. (C) Expression of annotated and unannotated miRNAs in their expressed tissues. (D) Distribution of multitissue expressed miRNAs’ Tissue Specificity Indexes. (E) Relationship between Tissue Specificity Index and number of expressed tissues in multitissue expressed miRNAs. Dots have been color coded based on their density. Fig. S37. Distribution of the number of expressed genes (A), transcripts (B), and miRNAs (C) across tissues. Fig. S38. Distribution of the number of annotated and unannotated genes (A), transcripts (B), and miRNAs (C) across tissues. Fig. S39. Overview of the bioinformatics steps used in this study. Fig. S40. Graphical representation of the method used to construct the tissue similarity network.

**Supplemental File 3**. Summary of RNA-seq and miRNA-seq reads.

**Supplemental File 4**. Detailed description of the number of transcripts, genes, and miRNAs expressed in each tissue.

**Supplemental File 5**. List of transcripts and genes expressed in each tissue and their expression values (RPKM). Individual tissue files are labeled as Supplemental_file5_<TISSUE NAME>_<Genes/Transcripts>.tsv.

**Supplemental File 6**. Transcript biotype enrichment analysis in adult and fetal tissues.

**Supplemental File 7**. Functional enrichment analysis of the top 5% of genes with the highest number of UTRs.

**Supplemental File 8**. Functional enrichment analysis of genes that remained bifunctional in all their expressed tissues.

**Supplemental File 9**. Functional enrichment analysis of noncoding genes in fetal tissues that were switched to protein coding with only coding transcripts in their matched adult tissue.

**Supplemental File 10**. Functional enrichment analysis of protein-coding genes that transcribed PATs as their main transcripts (PATs comprised >50% of their transcripts) in all their expressed tissues.

**Supplemental File 11**. Gene biotype enrichment analysis in adult and fetal tissues.

**Supplemental File 12**. Functional enrichment analysis of the top 5% of genes with the highest number of alternative splicing events.

**Supplemental File 13**. List of tissue-specific genes and transcripts.

**Supplemental File 14**. Genes’ and transcripts’ Tissue Specificity Indexes. Individual tissue files are labeled as Supplemental_file14_<Genes/Transcripts>.tsv.

**Supplemental File 15**. Functional enrichment analysis of the top 5% of multitissue expressed genes with the highest Tissue Specificity Indexes.

**Supplemental File 16**. List of QTLs’ closest expressed genes in each tissue. Individual tissue files are labeled as Supplemental_file16_<TISSUE NAME>.tsv.

**Supplemental File 17**. Trait enrichment analysis of testis-specific genes.

**Supplemental File 18**. Pituitary expressed genes closest to “percentage of normal sperm” QTLs that showed positive significant correlation with *SPACA5* gene in testis.

**Supplemental File 19**. List of expressed genes closest to “percentage of normal sperm” QTLs that were involved in testis–pituitary tissue axis and their functional enrichment analysis results.

**Supplemental File 20**. List of genes expressed closest to “percentage of normal sperm” QTLs that were involved in pituitary–testis tissue axis and their functional enrichment analysis results.

**Supplemental File 21**. Similarity of traits based on the integration of the assembled bovine transcriptome with publicly available QTLs.

**Supplemental File 22**. List of miRNAs expressed in each tissue and their expression values. Individual tissue files are labeled as Supplemental_file22_<TISSUE NAME>.tsv.

**Supplemental File 23**. Tissue sample collection and sequencing library preparation methods.

**Supplemental File 24**. List of independent omics datasets used in the experiment.

## Abbreviations

A3E: alternative 3′ splice site exon; A5E: alternative 5′ splice site exon; AFE: alternative first exon; ALE: alternative last exon; AS: alternative splicing; ATAC-seq: assay for transposase-accessible chromatin using sequencing; bp: base pair; BP: Biological Process; CDS: coding sequence; ChIP-seq: chromatin immunoprecipitation sequencing; CPM: counts per million; CTCF: CCCTC-binding factor; DMEM: Dulbecco’s modified Eagle medium; FLNC: full-length, nonchimeric; GO: Gene Ontology; GOA: Gene Ontology Annotation database; GWAS: genome-wide association study; H3K27ac: N-terminal acetylation of lysine 27 on histone H3; H3K4me1: trimethylation of lysine 4 on histone H1; H3K4me3: trimethylation of lysine 4 on histone H3; IACUC: Institutional Animal Care and Use Committee; LD: longissimus dorsi; lncRNA: long noncoding RNA; MAPQ: mapping quality; miRNA: microRNA; MXE: mutually exclusive exon; NCBI: National Center for Biotechnology Information; ncRNA: noncoding RNA; NMD: nonsense-mediated decay; NSD: nonstop decay; ONT-seq: Oxford Nanopore Technologies sequencing; ORF: open reading frame; PacBio Iso-seq: Pacific Biosciences single-molecule long-read isoform sequencing; PAT: potentially aberrant Transcript; poly(A): polyadenylation; PTBP1: polypyrimidine tract binding protein 1; QTL: quantitative trait loci; RAMPAGE: RNA Annotation and Mapping of Promoters for the Analysis of Gene Expression; Ribo-seq: ribosome footprinting followed by sequencing; RIE: retained intron exon; RNA-seq: Illumina high-throughput RNA sequencing; RPKM: reads per kilobase of transcript per million reads mapped; RPM: reads per million; SE: skipped exon; sncRNA: small noncoding RNA; SNP: single nucleotide polymorphism; tpg: transcripts per annotated gene; TSI: Tissue Specificity Index; TSS: transcript start site; TTS: transcript terminal site; UCD: University of California, Davis; USE: unique splice site exon; UTR: untranslated region; WTTS-seq: whole transcriptome termini site sequencing.
