# Supplementary material for: Enhanced bovine genome annotation through integration of transcriptomics and epi-transcriptomics datasets facilitates genomic biology
Source: Gigascience. 2024 Apr 16;13:giae019. doi: 10.1093/gigascience/giae019 (PMC11020238; doi:10.1093/gigascience/giae019)
Supplement: giae019_GIGA-D-23-00037_Revision_2 [file giae019_giga-d-23-00037_revision_2.pdf]

// GigaScience

Enhancing Bovine Genome AnnotationThrough Integration of Transcriptomics and Epi-Transcriptomics Datasets Facilitates Genomic Biology

--Manuscript Draft--

**Manuscript Number:**	GIGA-D-23-00037R2
**Full Title:**	Enhancing Bovine Genome AnnotationThrough Integration of Transcriptomics and Epi-Transcriptomics Datasets Facilitates Genomic Biology
**Article Type:**	Research
**Funding Information:**	National Institute of Food and Agriculture (2018-67015-27500) — Dr Huaijun Zhou<br>National Institute of Food and Agriculture (2015-67015-22940) — Dr Huaijun Zhou
**Abstract:**	Background<br><br>The accurate identification of the functional elements in the bovine genome is a fundamental requirement for high quality analysis of data informing both genome biology and genomic selection. Functional annotation of the bovine genome was performed to identify a more complete catalogue of transcript isoforms across bovine tissues.<br><br>Results<br><br>A total number of 160,820 unique transcripts (50% protein-coding) representing 34,882 unique genes (60% protein-coding) were identified across tissues. Among them, 118,563 transcripts (73% of the total) were structurally validated by independent datasets (PacBio Iso-seq data, ONT-seq data, de novo assembled transcripts from RNA-seq data) and comparison with Ensembl and NCBI gene sets. In addition, all transcripts were supported by extensive data from different technologies such as WTTS-seq, RAMPAGE, ChIP-seq, and ATAC-seq. A large proportion of identified transcripts (69%) were un-annotated, of which 86% were produced by annotated genes and 14% by un-annotated genes. A median of two 5’ untranslated regions were expressed per gene. Around 50% of protein-coding genes in each tissue were bifunctional and transcribed both coding and noncoding isoforms. Furthermore, we identified 3,744 genes that functioned as non-coding genes in fetal tissues, but as protein coding genes in adult tissues. Our new bovine genome annotation extended more than 11,000 annotated gene borders compared to Ensembl or NCBI annotations. The resulting bovine transcriptome was integrated with publicly available QTL data to study tissue-tissue interconnection involved in different traits and construct the first bovine trait similarity network.<br><br>Conclusions<br><br>These validated results show significant improvement over current bovine genome annotations.
**Corresponding Author:**	James Reecy<br>Iowa State University<br>Ames, IA UNITED STATES
**Corresponding Author Secondary Information:**	
**Corresponding Author's Institution:**	Iowa State University
**Corresponding Author's Secondary Institution:**	

Powered by Editorial Manager® and ProduXion Manager® from Aries Systems Corporation

**First Author:**	Hamid Beiki
**First Author Secondary Information:**	
**Order of Authors:**	Hamid Beiki
	Brenda M. Murdoch
	Carissa A. Park
	Chandlar Kern
	Denise Kontechy
	Gabrielle Becker
	Gonzalo Rincon
	Honglin Jiang
	Huaijun Zhou
	Jacob Thorne
	James E. Koltes
	Jennifer J. Michal
	Kimberly Davenport
	Monique Rijnkels
	Pablo J. Ross
	Rui Hu
	Sarah Corum
	Stephanie McKay
	Timothy P.L. Smith
	Wansheng Liu
	Wenzhi Ma
	Xiaohui Zhang
	Xiaoqing Xu
	Xuelei Han
	Zhihua Jiang
	Zhi-Liang Hu
	James Reecy
**Order of Authors Secondary Information:**	
**Response to Reviewers:**	We have uploaded a response to reviewer comment document with the rest of the manuscript files. In it, we addressed all review comments point by point.<br><br>One item of note, we have submitted all of the requested changes to GigaDB, but we have not seen that the changes have been made.<br><br>All the best,<br><br>Jim Reecy
**Additional Information:**	
**Question**	**Response**
Are you submitting this manuscript to a special series or article collection?	No

Powered by Editorial Manager® and ProduXion Manager® from Aries Systems Corporation

**Experimental design and statistics**<br><br>Full details of the experimental design and statistical methods used should be given in the Methods section, as detailed in our Minimum Standards Reporting Checklist. Information essential to interpreting the data presented should be made available in the figure legends.<br><br>Have you included all the information requested in your manuscript?	Yes
**Resources**<br><br>A description of all resources used, including antibodies, cell lines, animals and software tools, with enough information to allow them to be uniquely identified, should be included in the Methods section. Authors are strongly encouraged to cite Research Resource Identifiers (RRIDs) for antibodies, model organisms and tools, where possible.<br><br>Have you included the information requested as detailed in our Minimum Standards Reporting Checklist?	Yes
**Availability of data and materials**<br><br>All datasets and code on which the conclusions of the paper rely must be either included in your submission or deposited in publicly available repositories (where available and ethically appropriate), referencing such data using a unique identifier in the references and in the "Availability of Data and Materials" section of your manuscript.<br><br>Have you have met the above requirement as detailed in our Minimum Standards Reporting Checklist?	Yes

Manuscript

Click here to access/download;Manuscript;Manuscript_2nd_revision.docx

Enhanced Bovine Genome Annotation Through Integration of Transcriptomics and Epi-Transcriptomics Datasets Facilitates Genomic Biology

Hamid Beiki[1], Brenda M. Murdoch[2], Carissa A. Park[1], Chandlar Kern[3], Denise Kontechy[2], Gabrielle Becker[2], Gonzalo Rincon[4], Honglin Jiang[5], Huaijun Zhou[6], Jacob Thorne[2], James E. Koltes[1], Jennifer J. Michal[7], Kimberly Davenport[2], Monique Rijnkels[8], Pablo J. Ross[6], Rui Hu[5], Sarah Corum[4], Stephanie McKay[9], Timothy P.L. Smith[10], Wansheng Liu[3], Wenzhi Ma[3], Xiaohui Zhang[7], Xiaoqing Xu[6], Xuelei Han[7], Zhihua Jiang[7], Zhi-Liang Hu[1], James M. Reecy[1]

[1]Department of Animal Science, Iowa State University; [2]Department of Animal and Veterinary and Food Science, University of Idaho; [3]Department of Animal Science, Pennsylvania State University; [4]Zoetis; [5]Department of Animal and Poultry Sciences, Virginia Tech; [6]Department of Animal Science, University of California, Davis; [7]Department of Animal Science, Washington State University; [8]Department of Veterinary Integrative Biosciences, Texas A&M University; [9]University of Vermont; [10]USDA, ARS, USMARC.

Hamid Beiki [0000-0002-0516-1431]; Brenda M Murdoch [0000-0001-8675-3473]; Carissa A Park [0000-0002-2346-5201]; Chandlar Kern [0000-0003-3343-1598]; Denise Kontechy [0000-0002-9634-2421]; Gabrielle Becker [0000-0002-1455-6443]; Gonzalo Rincon [0000-0002-6149-9103]; Honglin Jiang [0000-0001-9540-5788]; Huaijun Zhou [0000-0001-6023-9521]; Jacob Thorne [0000-0003-3553-7628]; James E Koltes [0000-0003-1897-5685]; Jennifer J Michal

[0000-0002-4638-4156]; Kimberly Davenport [0000-0003-2796-9252]; Monique Rijnkels [0000-0002-8156-3651]; Pablo J Ross [0000-0002-3972-3754]; Stephanie McKay [0000-0003-1434-3111]; Timothy P L Smith [0000-0003-1611-6828]; Wansheng Liu [0000-0003-1788-7093]; Wenzhi Ma []; Xiaohui Zhang [0000-0002-6658-9589]; Xuelei Han [0000-0002-7957-0297]; Zhihua Jiang [0000-0003-1986-088X]; Zhi-Liang Hu [0000-0002-6704-7538]; James Reecy [0000-0003-4602-0990

Corresponding author:

James M. Reecy

Professor of Animal Breeding and Genetics, Department of Animal Science, Ames, IA, USA

jreecy@iastate.edu

Abstract

Background

The accurate identification of the functional elements in the bovine genome is a fundamental requirement for high quality analysis of data informing both genome biology and genomic selection. Functional annotation of the bovine genome was performed to identify a more complete catalogue of transcript isoforms across bovine tissues.

Results

A total number of 160,820 unique transcripts (50% protein-coding) representing 34,882 unique genes (60% protein-coding) were identified across tissues. Among them, 118,563 transcripts (73% of the total) were structurally validated by independent datasets (PacBio Iso-seq data, ONT-seq data, *de novo* assembled transcripts from RNA-seq data) and comparison with Ensembl and NCBI gene sets. In addition, all transcripts were supported by extensive data from different technologies such as WTTS-seq, RAMPAGE, ChIP-seq, and ATAC-seq. A large proportion of identified transcripts (69%) were un-annotated, of which 86% were produced by annotated genes and 14% by un-annotated genes. A median of two 5' untranslated regions were expressed per gene. Around 50% of protein-coding genes in each tissue were bifunctional and transcribed both coding and noncoding isoforms. Furthermore, we identified 3,744 genes that functioned as non-coding genes in fetal tissues, but as protein coding genes in adult tissues. Our new bovine genome annotation extended more than 11,000 annotated gene borders compared to Ensembl or NCBI annotations. The resulting bovine transcriptome was

integrated with publicly available QTL data to study tissue-tissue interconnection involved in different traits and construct the first bovine trait similarity network.

Conclusions

These validated results show significant improvement over current bovine genome annotations.

Introduction

Domestic bovine (*Bos taurus*) provide a valuable source of nutrition and an important disease model for humans [1]. Furthermore, cattle have the greatest number of genotype associations and genetic correlations of the domesticated livestock species, which means they provide an excellent model to close the genotype-to-phenotype gap. Furthermore, the functional elements of genome provide a means whereby complex biological pathways responsible for variation in a particular phenotype can be identified. Therefore, the accurate identification of these elements in the bovine genome is a fundamental requirement for high quality analysis of data from which both genome biology and genomic selection can be better understood.

Current annotations of farm animal genomes largely focus on the protein-coding regions [2] and fall short of explaining the biology of many important traits that are controlled at the transcriptional level [3-5]. In humans, 93% of trait-associated single nucleotide polymorphisms (SNP) identified by genome-wide association studies (GWAS) are found in non-coding regions [6]. Therefore, elucidating non-coding functional elements of the genome is essential for understanding the mechanisms that control complex biological processes.

Untranslated regions play critical roles in the regulation of mRNA stability, translation, and localization [7], but these regions have been poorly annotated in farm animals [2, 8]. A recent study of the pig transcriptome using single-molecule long-read isoform sequencing technology resulted in the extension of more than 6000 annotated gene borders compared to Ensembl or National Center for Biotechnology Information (NCBI) annotations [2].

Small non-coding RNAs, such as microRNAs (miRNA), are known to be involved in gene regulation through post-transcriptional regulation of expression via silencing, degradation, or sequestering to inhibit translation [9-11]. The number of annotated miRNAs in the current bovine genome annotation (Ensembl release 2018-11; 951 miRNAs) is much lower than the number reported in the highly annotated human genome (Ensembl release 2021-03; 1,877 miRNAs).

This study used a comprehensive set of transcriptome and chromatin state data from 50 cattle tissues and cell types to (1) increase the complexity of the bovine transcriptome, comparable to that reported for the highly annotated human genome, (2) improve the annotation of protein-coding, non-coding, and miRNA genes, (3) integration of transcriptome data with publicly available Quantitative Trait Loci (QTL) and gene association data to study tissue-tissue interconnection involved in different traits, and 4) construction the first bovine trait similarity network that recapitulates published genetic correlations.

Results

The diversity of RNA and miRNA transcript among 50 different bovine tissues, developmental stages, and cell types was assessed using polyadenylation (poly(A)) selected Illumina high-

throughput RNA sequencing (RNA-seq) data (47) and/or miRNA-seq (46) and data (Supplemental file 1). Most of the tissues studied were from Hereford cattle closely related to L1 Dominette 01449, the individual from which the bovine reference genome (ARS-UCD1.2) was sequenced. The 50 tissues and cell samples included follicular cells, myoblasts, 14 mammary gland samples from various stages of mammary gland development and lactation, eight fetal tissues (78-days of gestation), eight tissues from adult digestive tract, and 16 other adult organs (Supplemental file 1). A total of approximately 4.1 trillion RNA-seq reads and 1.2 billion miRNA-seq reads were collected, with a minimum of 27.5 million RNA-seq and 9.3 million miRNA-seq reads from each tissue/cell type (average 87.8 ± 49.7 million and 27.6 ± 12.9 million, respectively) (Supplemental file 2: Fig. S1 and Supplemental file 3).

Transcript-based analyses

The summary of predicted transcript/genes is presented in Table 1. All of the predicted splice junctions across tissues were supported by RNA-seq reads that spanned the splice junction, substantiating the accuracy of the transcript definition from RNA-seq reads.

A total of 31,476 transcripts appeared tissue-specific by virtue of being assembled from RNA-seq reads in just a single tissue, but 20,100 of those transcripts (64%) were actually expressed in multiple tissues. Thus, reliance solely on assembled transcripts in a given tissue to predict a tissue transcript atlas may overestimate tissue specificity due to a high false-negative rate for transcript detection. To solve this problem of over-prediction of tissue specificity, we marked a transcript as “expressed” in a given tissue only if (1) it had been assembled from RNA-seq data in that tissue; or (2) its expression and all of its splice junctions has been quantified using RNA-

seq reads in the tissue of interest with an expression level more than 1 reads per kilobase of transcript per Million reads mapped (RPKM) (see Methods section). This resulted in 145,258 transcripts (90%) expressed in more than one tissue (Fig. 1), among which 9,024 transcripts (5%) were found in all 47 tissues examined.

The unique transcripts identified were equally distributed between protein-coding transcripts and non-coding transcripts (ncRNAs) (Fig. 2). Non-coding transcripts were further classified as long non-coding RNAs (lncRNAs), nonsense-mediated decay (NMD) transcripts, non-stop decay (NSD) transcripts, and small non-coding RNAs (sncRNAs). While the majority of expressed transcripts in each tissue were protein coding (median of 62% of tissue transcripts), NMD transcripts and antisense lncRNAs each made up more than 10% of the transcripts (Supplemental file 2: Fig. S2A and B, Supplemental file 4 and 5). Fetal muscle and fetal gonad tissues showed the highest proportion of antisense lncRNAs compared to that observed in other tissues, and around 60% of antisense lncRNAs were expressed from these two tissues (Supplemental file 2: Fig. S2B). Compared to non-coding transcripts, protein-coding transcripts were more likely to have spliced exons (p-value < 2.2e-16) and were expressed in a higher number of tissues (p-value < 2.2e-16; Additional file1: Fig. S2C).

There were no significant correlations between the number of RNA-seq reads for a given tissue and the number of transcripts identified, except for a modest correlation for the antisense lncRNA class (Supplemental file 2: Fig. S3A). There was a significant positive correlation (p-value 1.3e-04) between the number of NMD transcripts in a tissue and the number of protein-coding transcripts, and the NMD transcript class showed the lowest median expression level across tissues compared to other transcript biotypes (Supplemental file 2: Fig. S2D and Fig. S3B).

Transcript similarity to other species

Protein/peptide homology analysis of transcripts with an open reading frame (protein-coding transcripts, lncRNAs, and sncRNAs) revealed a higher conservation of protein-coding transcripts compared to lncRNA and sncRNA transcripts (p-value < 2.2e-16) (Table 2). Bovine non-coding transcripts had significantly (p-value < 2.2e-16) less similarity to other species than protein-coding transcripts (Table 2 and Table 3). Within non-coding transcripts, sense intronic lncRNAs showed the highest conservation rate (Table 4).

Transcript expression diversity across tissues

A median of 70% of protein-coding transcripts were shared between pairs of tissues (Supplemental file 2: Fig. S4A), was significantly higher than that was observed for non-coding transcripts (53%; p-value < 2.2e-16; Supplemental file 2: Fig. S5). Clustering of tissues based on protein-coding transcripts was different than that observed based on non-coding transcripts (Supplemental file 2: Fig. S4B and Fig. S5B, Fig. S35F). The fetal tissues clustered together and were generally more similar to one another than to the corresponding adult tissue in both dendrograms. In addition, fetal tissues had significantly higher proportions of non-coding transcripts compared to protein-coding transcripts (p-value < 2.2e-16; Supplemental file 6).

Transcript validation

Prediction of transcripts and isoforms from RNA-seq data may produce erroneous predicted isoforms. The validity of transcripts was therefore examined by comparison to a library of isoforms taken from Ensembl (release 2021-03) and NCBI gene sets (Release 106), as well as isoforms identified through complete isoform sequencing with Pacific Biosciences, a de novo

assembly produced from its matched RNA-seq reads, and isoforms identified from Oxford Nanopore platforms (see Methods section). A total of 118,563 transcripts (73% of predicted transcripts) were structurally validated by independent datasets (Biosciences single-molecule long-read isoform sequencing (PacBio Iso-Seq), Oxford Nanopore Technologies sequencing ONT-seq) data, *de novo* assembled transcripts from RNA-seq data) and comparison with Ensembl and NCBI gene sets. A total of 145,258 transcripts were expressed in multiple tissues (90% of predicted transcripts), providing further support for their validity (Fig. 3). All transcripts were also extensively supported by data from different technologies such as Whole Transcriptome Termini Site Sequencing (WTTS-seq), RNA Annotation and Mapping of Promoters for the Analysis of Gene Expression (RAMPAGE), histone modification (H3K4me3, H3K4me1, H3K27ac), CTCF-DNA binding, and Assay for Transposase-Accessible Chromatin using sequencing (ATAC-seq) (Fig. 3).

Comparison of predicted transcript structures with annotated transcripts in the current bovine genome annotations (Ensembl release 2021-03 and NCBI Release 106) resulted in a total of 48,906 annotated transcripts that exactly matched previously annotated transcripts (30% of all transcripts), including 44,097 annotated NCBI transcripts, 29,179 annotated Ensembl transcripts, and 24,370 transcripts that were common to both annotated gene sets (Fig. 3). The median expression level of annotated transcripts in their expressed tissues was similar to that observed for un-annotated transcripts (Supplemental file 2: Fig. S6). Annotated transcripts were expressed in higher number of tissues than that observed for un-annotated transcripts (p-value 7.4e-03; Supplemental file 2: Fig. S6). In addition, compared to un-annotated transcripts,

annotated transcripts were enriched with protein-coding (p-value 1.37e-02) and spliced transcripts (p-value 3.76e-02).

The median length of coding sequence (CDS) of annotated transcripts was significantly longer than that observed in un-annotated transcripts (p-value 0.0) (Additional file1: Fig. S7A). In addition, un-annotated transcripts had longer 5' untranslated regions (UTR) compared to annotated transcripts (p-value 2.631E-06; Additional file1: Fig. S7A). Annotated protein-coding transcripts showed a higher GC content in their 5' UTRs than un-annotated transcripts (p-value 5.562E-18), but both classes of transcripts showed similar GC content within their CDS (Supplemental file 2: Fig. S7B).

Gene-based analyses

The transcripts correspond to a total of 34,882 genes, which were classified into protein coding, non-coding, and pseudogenes (Supplemental file 4 and 5, and Fig. 4). Genes transcribed at least a single "expressed" transcript (see Transcript level analysis section) in a given tissue, were marked as "expressed gene" in that tissue. Most genes expressed in each tissue were protein coding, followed by non-coding, and pseudogenes (Supplemental file 2: Fig. S8). Testis showed the highest number of expressed genes compared to other tissues (Supplemental file 2: Fig. S8). In addition, the proportion and number of transcribed pseudogenes was higher in testis than in other tissues (Supplemental file 2: Fig. S8). Fetal brain and fetal muscle tissues showed the highest number and percentage of non-coding genes compared to that observed in other tissues (Supplemental file 2: Fig. S8). There was no significant correlation between the number of input reads and the number of expressed genes across tissues, but the numbers of genes

from different coding potential classes were significantly correlated across tissues (Supplemental file 2: Fig. S9).

Transcripts corresponding to the predicted genes that had at least one exon overlapping an Ensembl- or NCBI-annotated gene were considered to belong to an annotated gene. This supports an intersection analysis of predicted and previously annotated genes that indicated 22,452 (64%) of our predicted genes correspond to previously annotated genes. Approximately 86% of un-annotated transcripts (96,412) were associated with this set of annotated genes. The remaining 12,430 genes (36% of predicted genes) represent un-annotated genes, i.e., genes not found on Ensembl (release 2021-03) or NCBI (release 106), with which 14% of un-annotated transcripts (15,502 transcripts) were associated. The median number of unique transcripts per annotated gene (tpg) was four, which was higher than that observed in either the Ensembl (1.5 tpg) or NCBI (2.3 tpg) annotated gene sets, while the median number of transcripts per un-annotated gene was one, with an average of 1.31 and standard deviation of 1.36. Most of the transcripts identified were transcribed from annotated genes, including 95% of protein-coding transcripts (76,492), 79% of lncRNA transcripts (37,683), 80% of sncRNA transcripts (281), and more than 95% of NMD transcripts (27,511). Annotated genes were enriched with protein-coding genes (p-value < 2.2e-16). The median transcript abundance from annotated genes in their expressed tissues was significantly higher than that observed for un-annotated genes (p-value < 2.2e-16; Supplemental file 2: Fig. S10A). The median number of tissues in which annotated genes were expressed was also significantly higher than that observed for un-annotated genes (p-value < 2.2e-16; Supplemental file 2: Fig. S10B).

More than a third (37%) of genes with at least one predicted protein-coding transcript displayed either multiple 5' UTRs or multiple 3' UTRs among associated transcript isoforms (Fig. 5). The 496 genes with the highest number of UTRs (the top 5% in this metric) were highly enriched (q-value 1.7E-7) for the "response to protozoan" Biological Process (BP) Gene Ontology (GO) term (Supplemental file 2: Fig. S11 and Supplemental file 7).

A median of 51% of the expressed protein-coding genes in each tissue transcribed both protein-coding and non-coding transcripts and were denoted as bifunctional genes. These genes were mostly previously annotated (95%) and had both coding and non-coding transcripts in a median of 21 tissues, representing 57% of their expressed tissues (Fig. 6A and B). Protein-coding transcripts and NMD transcripts covered more than 90% of the exonic length in bifunctional genes (Fig. 6C). This percentage was significantly lower for other types of non-coding transcripts transcribed from bifunctional genes (Fig. 6C). Although transcript terminal sites (TTS) of transcripts encoded by bifunctional genes were centralized around these genes' 3' ends, transcript start sites (TSS) varied greatly among transcript biotypes (Fig. 6C). The TTSs of NSD transcripts, sncRNAs, and intragenic lncRNAs were shifted from their protein-coding genes' start sites (Fig. 6C). Genes that transcribed both protein-coding and non-coding transcripts in all of their expressed tissues were highly enriched for "mRNA processing" (q-value 6.08E-16) and "RNA splicing" (q-value 1.35E-14) BP GO terms that were mostly (65%) related to different aspects of transcription and translation (Fig. 6D and Supplemental file 8).

A total of 3,744 genes were acting as noncoding in a median of two tissues (equivalent to 15% of their expressed tissues) and were switched to protein-coding in the remaining expressed tissues. Detailed investigation of these bifunctional genes in tissues from both adult and fetal

samples (brain, kidney, muscle, and spleen) revealed the total of 106 non-coding genes (90% annotated) in fetal tissues that were switched to protein-coding genes with only protein-coding transcripts in their matched adult tissues (Supplemental file 2: Fig. S12). Functional enrichment analysis of these genes resulted in the identification of enriched BP GO terms related to "humoral immune response", "sphingolipid biosynthetic process", "negative regulation of wound healing", "cellular senescence", "symporter activity", "regulation of lipid biosynthetic process", and "filopodium assembly" (Supplemental file 2: Fig. S12, Supplemental file 9).

A median of 32% of protein-coding genes in each tissue expressed at least a single potentially aberrant transcript (PAT), i.e., NMDs and NSDs. In this group of genes, the number of PATs was strongly correlated with the total number of transcripts (median correlation of 0.61 across all tissues). The median expression level of these genes in their expressed tissues (11.52 RPKM) was significantly higher (p-value < 2.2e-16) than for protein-coding genes with no PATs (4.48 RPKM). In each tissue, protein-coding genes with PATs showed a significantly higher number of introns (p-value < 2.2e-16; median of 65 introns per gene) than that observed in the remainder of protein-coding genes (median of 15 introns per gene). In addition, genes from this group were expressed in a median of 47 tissues, significantly higher (p-value < 2.2e-16) than that observed for the other group of genes (Supplemental file 2: Fig. S13A and B). These genes transcribed a median of two PATs in half of their expressed tissues, equivalent to a median of 22% of all their transcripts in each tissue. Protein-coding genes that transcribed PATs as their main transcripts (PATs comprised >50% of their transcripts) in all of their expressed tissues were highly enriched with RNA splicing–related BP GO terms (Supplemental file 10).

Gene similarity to other species

Eighty-five percent of protein-coding genes (18,087) encoded either homologous proteins or homologous ncRNAs (Supplemental file 2: Fig. S14A). Nineteen percent of protein-coding genes (4,043) encoded cattle-specific proteins (Supplemental file 2: Fig. S14A). Most of these genes (68%) were either annotated genes or genes with homology to another cattle gene(s) that has established homology to genes in other species (Supplemental file 2: Fig. S14C). The remaining 32% of cattle-specific, protein-coding genes (1,293) were denoted as protein-coding orphan genes (Supplemental file 2: Fig. S14C). A median of 70 protein-coding orphan genes were expressed in each tissue. The expression level of these genes was significantly lower than other types of protein-coding genes (Additional file 2: Fig. S15A and B). The median number of expressed tissues for protein-coding orphan genes was lower than for other types of protein-coding genes (Supplemental file 2: Fig. S15C). In addition, protein-coding orphan genes only transcribed protein-coding transcripts in their expressed tissue(s).

Fifty percent of non-coding genes (5,559) encoded either homologous short peptides (9-43 amino acids) or homologous ncRNAs (Supplemental file 2: Fig. S14B). There were 5,546 non-coding genes (51% of non-coding genes) that encoded cattle-specific ncRNAs (Supplemental file 2: Fig. S14B). Ninety-nine percent of these genes were either annotated genes or genes with homology to another cattle gene(s) that has established homology to genes in other species (Supplemental file 2: Fig. S14C). The remaining 1% (nine non-coding genes) were denoted as non-coding orphan genes (Supplemental file 2: Fig. S14C). The median number of expressed tissues for non-coding orphan genes was was higher (p-value < 2.2e-16) than for homologous non-coding genes and protein-coding orphan genes (Supplemental file 2: Fig. S15C).

A total of 2,990 pseudogenes were expressed. The median expression level of these genes in their expressed tissues was lower than that observed for protein-coding genes and similar to that observed for non-coding genes (Supplemental file 2: Fig. S16A). Pseudogenes were expressed in a median of four tissues (Supplemental file 2: Fig. S16B). In addition, a total of 1,002 pseudogene-derived lncRNAs were expressed. The median expression of pseudogene-derived lncRNAs was similar to that observed for other lncRNAs (Supplemental file 2: Fig. S17A). In addition, pseudogene-derived lncRNAs were expressed in fewer tissues than observed for other lncRNAs (Supplemental file 2: Fig. S17B).

Testis had the highest number of expressed pseudogene-derived lncRNAs compared to other tissues (Supplemental file 2: Fig. S8A and B). The correlation between the number of input reads and the number of pseudogene-derived lncRNAs was not significant (0.25, p-value 0.09).

Gene expression diversity across tissues

Tissue similarities increased dramatically from transcript level to gene level (Supplemental file 2: Fig. S4A, Fig. S5A, Fig. S18A, Fig. S19A). The median percentage of shared genes between pairs of tissues was significantly higher in protein-coding genes compared to non-coding genes (p-value < 2.2e-16; Supplemental file 2: Fig. S18A, Fig. S19A). Clustering of tissues based on protein-coding genes was similar to that observed based on protein-coding transcripts (Supplemental file 2: Fig. S18B, Fig. S19B). The same result was observed in non-coding genes and transcripts. In addition, clustering of tissues based on protein-coding genes was different than that of non-coding genes (Supplemental file 2: Fig. S4B, Fig. S5B, Fig. S18B, Fig. S19B, Fig. S35F).

Tissues with both fetal and adult samples (brain, kidney, muscle, and spleen) were used to investigate gene biotype differences between these developmental stages. Similar to what was observed at transcript level, fetal tissues were significantly enriched for non-coding genes and pseudogenes and were depleted for protein-coding genes (p-value < 2.2e-16; Supplemental file 10). These results were consistent across all tissues with both adult and fetal samples (Supplemental file 11).

Gene validation

A total of 32,460 genes (93% of predicted genes) were structurally validated by independent datasets (PacBio Iso-seq data, ONT-seq data, *de novo* assembled transcripts from RNA-seq data) and comparison with Ensembl and NCBI gene sets (see Method section). In addition, a total of 31,635 genes (90% of predicted genes) were expressed in multiple tissues (31,635 genes or 90%) (Fig. 7). All genes were extensively supported by data from different technologies such as WTTS-seq, RAMPAGE, histone modification (H3K4me3, H3K4me1, H3K27ac) and CTCF-DNA binding, and ATAC-seq data generated from the samples (Fig. 7).

Identification and validation of annotated gene border extensions

This new bovine gene set annotation extended (5' end extension, 3' end extension, or both) more than 11,000 annotated Ensembl or NCBI gene borders. Extensions were longer on the 3' side, but the median increase was 104 nt for the 5' end (Table 5). To validate gene border extensions, independent WTTS-seq and RAMPAGE datasets were utilized. More than 80% of annotated gene border extensions were validated by independent data (Fig. 8). The extension of annotated gene borders on both ends resulted in an approximate nine-fold expression

increase of these genes in the new bovine gene set annotation compared to their matched Ensembl and NCBI genes (Table 6).

Alternative splicing events

A total of 102,502 transcripts (85% of spliced transcripts) were involved in different types of Alternative Splicing (AS) events (see Methods section and Supplemental file 1: Fig. S20A), a large increase over Ensembl (63% of spliced transcripts) and NCBI (75% of spliced transcripts) annotations (Additional file1: FigureS20B). Skipped exons were observed in a greater number of transcripts compared to other types of AS events (Supplemental file 2: Fig. S21).

A median of 60% of tissue transcripts showed at least one type of AS event (Supplemental file 1: Fig. S22A). There was no significant correlation between the number of input reads and the number of AS event transcripts across tissues (Supplemental file 2: Fig. S22B).

The median expression level of AS transcripts (111,366) was similar to that observed for other types of transcripts (Supplemental file 2: Fig. S23A). In addition, AS transcripts were expressed in a higher number of tissues compared to the other transcript types (Supplemental file 2: Fig. S23B). Alternatively spliced transcripts were enriched with protein-coding transcripts ($p\text{-value} < 2.2e\text{-}16$). A switch from protein-coding to ncRNAs was the main biotype change resulting from AS events (Supplemental file 2: Fig. S24).

A median of four AS events were expressed in alternatively spliced genes (14,260 genes) (Supplemental file 2: Fig. S25). The top five percent of genes with the highest number of AS events were highly enriched for several BP GO terms related to different aspects of RNA splicing (Supplemental file 2: Fig. S26B, Supplemental file 12).

Comparison of tissues with both fetal and adult samples (brain, kidney, Longissimus Dorsi (LD) muscle, and spleen) revealed a significantly higher rate of AS events in fetal tissues (only genes expressed in both fetal and adult samples were included in this analysis) (Supplemental file 2: Fig. S27).

Tissue specificity

Nine percent of all genes and transcripts were only expressed in a single tissue and were denoted as tissue-specific (Supplemental file 2: Fig. S28A). Most tissue-specific genes (75%) and transcripts (84%) were un-annotated. Forty-nine percent of tissue-specific transcripts (11,748) were produced by annotated genes. Most tissue-specific genes and transcripts were protein-coding (Supplemental file 2: Fig. S28A and B). In addition, more than 70% of tissue-specific transcripts (11,222) were transcribed from non-tissue-specific genes. Compared to other tissues, testis and thymus had the highest number of tissue-specific genes and transcripts (Supplemental file 2: Fig. S28C, Supplemental file 12). The expression level of tissue-specific genes and transcripts was significantly lower than that of their non-tissue-specific counterparts (p-value < 2.2e-16; Supplemental file 2: Fig. S28D). A median of 71% of tissue-specific transcripts showed any type of AS event in their expressed tissues (Supplemental file 2: Fig. S29). This was only 3.9% for tissue-specific genes (Supplemental file 2: Fig. S29). Testis, myoblasts, mammary gland, and thymus had the highest proportion of tissue-specific genes displaying any type of AS event (Supplemental file 2: Fig. S29).

A total of 6,744 multi-tissue expressed genes (21% of all multi-tissue expressed genes) and 71,662 multi-tissue expressed transcripts (49% of all multi-tissue expressed transcripts) showed

Tissue Specificity Index (TSI) scores greater than 0.9 and were expressed in a tissue-specific manner (Supplemental file 14). These genes and transcripts were expressed in a median of six tissues and four tissues, respectively (Supplemental file 2: Fig. S30A and B). Functional enrichment analysis of the top five percent of genes with the highest TSI score resulted in the identification of “sexual reproduction” (p-value 3.06e-24) and “fertilization” (p-value 1.04e-8) as their top enriched BP GO terms (Supplemental file 2: Fig. S30C-E, Supplemental file 15).

Tying genes to phenotypes

There was a median of 7,263 predicted genes identified as the closest expressed gene to an existing QTL (QTL-associated genes) per tissue (Supplemental file 16). These genes had either QTLs located inside (median of 4,563 genes) or outside (median of 4,678 genes) their genomic borders (either from their 5’ end or 3’ end) with a median distance of 51.9 kilobases (KB) and a maximum distance of 2.6 million bases (MB) (Supplemental file 2: Fig. S31). Most QTL-associated genes were annotated genes (8,130 genes or 83%). In addition, the median number of AS events in these genes (eight) was significantly higher than that observed in other genes (median of seven AS events; p-value 5.69e-09).

Potential testis-pituitary axis

Testis tissue was not clustered with any other tissues and had the highest number of tissue-specific genes compared to the rest of the tissues (Supplemental file 2: Fig. S4, Fig. S5, Fig. S18, and Fig. S19). Testis-specific genes were highly enriched with different traits related to fertility (e.g., percentage of normal sperm and scrotal circumference), body weight (e.g., body weight gain and carcass weight), and feed efficiency (e.g., residual feed intake) (Supplemental file 17).

The extent of testis-pituitary axis involvement in the “percentage of normal sperm” was investigated using animals with both testis and pituitary samples (three samples per tissue). The *SPACA5* gene was the only testis-specific gene encoded protein with a signal peptide (SP) that was close to the “percentage of normal sperm” QTLs. The expression of this gene in testis samples showed significant positive correlation with 70 pituitary expressed genes that were closest to the “percentage of normal sperm” QTLs (Supplemental file 2: Fig. S32, Supplemental file 18). These pituitary genes were enriched with the “signal transduction in response to DNA damage” BP GO term (Supplemental file 2: Fig. S32). In addition, the expression of testis genes that encoded protein with a signal peptide that were close to the “percentage of normal sperm” QTLs was significantly correlated with expression of pituitary genes close to this trait (Fig. 9, Supplemental file 19). The same result was observed for the pituitary-testis tissue axis (Supplemental file 2: Fig. S33, Supplemental file 20).

Trait similarity network

The extent of genetic similarity between different bovine traits was investigated using their associated QTLs. A total of 1,857 significantly similar trait pairs (184 different traits) were identified and used to create a bovine trait similarity network (Supplemental file 21).

miRNAs

A total of 2,007 miRNAs (at least ten mapped reads in each tissue) comprised of 973 annotated and 1,034 un-annotated miRNAs were expressed (Supplemental file 22). In each tissue, a median of 704 annotated miRNAs and 549 un-annotated miRNAs were expressed (Fig. 10A). The median expression of un-annotated miRNAs was significantly lower than that observed for

annotated miRNAs (p-value 3.25e-25; Fig. 10B). In addition, un-annotated miRNAs were expressed in significantly lower number of tissues than for annotated miRNAs (p-value 1.00e-45; Fig. 10C). A median of 84.53% of miRNAs were shared between pairs of tissues (Supplemental file 2: Fig. S34). Clustering of tissues based on miRNAs was similar to what was observed based on non-coding genes (Supplemental file 2: Fig. S35).

A total of 113 miRNAs (5.6%) were expressed in a single tissue and were denoted as tissue-specific (Supplemental file 2: Fig. S36A). The proportion of tissue-specific miRNAs was higher for un-annotated miRNAs, such that 75% of the tissue-specific miRNAs were un-annotated. The number of un-annotated miRNAs was higher in pre-adipocytes compared to other tissues, followed by fetal gonad and testis (Supplemental file 2: Fig. S36B). Un-annotated miRNAs showed a significantly lower expression level compared to annotated miRNAs (p-value 1.4e-19; Supplemental file 2: FigureS36 C). In addition, a total of 1,047 multi-tissue expressed miRNAs were expressed in a tissue-specific manner (Supplemental file 2: Fig. S36D). These miRNAs were expressed in a median of 19 tissues (Supplemental file 2: Fig. S36E).

Chromatin features across 500-base pair (bp) windows surrounding upstream of miRNA precursors' start sites or downstream of miRNA precursors' terminal sites from independent cattle experiments were used to investigate the relationship between miRNAs and chromatin accessibility. More than 99% of un-annotated miRNAs and 94% of annotated miRNAs were supported by at least one of the H3K4me3, H3K4me1, H3K27ac, CTCF-DNA binding, or ATAC-seq peaks (Fig. 11).

Summary of expressed transcripts, genes, and miRNAs

The numbers of expressed transcripts, genes, and miRNAs in different tissues are summarized in Supplemental file 2: Fig. S37. In addition, the number of annotated and un-annotated genes, transcripts, and miRNAs in different tissues are summarized in Supplemental file 2: Fig. S38.

Discussion

Despite many improvements in the current bovine genome annotation ARS-UCD1.2 assembly (Ensembl release 2021-03 and NCBI release 106) compared to the previous genome assembly (UMD3.1), these annotations are still far from complete [12, 13]. In this study, using RNA-seq and miRNA-seq data from 50 different bovine tissues, developmental stages, and cell types, 12,444 un-annotated genes and 1,034 un-annotated miRNAs were identified that have not been reported in current bovine genome annotations (Ensembl release 2021-03, NCBI release 106 and miRbase [14]). In addition, we identified protein-coding transcripts with a median ORF length of 270 nt for 822 annotated bovine genes that have been annotated as non-coding in current bovine genome annotations (Supplemental file 2: Fig. S14C). The high frequency of validation of these un-annotated genes and un-annotated miRNAs using multiple independent datasets from different technologies verifies the improvement in terms of the number of genes and miRNAs using our methods.

Five prime and 3‘untranslated region length plays a critical role in regulation of mRNA stability, translation, and localization [7]. However, only a single 5’ UTR and 3’ UTR per gene is annotated in current bovine genome annotations (Ensembl release 2021-03 and NCBI release 106), and variations in UTR length are not available. In this study, 7,909 genes (22% of predicted genes)

with multiple UTRs were identified. Genes with multiple 5′ UTRs are common, primarily due to the presence of multiple promoters [15] or alternative splicing mechanisms within 5′ UTRs [15]. Fifty-four percent of human genes have multiple transcription start sites [15]. In addition, the length of 3′ UTRs often varies within a given gene, due to the use of different poly(A) sites [7, 16].

In this study, around 50% of expressed protein-coding genes in each tissue transcribed both coding and non-coding transcript isoforms. Several studies have shown evidence of the existence of bifunctional genes with coding and non-coding potential using RNA-seq and ribosome footprinting followed by sequencing (Ribo-seq) [17-19]. For example, steroid receptor RNA activator (SRA), a known bifunctional gene, acting as a lncRNA while also encoding a conserved protein SRAP, both of which contribute to the development and progression of prostate and breast cancers [20]. More than 20% of human protein-coding genes have been reported to transcribe non-coding isoforms, often generated by alternative splicing [21] and recurrently expressed across tissues and cell lines [19]. A considerable number of non-coding isoform variants of protein-coding genes appear to be sufficiently stable to have functional roles in cells [22]. It has been shown that the proportion of non-coding isoforms from protein-coding genes dramatically increases during myogenic differentiation of primary human satellite cells and decreases in myotonic dystrophy muscles [23]. In this study, 106 non-coding genes were identified in fetal tissues that switched to protein-coding genes in their matched adult tissues. Taken together this supports the notion that protein-coding/non-coding transcript switching plays an important role in tissue development in cattle as well.

Nonsense-mediated RNA decay is an evolutionarily conserved process involved in RNA quality control and gene regulatory mechanisms [24]. For instance, the RNA-binding protein polypyrimidine tract binding protein 1 (*PTBP1*) can promote the transcription of NMD transcripts via alternative splicing, which negatively regulates its own expression [25]. In this study, NMD transcripts comprised 18% of bovine transcripts that were transcribed from 30% of bovine genes (10,380). In humans, NMD-mediated degradation can affect up to 25% of transcripts [26] and 53% of genes [27]. As expected, in this study, most genes that transcribed NMD transcripts were protein coding (83% or 8,610 genes), while a considerable portion (17%) were pseudogenes. Many pseudogenes are annotated to give rise to NMD transcripts [28, 29]. Bioinformatic study of the human transcriptome revealed that 78% of NMD transcript–producing genes were protein coding, followed by pseudogenes (nine percent), long intergenic noncoding RNAs (six percent), and antisense transcripts (four percent) [29].

Despite the important regulatory function of lncRNAs and miRNAs, very low numbers of these elements have been annotated in the current bovine genome annotations (Table 7). In this study, a total of 10,689 lncRNA genes and 2,007 miRNA genes were expressed in the bovine transcriptome, which is similar to what has been reported for the human transcriptome (Table 7). While, a total of 3,770 human miRNAs and 1,203 cattle miRNAs have been reported in miRbase [14].

In this study, 1,002 pseudogene-derived lncRNAs were identified that were recurrently expressed across tissues and cell types. Ever-increasing evidence from different studies suggests pseudogene derived RNAs are key components of lncRNAs [30-32]. lncRNAs expressed

from pseudogenes have been shown to regulate genes with which they have sequence homology [30, 31] or to coordinate development and disease in metazoan systems [30].

Correct annotation of gene borders has an important role in defining promoter and regulatory regions. Our novel transcriptome analysis extended (5′-end extension, 3′-end extension, or both) more than 11,000 annotated Ensembl or NCBI gene borders. Extensions were longer on the 3′ side, which was relatively similar to that we observed in the pig transcriptome using PacBio Iso-Seq data [2].

A growing body of evidence indicates that a considerably large portion of lncRNAs encode microproteins that are less conserved than canonical open reading frames [33-37]. In this study, a vast majority (98%) of predicted lncRNAs had short ORFs (<44 amino acids) that were less conserved than canonical ORFs (Table 2).

Alternative splicing is the key mechanism to increase the diversity of the mRNA expressed from the genome and is therefore essential for response to diverse environments. In this study, skipped exons and retained introns were the most prevalent AS events identified in the bovine transcriptome, similar to what has been observed in other vertebrates and invertebrates [38]. A higher rate of AS events was observed in fetal tissues compared to their adult tissue counterparts. The same result has been observed in a recently published study in humans [39].

We hypothesized that the integration of the gene/transcript data with previously published QTL/gene association data would allow for the identification of potential molecular mechanisms responsible for a) tissue-tissue communication as well as b) genetic correlations between traits. To test the first hypothesis, we developed a novel approach to study the

involvement of tissue-tissue interconnection in different traits based on the integration of the transcriptome with publicly available QTL data. In particular, the interconnection between testis and pituitary tissues with respect to the “percentage of normal sperm” trait was investigated in more detail. This resulted in the identification of the regulation of ubiquitin-dependent protein catabolic process, the regulation of nuclear factor-κB (NF-κB) transcription factor activity, and Rab protein signal transduction as key components of this tissue-tissue interaction (Supplemental file 19 and 20). Interestingly, expressed genes that were closest to “percentage of normal sperm” QTLs, and also encoded protein with a signal peptide (short peptide present at the N-terminus of proteins that are destined toward the secretory pathway[40]) in both testis and pituitary tissues, were highly enriched for the BP GO term “regulation of ubiquitin-dependent protein catabolic process” (Supplemental file 18 and 19). The expression of these genes in testis tissue was significantly correlated with expression levels of pituitary expressed genes closest to “percentage of normal sperm” QTLs that were highly enriched for the “positive regulation of NF-kappaB transcription factor activity” BP GO term (Supplemental file 2: Fig. S32 and Supplemental file 19). Activation of NF-κB requires ubiquitination, and this modification is highly conserved across different species [41]. NF-κB induces secretion of adrenocorticotropic hormone from the pituitary [42], which directly stimulates testosterone production by the testis [43]. In addition, ubiquitinated proteins in testis cells are required for the progression of mature spermatozoa [44]. The expression levels of pituitary expressed genes closest to “percentage of normal sperm” QTLs that also encoded signal peptides were significantly correlated with expression levels of testis expressed genes closest to “percentage of normal sperm” QTLs (Supplemental file 2: Fig. S33). These testis genes

were highly enriched for the “Rab protein signal transduction” BP GO term (Supplemental file 20). Rab proteins have been reported to be involved in male germ cell development [45]. Thus, it appears that integration of gene data with QTL/association data can be used to identify putative molecular pathways underlying tissue-tissue communication mechanisms.

To test the second hypothesis, we also developed a novel approach to study trait similarities based on the integration of the transcriptome with publicly available QTL data. Using this approach, we could identify significant similarity between 184 different bovine traits. For example, clinical mastitis showed significant similarity with 23 different cattle traits that were greatly supported by published studies, such as milk yield [46], milk composition traits [47], somatic cell score [48], foot traits [49], udder traits [50], daughter pregnancy rate [51], length of productive life [52] and net merit [53]. Similar results were observed for residual feed intake, which showed significant similarity with 14 different traits such as average daily feed intake [54], average daily gain [55], carcass weight [56], feed conversion ratio [57], metabolic body weight [58], subcutaneous fat [59], and dry matter intake [60].

Taken together, these results identify a list of candidate genes that might be controlled by genetic variation responsible for the genetic mechanisms underlying genetic correlations (Supplemental file 19 and 20). If this is the case, in the future, these novel methods should be able to predict the impact of a given set of genetic variants that are associated with a trait of interest on other traits that were not measured in a given study. This might then lead to the optimization of variants used (or not used) in genomic selection to minimize any non-beneficial effect of selection on selected traits. However, it is important to acknowledge that (1) the nearest neighbor gene to a genotype association may not necessarily be the causal gene, (2)

the breed/gender differences between this study and the data from Animal QTLdb may impact the results, and (3) due to experimental limitations, the genetic and phenotypic association data were not used in this study. None the less, these results are intriguing in that meaningful genetic correlation can be recapitulated. Furthermore, these results indicate the potential for gene mechanisms whereby traits that have genetic correlations to be identified.

Conclusions

In-depth analysis of multi-omics data from 50 different bovine tissues, developmental stages, and cell types provided evidence to improve the annotation of thousands of protein-coding, lncRNA, and miRNA genes. These validated results increase the complexity of the bovine transcriptome (number of transcripts per gene, number of UTRs per gene, lncRNA transcripts, AS events, and miRNAs), comparable to that reported for the highly annotated human genome. The predicted un-annotated transcripts extend existing annotated gene models, by verifying such extensions using independent WTTS-seq and RAMPAGE data. The integrated transcriptome data with publicly available QTL data revealed putative molecular pathways that may underlie tissue-tissue communication mechanisms and candidate genes responsible for the genetic mechanisms that may underlie genetic correlations between traits. This integrative approach is particularly important in the selection of indicator traits for breeding purposes, study of artificial selection side effects in livestock species, and functional annotation of poorly annotated livestock genomes.

Methods

Tissue sample collection and sequencing library preparation methods are summarized in Supplemental file 23. The overview of the bioinformatics analysis steps is presented in Supplemental file 2: Fig. S39.

RNA-seq data analysis and transcriptome assembly

Single-end Illumina RNA-Seq reads (75 bp) from each tissue sample were trimmed to remove the adaptor sequences and low-quality bases using Trim Galore (RRID:SCR_011847) (version 0.6.4) [61] with --quality 20 and --length 20 option settings. The resulting reads were aligned against ARS-UCD1.2 bovine genome using STAR (RRID:SCR_004463) (version 020201) [62] with a cut-off of 95% identity and 90% coverage. FeatureCounts (RRID:SCR_012919) (version 2.0.2) [63] was used to quantify genes reported in the NCBI gene build (version 1.21) with -Q 255 -s 2 --ignoreDup --minOverlap 5 option settings. The resulting gene counts were adjusted for library size and converted to Counts Per Million (CPM) values using SVA R package (version 3.30.0) [64]. In each tissue, sample similarities were checked using hierarchical clustering and regression analysis of gene expression values (log2 based CPM), and outlier samples were expressed and removed from downstream analysis. Samples from each tissue were combined to get the most comprehensive set of data in each tissue. To reduce the processing time due to huge sequencing depth, the trimmed reads were in silico normalized using insilico_read_normalization.pl from Trinity package (RRID:SCR_013048) (version 2.6.6) [65] with --JM 350G and --max_cov 50 option settings. Normalized RNA-seq reads were aligned against ARS-UCD1.2 bovine genome using STAR (version 020201) [62] with a cut-off of 95% identity and 90% coverage. The normalized reads were assembled using *de novo* Trinity software (version 2.6.6) [65] combined with massively parallelized computing using HPCgridRunner (v1.0.1) [66]

and GNU parallel software [67]. The resulted transcript reads were mapped against ARS-UCD1.2 bovine genome using GMAP (RRID:SCR_008992) [68] with a cut-off of 95% identity and 90% coverage. In the next step, transcript reads were collapsed and grouped into putative gene models (clustering transcripts that had at least a one-nucleotide overlap) by the pbtranscript-ToFU from SMRT Analysis software (v2.3.0) [69] with min-identity = 95%, min-coverage = 90% and max_fuzzy_junction = 15 nt, whereas the 5'-end and 3'-end difference were not considered when collapsing the reads. Base coverage of the resulting transcripts was calculated using mosdepth (RRID:SCR_018929) (version 0.2.5) [70]. Predicted transcripts were required to have a minimum of three times base coverage in their assembled tissues. The predicted acceptor and donor splice sites were required to be canonical and supported by Illumina-seq reads that spanned the splice junction with 5-nt overhang. Spliced transcripts with the exact same splice junctions as their reference transcripts but that contained retained introns were removed from analysis, as they were likely pre-RNA sequences. Unspliced transcripts with a stretch of at least 20 A's (allowing one mismatch) in a genomic window covering 30 bp downstream of their putative terminal site were removed from analysis, as they were likely genomic-DNA contaminations. To decrease the false positive rate, unspliced transcripts that were only expressed in a single tissue were removed from downstream analysis. In addition, single-exon genes without histone mark (H3K4me3, H3K4me1, H3K27ac) or ATAC-seq peaks mapped to their promoter (see Relating transcripts and genes to epigenetic data section) were removed from downstream analysis as they were likely transcriptional noise. The resulting transcripts from each tissue were re-grouped into gene models using an in-house Python script.

Structurally similar transcripts from the different tissues (see Comparison of transcript

structures across datasets/tissues section) were collapsed using an in-house Python script to create the RNA-seq based bovine transcriptome.

The resulting transcripts and genes were quantified using align_and_estimate_abundance.pl from the Trinity package (version 2.6.6) [65] with --aln_method bowtie --est_method RSEM --SS_lib_type R option settings. The quantified counts were normalized for sequencing depth using RPKM method.

“Isoform” and “transcript” terms are used interchangeably throughout the manuscript.

PacBio Iso-Seq data analysis

Publicly available PacBio Iso-seq reads and matched RNA-seq reads (PRJNA386670) were used in this study. In brief, a total of six tissue from L1 Dominette 01449 (aged 11 years old), and testis from SuperBull 99375 (aged 9 years old) were used in this experiment (Supplemental file 24). RNA was extracted using TRIzol reagent as directed by the manufacturer (Invitrogen) with integrity examined using a BioAnalyzer (Agilent). Libraries for RNA-seq short-read sequencing were prepared using the TruSeq RNA Kit following the “TruSeq RNA Sample Preparation v2 Guide” as recommended by the manufacturer (Illumina). RNA-seq libraries were sequenced on a NextSeq500 instrument. IsoSeq libraries for long-read sequencing were prepared using the SMRTbell Template Prep Kit 1.0. cDNA was converted to SMRTbell template library following the “Iso-Seq using Clontech cDNA Synthesis and BluePippin Size Selection” protocol as directed by the manufacturer (Pacific Biosciences). The sequences were processed into HQ isoforms using SMRT Analysis v6.0 for each tissue independently but with all size fractions within tissue included in the analysis.

PacBio Iso-seq data has been processed as described for the pig transcriptome [2] with the following exceptions. Errors in the full-length, non-chimeric (FLNC) cDNA reads were corrected with the preprocessed RNA-Seq reads from the same tissue samples using the combination of proovread (RRID:SCR_017331) (v2.12) [71] and FMLRC (v1.0.0) [72] software packages. Error rates were computed as the sum of the numbers of bases of insertions, deletions, and substitutions in the aligned FLCN error-corrected reads divided by the length of aligned regions for each read (Table 8).

The RNA-seq-based transcriptome was assembled as described in the previous section.

Oxford Nanopore data analysis

Assembled isoforms from a previously published Oxford Nanopore experiment were used in this study [12]. In brief, a total of 32 tissues (Supplemental file 24) from two male and two female Line 1 Hereford cattle, aged 14 months old were used in this experiment. Barcoded cDNAs extracted from frozen tissues (-80 °C) were pooled at the University of California Davis and sequenced using Oxford Nanopore Technologies SQK-DCS109 kit according to the manufacturer's protocol [12].

Comparison of transcript structures across datasets/tissues

The structure of transcripts predicted from RNA-seq data were compared across tissues, and independent datasets including a library of annotated isoforms (Ensembl release 2021-03, and NCBI Release 106), as well as isoforms identified through complete isoform sequencing with Pacific Biosciences, a de novo assembly produced from its matched RNA-seq reads, and isoforms identified from Oxford Nanopore platforms. Transcripts whose 5' and 3' borders were

supported by RAMPAGE and/or WTTS data (see Transcript and gene border validation section) and whose splice junctions were identical (maximum fuzzy junction was set to 15 bp) were considered "structurally equivalent transcripts". The maximum of 100 nt fuzzy 5' and 3' transcript borders were applied when comparing transcripts were not supported by RAMPAGE and/or WTTS data. Other transcripts that did not met these criteria were considered "structurally different transcripts".

A pair of genes was considered as structurally equivalent across datasets if they transcribed at least single "structurally equivalent transcript".

Prediction of transcript and gene biotypes

Transcripts' open reading frames (ORFs) were predicted using the stand-alone version of ORFfinder [73] with "ATG and alternative initiation codons" as ORF start codon. The longest three ORFs were matched to the Uniprot (RRID:SCR_002380) vertebrate database using Blastp (RRID:SCR_001010) [73] with E-value cutoff of 10^{-6}, min coverage 60%, and min identity 95%. The ORFs with the lowest E-value to a protein were used as the representative, or if no matches were found, the longest ORF was used. Putative transcripts that had representative ORFs longer than 44 amino acids were labelled as protein-coding transcripts. If the representative ORF had a stop codon that was more than 50 bp upstream of the final splice junction, it was labelled as a nonsense-mediated decay transcript [74]. Transcripts with start codon but no stop codon before their poly(A) site were labelled non-stop decay RNAs. Putative non-coding transcripts (ORFs shorter than 44 amino acids and lack of coding potential predicted by CPC2 [75]) with lengths less than 200 bp that did not overlap with annotated or un-annotated miRNA

precursors (see miRNA-seq data analysis section) were labelled as small non-coding RNAs [74]. Putative non-coding transcripts with lengths greater than 200 bp were labelled as long non-coding RNAs [74]. Long non-coding RNAs overlapping one or more coding loci on the opposite strand were labelled as antisense lncRNAs. Long non-coding RNAs located in introns of coding genes on the same strand were labelled as sense-intronic lncRNAs. Long non-coding RNAs that had an exon(s) that overlapped with a protein-coding gene were labeled as Intragenic lncRNAs. Long non-coding RNAs located in intergenic regions of the genome were labeled as Intergenic lncRNAs.

Putative genes that transcribed at least a single protein-coding transcript were labelled as protein-coding genes. Putative genes with homology to existing vertebrate protein-coding genes (Blastx [73], E-value cut-off 10^{-6}, min coverage 90%, and min identity 95%) but containing a disrupted coding sequence, i.e., transcribe only nonsense-mediated decay or non-stop decay transcripts in all of their expressed tissues, were labelled as pseudogenes. The rest of the putative genes were labeled as non-coding.

ncRNAs homology analysis

Putative non-coding transcripts were matched to NCBI and Ensembl vertebrate ncRNA databases using Blastn (RRID:SCR_001598) [73] with E-value cutoff of 10^{-6}, min coverage 90%, and min identity 95%. Transcripts with at least one hit were considered as homologous ncRNAs.

Transcriptome termini site sequencing data analysis

T-rich stretches located at the 5′ end of each WTTS-seq raw read were removed using an in-house Perl script, as described previously [76]. T-trimmed reads were error-corrected using

Coral (version 1.4.1) [77] with -v -Y -u -a 3 option settings. The resulting reads with length greater than 300 nt were quality trimmed using FASTX Toolkit (RRID:SCR_005534) (version 0.0.14) [78] with -q 20 and -p 50 option settings. High-quality, error-corrected WTTS-seq reads were aligned against the ARS-UCD1.2 bovine genome using STAR (version 020201) [62] with a cut-of of 95% identity and 90% coverage.

Chromatin immunoprecipitation sequencing (ChIP-seq) data analysis

Regions of signal enrichment ("peaks") from a previously published ChIP-seq experiment were used in this study [79]. In brief, total eight tissue (Supplemental file 24) from two male Line 1 Hereford cattle, aged 14 months old were used in this experiment. ChIP-seq experiments were performed on frozen tissue (-80 °C) using the iDeal ChIP-seq kit for Histones (Diagenode Cat.#C01010059, Denville, NJ) based on protocol described at [79]. The following antibodies used were from Diagenode: H3K4me3 (in kit), H3K27me3 (#C15410069), H3K27ac (#C15410174), H3K4me1 (#C15410037), and CTCF (#15410210).

ATAC-seq data analysis

The UC Davis FAANG Functional Annotation Pipeline was applied to process the ATAC-seq data, as previously described [79]. Briefly, the ARS-UCD1.2 genome assembly and Ensembl genome annotation (v100) were used as references for cattle. Sequencing reads were trimmed with Trim Galore! (Krueger et al. 2015) (v.0.6.5) and aligned BWA (Li et al. 2013) (v0.7.17) to the ARS-UCD1.2 genome assembly with --fr option. Alignments with MAPQ scores <30 were filtered using Samtools (RRID:SCR_005227) (v.1.9). Duplicate reads were marked and removed using Picard (RRID:SCR_006525) (v.2.18.7). Regions of signal enrichment were called by MACS2

(RRID:SCR_013291) (v.2.1.1).

Relating transcripts and genes to epigenetic data

The promoter was defined as the genomic region that spans from 500 bp 5′ to 100 bp 3′ of the gene/transcript start site. Histone mark (H3K4me3, H3K4me1, H3K27ac), CTCF-DNA binding or ATAC-seq peaks mapped to the promoter of a given gene/transcript were related to that gene/transcript.

Transcript and gene border validation

RAMPAGE peaks from a previously published experiment [13] were used to validate gene/transcript start site (Supplemental file 24). Peaks within the genomic region that spans from 30 bp 5′ to 10 bp 3′ of a gene/transcript start site were assigned to that gene/transcript. WTTS-seq reads (median length of 161 bp) within the genomic region that spans from 10 bp 5′ to 165 bp 3′ of a gene/transcript terminal site were assigned to that gene/transcript.

Functional enrichment analysis

The potential mechanism of action of a group of genes was deciphered using ClueGO (RRID:SCR_005748) [80]. The latest update (May 2021) of the Gene Ontology Annotation database (GOA) [81] was used in the analysis. The list of genes with at least one transcript expressed in a given tissue was used as background for that tissue. The GO tree interval ranged from 3 to 20, with the minimum number of genes per cluster set to three. Term enrichment was tested with a right-sided hyper-geometric test that was corrected for multiple testing using the Benjamini-Hochberg procedure [82]. The adjusted p-value threshold of 0.05 was used to filter enriched GO terms. Enriched GO terms were grouped based on kappa statistics [83].

Alternative splicing analysis

Alternative splicing (AS) events (Supplemental file 2: Fig. S20A) are commonly distinguished in terms of whether RNA transcripts differ by inclusion or exclusion of an exon, in which case the exon involved is referred to as a “skipped exon” (SE) or “cassette exon”, “alternative first exon”, or “alternative last exon”. Alternatively, spliced transcripts may also differ in the usage of a 5' splice site or 3' splice site, giving rise to alternative 5' splice site exons (A5Es) or alternative 3' splice site exons (A3Es), respectively. A sixth type of alternative splicing is referred to as “mutually exclusive exons” (MXEs), in which one of two exons is retained in RNA but not both. However, these types are not necessarily mutually exclusive; for example, an exon can have both an alternative 5' splice site and an alternative 3' splice site, or have an alternative 5' splice site or 3' splice site, but be skipped in other transcripts. A seventh type of alternative splicing is “intron retention”, in which two transcripts differ by the presence of an unspliced intron in one transcript that is absent in the other. An eighth type of alternative splicing is “unique splice site exons” (USEs), in which two exons overlap with no shared splice junction. Alternative splicing events, except Unique Splice Site Exons, were detected using generateEvents from SUPPA (version 2.3) [84] with default settings. Unique Splice Site Exons were detected using an in-house Python script.

miRNA-seq data analysis

Single-end Qiagen miRNA-seq reads (50 bp) from each tissue sample were trimmed to remove the adaptor sequences and low-quality bases using Trim Galore (version 0.6.4) [61] with --quality 20, --length 16, --max_length 30 -a AACTGTAGGCACCATCAAT option settings. miRNA

reads were aligned against the ARS-UCD1.2 bovine genome using mapper.pl from mirDeep2 (RRID:SCR_010829) (version 0.1.3) [85] with -e -h -q -j -l 16 -o 40 -r 1 -m -v -n option settings. miRNA mature sequences along with their hairpin sequences for Bos taurus species were downloaded from miRbase [14]. These sequences, along with the aligned miRNA reads, were used to quantify annotated miRNAs in each sample using miRDeep2.pl from mirDeep2 (version 0.1.3) [85] with -t bta -c -v 2 setting options. miRNA normalized Reads Per Million (RPM) were used to check sample similarities using hierarchical clustering and regression analysis of gene expression values (log2 based CPM). Outlier samples, which did not cluster together indicating the potential for tissue miss-labelling, were detected, and removed from downstream analysis. In order to predict the most comprehensive set of un-annotated miRNAs, samples from different tissues were concatenated into a single file that were aligned against the ARS-UCD1.2 bovine genome using mapper.pl from mirDeep2 (version 0.1.3) [85] with the aforementioned settings. Aligned reads from the previous step were used, along with annotated miRNAs' mature sequences and their hairpins, to predict un-annotated miRNAs using miRDeep2.pl from mirDeep2 (version 0.1.3) [85] with the aforementioned settings. Samples from each tissue were combined to get the most comprehensive set of data for that tissue. Mature miRNA sequences and their hairpins for both annotated and predicted un-annotated miRNAs' sequences along with the aligned miRNA reads from each tissue were used to quantify annotated and un-annotated miRNAs in each tissue using mirDeep2 (version 0.1.3) [85] with the aforementioned settings.

Tissue-specificity index

Tissue Specificity Index (TSI) calculations were utilized to present more comprehensive information on transcript/gene/miRNA expression patterns across tissues. This index has a range of zero to one with a score of zero corresponding to ubiquitously expressed transcripts/genes/miRNAs (i.e., "housekeepers") and a score of one for transcripts/genes/miRNAs that are expressed in a single tissue (i.e., "tissue-specific") [86]. The TSI for a transcript/gene/miRNA j was calculated as [86]:

$$TSI_j = \frac{\sum_{i=1}^{N}\left(1 - x_{j,i}\right)}{N - 1}$$

where N corresponds to the total number of tissues measured, and $x_{j,i}$ is the expression intensity of tissue i normalized by the maximal expression of any tissue for transcript/gene/miRNA j.

QTL enrichment analysis

Publicly available bovine QTLs were retrieved from Animal QTLdb (RRID:SCR_001748) [87]. Closest expressed gene to a given trait's QTLs were denoted as QTL-associated genes for that trait. The median distance of QTLs located outside gene borders to the closest expressed gene was 51.9 kilobases and the maximum distance was 2.6 million bases. QTL enrichment was tested with a right-sided Fisher Exact test using an in-house Python script. The resulting p-

values were corrected for multiple testing by the Benjamini-Hochberg procedure [82]. The adjusted p-value threshold of 0.05 was used to filter QTLs.

Trait similarity network

For a given pair of traits, trait A was denoted as “similar” to trait B if a significant portion of trait A’s QTL-associated genes were also the closest expressed genes to trait B QTLs based on 1000 permutation tests. The resulting p-values were corrected for multiple testing using the Benjamini-Hochberg procedure [82]. The same procedure was used to test trait B’s similarity to trait A. The adjusted p-value threshold of 0.05 was used to filter significant trait similarities. A graphical presentation of the method used to construct the tissue similarity network is presented in Supplemental file 2: Fig. S40. The resulting network was visualized using Cystoscape software [88].

Testis-pituitary axis correlation significance test

The presence of signal peptides on representative ORFs of protein-coding transcripts was predicted using SignalP-5.0 [89]. Spearman correlation coefficients were used to study expression similarity between testis genes encoding signal peptides that were closest to the “percentage of normal sperm” QTLs (62 genes) and pituitary expressed genes closest to the “percentage of normal sperm” QTLs (246 genes). To test the statistical difference between these correlation coefficients (reference correlations) and random chance, 1000 random sets of 246 pituitary genes were selected, and their correlation coefficients with 62 previously described testis genes were calculated (random correlations). The reference correlations were

compared with 1000 sets of random correlations using a right-sided t-test. The resulting p-values were corrected for multiple testing by the Benjamini-Hochberg procedure [82]. The distribution-adjusted p-values were used to determine the significance level of expression similarities for genes involved in the testis-pituitary axis related to “percentage of normal sperm”. The same analysis was conducted to determine the significance of pituitary-testis axis involvement in this trait.

Tissue dendrogram comparison across different transcript and gene biotypes

Tissues were clustered based on the percentage of their transcripts/genes that were shared between tissue pairs using the hclust function in R. Cophenetic distances for tissue dendrograms were calculated using the cophenetic R function. The degree of similarity between dendrograms constructed based on different gene/transcript biotypes was obtained using the Spearman correlation coefficient between the dendrograms’ Cophenetic distances.

Figure legends

Figure 1. Distribution of the number of expressed transcripts (A) and genes (B) across tissues.

Figure 2. Classification of the predicted transcripts into different biotypes.

Figure 3. Support of predicted transcripts using data from different technologies and datasets.

Figure 4. Classification of the predicted genes into different biotypes.

Figure 5. Distribution of the number of 5’ UTRs and 3’ UTRs per gene in genes with multiple UTRs.

Figure 6. (A) Classification of protein-coding genes based on their novelty and types of encoded transcripts. (B) Number of expressed tissues for bifunctional genes. Dots have been color coded based on their density. (C) Location of different transcript biotypes on bifunctional genes. (D) Functional enrichment analysis of genes that remained bifunctional in all of their expressed tissues.

Figure 7. Support of predicted genes using data from different technologies and datasets

Figure 8. Functional enrichment analysis of non-coding genes in fetal tissues that were switched to protein coding with only coding transcripts in their matched adult tissue.

Figure 9- (A) Correlation between testis genes encoded protein with a signal peptide that were close to the "percentage of normal sperm" QTL and pituitary expressed genes closest to this trait (reference correlations). (B) Distribution of p-values resulting from a right-sided t-test between reference correlation coefficients and correlation coefficients derived from random chance (see methods for details).

Figure 10- (A) Distribution of the number of expressed annotated and un-annotated miRNAs across tissues. (B) Expression of annotated and un-annotated miRNAs across their expressed tissues. (C) Number of expressed tissues for annotated and un-annotated miRNAs.

Figure 11- Support of annotated (A) and un-annotated (B) miRNAs using different histone marks and CTCF-DNA binding data.

Tables

Table 1. Summary of expressed transcripts/genes

Feature	Annotation[1]		
	Current project	Ensembl (Release 2021-03)	NCBI (Release 106)
Number of genes	34,882 (21,116)	27,607 (21,880)	35,143 (21,355)
Number of transcripts	160,820 (79,957)	43,984 (37,538)	83,195 (47,280)
Number of spliced transcripts	130,531	37,299	73,423
Number of transcripts per gene	4.9	1.5	2.3
Median number of 5' UTRs per gene	2	1	1
Median number of 3' UTRs per gene	1	1	1

[1]Numbers in parentheses indicate the number of protein-coding genes/transcripts.

Table 2. Protein/peptide homology of transcripts with coding potential

Transcript biotype	Number of transcripts	Transcripts with protein/peptide homology to other species[1]
Protein-coding transcripts	85,658	73,268 (86%)
sncRNAs and lncRNAs that encode short peptides[2]	48,425	4,054 (8%)

[1]Number in parentheses indicates the percentage of each transcript biotype.

[2]Open reading frame of 9 to 43 amino acids

Table 3. Sequence homology of non-coding transcripts

Transcript biotype	Number of transcripts	Transcripts with sequence homology to ncRNAs in other species[1]
Long non-coding RNAs	48,661	23,707 (49%)
Small non-coding RNAs	526	194 (37%)
Non-stop decay RNAs	4,359	1,551 (35%)
Nonsense-mediated decay RNAs	32,781	18,195 (55%)

[1]Number in parentheses indicates the percentage of each transcript biotype.

Table 4. Sequence homology of different types of lncRNAs

lncRNA biotype	Number of transcripts	Transcripts with sequence homology to ncRNAs in other species[1]
antisense lncRNAs	29,987	13,793 (46%)
sense-intronic lncRNAs	1,694	1,029 (60%)
intragenic lncRNAs	5,569	2,314 (41%)
intergenic lncRNAs	11,841	5,820 (49%)

[1]Number in parentheses indicates the percentage of each transcript biotype.

Table 5. Gene border extensions in current ARS-UCD1.2 genome annotations by *de novo* assembled transcriptome from short-read RNA-seq data

Annotation	Type of gene extension	Number of genes	Median extension (nucleotides)
Ensembl	5’ extension only	1,848	128
(Release 2021-03)	3’ extension only	5,701	422
	Both ends extended	4,874	122, 5’<br>439, 3’
NCBI	5’ extension only	2,214	80
(Release 106)	3’ extension only	5,496	126
	Both ends extended	3,613	66, 5’<br>210, 3’

Table 6. Median number of reads mapped to the extended region of annotated genes[1]

Annotation	5' end extension	3' end extension	Both ends extension
Ensembl (release 2021-03)	92 (1.10)	220 (1.24)	1,766 (8.90)
NCBI (release 106)	72 (1.05)	95 (1.10)	2,009 (9.05)

[1]Numbers in parentheses indicate the median fold change in expression level resulting from gene extensions.

Table 7. Comparison of different gene builds based on gene biotypes

Species	Gene build	Protein-coding genes	lncRNA genes	miRNA genes	Other types of small non-coding genes[1]	Pseudo-genes
Bovine (ARS-UCD1.2)	Ensembl (Release 2021-03)	21,880	1,480	951	2,209	492
	NCBI (Release 106)	21,039	5,179	797	3,249	4,569
	Current project	21,116	10,689	2,007	87	3,029
Human (GRCh38.104)	Ensembl (release 2021-03)	20,442	16,876	1,877	2,930	15,266

[1]Small nucleolar RNAs, small non-coding RNAs, small Cajal body specific RNAs, small conditional RNAs, and tRNAs

Table 8. Summary of error-corrected, FLNC Iso-Seq reads and their matched RNA-seq reads

Tissue	Error-corrected FLNC Iso-Seq reads[1]	Median error rate in error-corrected FLNC Iso-Seq reads	Normalized RNA-seq reads used for error correction[2]
Thalamus	664,900 (90%)	0.21%	32,452,612
Testes	711,821 (86%)	1.43%	31,939,024
Liver	1,064,146 (84%)	1.84%	13,657,156
Medulla	380,531 (86%)	0.43%	48,256,918
Subcutaneous fat	215,759 (93%)	0.45%	42,043,313
Cerebral cortex	440,797 (87%)	1.01%	21,285,864
Jejunum	604,436 (90%)	2.331%	34,457,447

[1] Number in parentheses indicates mapping rate (90% coverage and 95% identity).

[2] In silico normalized using insilico_read_normalization.pl from Trinity (version 2.6.6) with the following settings: --max_cov 50 --max_pct_stdev 100 --single

Supplemental files

Supplemental file 1: List of different datasets generated in the experiment.

Supplemental file 2: Fig. S1 Distribution of the number of RNA-seq reads across tissues. **Fig. S2** (A) Comparison of tissues based on number of transcript biotypes and (B) percentage of transcript biotypes. (C) Comparison of transcript biotypes based on their number of expressed tissues and (D) their expression level across expressed tissues. **Fig. S3** (A) Relation between the number of input reads and the number of transcript biotypes (B) Comparison of expression level between different transcript biotypes. **Fig. S4** Tissue similarities (A) and clustering (B) based on the percentage of protein-coding transcripts shared between pairs of tissues. **Fig. S5** Tissue similarities (A) and clustering (B) based on the percentage of non-coding transcripts shared between pairs of tissues. **Fig. S6** Comparison of annotated and un-annotated transcripts based on their expression (A) and number of expressed tissues (B). **Fig. S7** Comparison of annotated and un-annotated protein-coding transcripts based on the length (A) and GC content (B) of their 5' UTR, CDS, and 3' UTR. **Fig. S8** (A) Comparison of tissues based on number of gene biotypes and (B) percentage of gene biotypes. **Fig. S9** Relation between the number of input reads and the number of gene biotypes**. Fig. S10** Comparison of annotated and un-annotated genes based on their expression (A) and number of expressed tissues (B). **Fig. S11** Functional enrichment analysis of the top five percent of genes with the highest number of UTRs. **Fig. S12** Similarity of tissues based on the number of non-coding genes in their fetal samples that switched to protein-coding genes with only coding transcripts in their adult samples. **Fig. S13** (A) Distribution of genes that transcribed PATs, based on their number of expressed tissues, percentage of genes' transcripts that are PATs and percentage of genes' expressed tissues in which PATs were transcribed. (B) Comparison of genes that transcribed PATs with other gene biotypes. **Fig. S14** (A) Homology analysis of protein-coding genes. (B) Homology analysis of non-

coding genes. (C) Detection of orphan genes based on homology classification of cattle-specific protein-coding genes and non-coding genes. **Fig. S15** Comparison of the expression level of homologous and orphan genes across (A) and within (B) their expressed tissues. (C) Comparison of homologous and orphan genes based on the number of expressed tissues. **Fig. S16** Comparison of different gene biotypes based on the expression (A) and the number of expressed tissues (B). **Fig. S17** Comparison of different pseudogene-derived lncRNAs and non-pseudogene derived lncRNAs based on the expression level (A) and the number of expressed tissues (B)**. Fig. S18** Tissue similarities (A) and clustering (B) based on the percentage of protein-coding genes shared between pairs of tissues. **Fig. S19** Tissue similarities (A) and clustering (B) based on the percentage of non-coding genes shared between pairs of tissues. **Fig. S20** (A) Different types of alternative splicing events. (B) Comparison of bovine genome builds based on the number of transcripts that showed any type of alternative splicing (AS) events**. Fig. S21** Comparison of tissues based on the number (A) and the percentage (B) of transcripts that showed different types of alternative splicing events. Comparison of tissues based on the number (C) and the percentage (D) of alternative splicing events**. Fig. S22** (A) Comparison of tissues based on the percentage of transcripts that showed any type of alternative splicing events, spliced transcripts from single-transcript genes, and unspliced transcripts and (B) the relation between the number of input reads and the number of these transcripts across tissues. **Fig. S23** Comparison of transcripts that showed different types of alternative splicing events based on (A) the expression level in the expressed tissues and (B) the number of expressed tissues. **Fig. S24** Transcript biotype switching due to alternative splicing events**. Fig. S25** Comparison of tissues based on the number of alternative splicing events per alternatively

spliced gene**. Fig. S26** (A) Distribution of the number of alternative splicing events per alternatively spliced gene. The 5% quantile is shown using a dashed red line. (B) Functional enrichment analysis of the top five percent of genes with the highest number of alternative splicing events. **Fig. S27** Comparison of the alternative splicing rate between adult and fetal tissues. **Fig. S28** (A) Distribution of gene's number of expressed tissues. Tissue-specific gene biotypes are shown in the pie chart. (B) Distribution of transcript's number of expressed tissues. Tissue-specific transcript biotypes are shown in the pie chart. (C) Comparison of tissues based on the number of tissue-specific genes and transcripts. (D) Comparison of the expression level of tissue-specific genes and transcripts versus their non-tissue-specific counterparts. **Fig. S29** Relationship between tissue specificity and alternative splicing events**. Fig. S30** Relationship between tissue specificity index and the number of multi-tissue expressed genes (A) and transcripts (B). Distribution of tissue specificity indexes in multi-tissue expressed genes (C) and transcripts (D). The 5% quantile is shown using dashed red lines. (E) Functional enrichment analysis of the top five percent of multi-tissue expressed genes with the highest tissue specificity indexes. **Fig. S31** Distribution of QTLs located outside gene borders in relation to the closest expressed gene**. Fig. S32** (A) Distribution of correlation coefficients between *SPACA5* gene expression and pituitary expressed genes closest to "percentage of normal sperm" QTLs. Dashed lines show the minimum significant positive and negative correlation (p-value <0.05). (B) Expression atlas of *SPACA5* gene in human tissues from The Human Protein Atlas [90]. **Fig. S33** (A) Correlation between pituitary genes with signal peptides that were close to the "percentage of normal sperm" QTL and testis expressed genes closest to this trait's QTL (reference correlations). (B) Distribution of p-values resulting from right-sided t-test between

reference correlation coefficients and correlation coefficients derived from random chance (see methods for details)**. Fig. S34** Tissue similarities (A) and clustering (B) based on the percentage of miRNAs shared between pairs of tissues. **Fig. S35** Clustering of tissues based on protein-coding genes (A), protein-coding transcripts (B), non-coding genes (C), non-coding transcripts (D), and miRNAs (E). (F) Comparison of tissue dendrograms based on the correlation between their Cophenetic distances. **Fig. S36** (A) Distribution of the number of expressed tissues for annotated and un-annotated miRNAs. Classification of miRNAs as annotated, or un-annotated is presented in the pie chart. (B) Comparison of tissues based on their number of tissue-specific miRNAs. (C) Expression of annotated and un-annotated miRNAs in their expressed tissues. (D) Distribution of multi-tissue expressed miRNAs' tissue specificity indexes. (E) Relationship between tissue specificity index and number of expressed tissues in multi-tissue expressed miRNAs. Dots have been color coded based on their density. **Fig. S37** Distribution of the number of expressed genes (A), transcripts (B), and miRNAs (C) across tissues. **Fig. S38** Distribution of the number of annotated and un-annotated genes (A), transcripts (B), and miRNAs (C) across tissues. **Fig. S39** Overview of the bioinformatics steps used in this study. **Fig. S40** Graphical representation of the method used to construct the tissue similarity network.

Supplemental file 3: Summary of RNA-seq and miRNA-seq reads.

Supplemental file 4: Detailed description of the number of transcripts, genes, and miRNAs expressed in each tissue.

Supplemental file 5: List of transcripts and genes expressed in each tissue and their expression values (RPKM). Individual tissue files are labeled as: Supplemental_file5_<TISSUE NAME>_<Genes/Transcripts>.tsv

Supplemental file 6: Transcript biotype enrichment analysis in adult and fetal tissues.

Supplemental file 7: Functional enrichment analysis of the top five percent of genes with the highest number of UTRs.

Additional file 8: Functional enrichment analysis of genes that remained bifunctional in all their expressed tissues.

Additional file 9: Functional enrichment analysis of non-coding genes in fetal tissues that were switched to protein coding with only coding transcripts in their matched adult tissue.

Additional file 10: Functional enrichment analysis of protein-coding genes that transcribed PATs as their main transcripts (PATs comprised >50% of their transcripts) in all their expressed tissues.

Supplemental file 11: Gene biotype enrichment analysis in adult and fetal tissues.

Supplemental file 12: Functional enrichment analysis of the top five percent of genes with the highest number of alternative splicing events.

Supplemental file 13: List of tissue-specific genes and transcripts.

Supplemental file 14: Genes and transcripts tissue specificity indexes. Individual tissue files are labeled as: Supplemental_file14_<Genes/Transcripts>.tsv

Supplemental file 15: Functional enrichment analysis of the top five percent of multi-tissue expressed genes with the highest tissue specificity indexes.

Supplemental file 16: List of QTL's closest expressed genes in each tissue. Individual tissue files are labeled as: Supplemental_file16_<TISSUE NAME>.tsv

Supplemental file 17: Trait enrichment analysis of testis-specific genes.

Supplemental file 18: Pituitary expressed genes closest to "percentage of normal sperm" QTLs that showed positive significant correlation with SPACA5 gene in testis.

Supplemental file 19: List of expressed genes closest to "percentage of normal sperm" QTLs that were involved in testis-pituitary tissue axis and their functional enrichment analysis results.

Supplemental file 20: List of genes expressed closest to "percentage of normal sperm" QTLs that were involved in pituitary-testis tissue axis and their functional enrichment analysis results.

Supplemental file 21: Similarity of traits based on the integration of the assembled bovine transcriptome with publicly available QTLs.

Supplemental file 22: List of miRNAs expressed in each tissue and their expression values. Individual tissue files are labeled as: Supplemental_file22_<TISSUE NAME>.tsv

Supplemental file 23: Tissue sample collection and sequencing library preparation methods

Supplemental file 24: List of independent omics datasets used in the experiment.

Abbreviations

A3Es: Alternative 3' splice site Exons; A5Es: Alternative 5' splice site Exons; AFEs: Alternative First Exon; ALEs: Alternative Last Exon; AS: Alternative Splicing; ATAC-seq: Assay for Transposase-Accessible Chromatin using sequencing; bp: base pair; BP: Biological Process; CDS: coding sequence; ChIP-seq: Chromatin Immunoprecipitation Sequencing; CPM: Counts Per Million; CTCF: CCCTC-binding factor; DMEM: Dulbecco’s Modified Eagle Medium; FLNC: Full-Length, Non-Chimeric; GO: Gene Ontology; GOA: Gene Ontology Annotation database; GWAS: Genome-Wide Association Studies; H3K27ac: N-terminal acetylation of lysine 27 on histone H3; H3K4me1: tri-methylation of lysine 4 on histone H1; H3K4me3: tri-methylation of lysine 4 on histone H3; IACUC: Institutional Animal Care and Use Committee; LD: Longissimus Dorsi; lncRNAs: long non-coding RNAs; miRNA: microRNAs; MXEs: Mutually Exclusive Exons; NCBI: National Center for Biotechnology Information; ncRNAs: non-coding RNAs; NMD: Nonsense-Mediated Decay; NSD: Non-Stop Decay; ONT-seq: Oxford Nanopore Technologies sequencing; ORFs: Open Reading Frames; PacBio Iso-Seq: Pacific Biosciences single-molecule long-read isoform sequencing; PAT: Potentially Aberrant Transcript; poly(A): Polyadenylation; PTBP1: polypyrimidine tract binding protein 1; QTL: Quantitative Trait Loci; RAMPAGE: RNA Annotation and Mapping of Promoters for the Analysis of Gene Expression; Ribo-seq: Ribosome footprinting followed by Sequencing; RIEs: Retained Intron Exons; RNA-seq: Illumina high-throughput RNA sequencing; RPKM: Reads Per Kilobase of Transcript per Million reads mapped; RPM: Reads Per Million; SEs: Skipped Exons; sncRNAs: small non-coding RNAs; SNP: Single Nucleotide Polymorphism; tpg: transcripts per annotated gene; TSI: Tissue Specificity Index; TSS: Transcript Start Sites; TTS: Transcript Terminal Sites; UCD: University of California, Davis;

USEs: Unique Splice Site Exons; UTR: untranslated region; WTTS-seq: Whole Transcriptome Termini Site Sequencing.

Data availability

RNA-seq and miRNA-seq, ATAC-seq, and WTTS-seq datasets generated in this study are submitted to the ArrayExpress database [91] under accession numbers E-MTAB-11699, E-MTAB-11815, and E-MTAB-12052, respectively. The constructed bovine trait similarity network is publicly available through the Animal Genome database [92]. The constructed cattle transcriptome and related sequences are publicly available in the Open Science Framework database [93]. Bioinformatics work-follow and custom codes used are available in the GitHub repository [94]. In addition, bioinformatics_workfloow.sh contains all bioinformatics work-follow used in this project. All additional supporting data are available in the GigaScience repository, GigaDB [95]

Ethics approval and consent to participate

Procedures for tissue collection followed the Animal Care and Use protocol (#18464) approved by the Institutional Animal Care and Use Committee (IACUC), University of California, Davis (UCD).

Consent for publication

Not applicable

Competing interests

The authors declare no competing interests.

Funding

This study was supported by Agriculture and Food Research Initiative Competitive Grant no. 2018-67015-27500 (H.Z., P.R. etc.) and sample collection was supported by no. 2015-67015-22940 (H.Z. and P.R.) from the USDA National Institute of Food and Agriculture.

Acknowledgments

We are grateful to Nathan Weeks for helping with massive parallel computing of transcriptome assembly.

Authors' contributions

H.B., B.M.M., H.J., H.Z., M.R., P.J.R., S.M., T.P.L.S., W.L., Z.J., and J.M.R. conceived and designed the project; C.K., W.M., and W.L. generated RNA-seq and miRNA-seq data; D.K., G.B., J.T., and K.D. participated in tissue collection; R.H and H.J prepared cells; J.J.M., X.Z., X.H., and Z.J. generated W.T.T.S-seq data, X.X., P.J.R. and H.J generated ChIP-seq data; M.R.J. generated ATAC-seq data; T.P.L.S. generated PacBio Iso-seq data; G.R. and S.C. conducted sequencing of RNA-seg, miRNA-seq, ChIP-seq, and ATAC-seq data; H.B. conducted bioinformatics data analysis and drafted the manuscript, which was edited by C.A.P., B.M.M., H.J., H.Z., J.E.K., M.R., P.J.R., S.M., T.P.L.S., W.L., Z.J. and J.M.R.; Z.H. created the web-based database for the trait similarity network; all authors read and approved the final manuscript.

Endnotes

Mention of trade names or commercial products in this publication is solely for the purpose of providing specific information and does not imply recommendation or endorsement by the U.S. Department of Agriculture. USDA is an equal opportunity provider and employer.

The results reported here were made possible with resources provided by the USDA shared computing cluster (Ceres) as part of the ARS SCINet initiative.

References

1. Roth JA and Tuggle CK. Livestock models in translational medicine. ILAR J. 2015;56 1:1-6. doi:10.1093/ilar/ilv011.
2. Beiki H, Liu H, Huang J, Manchanda N, Nonneman D, Smith TPL, et al. Improved annotation of the domestic pig genome through integration of Iso-Seq and RNA-seq data. BMC Genomics. 2019;20 1:344. doi:10.1186/s12864-019-5709-y.
3. Marceau A, Gao Y, Baldwin RLt, Li CJ, Jiang J, Liu GE, et al. Investigation of rumen long noncoding RNA before and after weaning in cattle. BMC Genomics. 2022;23 1:531. doi:10.1186/s12864-022-08758-4.
4. Muniz MMM, Simielli Fonseca LF, Scalez DCB, Vega AS, Silva D, Ferro JA, et al. Characterization of novel lncRNA muscle expression profiles associated with meat quality in beef cattle. Evol Appl. 2022;15 4:706-18. doi:10.1111/eva.13365.
5. Li W, Jing Z, Cheng Y, Wang X, Li D, Han R, et al. Analysis of four complete linkage sequence variants within a novel lncRNA located in a growth QTL on chromosome 1 related to growth traits in chickens. J Anim Sci. 2020;98 5 doi:10.1093/jas/skaa122.
6. Watanabe K, Stringer S, Frei O, Umicevic Mirkov M, de Leeuw C, Polderman TJC, et al. A global overview of pleiotropy and genetic architecture in complex traits. Nat Genet. 2019;51 9:1339-48. doi:10.1038/s41588-019-0481-0.
7. Jereb S, Hwang HW, Van Otterloo E, Govek EE, Fak JJ, Yuan Y, et al. Differential 3' Processing of Specific Transcripts Expands Regulatory and Protein Diversity Across Neuronal Cell Types. Elife. 2018;7 doi:10.7554/eLife.34042.
8. Schurch NJ, Cole C, Sherstnev A, Song J, Duc C, Storey KG, et al. Improved annotation of 3' untranslated regions and complex loci by combination of strand-specific direct RNA sequencing, RNA-Seq and ESTs. PLoS One. 2014;9 4:e94270. doi:10.1371/journal.pone.0094270.
9. Ambros V. The functions of animal microRNAs. Nature. 2004;431 7006:350-5. doi:10.1038/nature02871.

10. Bartel DP. MicroRNAs: genomics, biogenesis, mechanism, and function. Cell. 2004;116 2:281-97. doi:10.1016/s0092-8674(04)00045-5.

11. Yates LA, Norbury CJ and Gilbert RJ. The long and short of microRNA. Cell. 2013;153 3:516-9. doi:10.1016/j.cell.2013.04.003.

12. Halstead MM, Islas-Trejo A, Goszczynski DE, Medrano JF, Zhou H and Ross PJ. Large-Scale Multiplexing Permits Full-Length Transcriptome Annotation of 32 Bovine Tissues From a Single Nanopore Flow Cell. Front Genet. 2021;12:664260. doi:10.3389/fgene.2021.664260.

13. Goszczynski DE, Halstead MM, Islas-Trejo AD, Zhou H and Ross PJ. Transcription initiation mapping in 31 bovine tissues reveals complex promoter activity, pervasive transcription, and tissue-specific promoter usage. Genome Res. 2021;31 4:732-44. doi:10.1101/gr.267336.120.

14. Kozomara A, Birgaoanu M and Griffiths-Jones S. miRBase: from microRNA sequences to function. Nucleic Acids Res. 2019;47 D1:D155-D62. doi:10.1093/nar/gky1141.

15. Araujo PR, Yoon K, Ko D, Smith AD, Qiao M, Suresh U, et al. Before It Gets Started: Regulating Translation at the 5' UTR. Comp Funct Genomics. 2012;2012:475731. doi:10.1155/2012/475731.

16. Gerber S, Schratt G and Germain PL. Streamlining differential exon and 3' UTR usage with diffUTR. BMC Bioinformatics. 2021;22 1:189. doi:10.1186/s12859-021-04114-7.

17. Andrews SJ and Rothnagel JA. Emerging evidence for functional peptides encoded by short open reading frames. Nat Rev Genet. 2014;15 3:193-204. doi:10.1038/nrg3520.

18. Kumari P and Sampath K. cncRNAs: Bi-functional RNAs with protein coding and non-coding functions. Semin Cell Dev Biol. 2015;47-48:40-51. doi:10.1016/j.semcdb.2015.10.024.

19. Nam JW, Choi SW and You BH. Incredible RNA: Dual Functions of Coding and Noncoding. Mol Cells. 2016;39 5:367-74. doi:10.14348/molcells.2016.0039.

20. Hong CH, Ho JC and Lee CH. Steroid Receptor RNA Activator, a Long Noncoding RNA, Activates p38, Facilitates Epithelial-Mesenchymal Transformation, and Mediates Experimental Melanoma Metastasis. J Invest Dermatol. 2020;140 7:1355-63 e1. doi:10.1016/j.jid.2019.09.028.

21. Gonzàlez-Porta M, Frankish A, Rung J, Harrow J and Brazma A. Transcriptome analysis of human tissues and cell lines reveals one dominant transcript per gene. Genome Biol. 2013;14 7:R70. doi:10.1186/gb-2013-14-7-r70.

22. Mayba O, Gilbert HN, Liu J, Haverty PM, Jhunjhunwala S, Jiang Z, et al. MBASED: allele-specific expression detection in cancer tissues and cell lines. Genome Biol. 2014;15 8:405. doi:10.1186/s13059-014-0405-3.

23. Hubé F, Velasco G, Rollin J, Furling D and Francastel C. Steroid receptor RNA activator protein binds to and counteracts SRA RNA-mediated activation of MyoD and muscle differentiation. Nucleic Acids Res. 2011;39 2:513-25. doi:10.1093/nar/gkq833.

24. Kurosaki T, Popp MW and Maquat LE. Quality and quantity control of gene expression by nonsense-mediated mRNA decay. Nat Rev Mol Cell Biol. 2019;20 7:406-20. doi:10.1038/s41580-019-0126-2.

25. Wollerton MC, Gooding C, Wagner EJ, Garcia-Blanco MA and Smith CW. Autoregulation of polypyrimidine tract binding protein by alternative splicing leading to nonsense-mediated decay. Mol Cell. 2004;13 1:91-100. doi:10.1016/s1097-2765(03)00502-1.
26. Nickless A, Bailis JM and You Z. Control of gene expression through the nonsense-mediated RNA decay pathway. Cell Biosci. 2017;7:26. doi:10.1186/s13578-017-0153-7.
27. Supek F, Lehner B and Lindeboom RGH. To NMD or Not To NMD: Nonsense-Mediated mRNA Decay in Cancer and Other Genetic Diseases. Trends Genet. 2021;37 7:657-68. doi:10.1016/j.tig.2020.11.002.
28. Mitrovich QM and Anderson P. mRNA surveillance of expressed pseudogenes in C. elegans. Curr Biol. 2005;15 10:963-7. doi:10.1016/j.cub.2005.04.055.
29. Colombo M, Karousis ED, Bourquin J, Bruggmann R and Mühlemann O. Transcriptome-wide identification of NMD-targeted human mRNAs reveals extensive redundancy between SMG6- and SMG7-mediated degradation pathways. RNA. 2017;23 2:189-201. doi:10.1261/rna.059055.116.
30. Milligan MJ and Lipovich L. Pseudogene-derived lncRNAs: emerging regulators of gene expression. Front Genet. 2014;5:476. doi:10.3389/fgene.2014.00476.
31. Stewart GL, Enfield KSS, Sage AP, Martinez VD, Minatel BC, Pewarchuk ME, et al. Aberrant Expression of Pseudogene-Derived lncRNAs as an Alternative Mechanism of Cancer Gene Regulation in Lung Adenocarcinoma. Front Genet. 2019;10:138. doi:10.3389/fgene.2019.00138.
32. Lou W, Ding B and Fu P. Pseudogene-Derived lncRNAs and Their miRNA Sponging Mechanism in Human Cancer. Front Cell Dev Biol. 2020;8:85. doi:10.3389/fcell.2020.00085.
33. Anderson DM, Anderson KM, Chang CL, Makarewich CA, Nelson BR, McAnally JR, et al. A micropeptide encoded by a putative long noncoding RNA regulates muscle performance. Cell. 2015;160 4:595-606. doi:10.1016/j.cell.2015.01.009.
34. Mackowiak SD, Zauber H, Bielow C, Thiel D, Kutz K, Calviello L, et al. Extensive identification and analysis of conserved small ORFs in animals. Genome Biol. 2015;16:179. doi:10.1186/s13059-015-0742-x.
35. Olexiouk V, Crappé J, Verbruggen S, Verhegen K, Martens L and Menschaert G. sORFs.org: a repository of small ORFs identified by ribosome profiling. Nucleic Acids Res. 2016;44 D1:D324-9. doi:10.1093/nar/gkv1175.
36. Li J and Liu C. Coding or Noncoding, the Converging Concepts of RNAs. Front Genet. 2019;10:496. doi:10.3389/fgene.2019.00496.
37. Wei L-H and Guo JU. Coding functions of “noncoding” RNAs. Science. 2020;367 6482:1074-5. doi:10.1126/science.aba6117.
38. Sammeth M, Foissac S and Guigó R. A general definition and nomenclature for alternative splicing events. PLoS Comput Biol. 2008;4 8:e1000147. doi:10.1371/journal.pcbi.1000147.
39. Mazin PV, Khaitovich P, Cardoso-Moreira M and Kaessmann H. Alternative splicing during mammalian organ development. Nature Genetics. 2021;53 6:925-34. doi:10.1038/s41588-021-00851-w.

40. Wu Z, Yang KK, Liszka MJ, Lee A, Batzilla A, Wernick D, et al. Signal Peptides Generated by Attention-Based Neural Networks. ACS Synth Biol. 2020;9 8:2154-61. doi:10.1021/acssynbio.0c00219.
41. Chen J and Chen ZJ. Regulation of NF-κB by ubiquitination. Curr Opin Immunol. 2013;25 1:4-12. doi:10.1016/j.coi.2012.12.005.
42. Karalis KP, Venihaki M, Zhao J, van Vlerken LE and Chandras C. NF-kappaB participates in the corticotropin-releasing, hormone-induced regulation of the pituitary proopiomelanocortin gene. J Biol Chem. 2004;279 12:10837-40. doi:10.1074/jbc.M313063200.
43. O'Shaughnessy PJ, Fleming LM, Jackson G, Hochgeschwender U, Reed P and Baker PJ. Adrenocorticotropic hormone directly stimulates testosterone production by the fetal and neonatal mouse testis. Endocrinology. 2003;144 8:3279-84. doi:10.1210/en.2003-0277.
44. Richburg JH, Myers JL and Bratton SB. The role of E3 ligases in the ubiquitin-dependent regulation of spermatogenesis. Semin Cell Dev Biol. 2014;30:27-35. doi:10.1016/j.semcdb.2014.03.001.
45. Kumar S, Lee HJ, Park HS and Lee K. Testis-Specific GTPase (TSG): An oligomeric protein. BMC Genomics. 2016;17 1:792. doi:10.1186/s12864-016-3145-9.
46. Rajala-Schultz PJ, Gröhn YT, McCulloch CE and Guard CL. Effects of clinical mastitis on milk yield in dairy cows. J Dairy Sci. 1999;82 6:1213-20. doi:10.3168/jds.S0022-0302(99)75344-0.
47. Martí De Olives A, Díaz JR, Molina MP and Peris C. Quantification of milk yield and composition changes as affected by subclinical mastitis during the current lactation in sheep. J Dairy Sci. 2013;96 12:7698-708. doi:10.3168/jds.2013-6998.
48. Halasa T and Kirkeby C. Differential Somatic Cell Count: Value for Udder Health Management. Front Vet Sci. 2020;7:609055. doi:10.3389/fvets.2020.609055.
49. Remnant J, Green MJ, Huxley J, Hirst-Beecham J, Jones R, Roberts G, et al. Association of lameness and mastitis with return-to-service oestrus detection in the dairy cow. Vet Rec. 2019;185 14:442. doi:10.1136/vr.105535.
50. Miles AM, McArt JAA, Leal Yepes FA, Stambuk CR, Virkler PD and Huson HJ. Udder and teat conformational risk factors for elevated somatic cell count and clinical mastitis in New York Holsteins. Prev Vet Med. 2019;163:7-13. doi:10.1016/j.prevetmed.2018.12.010.
51. Lima FS, Silvestre FT, Peñagaricano F and Thatcher WW. Early genomic prediction of daughter pregnancy rate is associated with improved reproductive performance in Holstein dairy cows. J Dairy Sci. 2020;103 4:3312-24. doi:10.3168/jds.2019-17488.
52. Hertl JA, Schukken YH, Tauer LW, Welcome FL and Gröhn YT. Does clinical mastitis in the first 100 days of lactation 1 predict increased mastitis occurrence and shorter herd life in dairy cows? J Dairy Sci. 2018;101 3:2309-23. doi:10.3168/jds.2017-12615.
53. Kaniyamattam K, De Vries A, Tauer LW and Gröhn YT. Economics of reducing antibiotic usage for clinical mastitis and metritis through genomic selection. J Dairy Sci. 2020;103 1:473-91. doi:10.3168/jds.2018-15817.

54. Green TC, Jago JG, Macdonald KA and Waghorn GC. Relationships between residual feed intake, average daily gain, and feeding behavior in growing dairy heifers. J Dairy Sci. 2013;96 5:3098-107. doi:10.3168/jds.2012-6087.
55. Elolimy AA, Abdelmegeid MK, McCann JC, Shike DW and Loor JJ. Residual feed intake in beef cattle and its association with carcass traits, ruminal solid-fraction bacteria, and epithelium gene expression. J Anim Sci Biotechnol. 2018;9:67. doi:10.1186/s40104-018-0283-8.
56. Weber C, Hametner C, Tuchscherer A, Losand B, Kanitz E, Otten W, et al. Variation in fat mobilization during early lactation differently affects feed intake, body condition, and lipid and glucose metabolism in high-yielding dairy cows. J Dairy Sci. 2013;96 1:165-80. doi:10.3168/jds.2012-5574.
57. Yi Z, Li X, Luo W, Xu Z, Ji C, Zhang Y, et al. Feed conversion ratio, residual feed intake and cholecystokinin type A receptor gene polymorphisms are associated with feed intake and average daily gain in a Chinese local chicken population. J Anim Sci Biotechnol. 2018;9:50. doi:10.1186/s40104-018-0261-1.
58. Liu E and VandeHaar MJ. Relationship of residual feed intake and protein efficiency in lactating cows fed high- or low-protein diets. J Dairy Sci. 2020;103 4:3177-90. doi:10.3168/jds.2019-17567.
59. Clare M, Richard P, Kate K, Sinead W, Mark M and David K. Residual feed intake phenotype and gender affect the expression of key genes of the lipogenesis pathway in subcutaneous adipose tissue of beef cattle. J Anim Sci Biotechnol. 2018;9:68. doi:10.1186/s40104-018-0282-9.
60. Houlahan K, Schenkel FS, Hailemariam D, Lassen J, Kargo M, Cole JB, et al. Effects of Incorporating Dry Matter Intake and Residual Feed Intake into a Selection Index for Dairy Cattle Using Deterministic Modeling. Animals (Basel). 2021;11 4 doi:10.3390/ani11041157.
61. Krueger F: https://www.bioinformatics.babraham.ac.uk/projects/trim_galore/. (2019).
62. Dobin A, Davis CA, Schlesinger F, Drenkow J, Zaleski C, Jha S, et al. STAR: ultrafast universal RNA-seq aligner. Bioinformatics. 2013;29 1:15-21. doi:10.1093/bioinformatics/bts635.
63. Liao Y, Smyth GK and Shi W. featureCounts: an efficient general purpose program for assigning sequence reads to genomic features. Bioinformatics. 2014;30 7:923-30. doi:10.1093/bioinformatics/btt656.
64. Leek J, Johnson W, Parker HS, Fertig EJ, Jaffe AE, Zhang Y, et al. *sva: Surrogate Variable Analysis* . R package version 3.30.0. 2021.
65. Grabherr MG, Haas BJ, Yassour M, Levin JZ, Thompson DA, Amit I, et al. Full-length transcriptome assembly from RNA-Seq data without a reference genome. Nat Biotechnol. 2011;29 7:644-52. doi:10.1038/nbt.1883.
66. Hass B: https://hpcgridrunner.github.io/. (2015).
67. Tange O: GNU Parallel. https://doi.org/10.5281/zenodo.1146014. (2018).
68. Wu TD and Watanabe CK. GMAP: a genomic mapping and alignment program for mRNA and EST sequences. Bioinformatics. 2005;21 9:1859-75. doi:10.1093/bioinformatics/bti310.

69. PacificBiosciences: https://www.pacb.com/products-and-services/analytical-software/smrt-analysis/. (2018).
70. Pedersen BS and Quinlan AR. Mosdepth: quick coverage calculation for genomes and exomes. Bioinformatics. 2018;34 5:867-8. doi:10.1093/bioinformatics/btx699.
71. Hackl T, Hedrich R, Schultz J and Förster F. proovread: large-scale high-accuracy PacBio correction through iterative short read consensus. Bioinformatics. 2014;30 21:3004-11. doi:10.1093/bioinformatics/btu392.
72. Wang JR, Holt J, McMillan L and Jones CD. FMLRC: Hybrid long read error correction using an FM-index. BMC Bioinformatics. 2018;19 1:50. doi:10.1186/s12859-018-2051-3.
73. Wheeler DL, Church DM, Federhen S, Lash AE, Madden TL, Pontius JU, et al. Database resources of the National Center for Biotechnology. Nucleic Acids Res. 2003;31 1:28-33. doi:10.1093/nar/gkg033.
74. Aken BL, Ayling S, Barrell D, Clarke L, Curwen V, Fairley S, et al. The Ensembl gene annotation system. Database (Oxford). 2016;2016 doi:10.1093/database/baw093.
75. Kang YJ, Yang DC, Kong L, Hou M, Meng YQ, Wei L, et al. CPC2: a fast and accurate coding potential calculator based on sequence intrinsic features. Nucleic Acids Res. 2017;45 W1:W12-W6. doi:10.1093/nar/gkx428.
76. Zhou X, Li R, Michal JJ, Wu XL, Liu Z, Zhao H, et al. Accurate Profiling of Gene Expression and Alternative Polyadenylation with Whole Transcriptome Termini Site Sequencing (WTTS-Seq). Genetics. 2016;203 2:683-97. doi:10.1534/genetics.116.188508.
77. Salmela L and Schröder J. Correcting errors in short reads by multiple alignments. Bioinformatics. 2011;27 11:1455-61. doi:10.1093/bioinformatics/btr170.
78. Hannon GJ: FASTX-Toolkit. http://hannonlab.cshl.edu/fastx_toolkit. (2010).
79. Kern C, Wang Y, Xu X, Pan Z, Halstead M, Chanthavixay G, et al. Functional annotations of three domestic animal genomes provide vital resources for comparative and agricultural research. Nat Commun. 2021;12 1:1821. doi:10.1038/s41467-021-22100-8.
80. Bindea G, Mlecnik B, Hackl H, Charoentong P, Tosolini M, Kirilovsky A, et al. ClueGO: a Cytoscape plug-in to decipher functionally grouped gene ontology and pathway annotation networks. Bioinformatics. 2009;25 8:1091-3. doi:10.1093/bioinformatics/btp101.
81. Huntley RP, Sawford T, Mutowo-Meullenet P, Shypitsyna A, Bonilla C, Martin MJ, et al. The GOA database: gene Ontology annotation updates for 2015. Nucleic Acids Res. 2015;43 Database issue:D1057-63. doi:10.1093/nar/gku1113.
82. Kim KI and van de Wiel MA. Effects of dependence in high-dimensional multiple testing problems. BMC Bioinformatics. 2008;9 1:114. doi:10.1186/1471-2105-9-114.
83. Huang DW, Sherman BT, Tan Q, Collins JR, Alvord WG, Roayaei J, et al. The DAVID Gene Functional Classification Tool: a novel biological module-centric algorithm to functionally analyze large gene lists. Genome Biol. 2007;8 9:R183. doi:10.1186/gb-2007-8-9-r183.
84. Trincado JL, Entizne JC, Hysenaj G, Singh B, Skalic M, Elliott DJ, et al. SUPPA2: fast, accurate, and uncertainty-aware differential splicing analysis across multiple conditions. Genome Biol. 2018;19 1:40. doi:10.1186/s13059-018-1417-1.

85. Friedländer MR, Mackowiak SD, Li N, Chen W and Rajewsky N. miRDeep2 accurately identifies known and hundreds of novel microRNA genes in seven animal clades. Nucleic Acids Res. 2012;40 1:37-52. doi:10.1093/nar/gkr688.

86. Ludwig N, Leidinger P, Becker K, Backes C, Fehlmann T, Pallasch C, et al. Distribution of miRNA expression across human tissues. Nucleic Acids Res. 2016;44 8:3865-77. doi:10.1093/nar/gkw116.

87. Hu ZL, Park CA and Reecy JM. Building a livestock genetic and genomic information knowledgebase through integrative developments of Animal QTLdb and CorrDB. Nucleic Acids Res. 2019;47 D1:D701-D10. doi:10.1093/nar/gky1084.

88. Shannon P, Markiel A, Ozier O, Baliga NS, Wang JT, Ramage D, et al. Cytoscape: a software environment for integrated models of biomolecular interaction networks. Genome Res. 2003;13 11:2498-504. doi:10.1101/gr.1239303.

89. Almagro Armenteros JJ, Tsirigos KD, Sønderby CK, Petersen TN, Winther O, Brunak S, et al. SignalP 5.0 improves signal peptide predictions using deep neural networks. Nature Biotechnology. 2019;37 4:420-3. doi:10.1038/s41587-019-0036-z.

90. Uhlén M, Fagerberg L, Hallström BM, Lindskog C, Oksvold P, Mardinoglu A, et al. Proteomics. Tissue-based map of the human proteome. Science. 2015;347 6220:1260419. doi:10.1126/science.1260419.

91. ArrayExpress database. https://www.ebi.ac.uk/biostudies/arrayexpress.

92. Animal Genome database. https://www.animalgenome.org/host/reecylab/a.

93. Reecy, J, Beiki, H, & Hu, Z. Cattle FAANG Project. OSF. 2024. https://doi.org/10.17605/OSF.IO/JZE72

94. GitHub repository. https://github.com/hamidbeiki/Cattle-Genome.

95. Beiki H, Murdoch BM, Park CA, Kern C, Kontechy D, Becker G, et al. Supporting data for "Enhanced Bovine Genome Annotation Through Integration of Transcriptomics and Epi-Genetics Datasets Facilitates Genomic Biology" GigaScience Database. 2024. http://dx.doi.org/10.5524/102496

Figure 1

Click here to access/download;

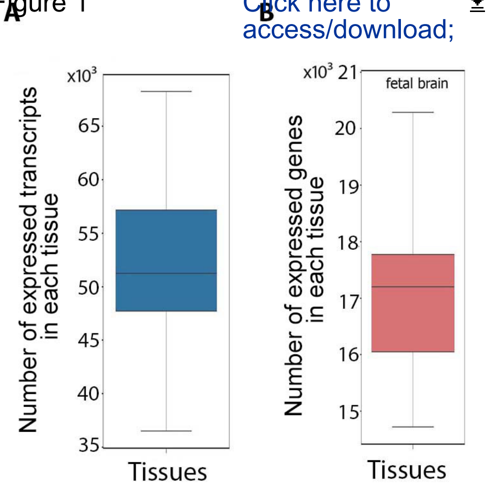

Figure 2

Click here to access/download;Figure;Fig_2.pdf

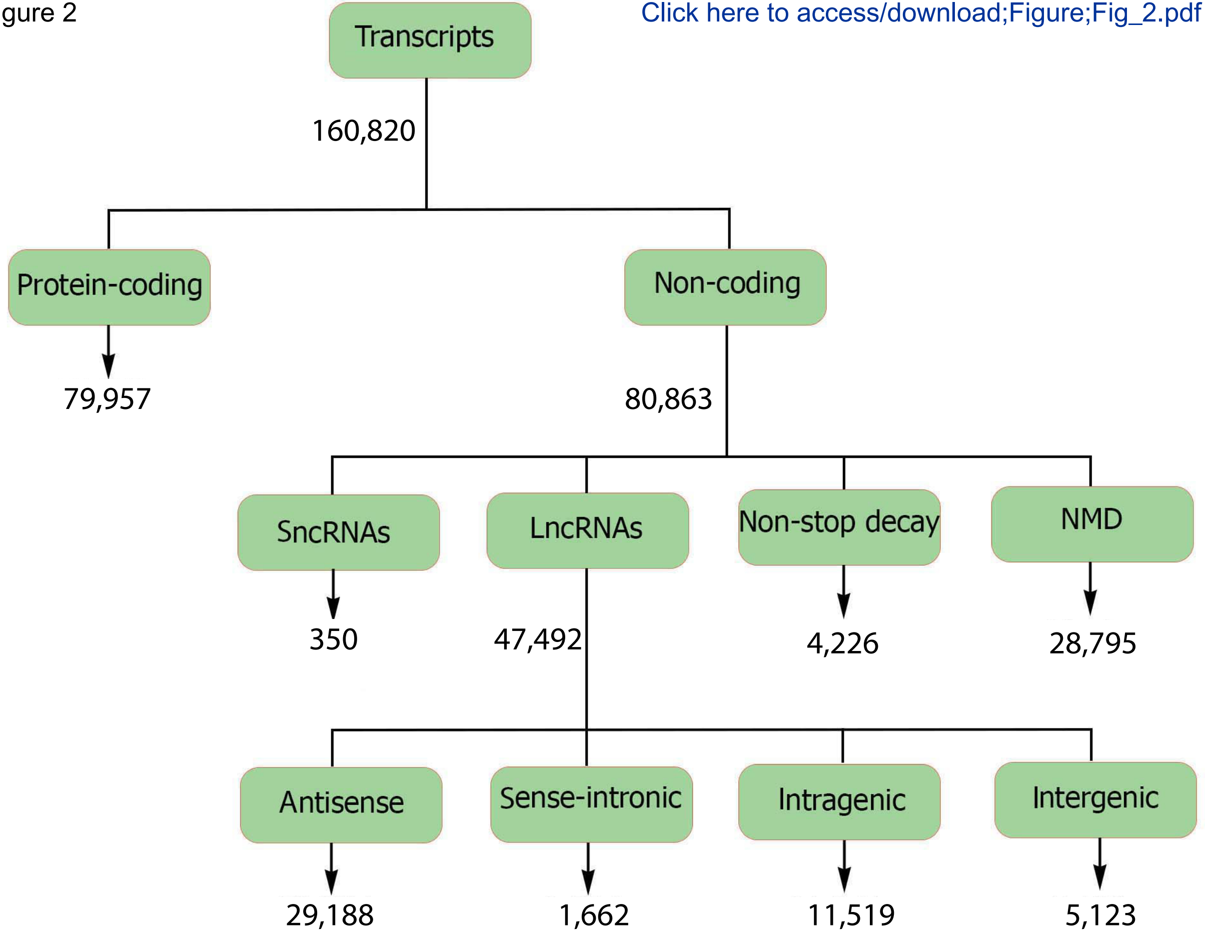

Click here to access/download;Figure;Fig_3.pdf

Figure 3

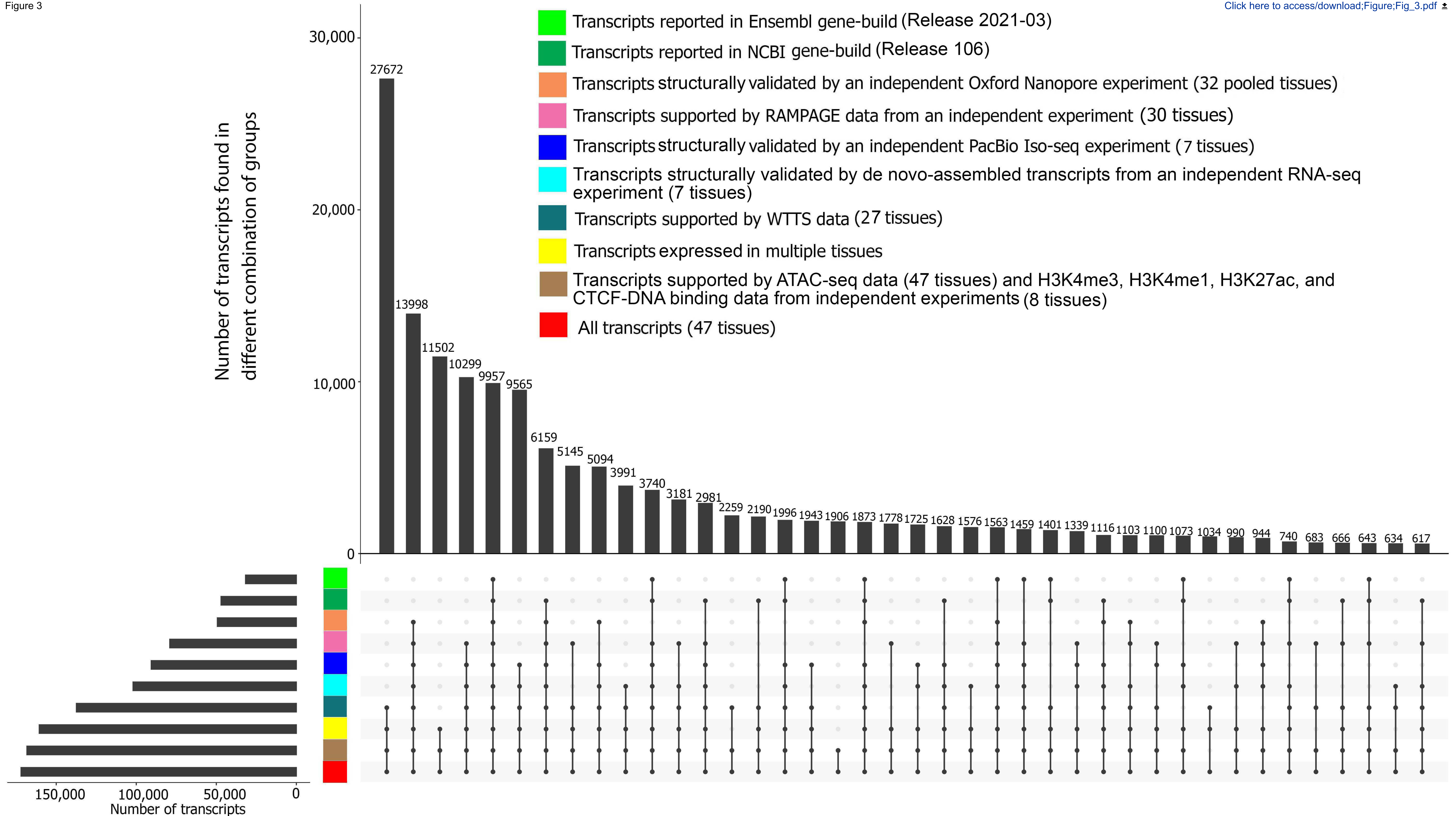

Figure 4

Click here to access/download;Figure;Fig_4.pdf

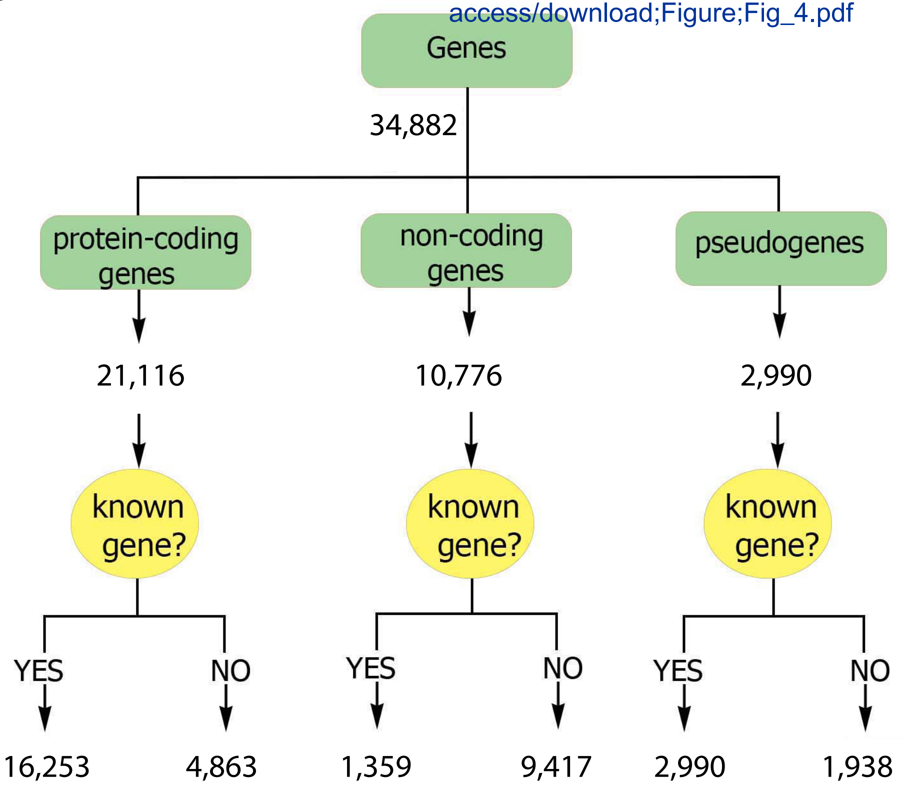

Figure 5

Click here to access/downl

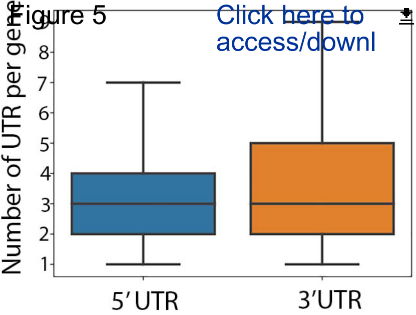

Figure 6

Click here to access/download;Figure;Fig_6.pdf

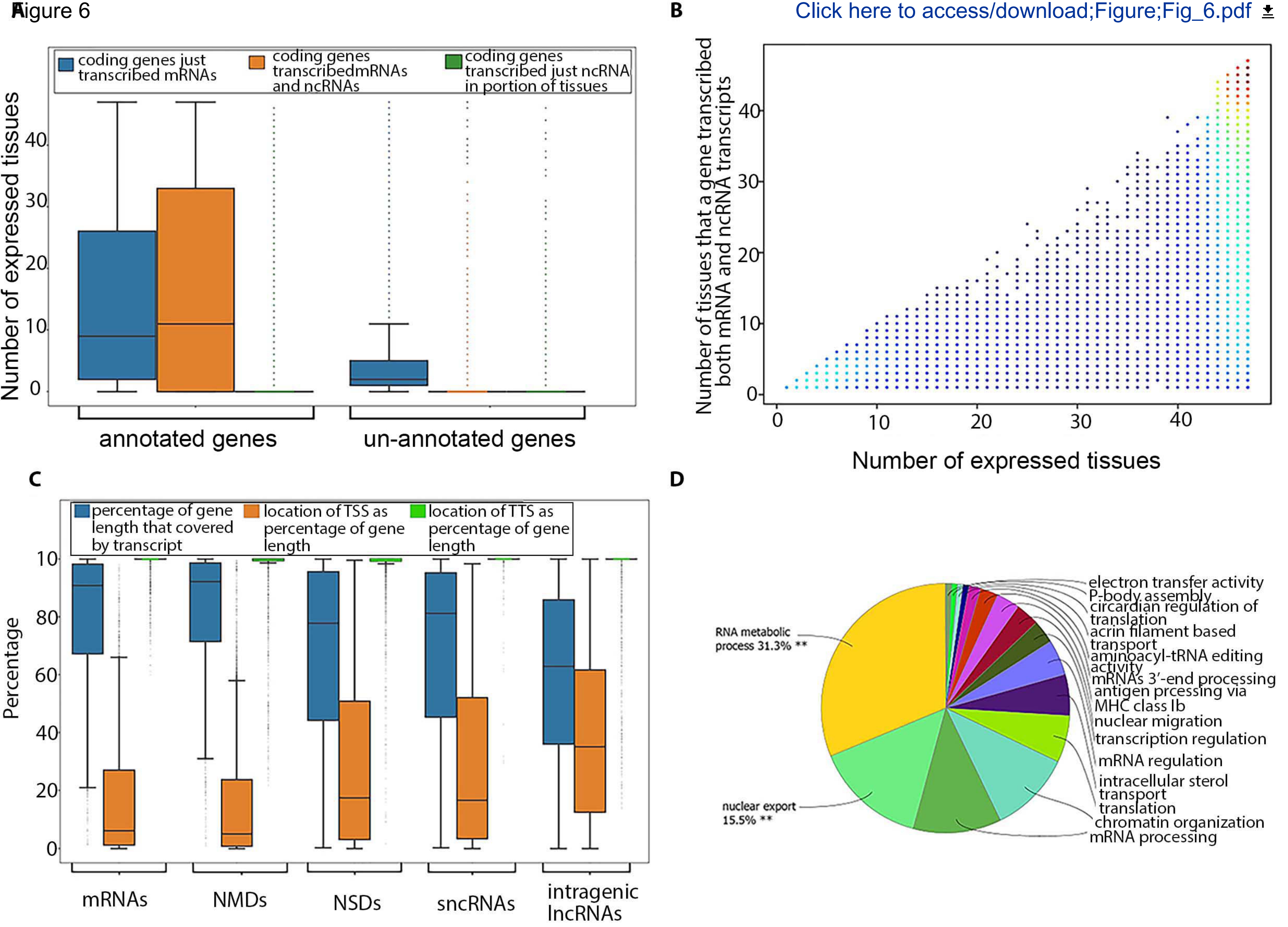

Figure 7

Click here to access/download;Figure;Fig_7.pdf

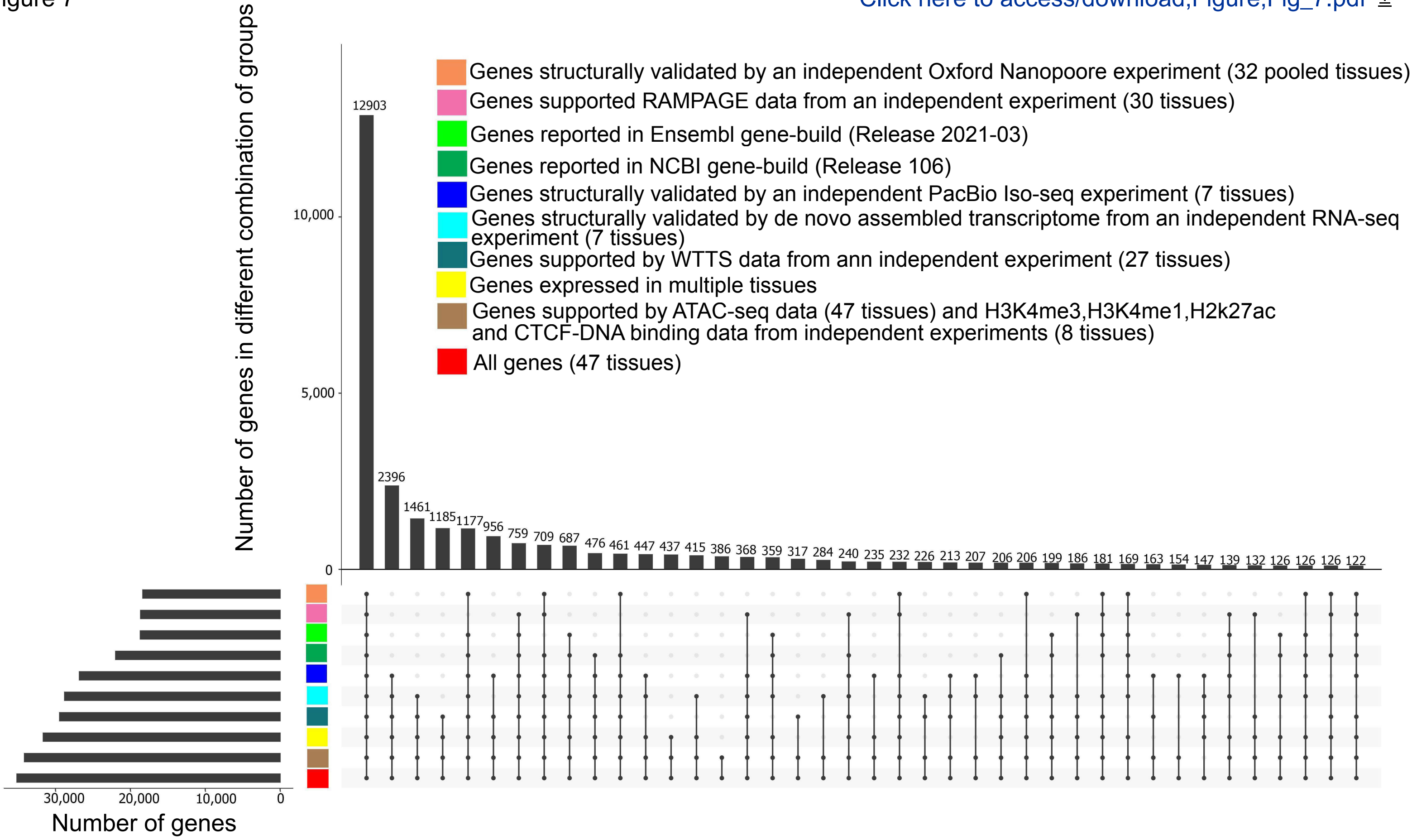

Figure 8

Click here to access/download;Figure;Fig_8.pdf

A

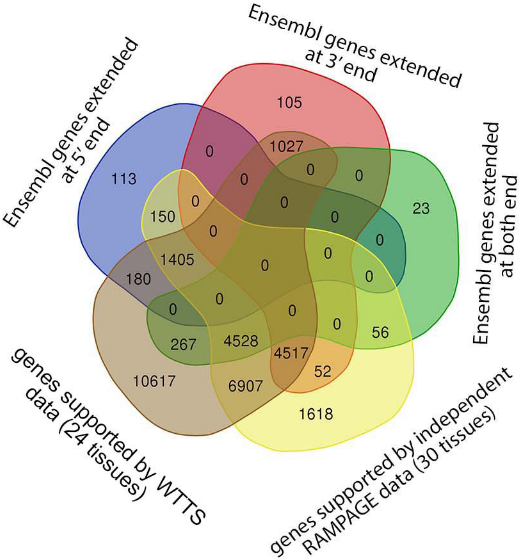

B

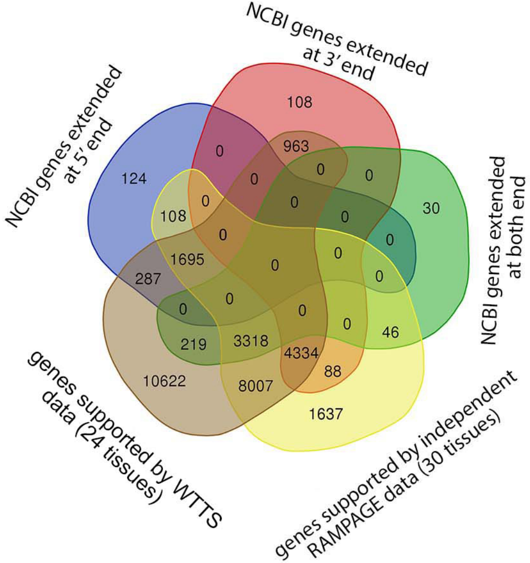

Figure 9 Click here to access/download;Figure;Fig_9.pdf

A

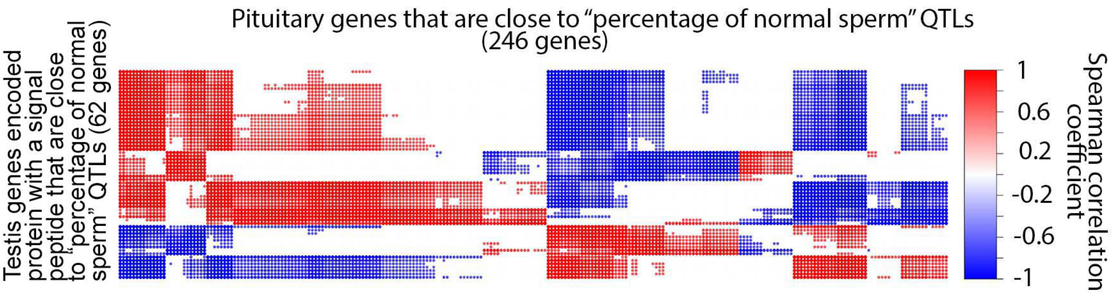

B

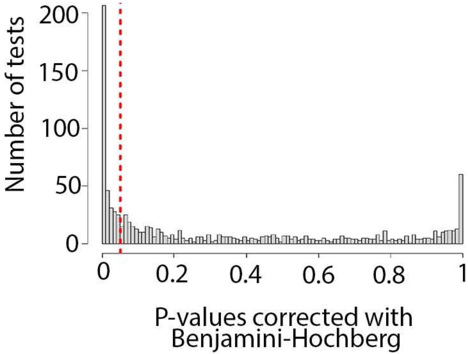

Figure 10

Click here to access/download;Figure;Fig_10.p

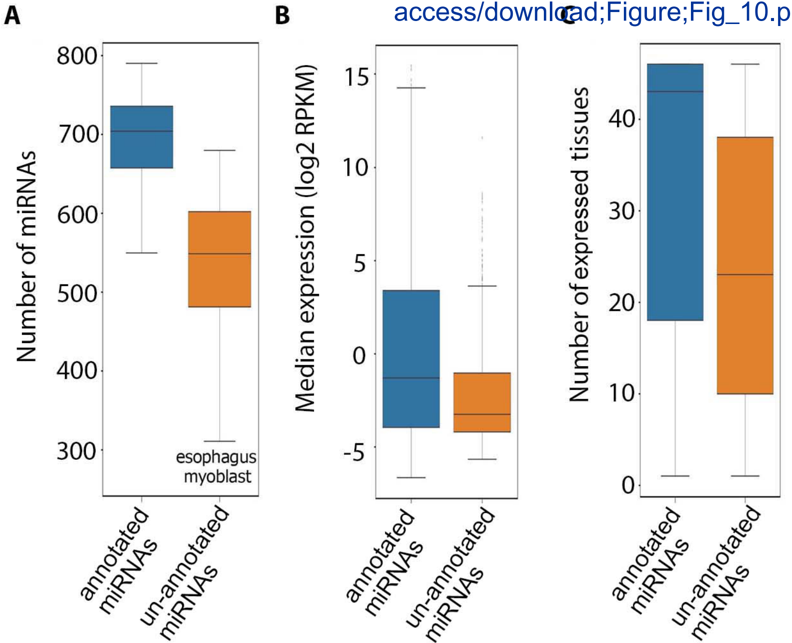

Figure 11

Click here to access/download;Figure;Fig_11.pdf

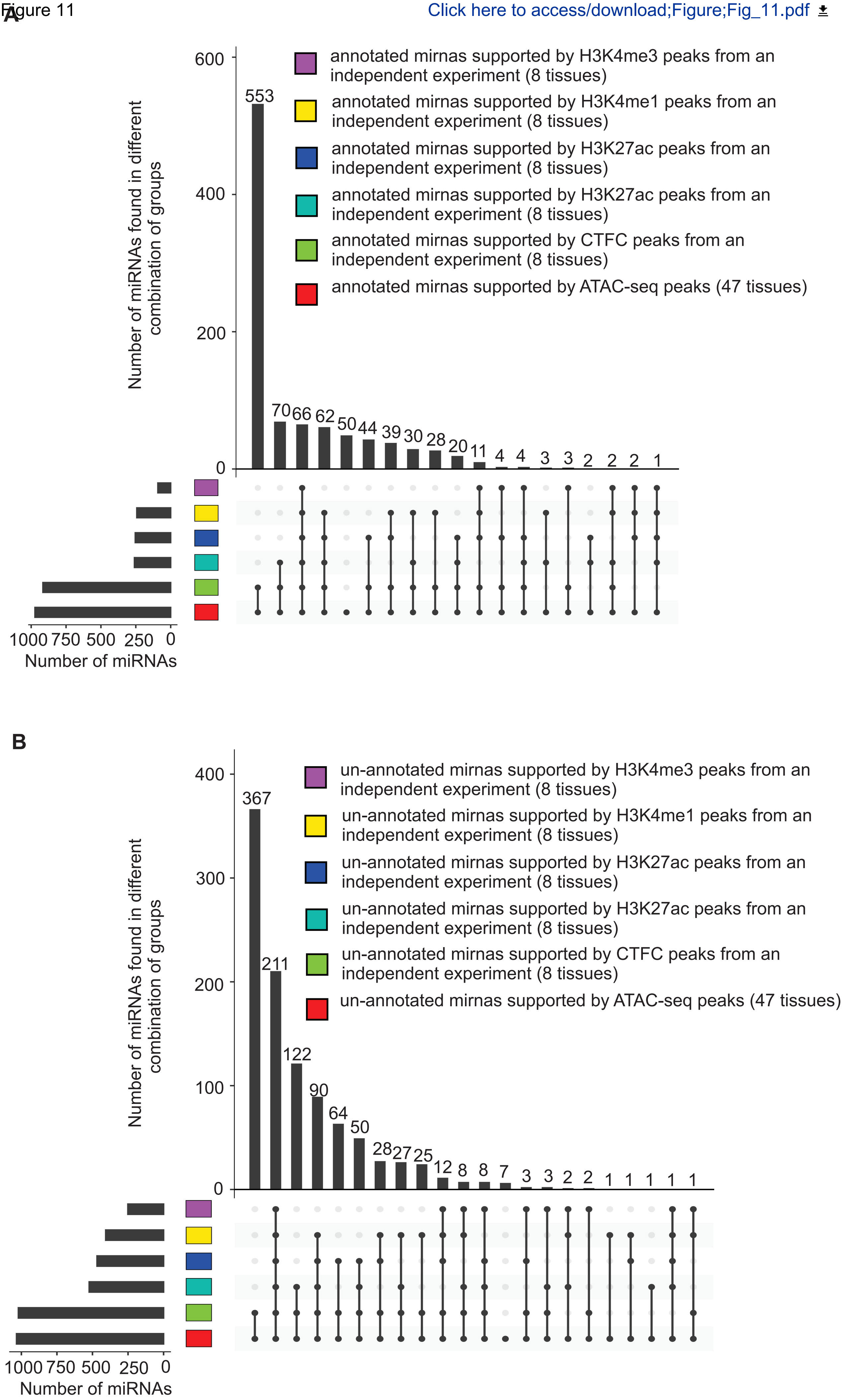

Supplementary File 1

Click here to access/download
Supplementary Material
Supplemental_file1 (1).tsv

Click here to access/download
Supplementary Material
Supplemental_file2 (1).docx

Supplementary Material File 3

Click here to access/download
Supplementary Material
Supplemental_file3.xlsx

Supplementary Material File 4

Click here to access/download
Supplementary Material
Supplemental_file4 (1).xlsx

Supplementary Material File 5

Click here to access/download
Supplementary Material
Supplemental_file5.xlsx

Supplementary Material File 6

Click here to access/download
Supplementary Material
Supplemental_file6.xlsx

Supplementary Material File 7

Click here to access/download
Supplementary Material
Supplemental_file7.xlsx

Supplementary Material File 8

Click here to access/download
Supplementary Material
Supplemental_file8.xlsx

Supplementary Material File 9

Click here to access/download
Supplementary Material
Supplemental_file9.xlsx

Supplementary Material File 10

Click here to access/download
Supplementary Material
Supplemental_file10.xlsx

Click here to access/download
Supplementary Material
Supplemental_file11.xlsx

Supplementary Material File 12

Click here to access/download
Supplementary Material
Supplemental_file12.xlsx

Click here to access/download
Supplementary Material
Supplemental_file13.xlsx

Supplemental File 14

Click here to access/download
Supplementary Material
Supplemental_file14.xlsx

Supplementary Material File 15

Click here to access/download
Supplementary Material
Supplemental_file15.xlsx

Supplemental File 16

Click here to access/download
Supplementary Material
Supplemental_file16.xlsx

Click here to access/download
Supplementary Material
Supplemental_file17.xlsx

Supplementary Material File 18

Click here to access/download
Supplementary Material
Supplemental_file18.xlsx

Click here to access/download
Supplementary Material
Supplemental_file19.xlsx

Supplementary Material File 20

Click here to access/download
Supplementary Material
Supplemental_file20.xlsx

Click here to access/download
Supplementary Material
Supplemental_file21.xlsx

Supplemental File 22

Click here to access/download
Supplementary Material
Supplemental_file22.xlsx

Supplementary File 23

Click here to access/download
Supplementary Material
Supplemental_file23 (1).docx

Supplementary Material File 24

Click here to access/download
Supplementary Material
Supplemental_file24.xlsx

 Click here to access/download;Personal Cover;Response to reviewer comments (1).docx

Dear Editor

Manuscript number: GIGA-D-23-00037

We are thankful to the reviewers for their thorough review. We have revised the present research manuscript in the light of their useful suggestions and comments. We hope this revision has improved the manuscript to a level of their satisfaction. Point by point answers to their specific comments are as follows.

Reviewer#1

Comment 1: The authors updated the manuscript title to "Improved annotation of the bovine genome identifies relationships between phenotypic traits". The study just searches for overlapping between the transcripts and publicly available QTL information. This approach helps to better understand the putative function of this transcript. However, it was not tested real associations between these transcripts and the traits. I would suggest the authors review the title of the manuscript. In my opinion, the study is much more focused on the improved annotation of the bovine genome and a screening of transcript isoforms than on the relationship between traits.

Response: The manuscript title was revised to “Enhancing Bovine Genome Annotation Throughout Integration of Transcriptomics and Epi-Transcriptomics Datasets Facilitates Genomic Biology”

Comment 2: The changes in the discussion section were not tracked, which resulted in difficulty in following the edits.

Response: We are sorry for the confusion that this created.

Comment 3: In the conclusion, the authors mentioned that: "The integrated transcriptome data with publicly available QTL data revealed putative molecular pathways that may underlie tissue-tissue communication mechanisms and candidate genes responsible for the genetic mechanisms that may underlie genetic correlations between traits". It is not clear how the authors found this relationship between the QTLs and molecular pathways. The authors mentioned in the discussion section the analysis of the interconnection between testis and pituitary tissues with respect to the "percentage of normal sperm" and a potential association with a specific GO term. First, not necessarily a GO term represents a molecular pathway. Additionally, the authors mention only this example in the discussion section. The authors should provide a more comprehensive discussion about this approach and how the other results support potential associations between traits, mainly in the light of the next paragraph, where the results of the trait similarity results are discussed.

Response:

We hypothesized that the integration of the gene/transcript data with previously published QTL/gene association data would allow for the identification of potential molecular mechanisms responsible for a) tissue-tissue communication as well as b) genetic correlations between traits (lines 511-514). To test the first hypothesis, we developed a novel approach to study the involvement of tissue-tissue interconnection in different traits based on the integration of the transcriptome with publicly available QTL data (lines 514-516). In particular, the interconnection between testis and pituitary tissues with respect to the "percentage of normal sperm" trait was investigated in more detail based on three reasons: (1) testis tissue showed the highest number of tissue-specific genes compared to the rest of the tissues (Supplemental file 2: Fig. S4, Fig. S5, Fig. S18, and Fig. S19), and these genes were highly enriched with fertility related traits such as percentage of normal sperm (Supplemental file 17) (lines 386-388)., (2) the SPACA5 , a testis-specific gene, encoded protein with a signal peptide (SP) that was close to the "percentage of normal sperm" QTLs (lines 391-392). The expression of this gene in testis samples showed significant positive correlation with 70 pituitary expressed genes that were closest to the "percentage of normal sperm" QTLs (Supplemental file 2: Fig. S32, Supplemental file 18) (lines 392-395)., (3) there is a well-established hormonal interrelation between pituitary gland and testis. Our analysis resulted in the identification of the regulation of ubiquitin-dependent protein catabolic process, the regulation of nuclear factor-κB (NF-κB) transcription factor activity, and Rab protein signal transduction as key components of this tissue-tissue interaction (Supplemental file 19 and 20) (lines 518-521). Activation of NF-κB requires ubiquitination, and this modification is highly conserved across different species (lines 529-530). NF-κB induces secretion of adrenocorticotropic hormone from the pituitary, which directly stimulates testosterone production by the testis (lines 530-532). In addition, ubiquitinated proteins in testis cells are required for the progression of mature spermatozoa (lines 532-533). The expression levels of pituitary expressed genes closest to "percentage of normal sperm" QTLs that also encoded signal peptides were significantly correlated with expression levels of testis expressed genes closest to "percentage of normal sperm" QTLs (Supplemental file 2: Fig. S33) (lines 533-536). These testis genes were highly enriched for the "Rab protein signal transduction" BP GO term (Supplemental file 20). Rab proteins have been reported to be involved in male germ cell development (lines 536-538). These results clearly show that our new approach is supported by the biology of traits and Gene Ontology (GO) terms. Thus, it appears that integration of gene data with QTL/association data can be used to identify putative molecular pathways underlying tissue-tissue communication mechanisms (lines 538-540). The limitations of this approach have been discussed in lines 557-561.

Reviewer#3

Comment 1: Please ensure the data provided in the private dropbox area of GigaDB (user115) is correct with regards to the revised manuscript.

Response: All data provided to the GigaDB are accurate and reflect the most recent version of the manuscript. We have however not received confirmation from GigaDB that the revised files have been received.

Comment 2: In the abstract it is stated "A total number of 171,985 unique transcripts (50% protein-coding) representing 35,150 unique genes (64% protein-coding)".
The supplemental_file14 contains lists of all genes and transcripts, however it only includes 34882 and 160820 unique genes and transcripts respectively not the same as stated in the abstract, please clarify which is correct? And ensure other mentions of those numbers in the manuscript are also correct.

Response: The number of transcript/genes were corrected through the manuscript to reflect the supplemental data (total of 160,82 transcripts and 34,882 genes) (lines, 38-40, 45, 114-115,161-162, 187, 204-213, 284, 288, 366-367,477-480, 487, 491, Table 1, Table 7, Figure 2, and Figure 4)

Comment 3: "The diversity of RNA and miRNA transcript among 50 different bovine tissues and cell types was assessed…" I am still unclear how the number 50 has been reached? Supplemental_file1 includes 51 different names of tissues, however, 5 of those names are actually mammary gland at different time points, so its debatable if they constitute different tissue or cell type?

From a data archiving perspective, the Tissue values should all use valid ontology terms as the tissue field is not meant for distinguishing different time points of sampling, there are other metadata fields for that information.
The use of valid ontology terms will enable others to discover and re-use these data appropriately and is considered good-practice.

Response: lines, 90-91, 439, and 565-566, were revised as they caused ambiguity. In addition, there are 50 tissue, developmental stages, and cell types listed for RNA and miRNA datasets (combined) in the most recent version of submitted Supplemental_file1.tsv file.

Comment 4: The section on trait similarity is perplexing me (and this maybe my lack of experience in this area). Many of the traits mentioned in the network are related to phenotypic measurements, e.g. sperm volume. So, does that mean you have captured many phenotypic values for all the sampled animals? If so, where are those data?

The most recent version of submitted Supplemental_file1.tsv file listed 50 different tissue, developmental stage, and cell lines for RNA and miRNA datasets (combined).

Response: Line 801, Publicly available bovine QTLs were retrieved from Animal QTLdb. In addition, the limitation of this approach has been discussed on lines 557-561.

Comment 5: Where the bioinformatics analysis steps are mentioned; "The overview of the bioinformatics analysis steps is presented in Supplemental file 2: Fig. S39." The authors should include reference to the annotated script file provided to GigaDB.

Response: The GitHub directory included the bioinformatics work-follow and custom scripts, was added to Supplemental file 2: Fig. S39 legend.

Comment 6: The statement "…outlier samples were expressed and removed from downstream analysis." requires evidence. All sequence data generated must be submitted to the archives and cited by accession number, especially where you have removed it from further analysis as an outlier. If you do not provide those data, you are open to accusations of cherry-picking your data.

Response: Unfortunately, we do not have access to these data samples anymore.

Comment 7: The description of the supplemental file 5 in the manuscript differs from the content, please check all supplemental files contain the expected data and are correctly described in the manuscript.

Response: We are not sure what file you were referring to because everything in our perspective looks correct and the most recent version of "Supplemental file 5" (submitted to GigaDB on Jul 18, 2023) includes gene/transcript quantification.

Comment 8: The addition of supplemental_file23.docx has helped clarify some aspects, but it has also drawn attention to some (possibly) missing data;

- The section sub headed "Cell sample collections" describes how some cells were grown, however the main manuscript does not describe these results clearly and I am unable to determine what analysis was actually done with those cells? Were they sequenced? If so, which BioSample accessions do they relate to?

For better clarity, would it be possible to list the unique Animal IDs within each section, e.g. Adult tissue collection change "Eleven cattle (6 males and 5 females) were slaughtered…" to "Eleven cattle (6 males- M08, M09, M10, M11, M130, M22, M23, and 5 females- F05, F06, F07, F12) were slaughtered…"

As you can see above, by looking at the "Samples_meta-data.tsv" provided and filtering for age 420days* it appears there are actually 7 males and 4 females not 6 and 5 as stated in the MS, please clarify which is correct.

*- why use 420 days in the archive but 4 months in the paper? Try to be consistent.

Response: As indicated in 'supplemental_file23.docx,' the cell types used in this study include adipocytes, pre-adipocytes, and myocytes. They were all sequenced, and their respective ENA Run Accessions were listed in 'Supplemental_file1.tsv' file (adipocytes: ERR9846745, ERR9846746, ERR9846747; pre-adipocytes: ERR9707987, ERR9707989, ERR9708039, ERR9708041, ERR9708042, ERR9846824, ERR9846825, ERR9846826; myocytes: ERR9708029, ERR9708030, ERR9708033, ERR9708034, ERR9708038, ERR9846810, ERR9846811). A revised version of 'Samples_meta-data.tsv,' matching 'Supplemental_file1.tsv' was submitted to GigaDB. The age of Herefords breed animals was corrected to '420 days' throughout the manuscript (Supplemental file 23, line 20). In addition, animal IDs were added to Supplemental File 23 for better clarity (lines 18-19, and 25-26).

Comment 9: "Mammary gland tissue collection. The 14 animals used in this study… Samples were collected from animals at 4 time points: virgin state before pregnancy between 13 and 15 months of age (virgin), mid-pregnant at day 100 of pregnancy, late pregnant ~2 weeks pre-calving, and early lactation ~2 weeks post-calving."
In the supplemental_file1 table, when I filter for tissue= mammary gland (virgin), mammary gland (late pregnant), mammary gland (early lactating), or mammary gland (mid pregnant); I can only find 10 different Animal IDs; mam-01, mam-02, mam-03, mam-09, mam-10, mam-11, mam-13, mam-14, mam-15, mam-16. Where are the data for the other 4 animals? It appears maybe there is a 5th mammary tissue "mammary gland (adult)" that may account for the other 4 samples, which means the manuscript statement of 4 time points is incorrect.

Response: The number of collected time points for mammary-gland samples was corrected to 5 (Supplemental file 23: lines 32-35). In addition, the age of animals related to the "mammary gland (adult)" were corrected in the revised 'Samples_meta-data.tsv', and 'Supplemental_file1.tsv' files. We also updated these samples metadata at ArrayExpress database (E-MTAB-11699) to reflect this revision.

Comment 10: "RNA-seq library construction. Tissue samples (Supplemental file 1) were collected from live" - supplemental_file1 does not contain a list of tissues, it is a table of all different sequence run experiments.

Response: The 'Tissue' column in the 'Supplemental_file1.tsv' contains the list of tissues for each dataset used in the study.

Comment 11: The section titled "Sequencing the transcriptomes of seven bovine tissues by using the PacBio Iso-Seq and Illumina RNA-Seq technologies" it is unclear to me why it starts by stating previously published data were used and then goes on to describe how you extracted RNA. Is that a description of how those previously published data were created? Or is it describing additional sequencing carried out by yourselves for this study? If the later, please clarify which NCBI accessions relate to those data.

<u>Response:</u> The section titled "Sequencing the transcriptomes of seven bovine tissues by using the PacBio Iso-Seq and Illumina RNA-Seq technologies" was removed from Supplemental_file23.docx. For clarity, a brief description of the experiment was added to the "PacBio Iso-Seq data analysis" section (lines 631-643).
