# Supplementary material for: Enhanced bovine genome annotation through integration of transcriptomics and epi-transcriptomics datasets facilitates genomic biology
Source: Gigascience. 2024 Apr 16;13:giae019. doi: 10.1093/gigascience/giae019 (PMC11020238; doi:10.1093/gigascience/giae019)
Supplement: giae019_Supplemental_Files [file giae019_supplemental_files.zip › Supplemental_file2 (1).docx]

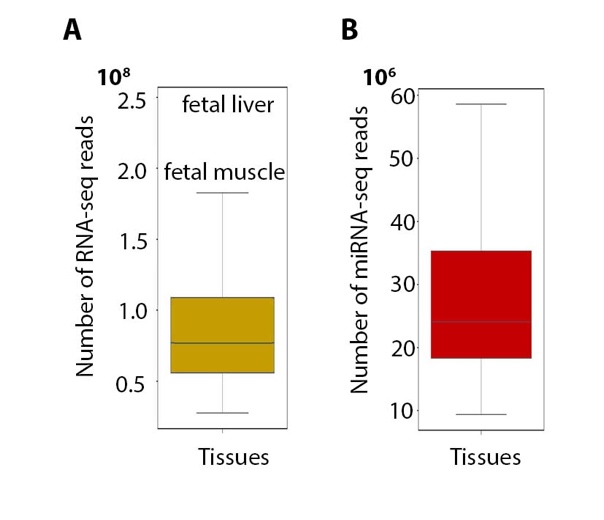


**Figure S1-** Distribution of the number of RNA-seq reads across tissues.


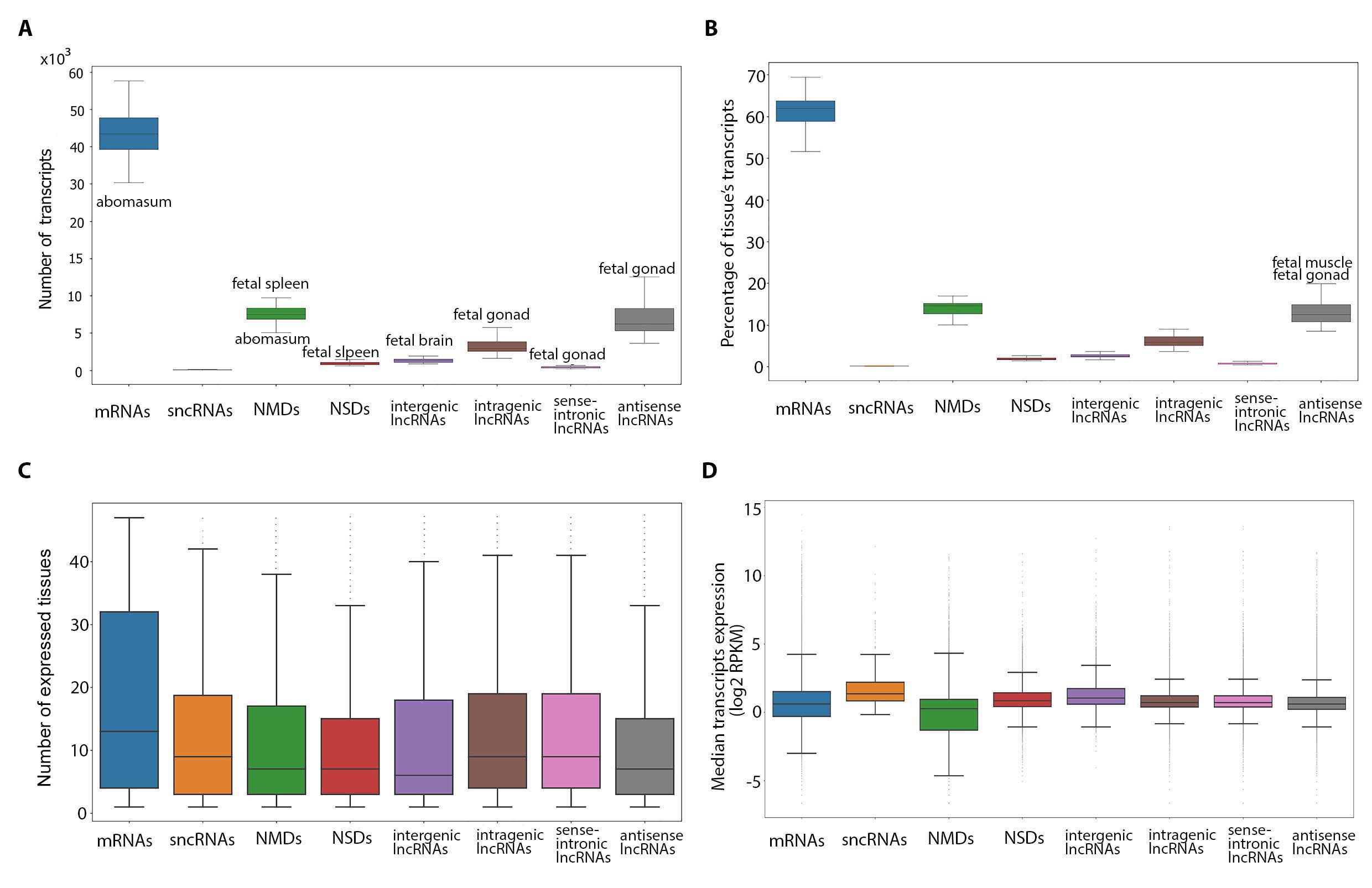


**Figure S2-** (A) Comparison of tissues based on number of transcript biotypes and (B) percentage of transcript biotypes. (C) Comparison of transcript biotypes based on their number of detected tissues and (D) their expression level across detected tissues.


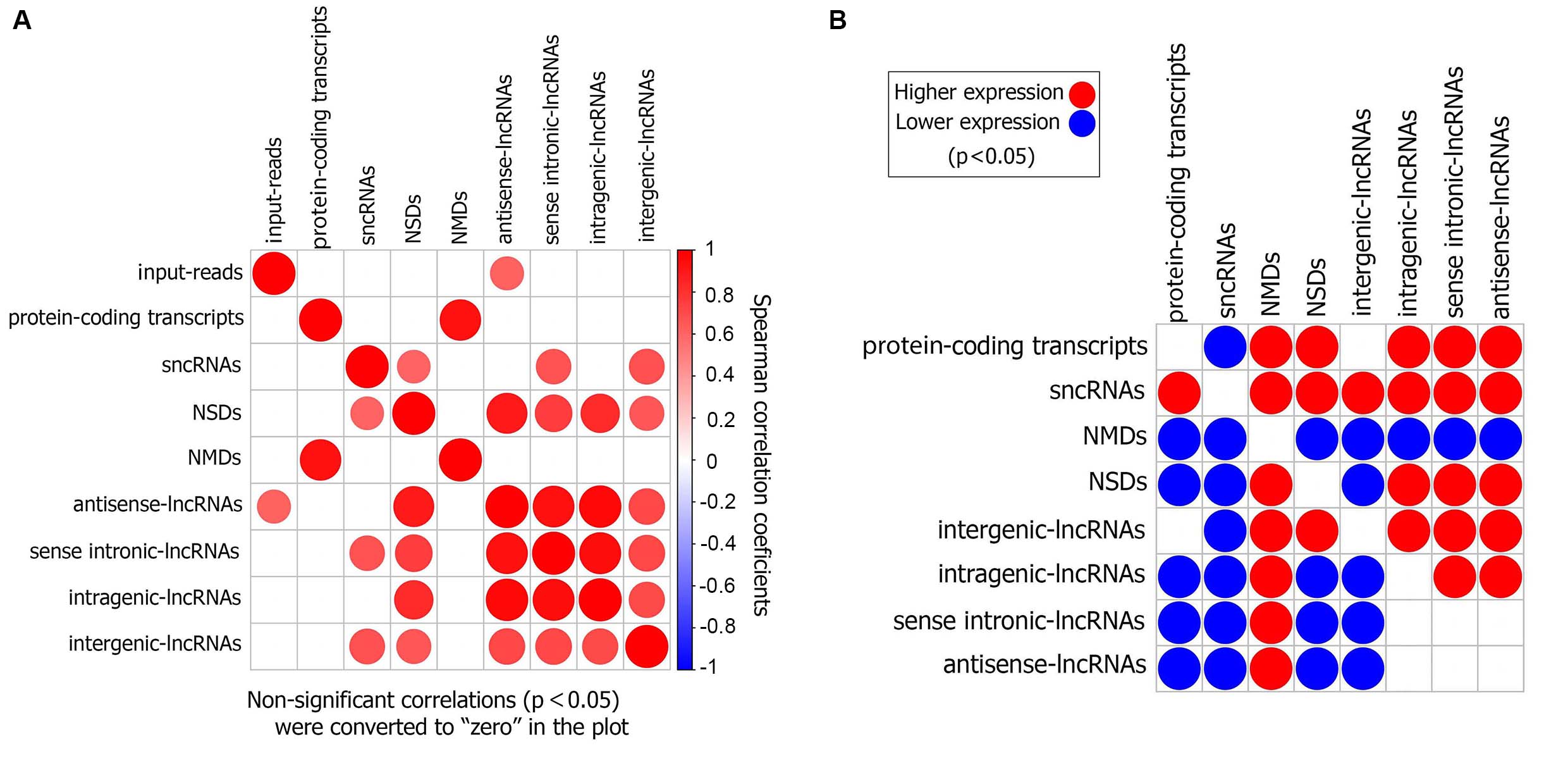


**Figure S3-** (A) relation between number the of input reads and the number of transcript biotypes (B) Comparison of expression level between different transcript biotypes.


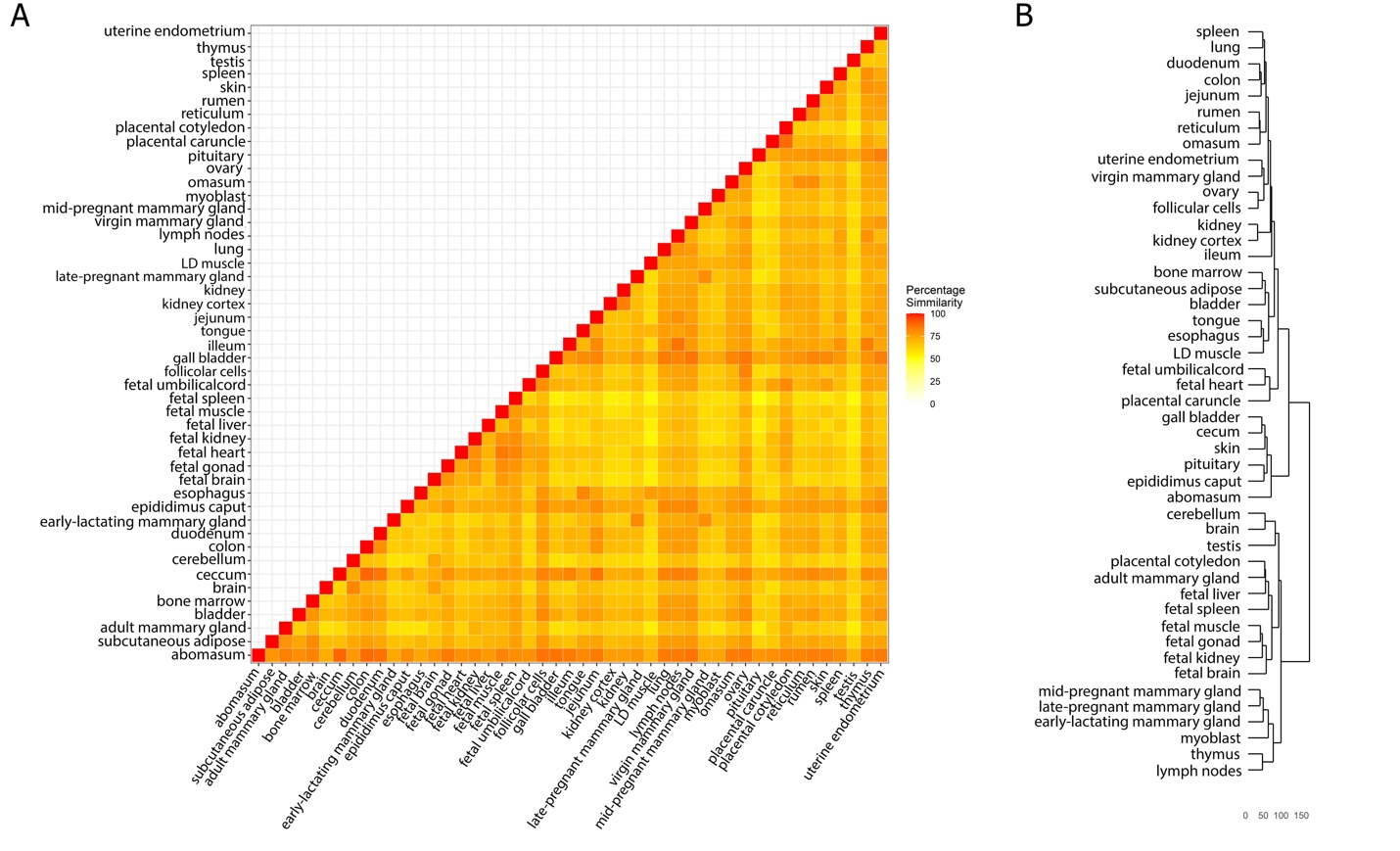


**Figure S4-** Tissue similarities (A) and clustering (B) based on the percentage of protein coding transcripts shared between pairs of tissues.


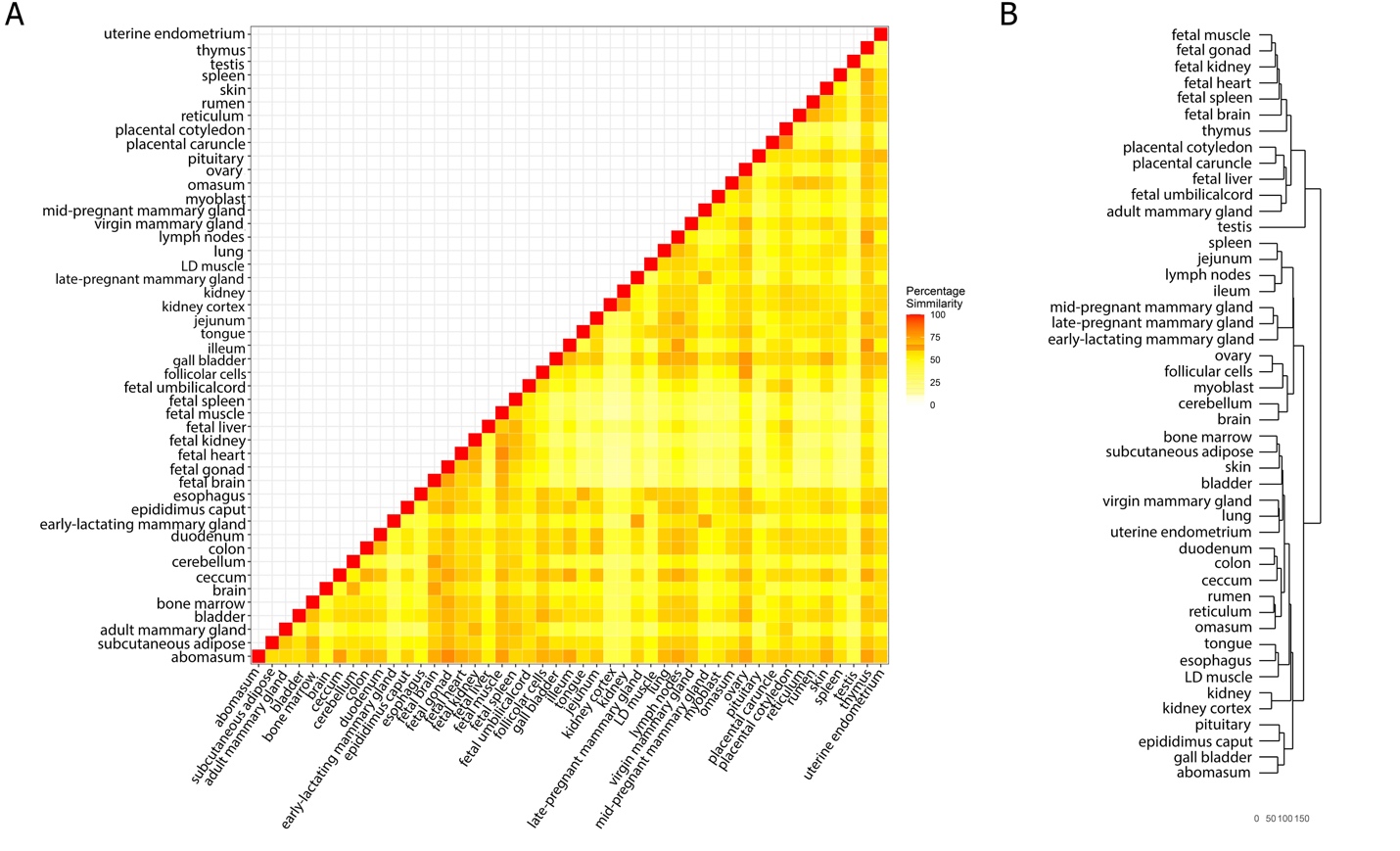


**Figure S5-** Tissue similarities (A) and clustering (B) based on the percentage of non-coding transcripts shared between pairs of tissues.


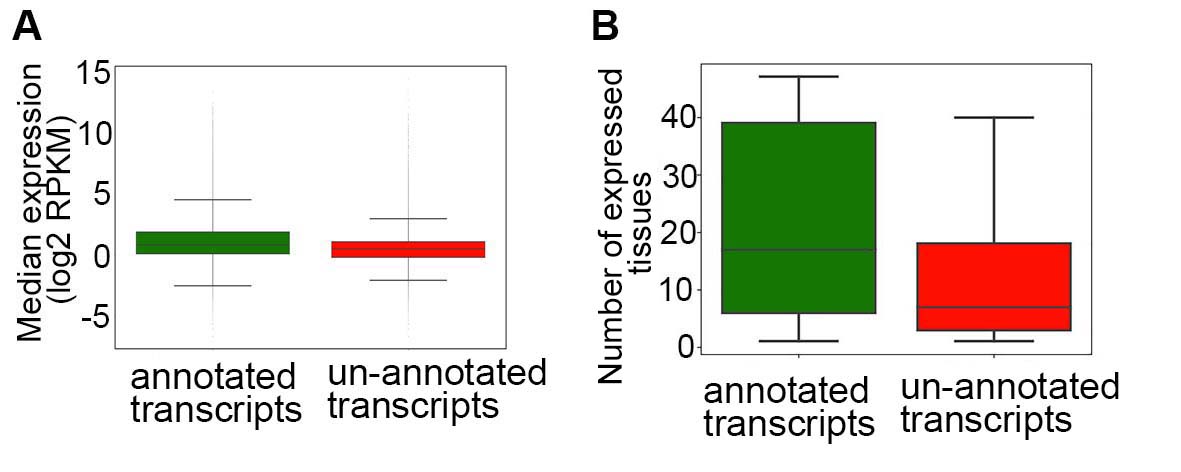


**Figure S6-** Comparison of known and novel transcripts based on their expression (A) and number of detected tissues (B).


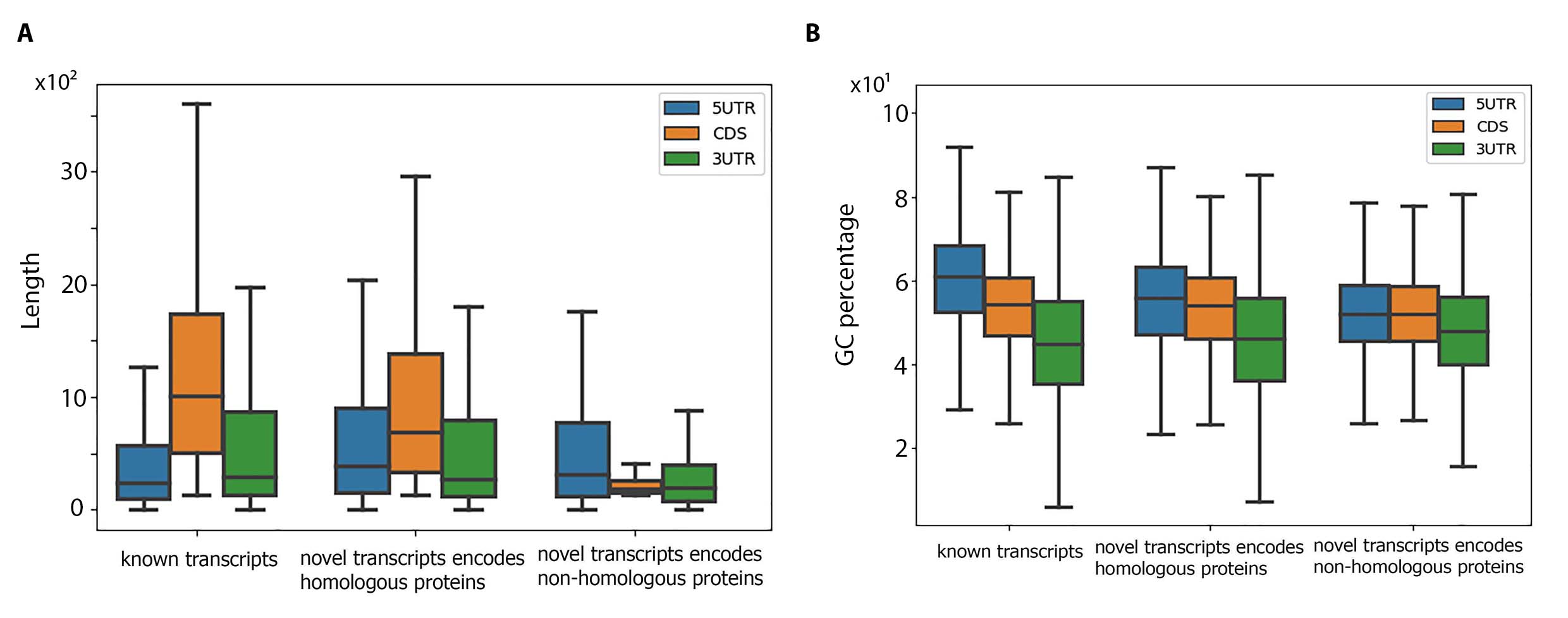


**Figure S7-** Comparison of known and novel protein coding transcripts based on the length (A) and GC content (B) of their 5’UTR, CDS and 3’UTR.


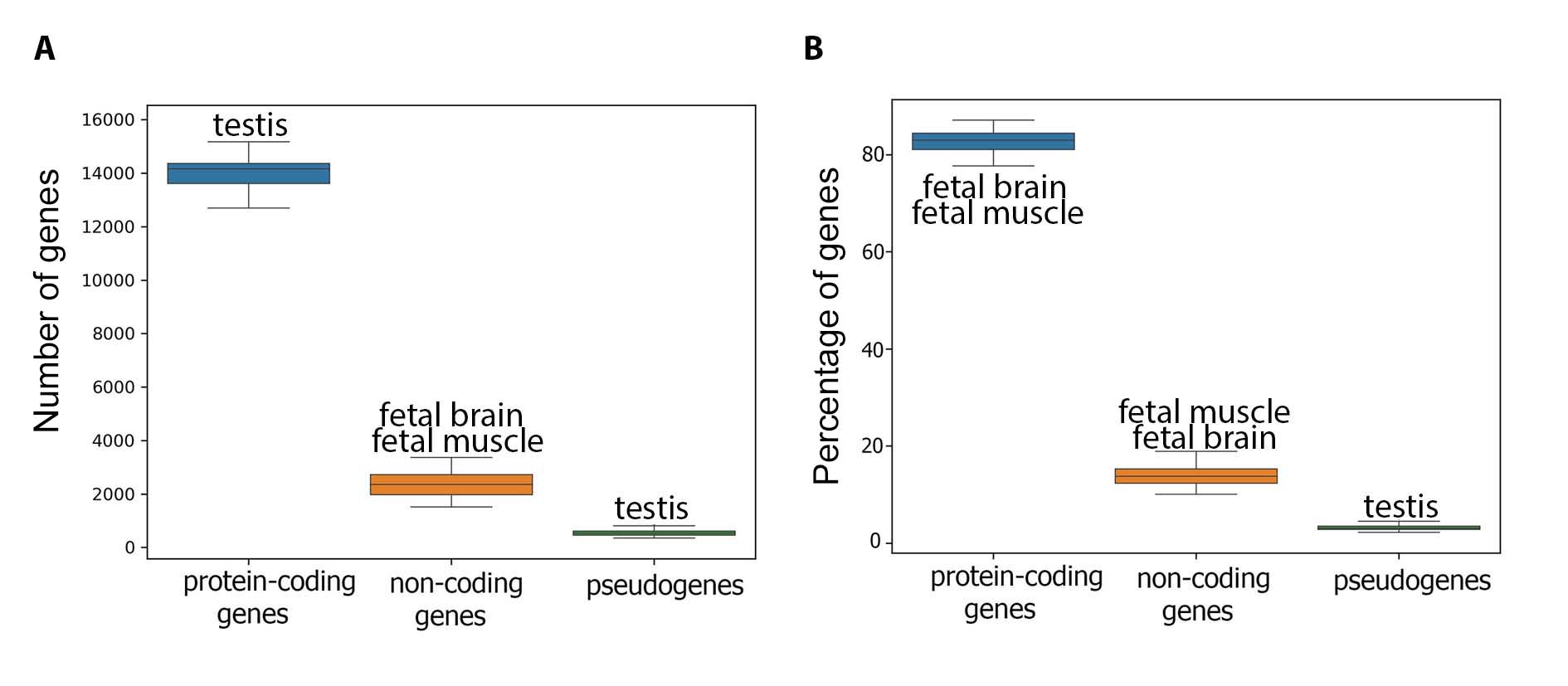


**Figure S8-** (A) Comparison of tissues based on number of gene biotypes and (B) percentage of gene biotypes.


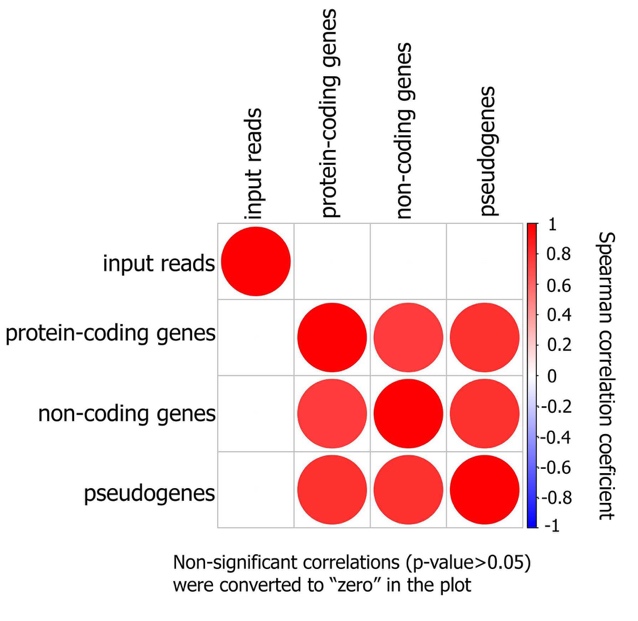


**Figure S9-** Relation between the number of input reads and the number of gene biotypes**.**


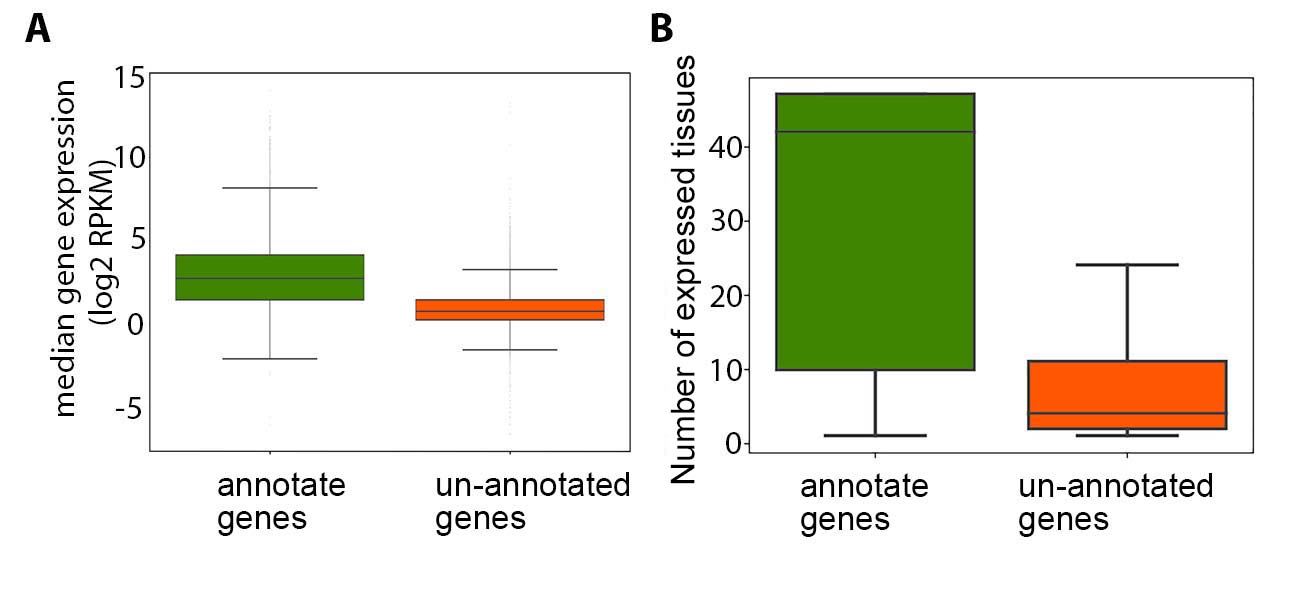


**Figure S10-** Comparison of known and novel genes based on their expression (A) and number of detected tissues (B).


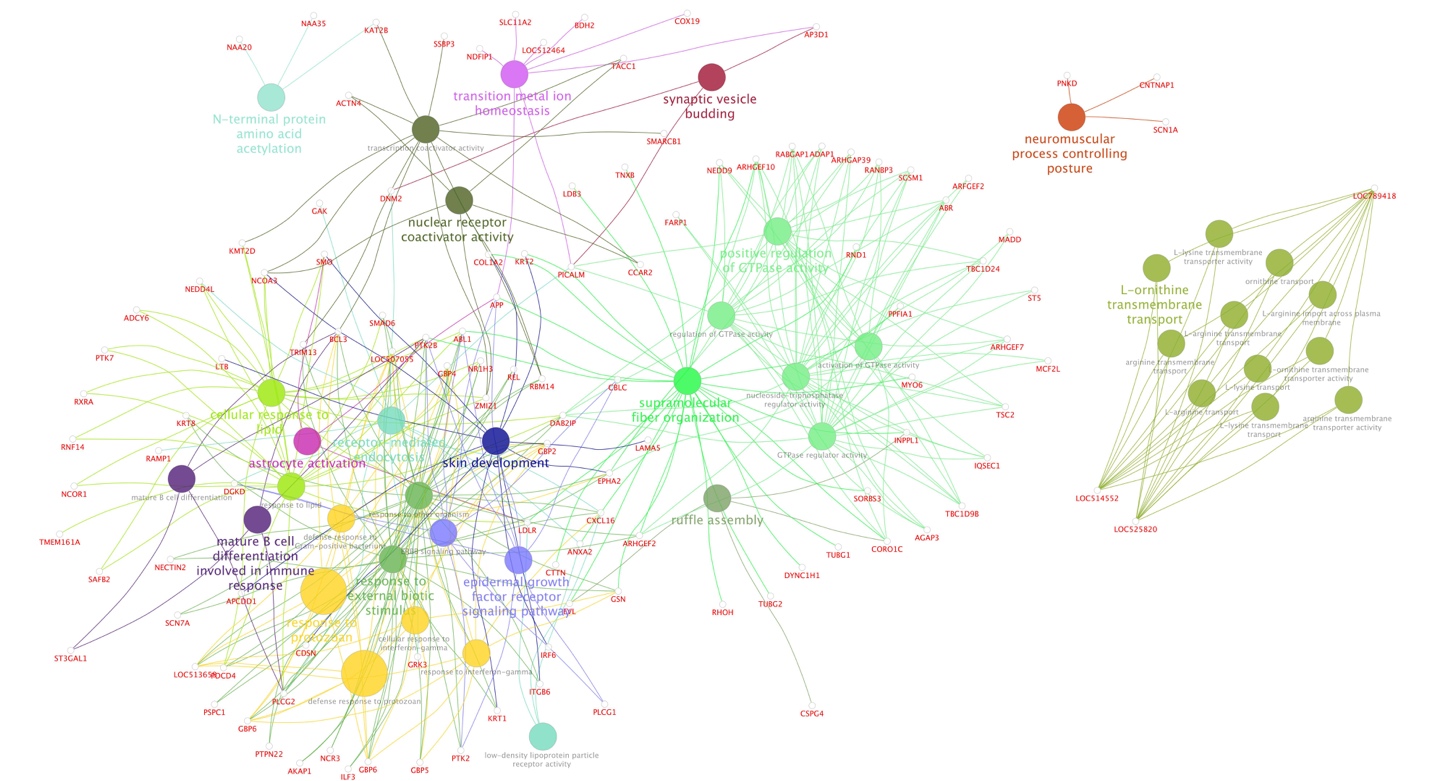


**Figure S11-** Functional enrichment analysis of the top five percent of genes with the highest number of UTRs.


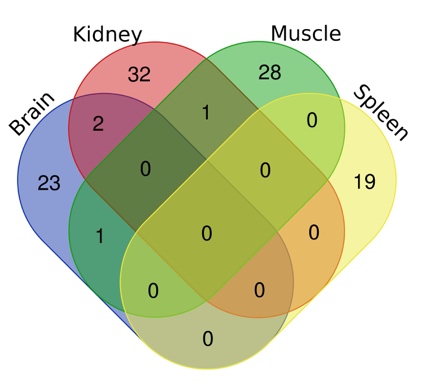


**Figure S12-** Similarity of tissues based on the number of non-coding genes in their fetal samples that switched to protein-coding genes that purely encoded coding transcripts in their adult samples.


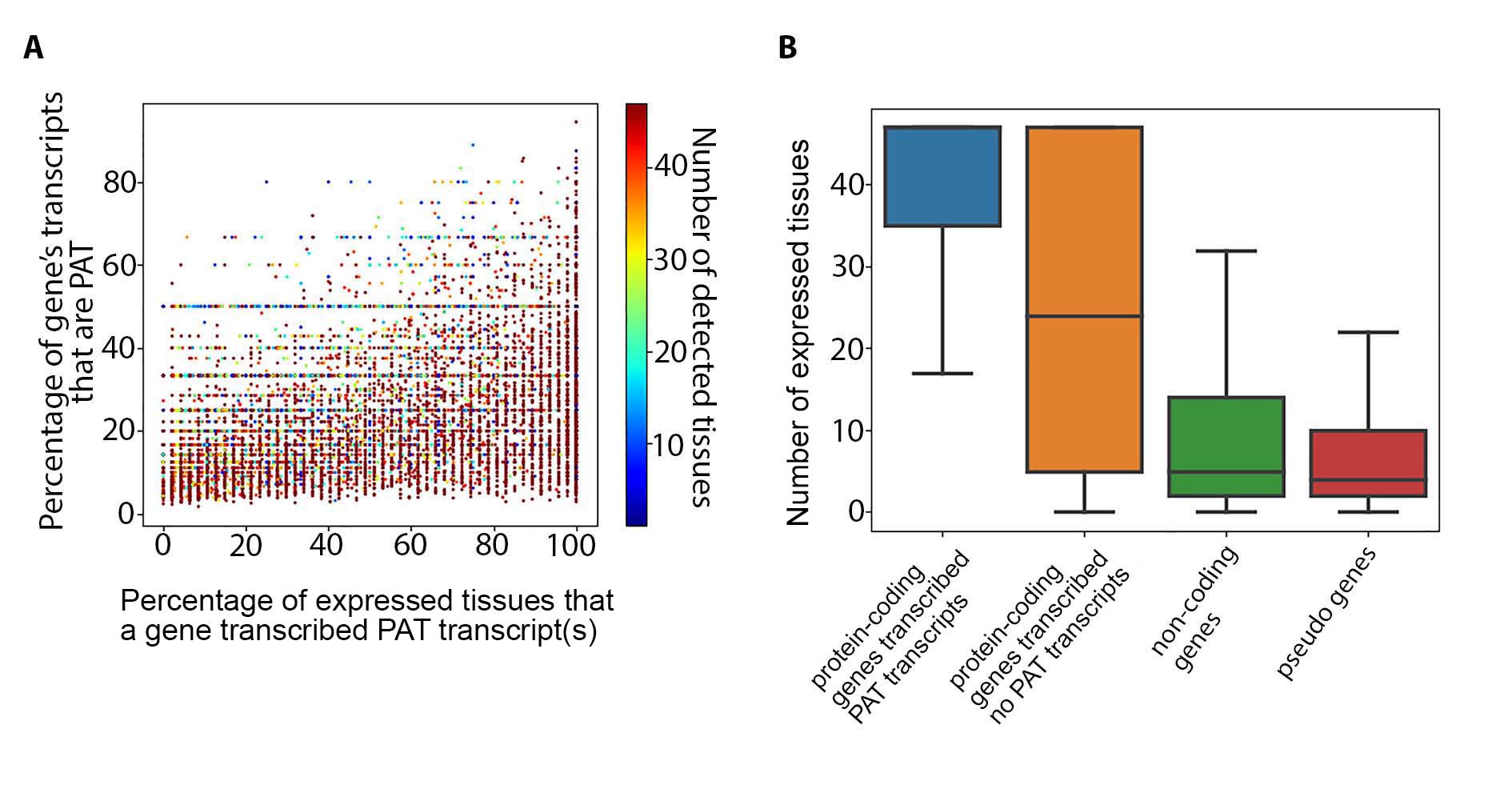


**Figure S13-** (A) Distribution of genes that transcribed PATs based on their number of detected tissues, percentage of a gene’s transcripts that are PATs and percentage of a gene’s detected tissues in which it transcribed PAT transcripts. (B) Comparison of genes transcribing PATs transcripts with other gene biotypes.


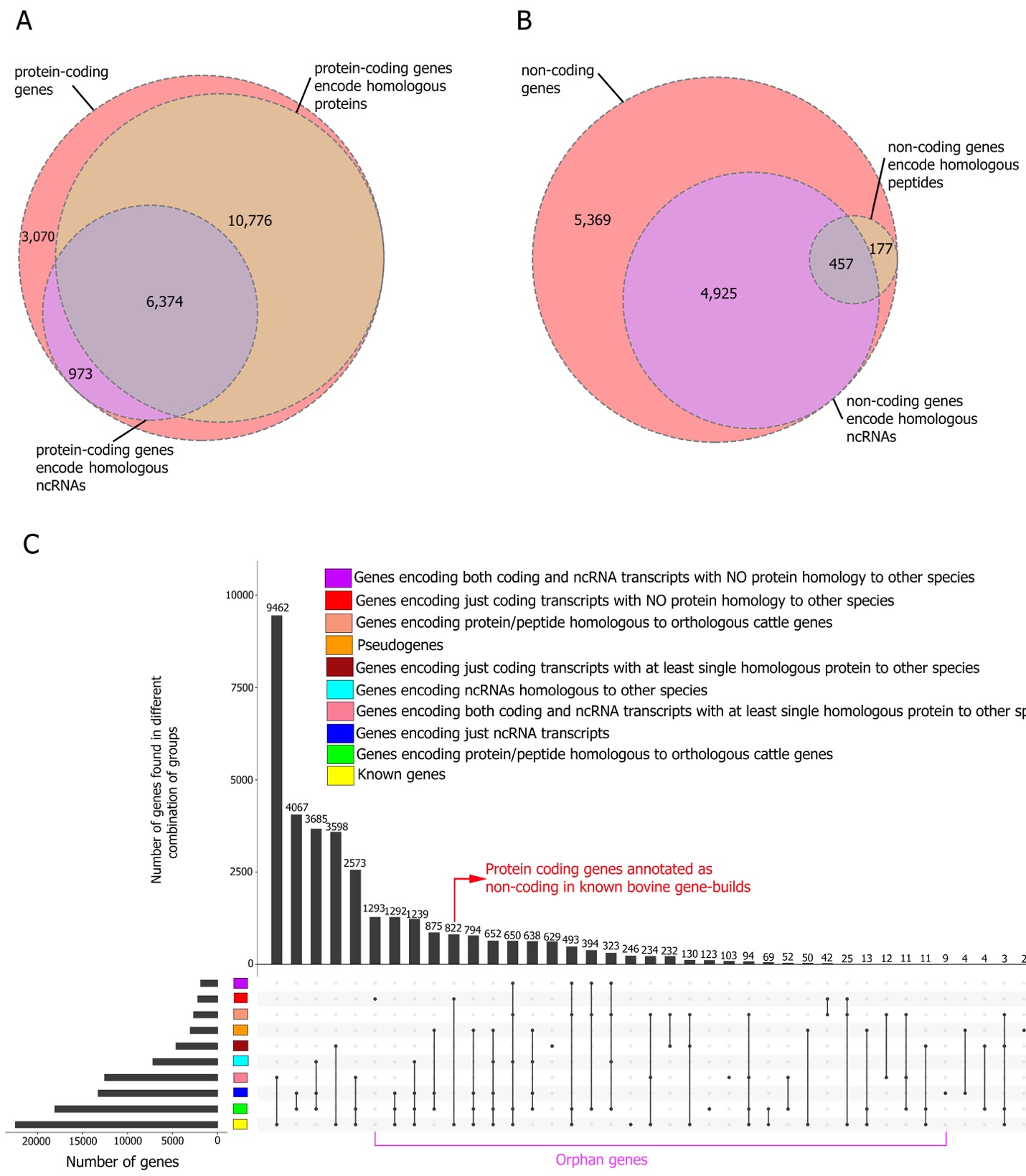


**Figure S14-** (A) Homology analysis of protein-coding genes. (B) Homology analysis of non-coding genes. (C) Detection of orphan genes based on homology classification of cattle-specific protein coding genes and non-coding genes.

**
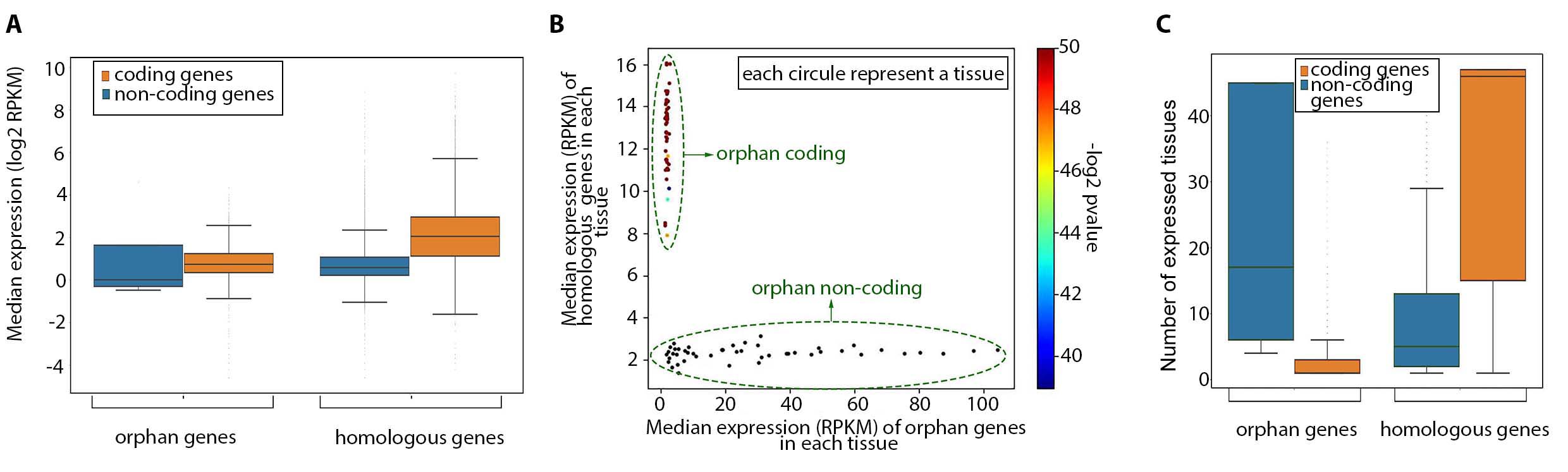
**

**Figure S15-** Comparison of the expression level of homologous and orphan genes across (A) and within (B) their detected tissues. (C) Comparison of homologous and orphan genes based on the number of detected tissues.


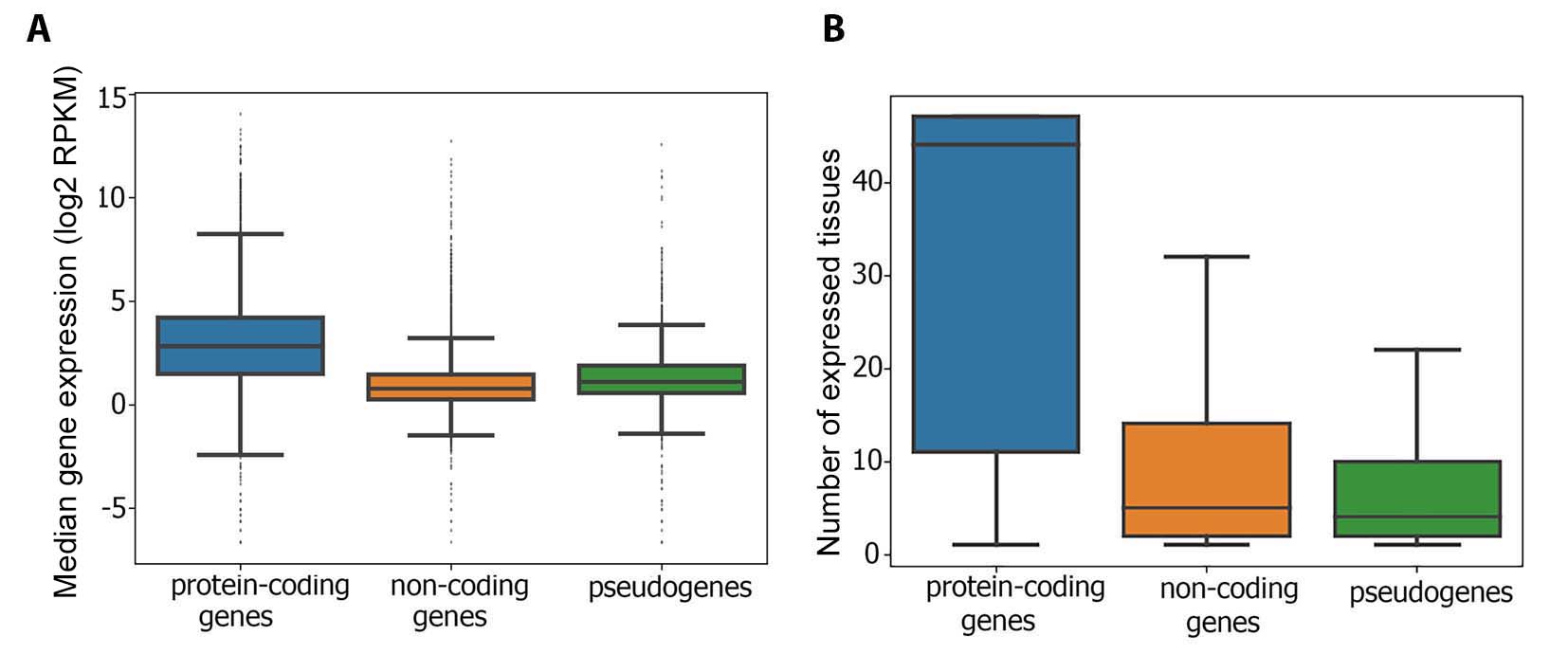


**Figure S16-** Comparison of different gene biotypes based on the expression (A) and the number of detected tissues (B).


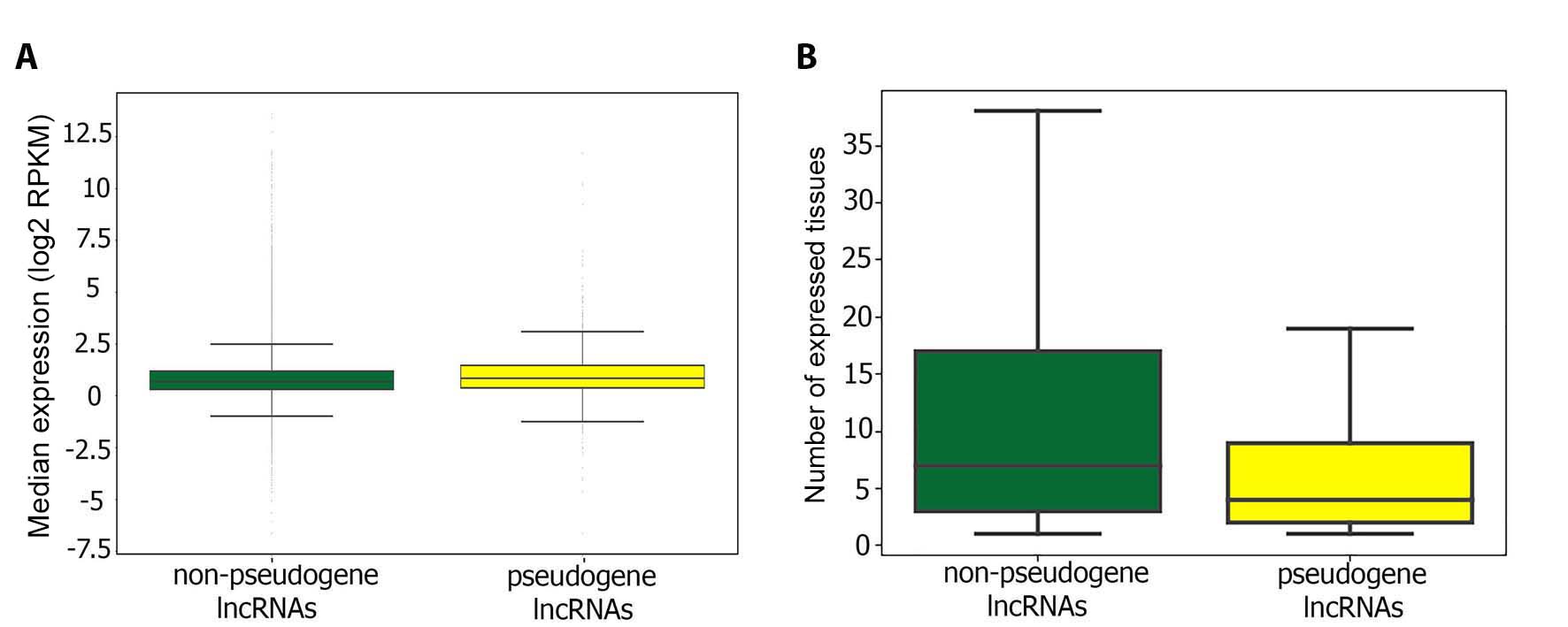


**Figure S17-** Comparison of different pseudogene derived lncRNAs and non-pseudogene derived lncRNAs based on the expression (A) and the number of detected tissues (B)**.**


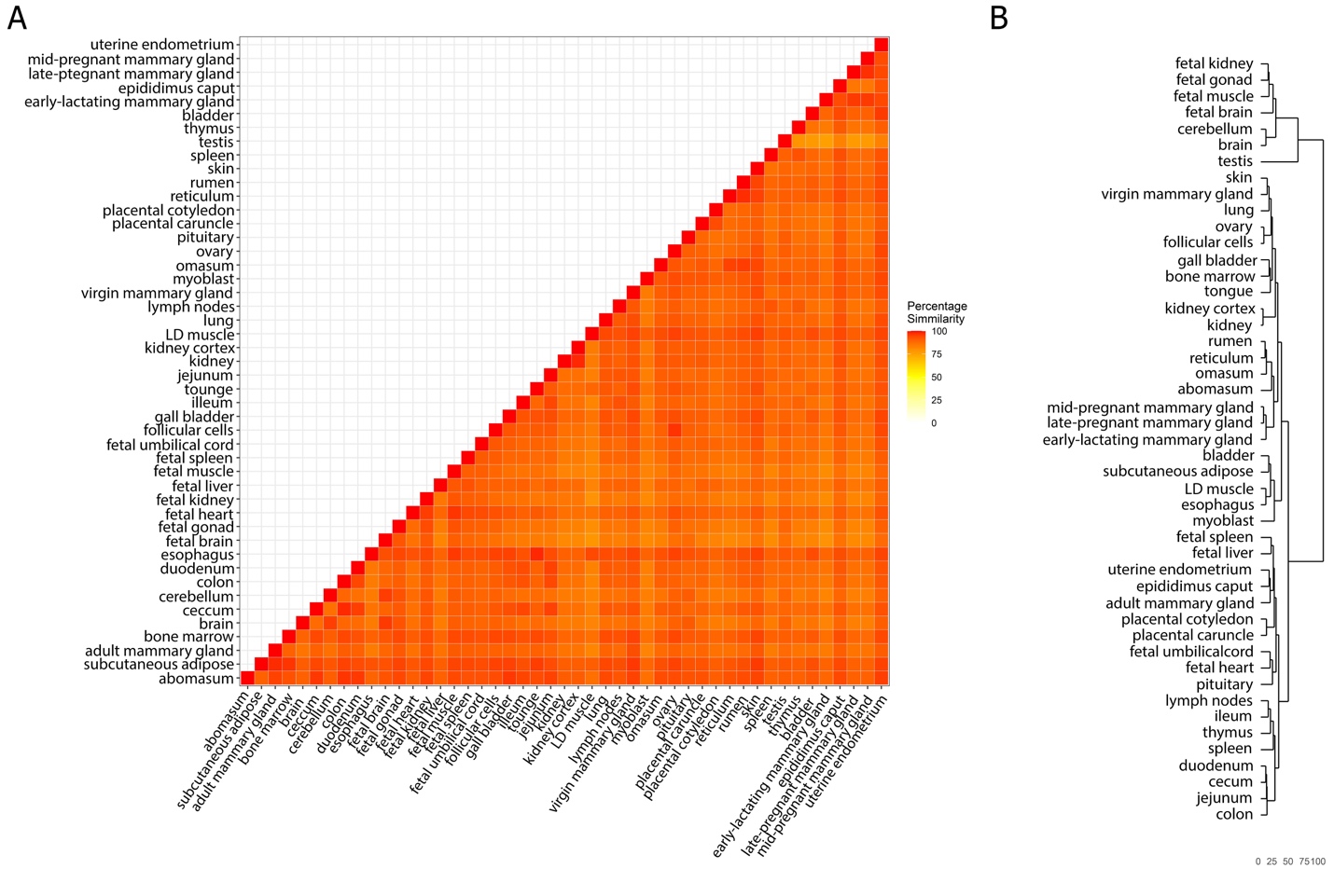


**Figure S18-** Tissue similarities (A) and clustering (B) based on the percentage of protein coding genes shared between pairs of tissues.


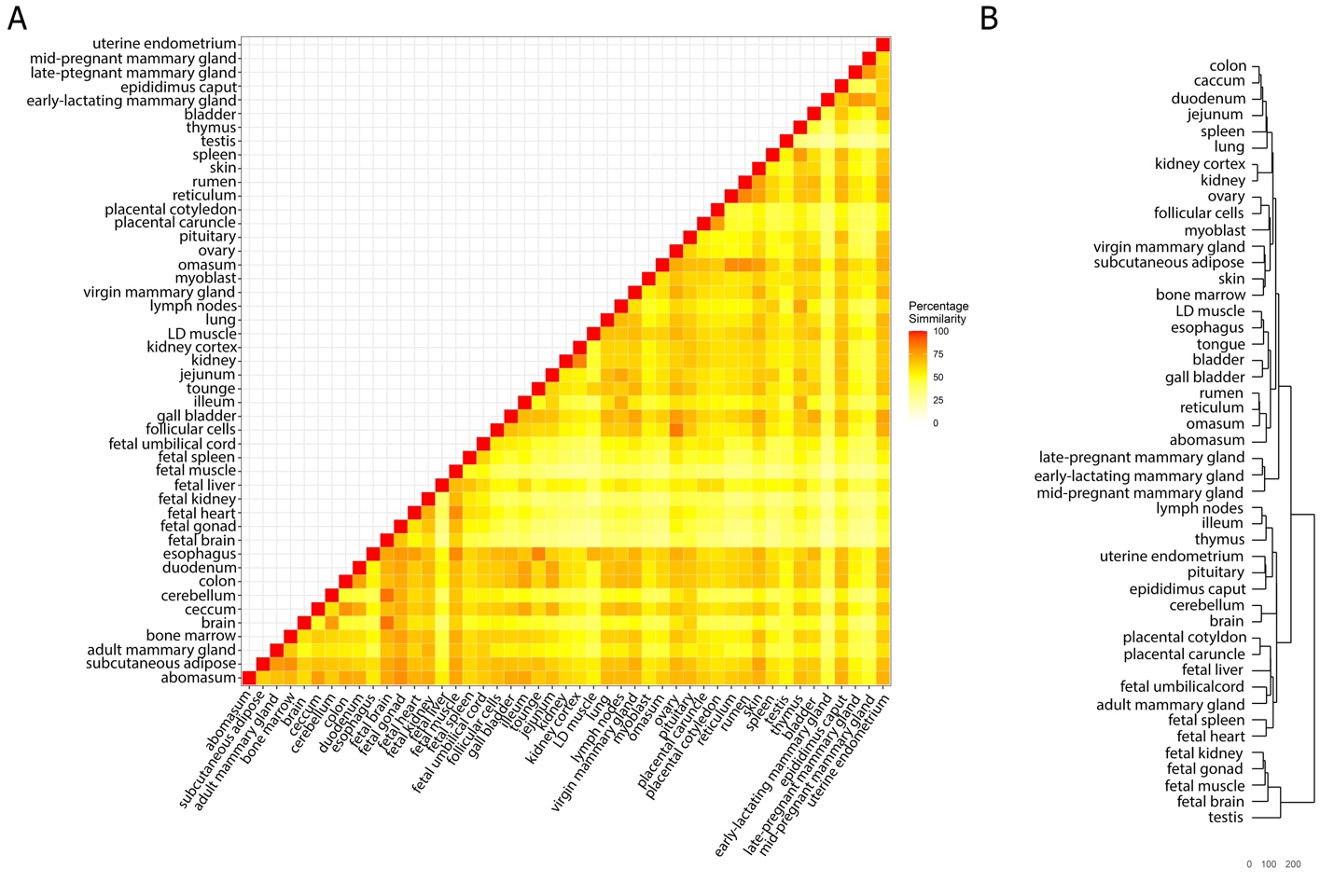


**Figure S19-** Tissue similarities (A) and clustering (B) based on the percentage of non-coding genes shared between pairs of tissues.


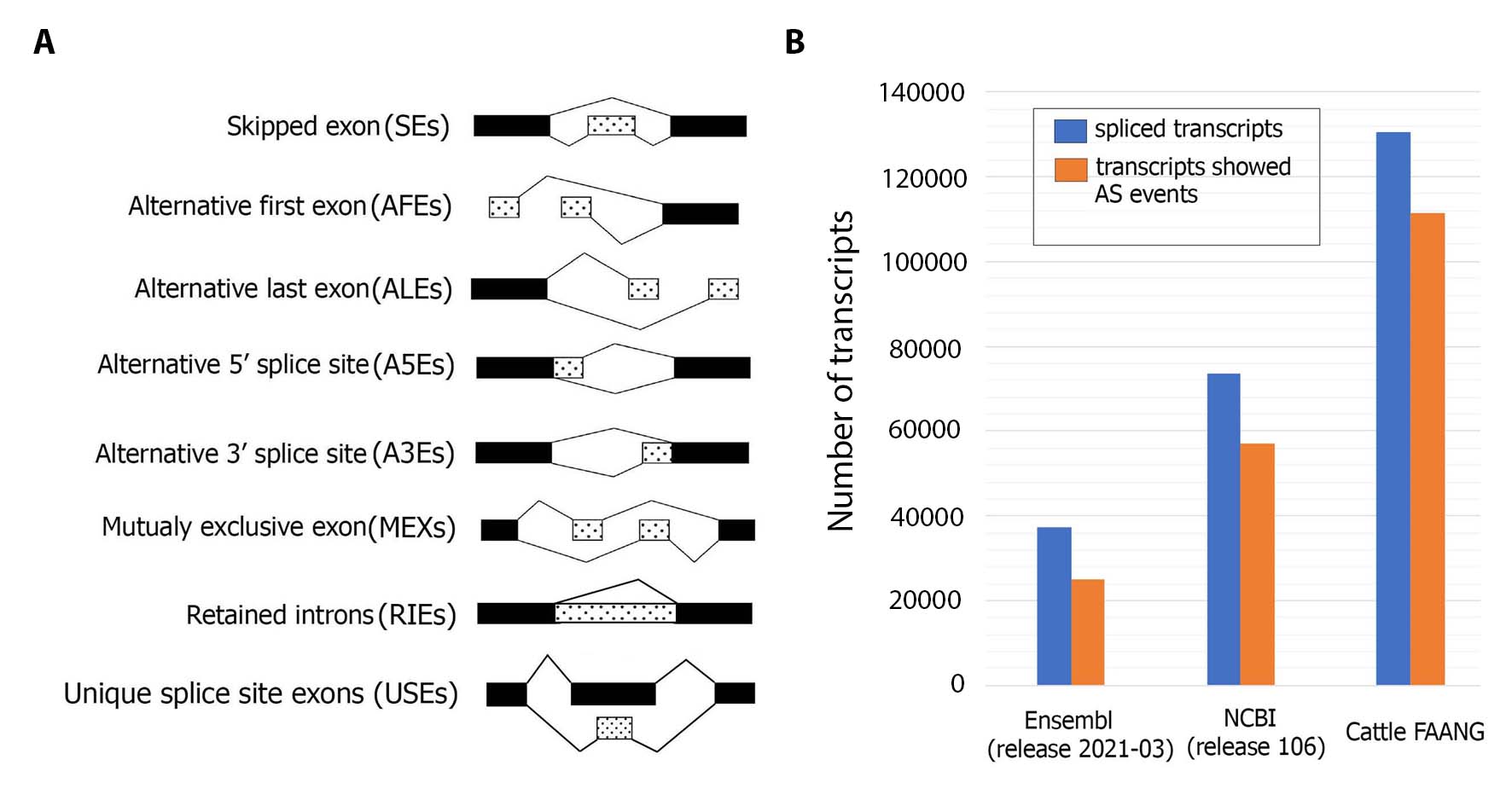


**Figure S20-** (A) Different types of alternative splicing events. (B) Comparison of bovine gene-builds based on the number of transcripts that showed any type of alternative splicing events (AS)**.**


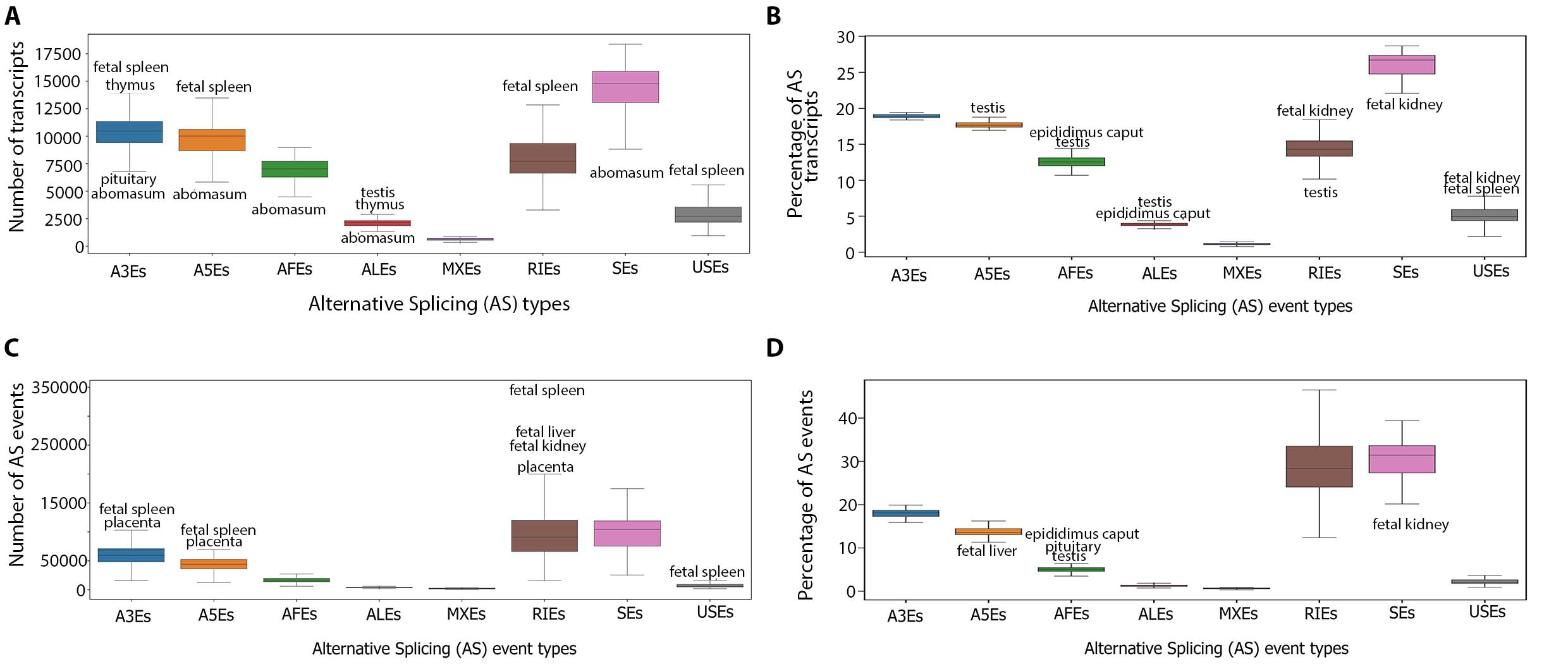


**Figure S21-** Comparison of tissues based on the number and the percentage of transcripts that showed different types of alternative splicing events (A and B, respectively). Comparison of tissues based on the number and the percentage of alternative splicing events (C and D, respectively)**.**


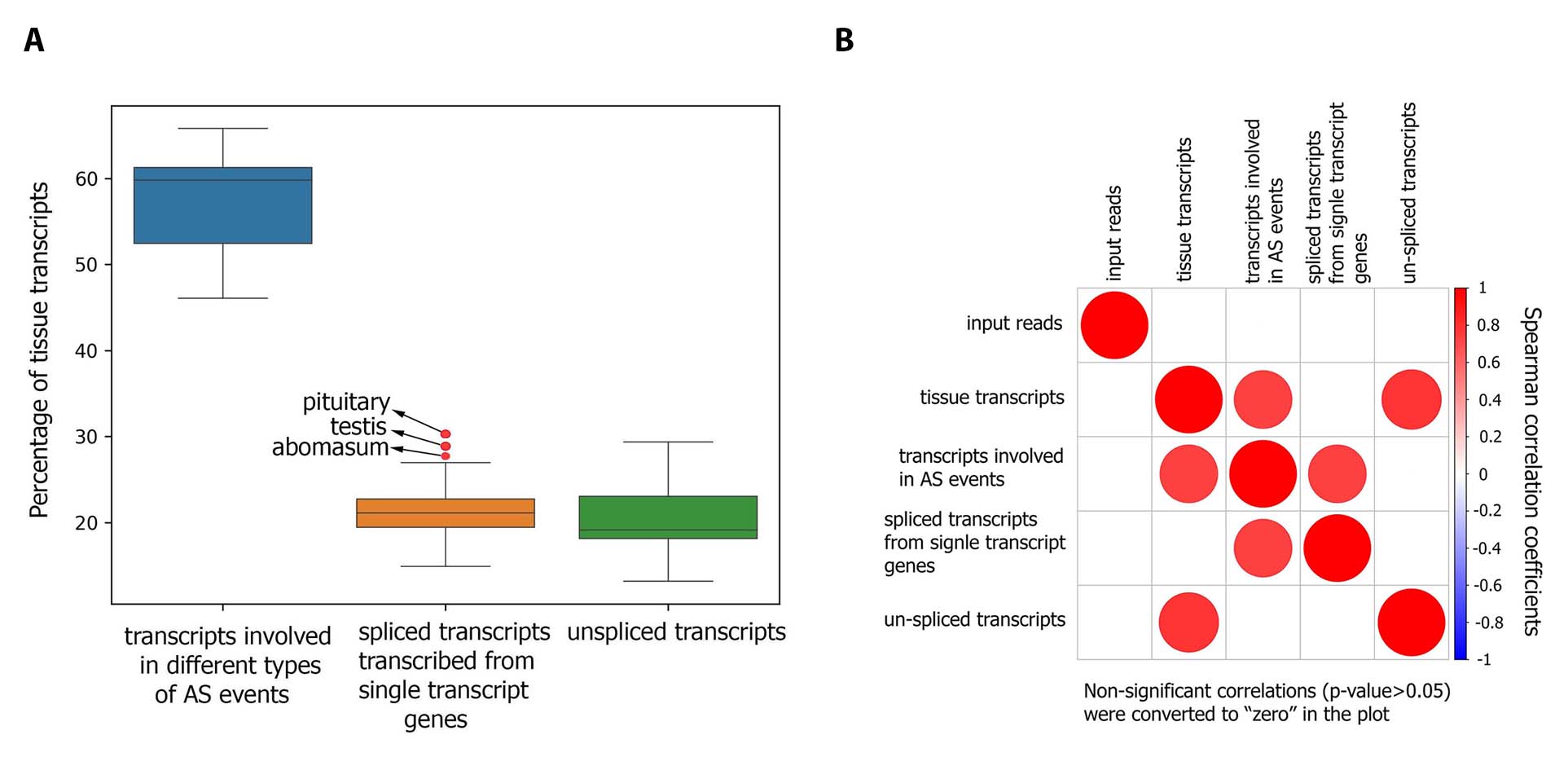


**Figure S22-** (A) Comparison of tissues based on the percentage of transcripts that showed any type of alternative splicing events, spliced transcripts from single-transcript genes, and unspliced transcripts and (B) the relation between the number of input reads and the number of these transcripts across tissues.


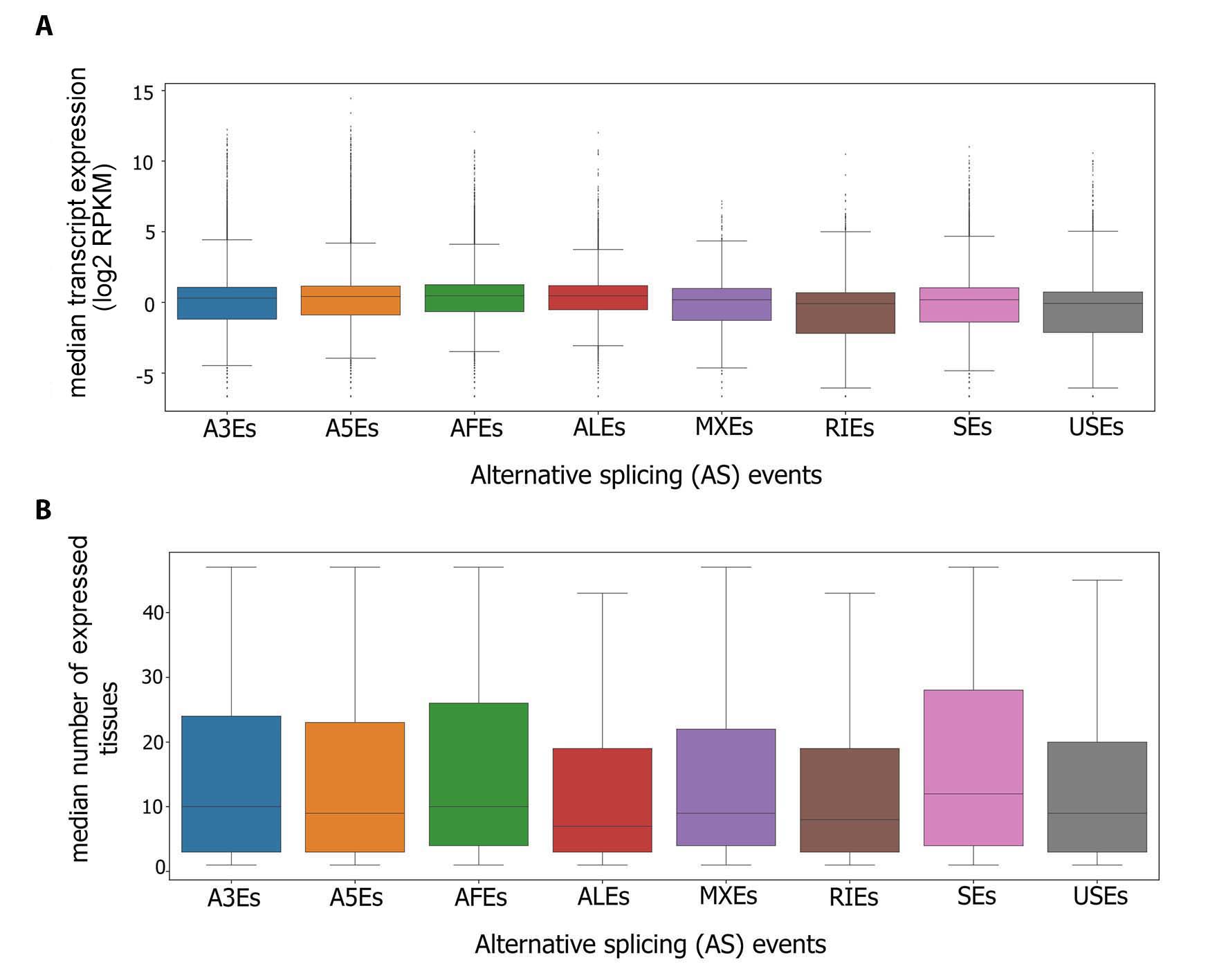


**Figure S23-** Comparison of transcripts that showed different types of alternative splicing events based on (A) the expression level in the detected tissues and (B) the number of detected tissues.


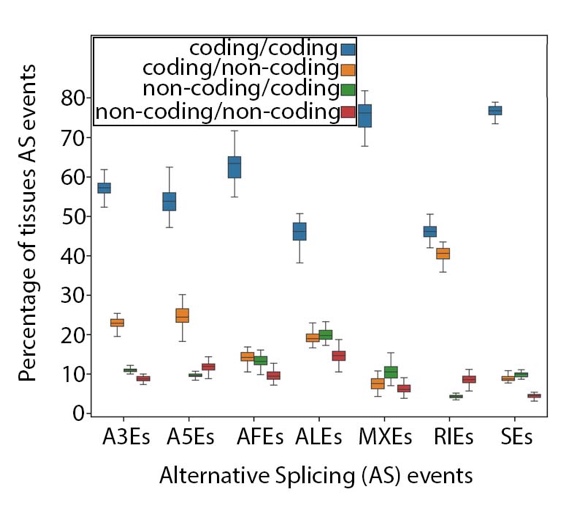


**Figure S24-** Transcript biotype switching due to alternative splicing events**.**


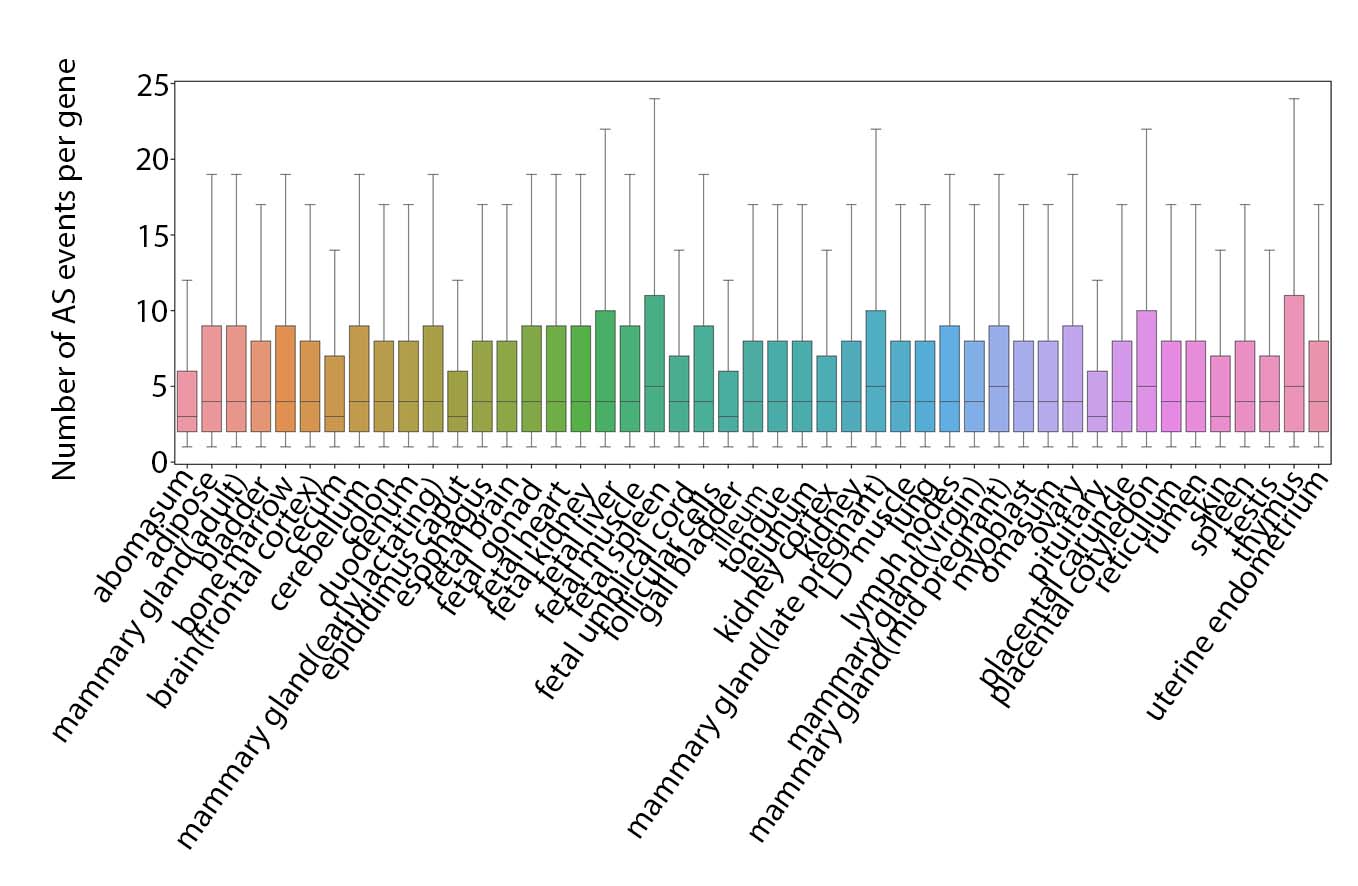


**Figure S25-** Comparison of tissues based on the number of alternative splicing events per alternatively spliced gene**.**


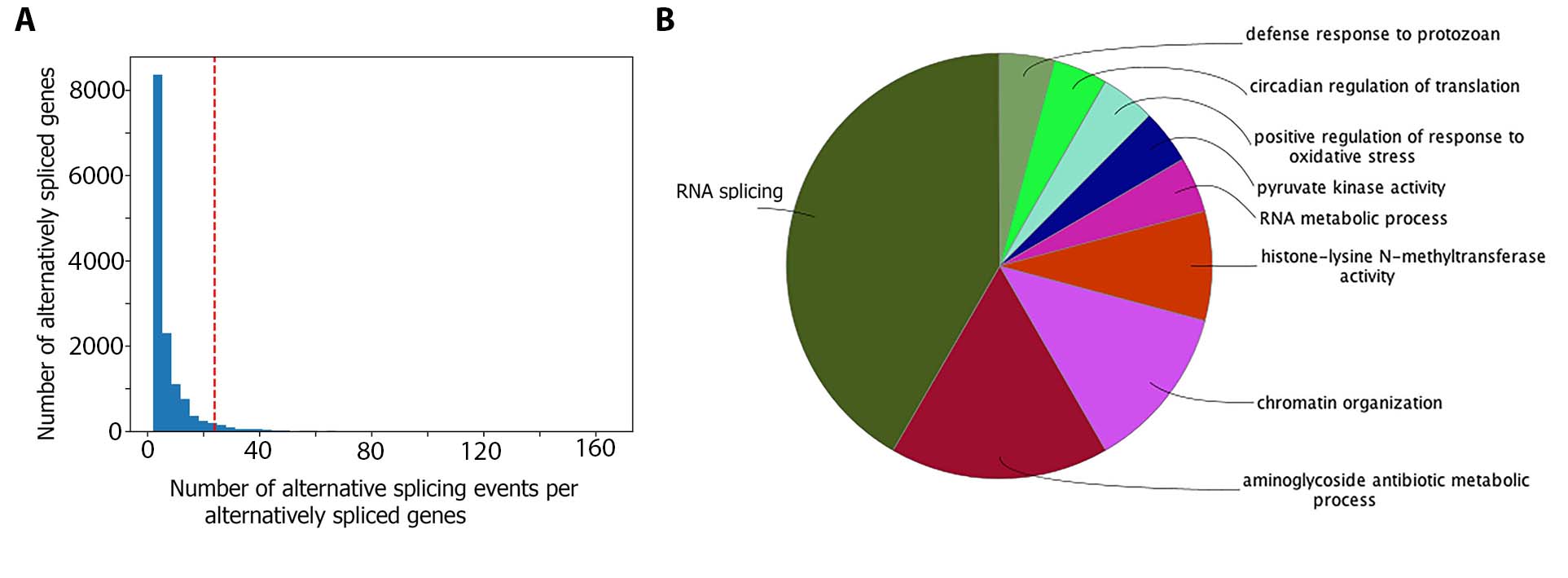


**Figure S26-** (A) Distribution of the number of alternative splicing events per alternatively spliced gene. The 5% quantile is shown using a dashed red line. (B) Functional enrichment analysis of the top five percent of genes with the highest number of alternative splicing events.


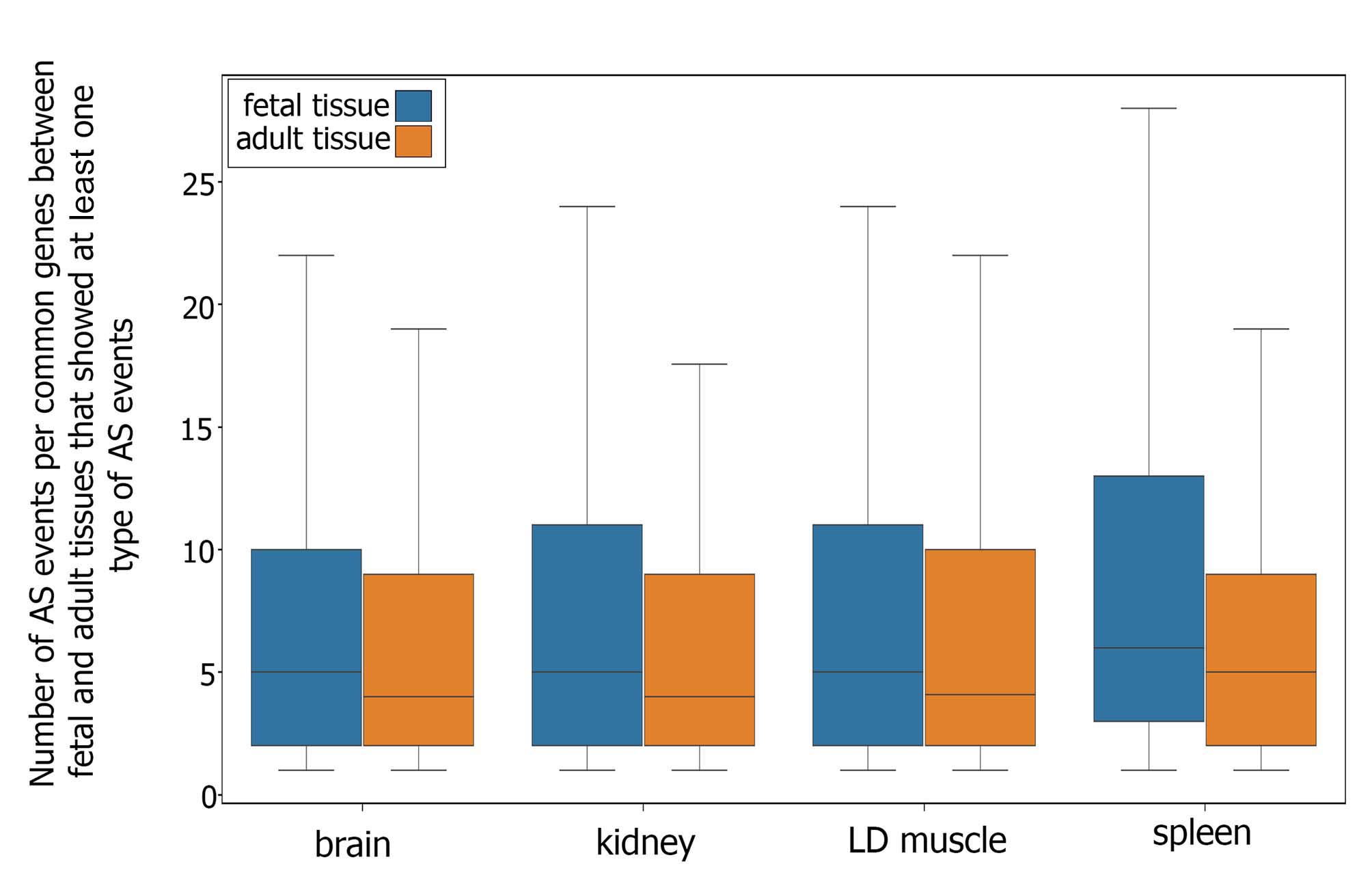


**Figure S27-** Comparison of alternative splicing rate between adult and fetal tissues.


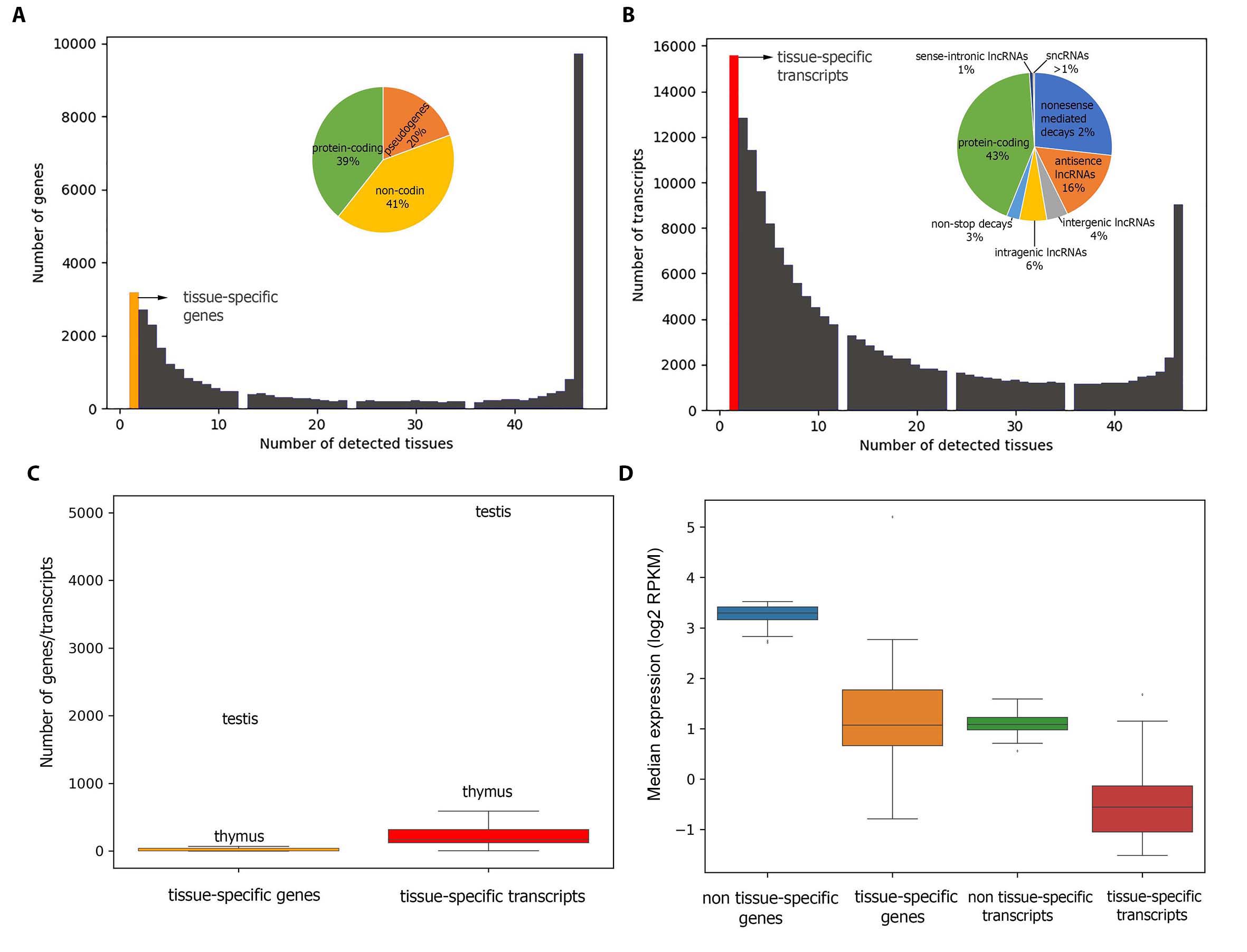


**Figure S28-** (A) Distribution of genes number of detected tissues for genes. Tissue-specific gene biotypes are shown in the pie chart. (B) Distribution of number of detected tissues for transcripts. Tissue-specific transcript biotypes are shown in the pie chart. (C) Comparison of tissues based on the number of tissue-specific genes and transcripts. (D) Comparison of the expression level of tissue-specific genes and transcripts with their non-tissue-specific counterparts.


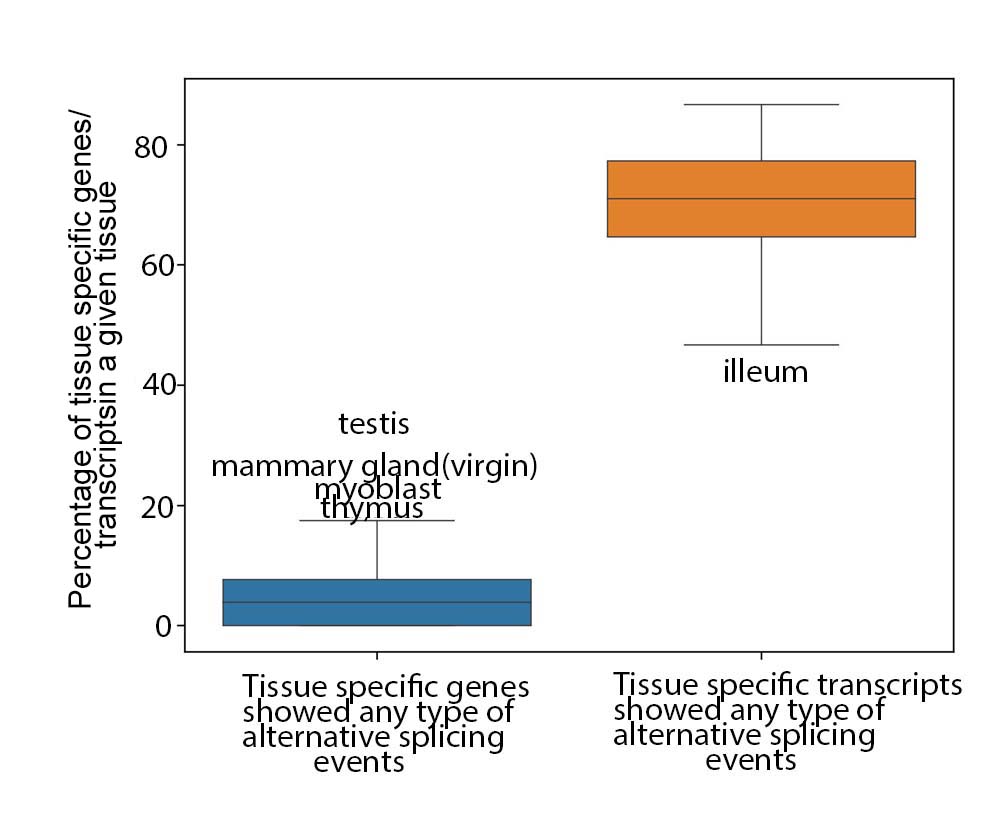


**Figure S29-** Relation between tissue specificity and alternative splicing events**.**


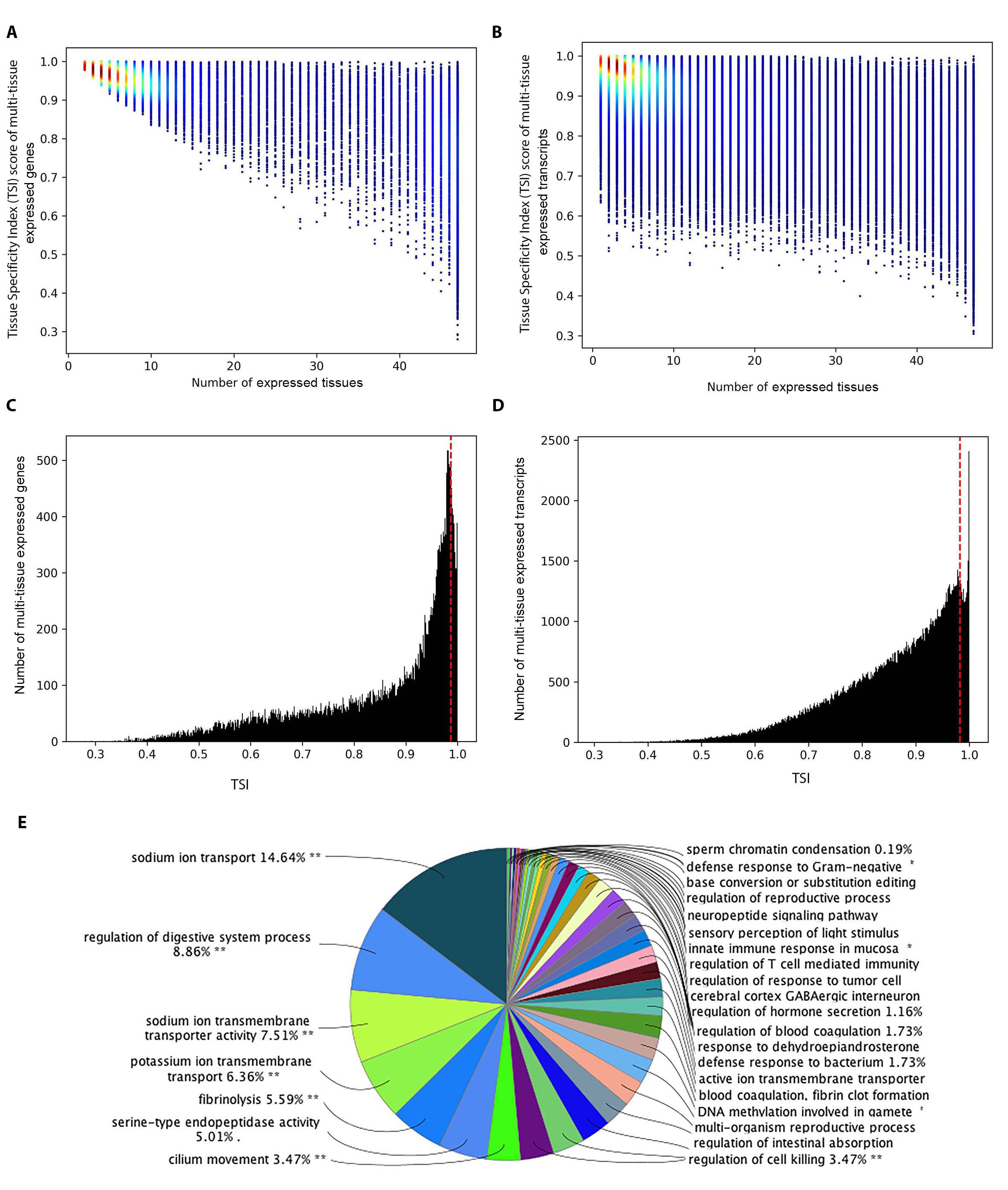


**Figure S30-** Relation between tissue specificity index and the number of tissues in multi-tissue detected genes (A) and transcripts (B). Distribution of tissue specificity indexes in multi-tissue detected genes (C) and transcripts (D). The 5% quantile is shown with dashed red lines. (E) Functional enrichment analysis of the top five percent of multi-tissue detected genes with the highest tissue specificity indexes.


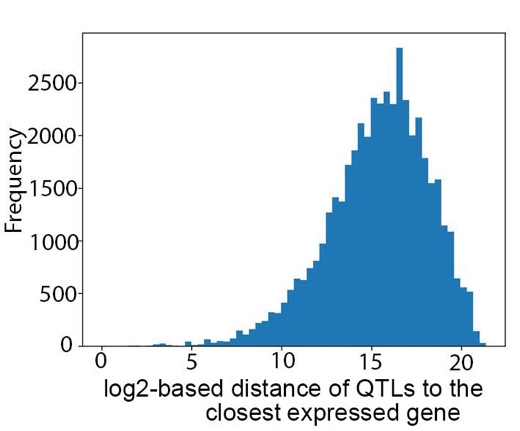


**Figure S31-** Distribution of QTLs located outside gene borders to the closest gene**.**


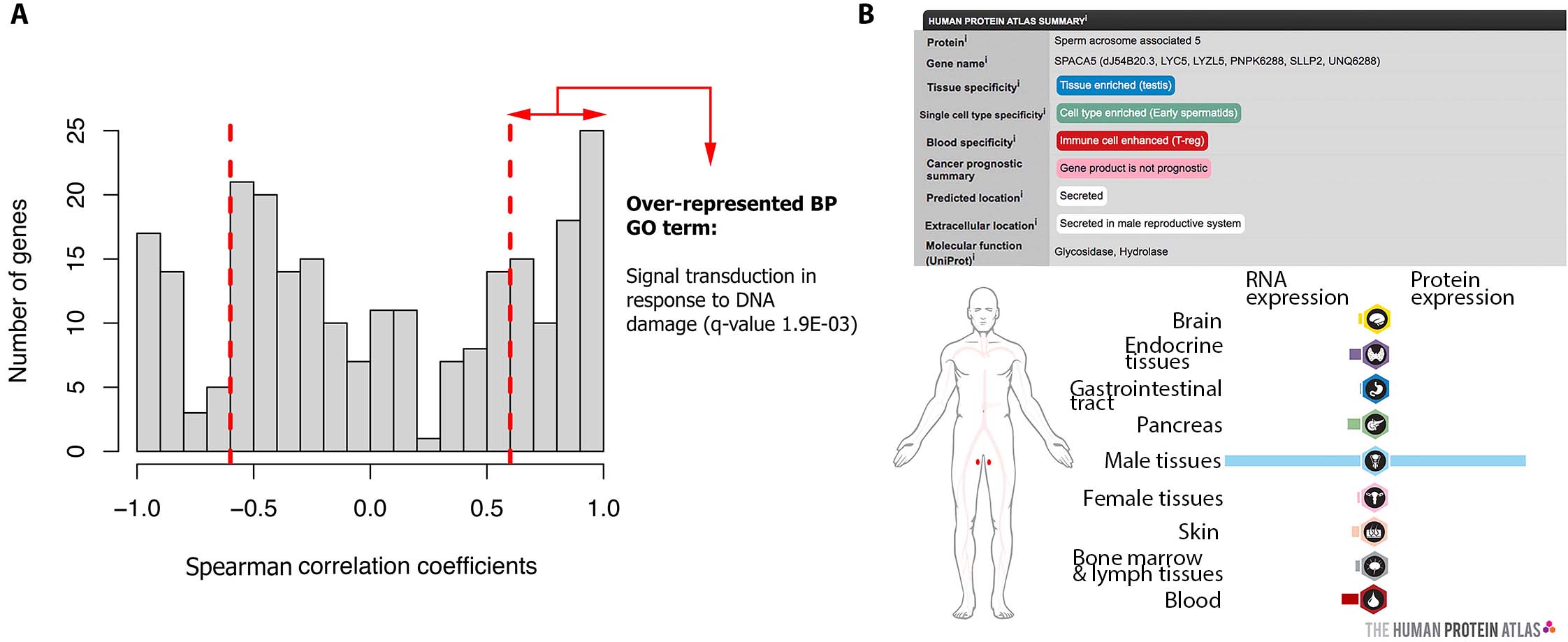


**Figure S32-** (A) Distribution of correlation coefficients between SPACA5 gene expression and pituitary genes closest to “percentage of normal sperm” QTLs. Dashed lines show the minimum significant positive and negative correlation (p-value <0.05). (B) Expression atlas of SPACA5 gene in human tissues from The Human Protein Atlas (Uhlén et al. 2015).


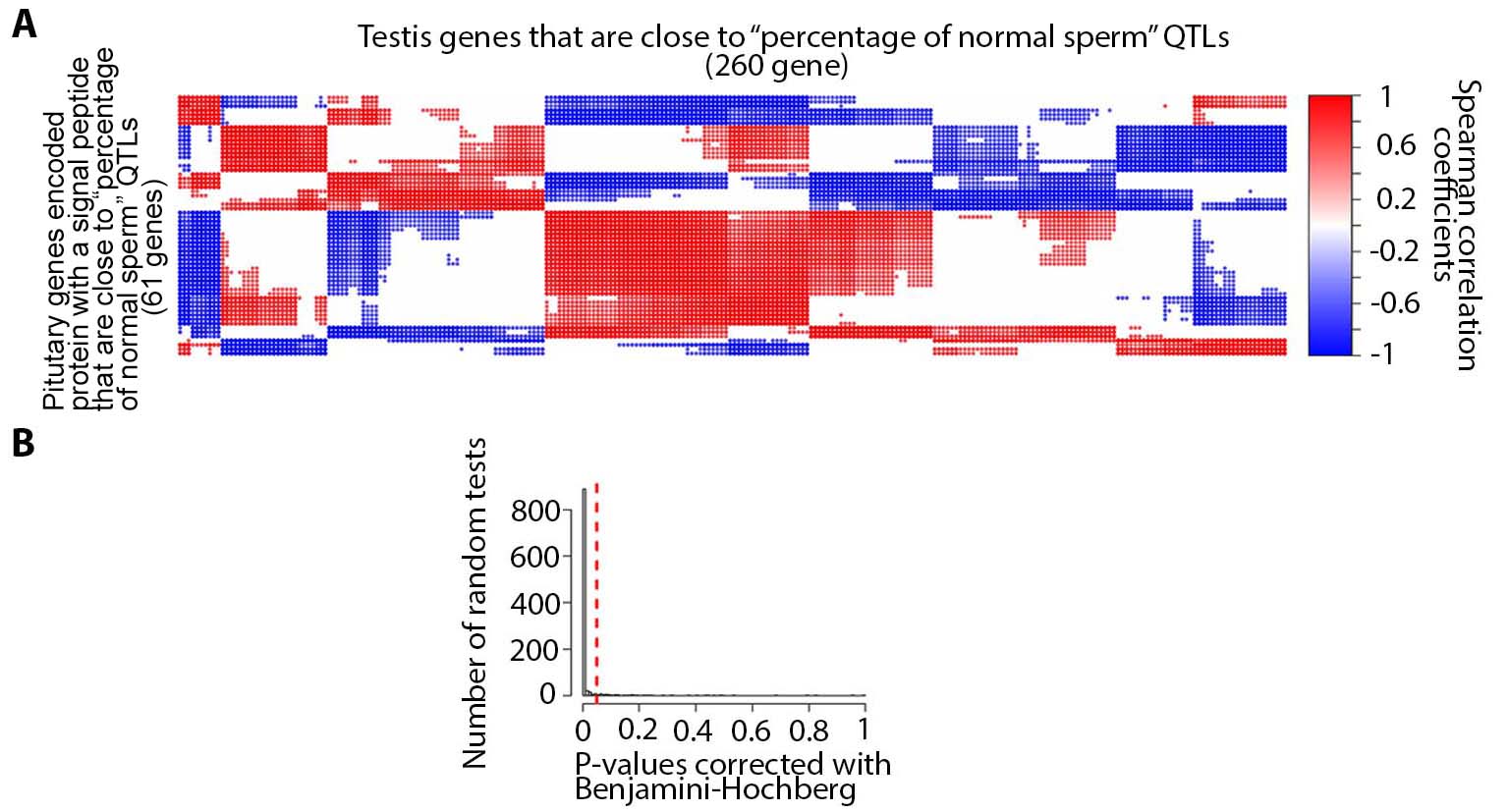


**Figure S33-** (A) Expression correlation between pituitary genes with signal peptide that were close to the “percentage of normal sperm” and testis genes closest to this trait (reference correlations). (B) distribution of p-values resulting from right sided t-test between reference correlations coefficients and correlation coefficients derived from random chance (see methods for details)**.**


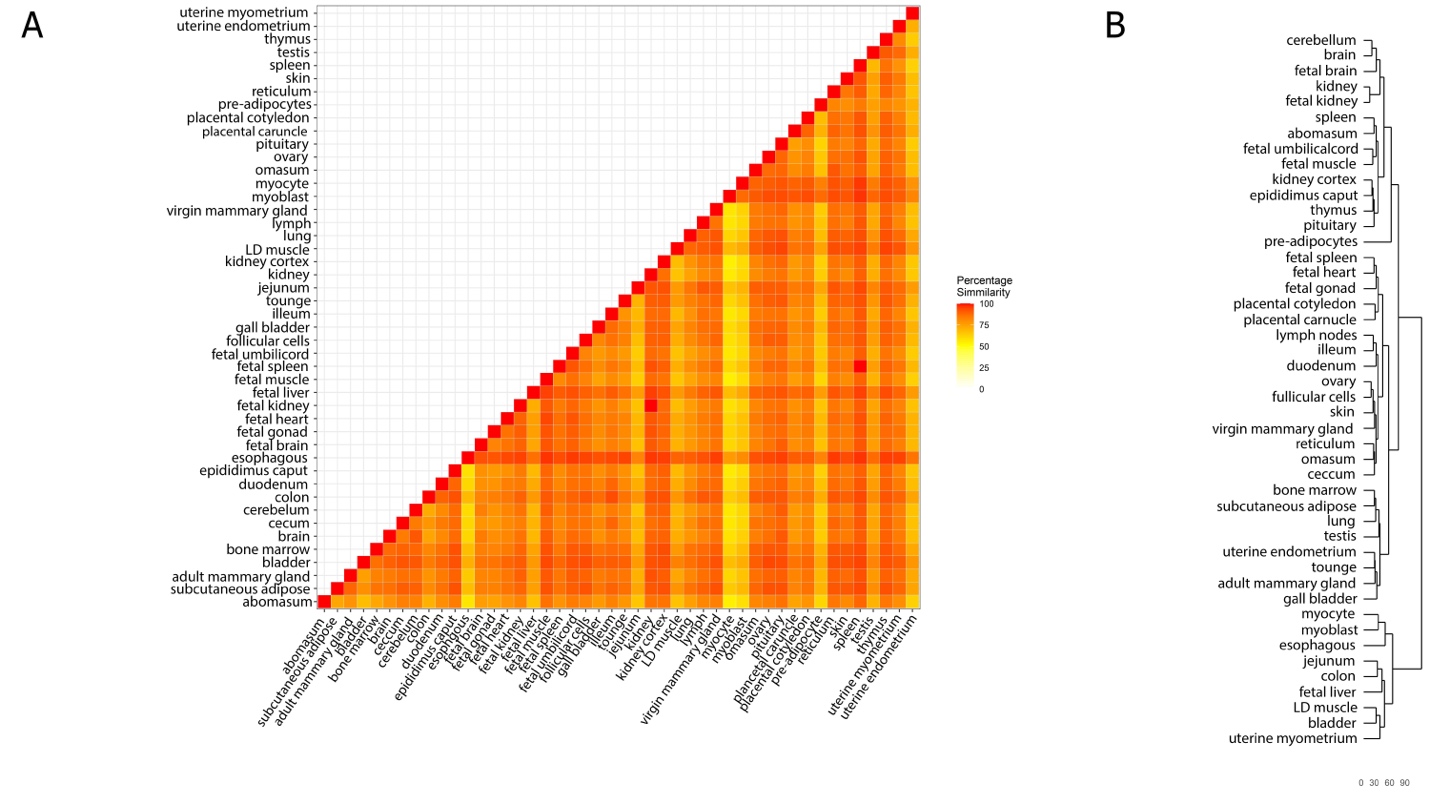


**Figure S34-** Tissue similarities (A) and clustering (B) based on the percentage of miRNAs shared between pairs of tissues.


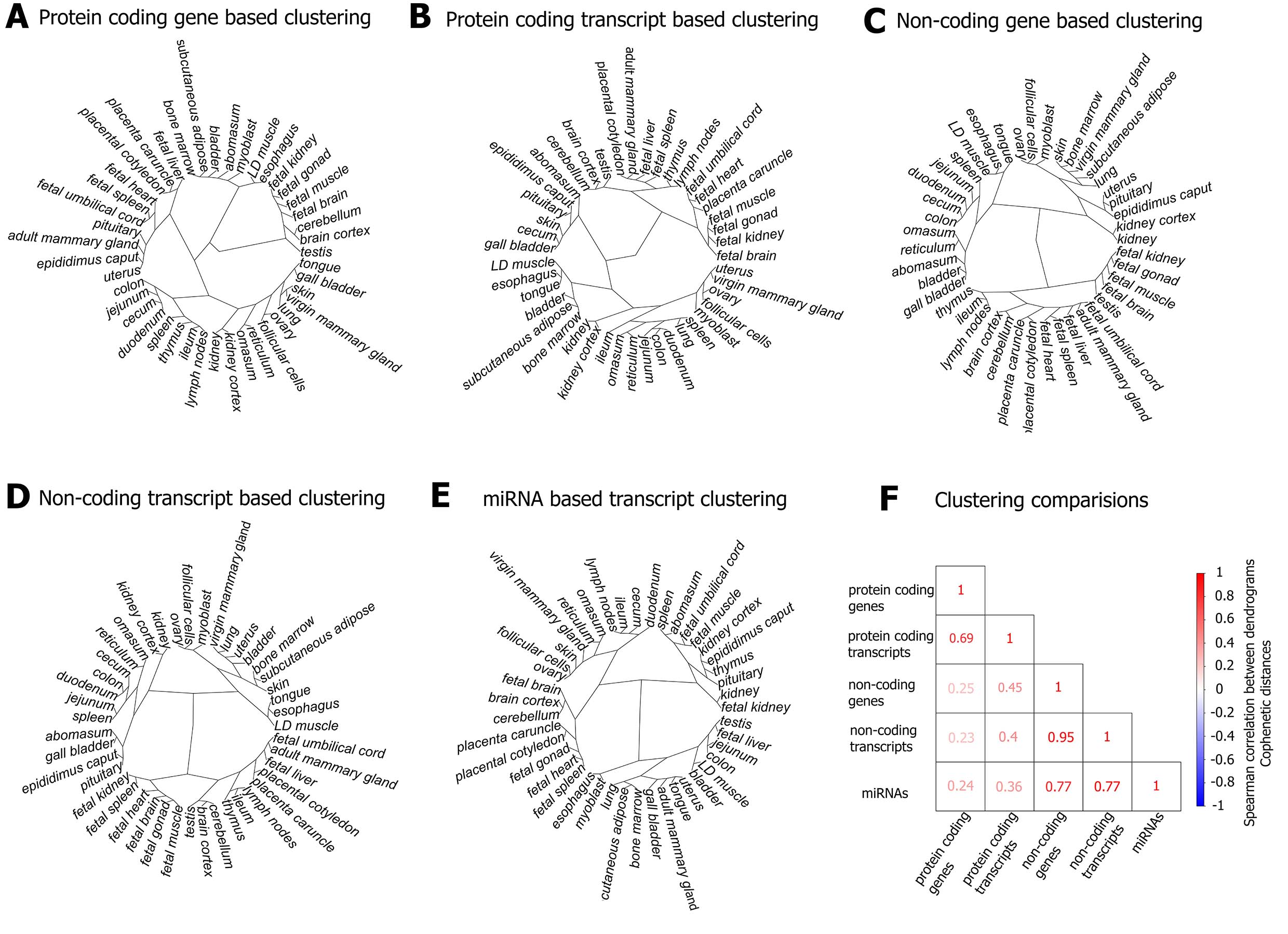


**Figure S35-** Clustering of tissues based on protein-coding genes (A), protein-coding transcripts (B), non-coding genes (C), non-coding transcripts (D) and miRNAs (E). (F) Comparison of tissue dendrograms based on the correlation between their Cophenetic distances.


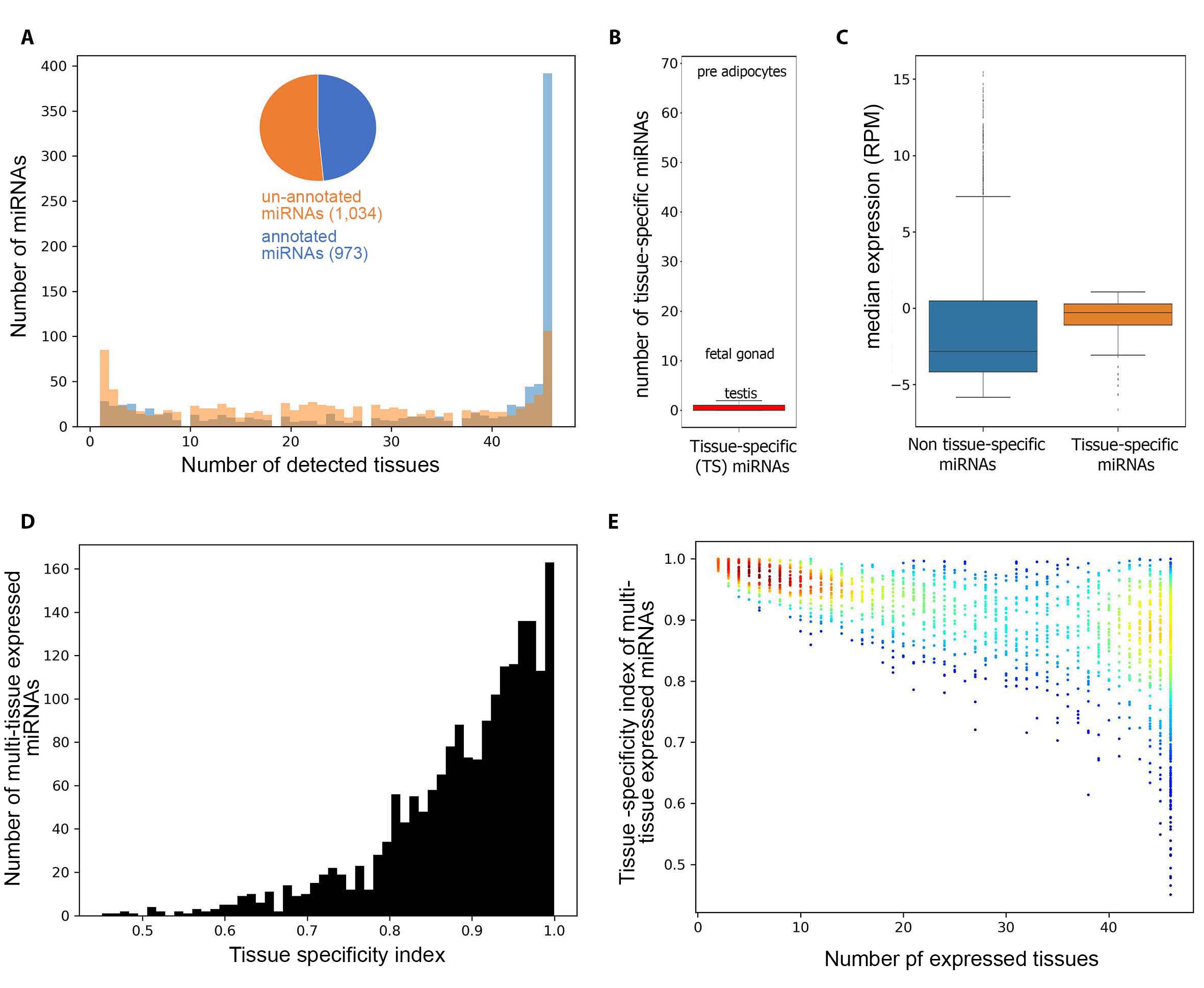


**Figure S36-** (A) Distribution of the number of detected tissues for known and novel miRNAs. Classification of miRNAs to known and novel was presented in the pie chart. (B) Comparison of tissues based on their number of tissue-specific miRNAs. (C) expression of known and novel miRNAs in their detected tissues. (D) Distribution of the tissue specificity indexes of multi-tissue detected miRNAs. (E) Relation between tissue specificity index and number of detected tissues in multi-tissue detected miRNAs. Dots have been color coded based on their density.


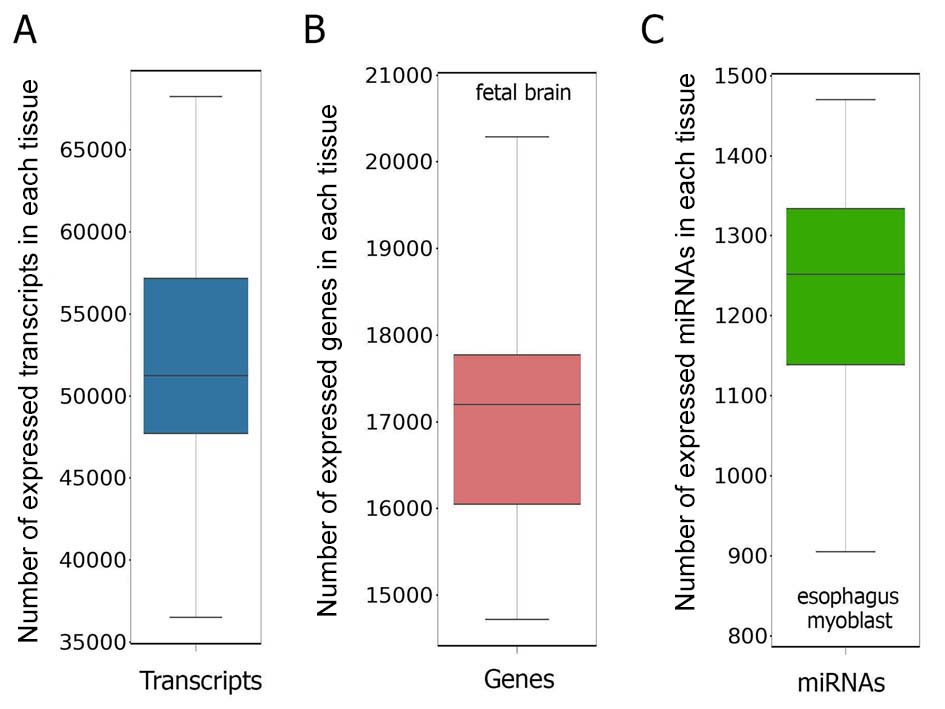


**Figure S37-** Distribution of the number of detected genes (A), transcripts (B), and miRNAs (C) across tissues.


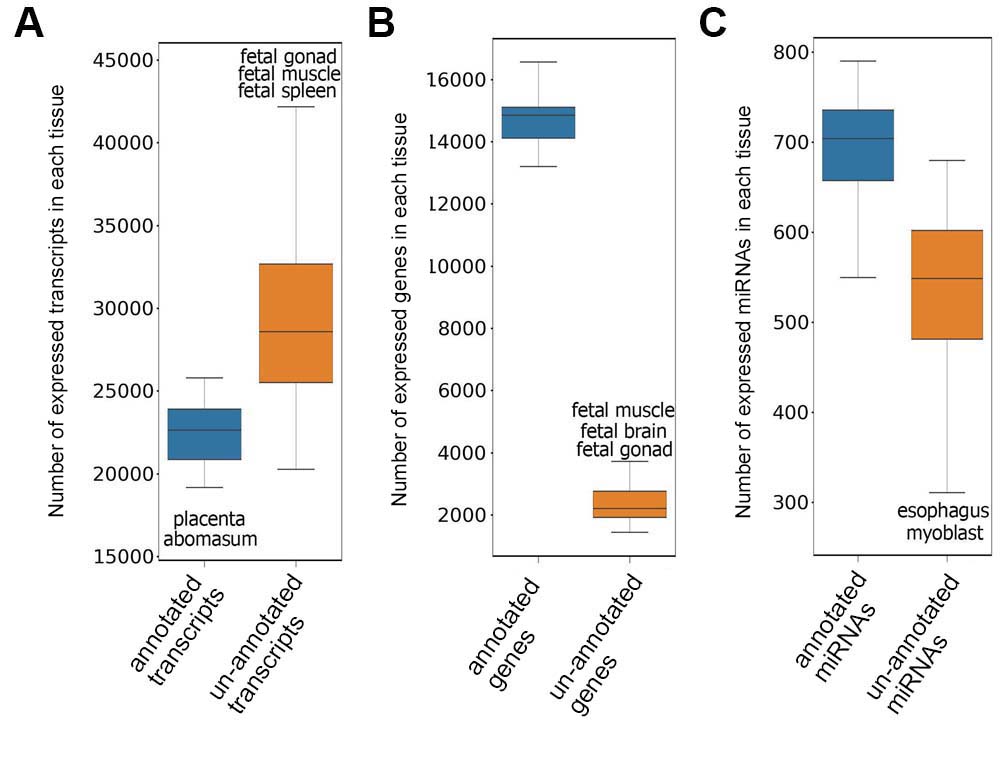


**Figure S38-** Distribution of the number of known and novel genes (A), transcripts (B), and miRNAs (C) across tissues.

**Figure S39-** Overview of the bioinformatics steps used in this study (see Methods for details)


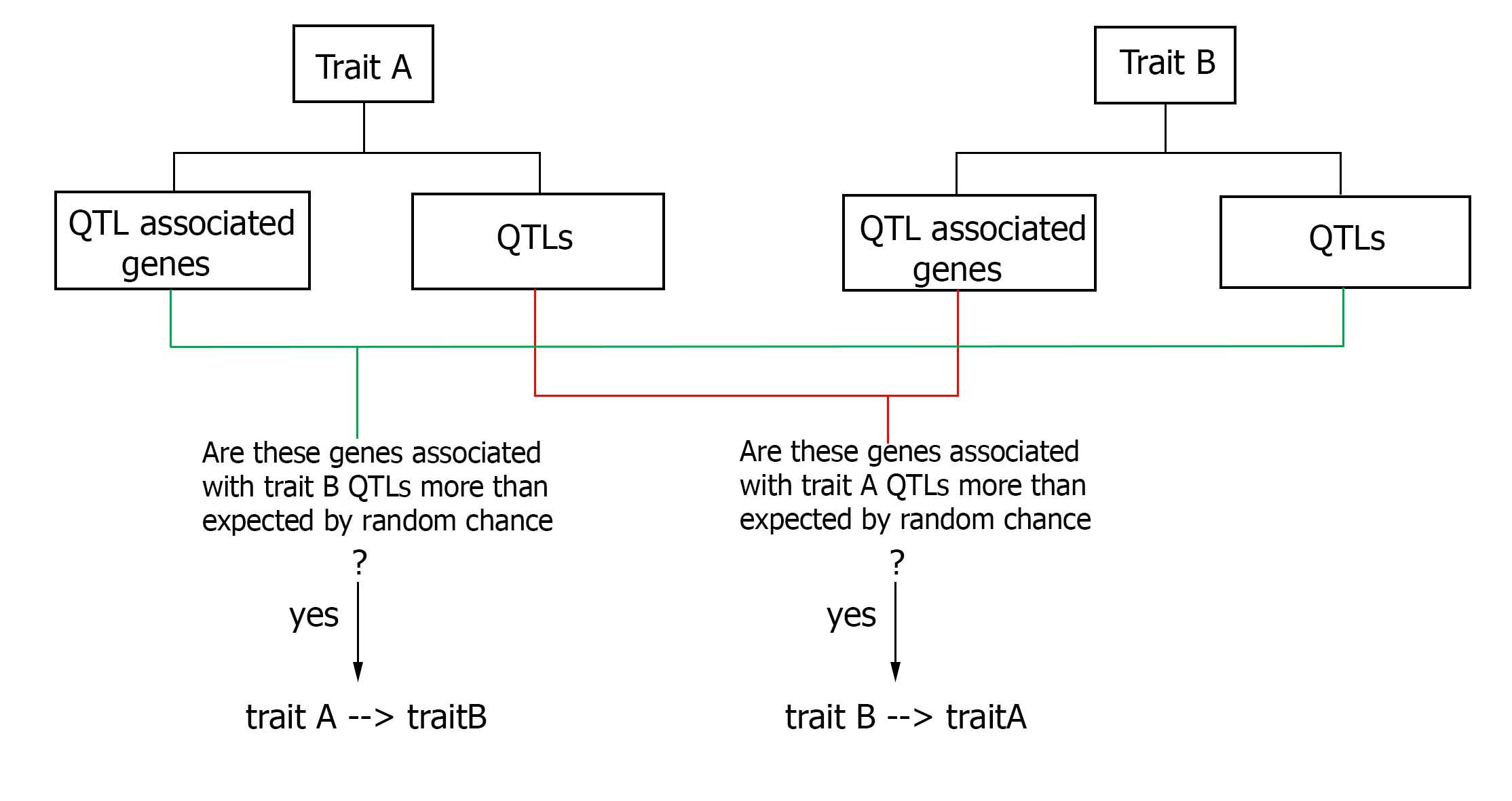


**Figure S40-** Graphical representation of the method used to construct tissue similarity network. Bioinformatics work-follow and custom codes used are available at https://github.com/hamidbeiki/Cattle-Genome.

**References**

Uhlén M, Fagerberg L, Hallström BM, Lindskog C, Oksvold P, Mardinoglu A, Sivertsson Å, Kampf C, Sjöstedt E, Asplund A et al. 2015. Proteomics. Tissue-based map of the human proteome. *Science* **347**: 1260419.
