# Supplementary material for: Enhanced bovine genome annotation through integration of transcriptomics and epi-transcriptomics datasets facilitates genomic biology
Source: Gigascience. 2024 Apr 16;13:giae019. doi: 10.1093/gigascience/giae019 (PMC11020238; doi:10.1093/gigascience/giae019)
Supplement: giae019_Supplemental_Files [file giae019_supplemental_files.zip › Supplemental_file23 (1).docx]

**Tissue and cell collection, and library construction RNA-seq, miRNA-seq, WTTS-seq, ATAC-seq, and ChIP-seq libraries**

**Cell sample collections.** Satellite cells were isolated from skeletal muscle by pronase digestion as described previously (Leng et al. 2019). The isolated satellite cells were activated to proliferate as myoblasts by culturing in growth medium composed of Dulbecco’s Modified Eagle Medium (DMEM), 10% fetal bovine serum (FBS), and 1% antibiotics-antimycotics. To induce myoblasts to differentiate into myocytes, myoblasts cultured in growth medium were switched to differentiation medium composed of DMEM and 2% horse serum for 2 days. Pre-adipocytes from subcutaneous fat were isolated by collagenase digestion as previously described (Hausman et al. 2008). To induce preadipocytes to differentiate into adipocytes, pre-adipocytes were initially cultured in growth medium (DMEM/F12, 10% FBS, 1% antibiotics-antimycotics) to reach confluency, then in induction medium (DMEM/F12, 10% FBS, 1% antibiotics-antimycotics, 10 μg/mL insulin, 1 μM dexamethasone, 0.5 mM isobutyl methylxanthine, and 200 μM indomethacin) for 2 days, and lastly in maintenance medium (DMEM/F12, 10% FBS, 1% antibiotics-antimycotics, 1 μg/mL insulin) for 10 days.

**Adult tissue collections.** Procedures for tissue collection followed the Animal Care and Use protocol (#18464) approved by the Institutional Animal Care and Use Committee (IACUC), University of California, Davis (UCD). Eleven cattle (6 males: M08, M09, M10, M11, M12, M23; and 5 females:F05, F06, F07, F12, M130) were slaughtered at UCD using captive bolt under USDA inspection at 420 days old and were intact male and female Line 1 Herefords that had the same sire, provided by Fort Keogh Livestock and Range Research Lab [1]. Tissue samples were flash frozen in liquid nitrogen then stored at –80 °C until further assay processing.

# **Fetal tissue collections.** Fetal sample collection and tissue collection were approved by IACU), University of Idaho (2017-67). Six pregnant females at day 78 of gestation Line 1 Herefords (Fetus male 1 male, Fetus male 2 male, Fetus male 3 male, Fetus male 4 male, Fetus male 5 male, Fetus male 6 male) were slaughtered at UI meats lab using captive bolt under USDA inspection. Animals were provided by Fort Keogh Livestock and Range Research Lab [1]. Tissue samples were flash frozen in liquid nitrogen then stored at –80 °C until further assay processing.

**Mammary gland tissue collection.** The 14 animals used in this study were Holstein-Friesian heifers from a single herd managed at the AgResearch Research Station in Ruakura, NZ. All experimental protocols were approved by the AgResearch, NZ, ethics committee, and carried out according to their guidelines. Samples were collected from animals at 5 time points: virgin state before pregnancy between 13 and 15 months of age (virgin), mid-pregnant at day 100 of pregnancy, late pregnant ~2 weeks pre-calving, early lactation ~2 weeks post-calving, and adult state ~36 month of age. Tissue samples were obtained by mammary biopsy using the Farr method [2]. Lactating cows were milked before biopsy and sampled within 5 hours of milking. Biopsy sites were clipped and given aseptic skin preparation (povidone iodine base scrub and iodine tincture) and subcutaneous local anesthetic (4 ml per biopsy site). Core biopsies were taken using a powered sampling cannula (4.5 mm internal diameter) inserted into a 2 cm incision. The resulting samples of mammary gland parenchyma measured 70 mm in length, with a 4 mm diameter.

Due to the limited amount of tissue sample collected from an individual animal. RNA for RNA-seq analysis was isolated from 4 animals, RNA for miRNA-seq was isolated from 6 animals, RNA for WTTS-seg was isolated from 4 animals, and DNA for ATAC-seq analysis from 7 animals (Supplemental file 1).

**RNA-seq library construction.** Tissue and cell samples (Supplemental file 1) were collected from live animal and stored at -80 °C and grounded to a powder using a mortar and pestle and liquid nitrogen. The tissue was next homogenized in QIAzol Lysis Reagent (Qiagen Catalog No. 79306) using a QIAshredder spin column (Qiagen Catalog No. 79656). After centrifugation, the lysate was mixed with chloroform, shaken vigorously for 15 sec, incubated for 2 – 3 min at room temperature, and centrifuged for 15 min at 12,000 x g at 4°C. The upper, aqueous phase was transferred to a new collection tube and 1.5 vol of 100% ethanol was added and mixed thoroughly by pipetting up and down several times. Total RNA was then isolated from the sample using the RNeasy Mini Kit (Qiagen Catalog No. 74106) according to the manufacturer’s instructions. Contaminating DNA was removed by treating total RNA with DNase (AM1906, Ambion). Total RNA quantity was measured with the Quant-It RiboGreen RNA Assay Kit (Life Technologies Corp., Carlsbad, CA) and quality assessed by fragment analysis (Advance Analytical Technologies, Inc., Ankeny IA).

High-quality total RNA (RIN > 7) was extracted from frozen mammary tissue using NucleoSpin® miRNA isolation kit (MACHEREY-NAGEL) according to the manufacturer’s protocol, isolating large and small (<200 bp) fractions separately. The “large” RNA fraction was used to prepare strand-specific poly(A)+ RNA-seq libraries for sequencing. The “small” RNA fraction was used to make miRNA-seq libraries using NEXTflex^TM^ Small RNA-Seq Kit v3.

**miRNA-seq library construction.** Tissue samples (Supplemental file 1) were collected similarly to the method described in the previous section. QIAseq miRNA Library Kit (Qiagen, cat no. 331505) and QIAseq miRNA NGS 96 Index IL Kit (Qiagen, cat no. 331565) were used to isolate miRNAs from all tissues except mammary gland. miRNAs from mammary gland were isolated using NEXTflex^TM^ Small RNA-Seq Kit v3 (Illumina) according to the manufacturer’s instructions. The isolated miRNA was subjected to 3’ ligation to ligate a pre-adenylated DNA adaptor to the 3’ ends of all miRNAs. An RNA adaptor was then ligated to the 5’ end of the mature miRNA to complete 5’ ligation. cDNA synthesis was completed using a reverse transcriptase (RT) primer containing integrated unique molecular identifiers (UMI). The RT primer bound to the 3’ adaptor region and facilitated conversion of the 3’/5’ ligated miRNAs into cDNA while a UMI was assigned to every miRNA molecule. After reverse transcription, a clean-up of the cDNA was performed using a streamlined magnetic bead-based method. Library amplification was accomplished by a universal forward primer from a plate being paired with 1 of 96 dried reverse primers in the same plate (Qiagen, cat no. 331565) to assign each sample a unique custom index. Following library amplification, a clean-up of the miRNA library was performed using a streamlined magnetic bead-based method. Libraries were then evaluated for quantity and quality measures before being normalized and pooled for llumina sequencing (1$\times$50bp).

**WTTS-seq library construction.** Construction of the WTTS-seq libraries from tissue samples (Supplemental file 1) involved fragmentation, poly(A)+ RNA enrichment, first-strand cDNA synthesis by reverse transcription and second-strand cDNA synthesis by PCR as described previously [3]. The starting material was 2.5 µg of total RNA per library, which was fragmented with 1 μl of 10X RNA fragmentation buffer (Ambion, AM8740), followed by enrichment of poly(A)+ RNA using Dynabeads (Ambion 61002). The poly(A)+ RNA molecules were then used for the first-strand cDNA synthesis with both 5’ adaptor (switching primer, 100 µM) and 3’ adaptor (containing oligo (dT10), 100 µM) catalyzed by the SuperScript III reverse transcriptase (200 U/μl) (Invitrogen, 18080). The first-strand cDNA molecules were chemically enriched with RNases I and H and used to synthesize the second-strand cDNA using PCR. Base PCR conditions were as follow: initial denaturation at 98 °C for 30 s, PCR cycles of 98 °C for 10 s, 50°C for 30 s, and 72°C for 30 s, and final extension at 72°C for 10 min. The size-selected cDNA (200 – 500 bp) was purified with SPRI beads (Agencourt AMPure XP beads, Beckman Coulter, Brea, CA) and sequenced using an Ion PGM™ Sequencer at Washington State University.

**ATAC-seq library construction.** Frozen tissue samples (Supplemental file 1) were pulverized under liquid nitrogen using mortar and pestle. Permeabilized nuclei were obtained by resuspending pulverized tissue (5-15 mg) in 250 µL Nuclear Permeabilization Buffer (0.2% IGEPAL-CA630 [I8896, Sigma], 1 mM DTT [D9779, Sigma], Protease inhibitor [05056489001, Roche], and 5% BSA [A7906, Sigma] in PBS [10010-23, Thermo Fisher Scientific]), and incubating for 10 min on a rotator at 4°C. Nuclei were then pelleted by centrifugation for 5 min at 500 x g at 4°C. The pellet was resuspended in 25 µL ice-cold Tagmentation Buffer (33 mM Tris-acetate [pH = 7.8; BP-152, Thermo Fisher Scientific], 66 mM K-acetate [P5708, Sigma], 11 mM Mg-acetate [M2545, Sigma], 16% DMF [DX1730, EMD Millipore] in molecular biology grade water [46000-CM, Corning]). An aliquot was then taken and counted by hemocytometer to determine nuclei concentration. Approximately 50,000 nuclei were resuspended in 20 µL ice-cold Tagmentation Buffer and incubated with 1 µL Tagmentation enzyme (FC-121-1030, Illumina) at 37 °C for 30 min with shaking at 500 rpm. The tagmentated DNA was purified using MinElute PCR purification kit (28004, Qiagen). The libraries were amplified using NEBNext High-Fidelity 2X PCR Master Mix (M0541, NEB) with primer extension at 72°C for 5 min, denaturation at 98°C for 30 s, followed by 8 cycles of denaturation at 98°C for 10 s, annealing at 63°C for 30 s and extension at 72°C for 60 s. Amplified libraries were then purified using MinElute PCR purification kit (28004, Qiagen), and two size selection steps were performed using SPRIselect bead (B23317, Beckman Coulter) at 0.55X and 1.5X bead-to-sample volume ratios, respectively. ATAC-seq libraries were sequenced on an Illumina Nextseq 500 platform using Nextra V2 sequencing chemistry to generate 2 × 75 paired-end reads.

**References**

1. Tixier-Boichard M, Fabre S, Dhorne-Pollet S, Goubil A, Acloque H, Vincent-Naulleau S, et al. Tissue Resources for the Functional Annotation of Animal Genomes. Front Genet. 2021;12:666265. doi:10.3389/fgene.2021.666265.

2. Farr VC, Stelwagen K, Cate LR, Molenaar AJ, McFadden TB and Davis SR. An improved method for the routine biopsy of bovine mammary tissue. J Dairy Sci. 1996;79 4:543-9. doi:10.3168/jds.S0022-0302(96)76398-1.

3. Zhou X, Li R, Michal JJ, Wu XL, Liu Z, Zhao H, et al. Accurate Profiling of Gene Expression and Alternative Polyadenylation with Whole Transcriptome Termini Site Sequencing (WTTS-Seq). Genetics. 2016;203 2:683-97. doi:10.1534/genetics.116.188508.
