# Supplementary material for: Enhanced bovine genome annotation through integration of transcriptomics and epi-transcriptomics datasets facilitates genomic biology
Source: Gigascience. 2024 Apr 16;13:giae019. doi: 10.1093/gigascience/giae019 (PMC11020238; doi:10.1093/gigascience/giae019)
Supplement: giae019_Response_to_Reviewer_Comments_Original_Submission [file giae019_response_to_reviewer_comments_original_submission.pdf]

Author's Response To Reviewer Comments

Dear Editor

Manuscript number: GIGA-D-23-00037

We are thankful to the reviewers for their thorough review. We have revised the present research manuscript in the light of their useful suggestions and comments. We hope this revision has improved the manuscript to a level of their satisfaction. Point by point answers to their specific comments are as follows. Please notice that that the line numbers were changed after revision. However, any changes were highlighted with red color in the revised version. With the exception of text that was deleted. Supplemental files 5, 14, 16, and 22 were submitted to GigaDB database.

Reviewer#1

Comment 1: Maybe a flow chart including samples (their number), methods, etc. will be helpful for authors to understand of the outline of this study when it supplied so much information. Besides, subheadings for the Results part needs to be detailed to supply a clear aim or result, for example, "Transcript level analyses".

Response: Lines 582 to 583 the overview of the bioinformatics steps used in this study has been provided. Lines 103 and 187, the "Transcript level analysis" and "Gene level analysis" have been changed to "Transcript-based analysis" and "Gene-based analysis" to provide more clear title for the subsections.

Comment 2: Predicted un-annotated genes and transcripts were highly supported by independent Pacific Biosciences single molecule long-read isoform sequencing (PacBio Iso-Seq), Oxford Nanopore Technologies sequencing (ONT-seq), Illumina high-throughput RNA sequencing (RNA-seq), Whole Transcriptome Termini Site Sequencing (WTTS-seq), RNA Annotation and Mapping of Promoters for the Analysis of Gene Expression (RAMPAGE), chromatin immunoprecipitation sequencing (ChIP-seq), and Assay for Transposase-Accessible Chromatin using sequencing ATAC-seq) data.

How did this validation applied using those different datasets? Which one was treated as standard, or were they validated mutually by overlapping? Detail information is needed to supply to help others to refer this study when they compare with their own datasets. Standard workflow will help the cattle study to go faster, and this will be a very important contribution.

Response: Lines 646 to 657, the detailed description of the comparison of transcript structures across

dataset has been provided.

Comment 3: Testis showed the highest number of expressed genes with observed transcripts compared to other tissues. Fetal brain and fetal muscle tissues showed the highest number and percentage of non-coding genes compared to that observed in other tissues.

When evaluated the gene/transcript number for different tissues, were the numbers corrected by the sequencing depth/the sample number of different tissues? How to define the testis including the highest number of expressed genes? Is there any potential interesting biological mechanism for this phenomenon?

Response: Lines 111-115, and 628-629, the quantified gene, transcript counts were normalized for the sequencing depth using reads per kilobase of transcript per Million reads mapped (RPKM) method.

Testis showed the highest number of expressed genes compared to other tissues (Supplemental file 2: Fig. S8). In addition, the testis stands out, compared to other tissues, for the high number of tissue-specific genes and transcripts (Supplemental file 2: Fig. S28C, Supplemental file 13). The same results have been observed in human [1-4]. Although the reason behind these phenomena is largely remained unknown, it can be referred to the complex anatomical and functional features of testis [4].

References

1. Djureinovic D, Fagerberg L, Hallstrom B, Danielsson A, Lindskog C, Uhlen M, et al. The human testis-specific proteome defined by transcriptomics and antibody-based profiling. Mol Hum Reprod. 2014;20 6:476-88. doi:10.1093/molehr/gau018.

2. Fagerberg L, Hallstrom BM, Oksvold P, Kampf C, Djureinovic D, Odeberg J, et al. Analysis of the human tissue-specific expression by genome-wide integration of transcriptomics and antibody-based proteomics. Mol Cell Proteomics. 2014;13 2:397-406. doi:10.1074/mcp.M113.035600.

3. Uhlen M, Fagerberg L, Hallstrom BM, Lindskog C, Oksvold P, Mardinoglu A, et al. Proteomics. Tissue-based map of the human proteome. Science. 2015;347 6220:1260419. doi:10.1126/science.1260419.

4. Pineau C, Hikmet F, Zhang C, Oksvold P, Chen S, Fagerberg L, et al. Cell Type-Specific Expression of Testis Elevated Genes Based on Transcriptomics and Antibody-Based Proteomics. J Proteome Res. 2019;18 12:4215-30. doi:10.1021/acs.jproteome.9b00351.

Reviewer#2

Comment 1: My main concern is regarding the way that the results are presented and discussed. Despite the authors presenting very interesting results, the manuscript is very difficult to follow. In addition to a very long manuscript, which could be understandable due to the amount of analysis and results, the text

seems to be extremely repetitive and basically descriptive. The results section, which has almost 20 pages, is composed of a series of sub-sections that are mainly descriptive statistics of the data. This kind of information could be summarized in Tables/Figures and the main results presented in the text. I suggest the authors perform a deep review in the Results section in order to provide a reduced version with the most relevant results, which will be further discussed. Additionally, the same information is presented in several parts of the manuscript. For example, the tissue-specific genes and transcripts are mentioned in multiple parts of the results section. In my opinion, the main objective of the authors "to facilitate the functional genomics of cattle" relies much more on other results rather than on the description of a number of transcripts, expressed genes, etc. For example, a deeper analysis of the alternative splicing across tissues would result in much more interesting results from the functional point-of-view. Additionally, the authors could focus on the functionality of the transcript with specific expression signatures (in a cluster of tissues, for example). The extensive description of summary statistics reduces substantially the impact and novelty of the results.

Response: The redundant summary statistics and unnecessary results were removed throughout the manuscript. The detailed description of different alternative splicing events was moved to the method section, to make the manuscript shorter (lines 734-750). The redundant tissue-specific transcript result was removed as it caused confusion (lines 103-105). Tissue sample collection and sequencing library preparation methods were moved to the Supplemental file 23, to make the manuscript shorter (lines 581-582)

The functionality of transcripts/genes were discussed thought the manuscript (lines 222-224, 235-238, 244-248, 260-262, 345-347, 371-374, 396-400, and 519-533). we provided an initial publication from which additional publications will arise. We fully acknowledge that there are additional analyses that can be performed based on this data, however it is beyond the scope of this publication.

Comment 2: The material and methods section should be improved. I understand that due to the length of the manuscript, the authors decided to not show some details regarding the analysis and only cite the original manuscript where the analyses were performed. However, the authors should present the most relevant points, arguments, and decisions from each methodology. A reduction in other parts of the manuscript will allow the authors to improve this section as well.

Response: Lines 641-645, and 700-705, a brief description of the independent Oxford Nanopore and ChIP seq experiments that their resulted data were used in this study, has been added to the manuscript to improve the section.

Comment 3: The Discussion section is pretty much an overview of the results section. I believe that because the authors choose to focus mainly on the description of the number of transcripts, isoforms, genes, etc. providing discussion based on functionality became a difficult task. Here, the authors should discuss how the results help to improve the functional annotation in the cattle genome. In general, the discussion is generic and don't cover specific results obtained in the analysis. For example, which is the functional profile of the genes with specific alternative splicing in a given tissue or group of tissues? This is interesting from the functional perspective. The results of the QTL-transcriptome associations should be discussed more in detail, providing more information regarding these associations and the specific patterns of association regarding the tissues and isoforms. However, it is very important to highlight the limitation of this approach, such as the limitations related to the database, the original association studies, breed-specific associations, etc.

Response: In the discussion section, we explained how our effort improved the current annotation of cattle genome both in quantity, i.e., number of novel genes/transcripts/miRNAs (lines 437-448), and quality, i.e., UTRs and regulatory elements (lines 449-457), bifunctional genes (lines 458-473), known gene border extensions (lines 497-501), through comparison our assembled transcriptome with current genome annotations or greatly annotated human genome. We latter discussed our finding on (1) pseudogene-derived lncRNAs and their role in gene regulation (lines 492-496), (2) similarity of alternative splicing events in cattle and other vertebrates (lines 506-509), (2) change of the alternative splicing between fetal and adult tissues and how this finding supported by other experiments in human genome (lines 509-511), (3) integration of our assembled transcriptome with previously published QTL/gene association data and how this novel approach can be used to identify tissue-tissue communication mechanisms (lines 512-541), and study trait similarity network (lines 542-551). The limitation of this approach was presented in lines 558-562.

The functional enrichment analysis of the top five percent of genes with the highest number of alternative-splicing events was presented in lines 344-347 It should be noted that due to the genome-wide scope of this experiment, and the number of studied tissues, there are so many contests that could be performed, and addressing all of them would make the manuscript extremely long, which constricts the reviewer's first comment. While we fully understand the review comment, we will not be able to provide all possible evidence.

Comment 4: Finally, I would suggest the authors remove multi-omics from the title. The study focuses on a multi-platform and multi-technique approach to evaluate transcriptomics. The closest analysis from other omics was the integration of ATAC-Seq and Chip-Seq data. However, the main results are focused on a single omics, transcriptomics.

Response: The manuscript title was changed to "Utilization of functional genomics data to identify relationships between phenotypic traits in cattle".

Comment 5: The abstract should be substantially improved. There are few explanations about the scientific question and hypothesis of the study. Additionally, the authors don't provide basic information regarding the dataset used in the study. Which were tissues analyzed? How many animals? The conclusions are vague and don't provide a perspective of the results.

Response: The nature of this experiment is different than a traditional treatment by treatment experiment in combination of limitation of the length of the abstract is not possible to state all of the hypothesis that been tested.

Comment 6: Lines 51-53: This sentence is not connected with the previous one. Please, inform us how functional elements may help to fill the mentioned gap.

Response: Lines 61-63, a new sentence was added to the paragraph to fill the gap.

Comment 7: Line 56: Reference 2, Does this reference really reach this conclusion?

Response: Lines 66-68, the citation was changed as it caused confusion.

Comment 8: Line 58: Reference 3, The reference regarding this topic is quite old. Please, provide an updated one since the topic of the sentence passed through an intense development and increase in the number of publications in the last decade.

Response: Line 70, the citation was updated.

Comment 9: The last paragraph of the introduction presents a summary of the results obtained. The authors could use this part of the introduction to clearly state the objectives of the study.

Response: Lines 83-89, the paragraph was rewritten to reflect the study objectives.

Comment 10: Line 85: The word "diversity" is repeated in the sentence.

Response: Lines 91, the redundant word was removed.

Comment 11: Line 91: Where is the description of all tissues?

Response: Line 91-93, the list of tissues was provided in Supplemental file 1.

Comment 12: Line 103-105: How? It is not clear how these 20,010 transcripts were actually expressed in multiple tissues.

Response: Lines 109-115, reliance solely on assembled transcripts in a given tissue to predict a tissue transcript atlas may overestimate tissue specificity due to a high false-negative rate for transcript detection. To solve this problem of over-prediction of tissue specificity, we marked a transcript as

“expressed” in a given tissue only if (1) it had been assembled from RNA-seq data in that tissue; or (2) its expression and all of its splice junctions has been quantified using RNA-seq reads in the tissue of interest with an expression level more than 1 reads per kilobase of transcript per Million reads mapped (RPKM)

Comment 13: Line 156: "Significantly higher than that was", please, review this sentence.

Response: Line 116-146, the sentence was corrected as it caused confusion.

Comment 14: Line 159-163: This sentence is confusing.

Response: Line 148-151, the sentence was corrected as it caused confusion.

Comment 15: Line 226-227: Please, replace "This supported an intersection analysis" with "This supports an intersection analysis".

Response: Line 201-203, the sentence was corrected as it caused confusion.

Comment 16: Line 247-250: This is a very broad BP term. How this could be interpreted?

Response: The details of all over-represented GO terms were provided in the supplemental file 7, and only the most enriched term was reported in the manuscript body. High level of similarity between enriched GO terms (based on the similarity of their associated genes), makes it fair to use “response to protozoan” as the representative biological function for genes with the highest number of UTRs (Supplemental file 2: Fig. 11)

Comment 17: Line 266-267: How does a protein-coding gene transcribe only non-coding transcripts? Please, provide more details to the readers.

Response: Line 239-241, the sentence was re-written as it caused confusion. In addition, bifunctional genes were discussed in more detail in the discussion section (lines 458-473).

Comment 18: Line 409-410: It seems that this information is repeated.

Response: Lines 115-117, the redundant sentence was removed

Comment 19: Line 611: It is missing a parenthesis.

Response: Line 554, the missed parenthesis was fixed.

Comment 20: The conclusions are generic and don't cover the main results obtained in the studies from a perspective of how those results fill the current gap observed in the literature. How the specific results obtained.

Response: Lines 566-578, the conclusion section was modified to cover the study objectives provided in lines 83-89

Reviewer#3

Comment 1: In the Methods section, sub heading RNA-seq library construction it says, "Tissue samples (Supplemental file 22) were collected from storage at -80 °C". A section prior to that describes adult tissue collection methods stating that 2 male and 2 female cattle were used. Neither section nor Sup file 22 include the animal identifier or any means to determine which tissue samples were used from which donor animal. Maybe sup file 22 could be expanded to include columns for each of the 4 animals with y/n datum to identify which tissues were sequenced from each animal? Or perhaps instead of y/n you could include the BioSample accession number of the deposited data for those used.

Response: The number of sampled animals were corrected in the Supplemental file 23 (lines 18, and 24). In addition, the detail of datasets generated in the experiment was provided in Supplemental file 1 (line 81).

Comment 2: The RNA-seq library construction section also mentioned that RNA quantity and quality was measured. While not required, we would encourage you to share those results in GigaDB.

Response: Given the Information is not required for the manuscript; we would prefer not to provide those Information.

Comment 3: Mammary gland tissue collection and RNA-seq library construction section; previous discussion on this topic resulted in you changing the text to:

"Mammary gland tissue collection. The 14 animals used in this study were Holstein-Friesian heifers from a single herd managed at the AgResearch Research Station in Ruakura, NZ. All experimental protocols were approved by the AgResearch, NZ, ethics committee and carried out according to their guidelines. Samples were collected from animals at 4-time points: virgin state before pregnancy between 13 and 15 months of age (virgin), mid-pregnant at day 100 of pregnancy, late pregnant ~2 weeks pre-calving, and early lactation ~2 weeks post-calving. Tissue samples were obtained by mammary biopsy using the Farr method [2]. Lactating cows were milked before biopsy and sampled within 5 hours of milking. Biopsy sites were clipped and given aseptic skin preparation (povidone-iodine base scrub and iodine tincture) and subcutaneous local anesthetic (4 ml per biopsy site). Core biopsies were taken using a powered sampling cannula (4.5 mm internal diameter) inserted into a 2 cm incision. The

resulting samples of mammary gland parenchyma measured 70 mm in length with a 4 mm diameter.

Due to the limited amount of tissue samples collected from an individual animal. RNA for RNA-seq analysis was isolated from 4 animals, RNA for miRNA-seq was isolated from 6 animals, RNA for WTTS-seg was isolated from 4 animals, and DNA for ATAC-seq analysis from 7 animals (SUPPLEMENT FILE NO)."

Based on the revised text it is still not possible to determine which individuals have been used for which assays. Could a similar table to the one suggested for the tissue samples above (1) be created here?

Response: Lines 91-93, and Supplemental file 23 (line 43) the detail of datasets generated in the experiment was provided in Supplemental file 1.

Comment 4: The Illumina RNA-Seq technologies section includes the text "Only samples with RIN values >8 were used for cDNA synthesis" (note- RIN needs to be added to the list of abbreviations in the document), it is not possible to determine from this which samples were actually used in this experiment and which were not. Perhaps it would be appropriate to share the RNA integrity analysis results here? GigaDB can host electrophoresis gel images if that is how it was performed.

Response: Given the Information is not required for the manuscript; we would prefer not to provide those Information.

Comment 5: The supplemental files provided in the user115 area. These all include the tissue name in their file-names, some have spelling mistakes, but even taking those into account I find 51 different tissues in those names, but the manuscript states 47 were investigated. Its probably just a classification and/or different subsets of things, but for transparency using a consistent nomenclature and providing accession numbers will be useful. Please ensure the files are named correctly with the appropriate tissue names.

Response: Lines 91-93, The diversity of RNA and miRNA transcript among 50 different bovine tissues and cell types was assessed using polyadenylation (poly(A)) selected RNA-seq (47 tissues) and miRNA-seq (46 tissues) and data (Supplemental file 1). The misspelled tissue names were corrected in figures and supplemental files.

Comment 6: miRNAs. The set of "supplemental file 21" files provided in user115 area all list the miRNAs by

some sort of identifier and state whether they are known or novel. Do those identifiers relate directly to miRbase? And have they all been deposited and released already? I tried to search for one of the novel ones "bta-miR-X44036" in miRbase but it did not find anything.

Response: The second column in supplemental file 22 identifies the novelty of predicted miRNAs. All miRNA with "bta-miR-X…" ID structure, were identified as "novel" in supplemental file 22.

Comment 7: Gene expression analysis. I believe from the methods section that you pooled all transcripts from all similar/same tissues and determined the tissue the expression levels based on those. From my limited understanding of statistics, I would assume it better to do a per sample analysis of the expression levels first to enable one to determine confidence levels by biological replicates.

The methods also state that "…outlier samples were expressed and removed from downstream analysis. Samples from each tissue were combined to…". For transparency and reproducibility, please provide a list of the removed samples and a list of those samples data that were combined (ideally that will include both the tissue names and the relevant SRA sequence run accession numbers).

Response: Sample-wise analysis were used to detect outlier samples (lines 592-594, and Supplemental file 2: Fig. S39), and tissue-tissue interconnection analysis (lines 390-391, Supplemental file 2: Fig. S39). The outlier samples were removed from the downstream analysis and were not submitted to SRA. Samples from each tissue were combined to get the most comprehensive set of data in each tissue for transcriptome assembly process (lines 595-596, Supplemental file 2: Fig. S39). The detail of datasets generated in the experiment was provided in Supplemental file 1 (lines 91-93).

Comment 8: "The resulting transcripts from each tissue were re-grouped into gene models using an in-house Python script. Structurally similar transcripts from the different tissues (see Comparison of transcript structures across datasets/tissues section) were collapsed using an in-house Python script to create the RNA-seq based bovine transcriptome."

Please confirm that those two in-house scripts are included in the GitHub repository cited in the data availability section? If not, please add them there.

Response: Lines 1032-1033, custom codes used in the experiment are available at https://github.com/hamidbeiki/Cattle-Genome.

Comment 9: ONT data analysis. You have cited the manuscript describing the data you have reused (Halstead et al 2021) which is great, thank you. However, having had a quick look at that manuscript it is not clear exactly what data you have reused, the only accession they quote in that manuscript is to a massive series of data hosted in GEO (GSE160028) which includes Pig, Cow and Chicken data. For the convenience of your readers would you also be able to point to a more useful accession of the data you actually utilized here e.g. the assembled isoform sequences?

Response: Lines 641-645, the detail of ONT samples used in the study was provided in Supplemental file

24

Comment 10: The correlation between the various methods sections and the data being made available is very difficult to determine with any certainty. Perhaps it would be beneficial to expand the sample table provided to include all the unique identifiers for every sample and correlate those to the methodologies listed in the manuscript. It maybe appropriate to incorporate a column to denote the samples removed from certain analysis, with an explanation as to why?

Including the ENA sample and/or BioSample accessions in the sample table (the ENA sample accessions start with ERS, BioSample accessions start with SAMEA) will greatly enhance the transparency of the data utilised in this study. In addition it will allow you to double check the metadata you have provided on each sample.

For example; I picked one at random to look into more closely. It is listed in the Samples_meta-daat.tsv spreadsheet you provided as having the accession "ERR10162191" (which is a run accession not a sample accession). I have compared this to the data submitted to Array Express (https://www.ebi.ac.uk/biostudies/arrayexpress/studies/E-MTAB-12052/sdrf?full=true) to find that run accession number and look up the relevant BioSample and ENA Sample accessions (ERS13425945, SAMEA111328380). In doing so I noticed that the "individual" value given in your spreadsheet says "M08" yet in Array Express it says "M22"? Clearly, one of those cannot be correct. As it was honestly the first and only sample, I looked at in such depth, it worries me that there maybe other inconsistencies that you will need to check and correct.

May I suggest you have someone in your team take a very careful look at the Samples submitted to Array Express, including the various different accessions that they assign (ENA sample accessions and BioSample accessions) and ensure that all sample have been submitted and have accurate and complete metadata, the geolocation information should be included with all samples. (NB the more metadata you can provide to the archives the more discoverable and reusable your data becomes). Then prepare the Samples spreadsheet from that information and relate it directly to the experiments described in the manuscript at the sample level.

Response: The detail of datasets generated in this experiment and independent datasets used in the experiment was provided in Supplemental file 1 (lines 91-93) and Supplemental file 24 (lines 641-645), respectively. The “ENA Accession” was corrected to “ENA Run Accession” in Supplemental file 1 as it caused confusion. The misunderstanding was raised from “Description” column provided by ArrayExpress database. This column reflecting the old animal id that we used in this study. The animal related to the "ERR10162191" sample is M08 in both Supplemental file 1 and ArrayExpress database. To check this sample metadata on the ArrayExpress database we followed the following steps: (1) find the related experiment id (E-MTAB-12052) from the Supplemental file 1 in the database (https://www.ebi.ac.uk/biostudies/arrayexpress/studies/E-MTAB-12052?query=E-MTAB-12052); (2) download the experiment metadata file (E-MTAB-12052.sdrf.txt); (3) look for ERR10162191 sample at “Comment[ENA_RUN]” column and related it’s animal id at “Characteristics[individual]” column. Samples metadata were checked to ensure the accuracy of information. We are in the progress of working with the ArrayExpress database to fix the metadata issues.
