# Supplementary material for: Enhanced bovine genome annotation through integration of transcriptomics and epi-transcriptomics datasets facilitates genomic biology
Source: Gigascience. 2024 Apr 16;13:giae019. doi: 10.1093/gigascience/giae019 (PMC11020238; doi:10.1093/gigascience/giae019)
Supplement: giae019_Response_to_Reviewer_Comments_Revision_1 [file giae019_response_to_reviewer_comments_revision_1.pdf]

Author's Response To Reviewer Comments

We have uploaded a response to reviewer comment document with the rest of the manuscript files. In it, we addressed all review comments point by point.

One item of note, we have submitted all of the requested changes to GigaDB, but we have not seen that the changes have been made.

All the best,

Jim Reecy
