# Supplementary material for: Enhanced bovine genome annotation through integration of transcriptomics and epi-transcriptomics datasets facilitates genomic biology
Source: Gigascience. 2024 Apr 16;13:giae019. doi: 10.1093/gigascience/giae019 (PMC11020238; doi:10.1093/gigascience/giae019)
Supplement: giae019_Reviewer_1_Report_Original_Submission — Yang Zhou -- 4/12/2023 Reviewed [file giae019_reviewer_1_report_original_submission.pdf]

Reviewer Report

Title: Enhancing Bovine Genome AnnotationThrough Integration of Transcriptomics and Epi-Transcriptomics Datasets Facilitates Genomic Biology

Version: Original Submission **Date:** 4/12/2023

Reviewer name: Yang Zhou

Reviewer Comments to Author:

This is an amazing study including huge annotation work for cattle genome. It identified 171,985 high quality unique transcripts with 69% of them un-annotated. Moreover, genes'/transcripts' diversity among tissues, different development stages were compared and their similarities with other species were analyzed. This updated the known knowledge of cattle transcriptome, and will be significant for understanding the regulation mechanism of body traits. This manuscript was recommended to be accepted after a minor revision.

1. Maybe a flow chart including samples (their number), methods, etc will be helpful for authors to understand of the outline of this study when it supplied so much information. Besides, subheadings for the Results part needs to be detailed to supply a clear aim or result, for example, "Transcript level analyses".

2. Predicted un-annotated genes and transcripts were highly supported by independent Pacific Biosciences single molecule long-read isoform sequencing (PacBio Iso-Seq), Oxford Nanopore Technologies sequencing (ONT-seq), Illumina high-throughput RNA sequencing (RNA-seq), Whole Transcriptome Termini Site Sequencing (WTTS-seq), RNA Annotation and Mapping of Promoters for the Analysis of Gene Expression (RAMPAGE), chromatin immunoprecipitation sequencing (ChIP-seq), and Assay for Transposase-Accessible Chromatin using sequencing ATAC-seq) data.
How did this validation applied using those different datasets? Which one was treated as standard, or were they validated mutually by overlapping? Detail information is needed to supply to help others to refer this study when they compare with their own datasets. Standard work flow will help the cattle study to go faster, and this will be a very important contribution.

3. Testis showed the highest number of expressed genes with observed transcripts compared to other tissues. Fetal brain and fetal muscle tissues showed the highest number and percentage of non-coding genes compared to that observed in other tissues
When evaluated the gene/transcript number for different tissues, were the numbers corrected by the sequencing depth/the sample number of different tissues? How to define the testis including the highest number of expressed genes? Is there any potential interesting biological mechanism for this phenomenon?

Methods

Are the methods appropriate to the aims of the study, are they well described, and are necessary controls included? Choose an item.

Conclusions

Are the conclusions adequately supported by the data shown? Choose an item.

Reporting Standards

Does the manuscript adhere to the journal's guidelines on minimum standards of reporting? Choose an item.

Choose an item.

Statistics

Are you able to assess all statistics in the manuscript, including the appropriateness of statistical tests used? Choose an item.

Quality of Written English

Please indicate the quality of language in the manuscript: Choose an item.

Declaration of Competing Interests

Please complete a declaration of competing interests, considering the following questions:

- Have you in the past five years received reimbursements, fees, funding, or salary from an organisation that may in any way gain or lose financially from the publication of this manuscript, either now or in the future?
- Do you hold any stocks or shares in an organisation that may in any way gain or lose financially from the publication of this manuscript, either now or in the future?
- Do you hold or are you currently applying for any patents relating to the content of the manuscript?
- Have you received reimbursements, fees, funding, or salary from an organization that holds or has applied for patents relating to the content of the manuscript?
- Do you have any other financial competing interests?
- Do you have any non-financial competing interests in relation to this paper?

If you can answer no to all of the above, write 'I declare that I have no competing interests' below. If your reply is yes to any, please give details below.

I declare that I have no competing interests

I agree to the open peer review policy of the journal. I understand that my name will be included on my report to the authors and, if the manuscript is accepted for publication, my named report including any attachments I upload will be posted on the website along with the authors' responses. I agree for my

report to be made available under an Open Access Creative Commons CC-BY license (http://creativecommons.org/licenses/by/4.0/). I understand that any comments which I do not wish to be included in my named report can be included as confidential comments to the editors, which will not be published.

Choose an item.

To further support our reviewers, we have joined with Publons, where you can gain additional credit to further highlight your hard work (see: https://publons.com/journal/530/gigascience). On publication of this paper, your review will be automatically added to Publons, you can then choose whether or not to claim your Publons credit. I understand this statement.

Yes Choose an item.
