# Supplementary material for: Enhanced bovine genome annotation through integration of transcriptomics and epi-transcriptomics datasets facilitates genomic biology
Source: Gigascience. 2024 Apr 16;13:giae019. doi: 10.1093/gigascience/giae019 (PMC11020238; doi:10.1093/gigascience/giae019)
Supplement: giae019_Reviewer_2_Report_Original_Submission — Pablo Augusto de Souza Fonseca -- 4/20/2023 Reviewed [file giae019_reviewer_2_report_original_submission.pdf]

Reviewer Report

Title: Enhancing Bovine Genome AnnotationThrough Integration of Transcriptomics and Epi-Transcriptomics Datasets Facilitates Genomic Biology

Version: Original Submission **Date:** 4/20/2023

Reviewer name: Pablo Augusto de Souza Fonseca

Reviewer Comments to Author:

The authors of "Facilitating Functional genomics of cattle through integration of multi-omics data" performed an extensive analysis of the transcriptome of multiple tissues, in different developmental stages, for cattle. The manuscript has a very interesting dataset and the analyses performed are scientifically sound. However, there are important points that must be adjusted and/or clarified.
Major comments:
My main concern is regarding the way that the results are presented and discussed. Despite the authors presenting very interesting results, the manuscript is very difficult to follow. In addition to a very long manuscript, which could be understandable due to the amount of analysis and results, the text seems to be extremely repetitive and basically descriptive. The results section, which has almost 20 pages, is composed of a series of sub-sections that are mainly descriptive statistics of the data. This kind of information could be summarized in Tables/Figures and the main results presented in the text. I suggest the authors perform a deep review in the Results section in order to provide a reduced version with the most relevant results, which will be further discussed. Additionally, the same information is presented in several parts of the manuscript. For example, the tissue-specific genes and transcripts are mentioned in multiple parts of the results section. In my opinion, the main objective of the authors "to facilitate the functional genomics of cattle" relies much more on other results rather than on the description of a number of transcripts, expressed genes, etc. For example, a deeper analysis of the alternative splicing across tissues would result in much more interesting results from the functional point-of-view. Additionally, the authors could focus on the functionality of the transcript with specific expression signatures (in a cluster of tissues, for example). The extensive description of summary statistics reduces substantially the impact and novelty of the results.
The material and methods section should be improved. I understand that due to the length of the manuscript, the authors decided to not show some details regarding the analysis and only cite the original manuscript where the analyses were performed. However, the authors should present the most relevant points, arguments, and decisions from each methodology. A reduction in other parts of the manuscript will allow the authors to improve this section as well.
The Discussion section is pretty much an overview of the results section. I believe that because the authors choose to focus mainly on the description of the number of transcripts, isoforms, genes, etc. providing discussion based on functionality became a difficult task. Here, the authors should discuss how the results help to improve the functional annotation in the cattle genome. In general, the discussion is generic and don't cover specific results obtained in the analysis. For example, which is the functional profile of the genes with specific alternative splicing in a given tissue or group of tissues? This is

interesting from the functional perspective. The results of the QTL-transcriptome associations should be discussed more in detail, providing more information regarding these associations and the specific patterns of association regarding the tissues and isoforms. However, it is very important to highlight the limitation of this approach, such as the limitations related to the database, the original association studies, breed-specific associations, etc.

Finally, I would suggest the authors remove multi-omics from the title. The study focuses on a multi-platform and multi-technique approach to evaluate transcriptomics. The closest analysis from other omics was the integration of ATAC-Seq and Chip-Seq data. However, the main results are focused on a single omics, transcriptomics.

Minor comments:

The abstract should be substantially improved. There are few explanations about the scientific question and hypothesis of the study. Additionally, the authors don't provide basic information regarding the dataset used in the study. Which were tissues analyzed? How many animals? The conclusions are vague and don't provide a perspective of the results.

Lines 51-53: This sentence is not connected with the previous one. Please, inform us how functional elements may help to fill the mentioned gap.

Line 56: Reference 2, Does this reference really reach this conclusion?

Line 58: Reference 3, The reference regarding this topic is quite old. Please, provide an updated one since the topic of the sentence passed through an intense development and increase in the number of publications in the last decade.

The last paragraph of the introduction presents a summary of the results obtained. The authors could use this part of the introduction to clearly state the objectives of the study.

Line 85: The word "diversity" is repeated in the sentence.

Line 91: Where is the description of all tissues?

Line 103-105: How? It is not clear how these 20,010 transcripts were actually expressed in multiple tissues.

Line 156: "Significantly higher than that was", please, review this sentence.

Line 159-163: This sentence is confusing.

Line 226-227: Please, replace "This supported an intersection analalysis" with "This supports an intersection analysis".

Line 247-250: This is a very broad BP term. How this could be interpreted?

Line 266-267: How does a protein-coding gene transcribe only non-coding transcripts? Please, provide more details to the readers.

Line 409-410: It seems that this information is repeated.

Line 611: It is missing a parenthesis.

The conclusions are generic and don't cover the main results obtained in the studies from a perspective of how those results fill the current gap observed in the literature. How the specific results obtained

Methods

Are the methods appropriate to the aims of the study, are they well described, and are necessary controls included? Choose an item.

Conclusions

Are the conclusions adequately supported by the data shown? Choose an item.

Reporting Standards

Does the manuscript adhere to the journal's guidelines on minimum standards of reporting? Choose an item.

Choose an item.

Statistics

Are you able to assess all statistics in the manuscript, including the appropriateness of statistical tests used? Choose an item.

Quality of Written English

Please indicate the quality of language in the manuscript: Choose an item.

Declaration of Competing Interests

Please complete a declaration of competing interests, considering the following questions:

- Have you in the past five years received reimbursements, fees, funding, or salary from an organisation that may in any way gain or lose financially from the publication of this manuscript, either now or in the future?
- Do you hold any stocks or shares in an organisation that may in any way gain or lose financially from the publication of this manuscript, either now or in the future?
- Do you hold or are you currently applying for any patents relating to the content of the manuscript?
- Have you received reimbursements, fees, funding, or salary from an organization that holds or has applied for patents relating to the content of the manuscript?
- Do you have any other financial competing interests?
- Do you have any non-financial competing interests in relation to this paper?

If you can answer no to all of the above, write 'I declare that I have no competing interests' below. If your reply is yes to any, please give details below.

I declare that I have no competing interests.

I agree to the open peer review policy of the journal. I understand that my name will be included on my report to the authors and, if the manuscript is accepted for publication, my named report including any attachments I upload will be posted on the website along with the authors' responses. I agree for my

report to be made available under an Open Access Creative Commons CC-BY license (http://creativecommons.org/licenses/by/4.0/). I understand that any comments which I do not wish to be included in my named report can be included as confidential comments to the editors, which will not be published.

Choose an item.

To further support our reviewers, we have joined with Publons, where you can gain additional credit to further highlight your hard work (see: https://publons.com/journal/530/gigascience). On publication of this paper, your review will be automatically added to Publons, you can then choose whether or not to claim your Publons credit. I understand this statement.

Yes Choose an item.
