# Supplementary material for: Enhanced bovine genome annotation through integration of transcriptomics and epi-transcriptomics datasets facilitates genomic biology
Source: Gigascience. 2024 Apr 16;13:giae019. doi: 10.1093/gigascience/giae019 (PMC11020238; doi:10.1093/gigascience/giae019)
Supplement: giae019_Reviewer_2_Report_Revision_1 — Pablo Augusto de Souza Fonseca -- 9/5/2023 Reviewed [file giae019_reviewer_2_report_revision_1.pdf]

Reviewer Report

Title: Enhancing Bovine Genome AnnotationThrough Integration of Transcriptomics and Epi-Transcriptomics Datasets Facilitates Genomic Biology

Version: Revision 1 **Date:** 9/5/2023

Reviewer name: Pablo Augusto de Souza Fonseca

Reviewer Comments to Author:

The authors provided detailed answers to all my comments and questions.
I have just minor comments regarding the manuscript.
The authors updated the manuscript title to "Improved annotation of the bovine genome identifies relationships between phenotypic traits". The study just searches for overlapping between the transcripts and publicly available QTL information. This approach helps to better understand the putative function of this transcript. However, it was not tested real associations between these transcripts and the traits. I would suggest the authors review the title of the manuscript. In my opinion, the study is much more focused on the improved annotation of the bovine genome and a screening of transcript isoforms than on the relationship between traits.
The changes in the discussion section were not tracked, which resulted in difficulty in following the edits.
In the conclusion, the authors mentioned that: "The integrated transcriptome data with publicly available QTL data revealed putative molecular pathways that may underlie tissue-tissue communication mechanisms and candidate genes responsible for the genetic mechanisms that may underlie genetic correlations between traits". It is not clear how the authors found this relationship between the QTLs and molecular pathways. The authors mentioned in the discussion section the analysis of the interconnection between testis and pituitary tissues with respect to the "percentage of normal sperm" and a potential association with a specific GO term. First, not necessarily a GO term represents a molecular pathway. Additionally, the authors mention only this example in the discussion section. The authors should provide a more comprehensive discussion about this approach and how the other results support potential associations between traits, mainly in the light of the next paragraph, where the results of the trait similarity results are discussed.

Methods

Are the methods appropriate to the aims of the study, are they well described, and are necessary controls included? Choose an item.

Conclusions

Are the conclusions adequately supported by the data shown? Choose an item.

Reporting Standards

Does the manuscript adhere to the journal's guidelines on minimum standards of reporting? Choose an item.

Choose an item.

Statistics

Are you able to assess all statistics in the manuscript, including the appropriateness of statistical tests used? Choose an item.

Quality of Written English

Please indicate the quality of language in the manuscript: Choose an item.

Declaration of Competing Interests

Please complete a declaration of competing interests, considering the following questions:

- Have you in the past five years received reimbursements, fees, funding, or salary from an organisation that may in any way gain or lose financially from the publication of this manuscript, either now or in the future?
- Do you hold any stocks or shares in an organisation that may in any way gain or lose financially from the publication of this manuscript, either now or in the future?
- Do you hold or are you currently applying for any patents relating to the content of the manuscript?
- Have you received reimbursements, fees, funding, or salary from an organization that holds or has applied for patents relating to the content of the manuscript?
- Do you have any other financial competing interests?
- Do you have any non-financial competing interests in relation to this paper?

If you can answer no to all of the above, write 'I declare that I have no competing interests' below. If your reply is yes to any, please give details below.

I declare that I have no competing interests

I agree to the open peer review policy of the journal. I understand that my name will be included on my report to the authors and, if the manuscript is accepted for publication, my named report including any attachments I upload will be posted on the website along with the authors' responses. I agree for my report to be made available under an Open Access Creative Commons CC-BY license (http://creativecommons.org/licenses/by/4.0/). I understand that any comments which I do not wish to be included in my named report can be included as confidential comments to the editors, which will not be published.

Choose an item.

To further support our reviewers, we have joined with Publons, where you can gain additional credit to further highlight your hard work (see: https://publons.com/journal/530/gigascience). On publication of this paper, your review will be automatically added to Publons, you can then choose whether or not to claim your Publons credit. I understand this statement.

Yes Choose an item.
