# Supplementary material for: Enhanced bovine genome annotation through integration of transcriptomics and epi-transcriptomics datasets facilitates genomic biology
Source: Gigascience. 2024 Apr 16;13:giae019. doi: 10.1093/gigascience/giae019 (PMC11020238; doi:10.1093/gigascience/giae019)
Supplement: giae019_Reviewer_3_Report_Original_Submission — Christopher Hunter, Ph.D. -- 5/2/2023 Reviewed [file giae019_reviewer_3_report_original_submission.pdf]

Reviewer Report

Title: Enhancing Bovine Genome AnnotationThrough Integration of Transcriptomics and Epi-Transcriptomics Datasets Facilitates Genomic Biology

Version: Original Submission **Date:** 5/2/2023

Reviewer name: Christopher Hunter, Ph.D.

Reviewer Comments to Author:

The editorial team requested that I carry out a peer-review of your manuscript focussing particularly on transparency and reproducibility.
In general I believe the manuscript covers a great deal of work and provides a very useful resource, and I would recommend acceptance after various revisions. My major concerns are all related to the metadata and correlation between data-deposited and the descriptions in the manuscript.
I can see from the Data Availability statement in the manuscript that you have submitted the raw sequence data to the SRA at EBI (ENA) via the ArrayExpress submission route. They have been assigned under accession numbers E-MTAB-11699 (RNA-Seq & miRNA-seq), E-MTAB-11815 (ATAC-seq), and E-MTAB-12052 (WTTS-seq).
You have also provided a "Sample_meta-data.tsv" file via the user115 area provided by GigaDB.
1 - In the Methods section, sub heading RNA-seq library construction it says "Tissue samples (Supplemental file 22) were collected from storage at -80 Â°C". A section prior to that describes Adult tissue collection methods stating that 2 male and 2 female cattle were used. Neither section nor Sup file 22 include the animal identifier or any means to determine which tissue samples were used from which donor animal. Maybe sup file 22 could be expanded to include columns for each of the 4 animals with y/n datum to identify which tissues were sequenced from each animal? Or perhaps instead of y/n you could include the BioSample accession number of the deposited data for those used.
2 - The RNA-seq library construction section also mentioned that RNA quantity and quality was measured. While not required, we would encourage you to share those results in GigaDB.
3 - Mammary gland tissue collection and RNA-seq library construction section; previous discussion on this topic resulted in you changing the text to:
"Mammary gland tissue collection. The 14 animals used in this study were Holstein-Friesian heifers from a single herd managed at the AgResearch Research Station in Ruakura, NZ. All experimental protocols were approved by the AgResearch, NZ, ethics committee and carried out according to their guidelines. Samples were collected from animals at 4-time points: virgin state before pregnancy between 13 and 15 months of age (virgin), mid-pregnant at day 100 of pregnancy, late pregnant ~2 weeks pre-calving, and early lactation ~2 weeks post-calving. Tissue samples were obtained by mammary biopsy using the Farr method [2]. Lactating cows were milked before biopsy and sampled within 5 hours of milking. Biopsy sites were clipped and given aseptic skin preparation (povidone-iodine base scrub and iodine tincture) and subcutaneous local anesthetic (4 ml per biopsy site). Core biopsies were taken using a powered sampling cannula (4.5 mm internal diameter) inserted into a 2 cm incision. The resulting samples of mammary gland parenchyma measured 70 mm in length with a 4 mm diameter.

Due to the limited amount of tissue samples collected from an individual animal. RNA for RNA-seq analysis was isolated from 4 animals, RNA for miRNA-seq was isolated from 6 animals, RNA for WTTS-seg was isolated from 4 animals, and DNA for ATAC-seq analysis from 7 animals (SUPPLEMENT FILE NO)."
Based on the revised text it is still not possible to determine which individuals have been used for which assays. Could a similar table to the one suggested for the tissue samples above (1) be created here?
4 - The Illumina RNA-Seq technologies section includes the text "Only samples with RIN values >8 were used for cDNA synthesis" (note- RIN needs to be added to the list of abbreviations in the document), it is not possible to determine from this which samples were actually used in this experiment and which were not.
Perhaps it would be appropriate to share the RNA integrity analysis results here? GigaDB can host electrophoresis gel images if that is how it was performed.
5 - The supplemental files provided in the user115 area. These all include the tissue name in their file-names, some have spelling mistakes, but even taking those into account I find 51 different tissues in those names, but the manuscript states 47 were investigated. Its probably just a classification and/or different subsets of things, but for transparency using a consistent nomenclature and providing accession numbers will be useful. Please ensure the files are named correctly with the appropriate tissue names.
6 - miRNAs. The set of "supplemental file 21" files provided in user115 area all list the miRNAs by some sort of identifier and state whether they are known or novel. Do those identifiers relate directly to miRbase? And have they all been deposited and released already? I tried to search for one of the novel ones "bta-miR-X44036" in miRbase but it did not find anything.
7 - Gene expression analysis. I believe from the methods section that you pooled all transcripts from all similar/same tissues and determined the tissue the expression levels based on those. From my limited understanding of statistics I would assume it better to do a per sample analysis of the expression levels first to enable one to determine confidence levels by biological replicates.
The methods also state that "…outlier samples were expressed and removed from downstream analysis. Samples from each tissue were combined to…". For transparency and reproducibility, please provide a list of the removed samples and a list of those samples data that were combined (ideally that will include both the tissue names and the relevant SRA sequence run accession numbers).
8 - "The resulting transcripts from each tissue were re-grouped into gene models using an in-house Python script. Structurally similar transcripts from the different tissues (see Comparison of transcript structures across datasets/tissues section) were collapsed using an in-house Python script to create the RNA-seq based bovine transcriptome."
Please confirm that those two in-house scripts are included in the GitHub repository cited in the data availability section? If not, please add them there.
9- ONT data analysis. You have cited the manuscript describing the data you have reused (Halstead et al 2021) which is great, thank you. However, having had a quick look at that manuscript it is not clear exactly what data you have reused, the only accession they quote in that manuscript is to a massive series of data hosted in GEO (GSE160028) which includes Pig, Cow and Chicken data. For the convenience of your readers would you also be able to point to a more useful accession of the data you actually utilised here e.g. the assembled isoform sequences ?
10 - The correlation between the various methods sections and the data being made available is very

difficult to determine with any certainty. Perhaps it would be beneficial to expand the sample table provided to include all the unique identifiers for every sample and correlate those to the methodologies listed in the manuscript. It maybe appropriate to incorporate a column to denote the samples removed from certain analysis, with an explanation as to why?

Including the ENA sample and/or BioSample accessions in the sample table (the ENA sample accessions start with ERS, BioSample accessions start with SAMEA) will greatly enhance the transparency of the data utilised in this study. In addition it will allow you to double check the metadata you have provided on each sample.

For example; I picked one at random to look into more closely. It is listed in the Samples_meta-daat.tsv spreadsheet you provided as having the accession "ERR10162191" (which is a run accession not a sample accession). I have compared this to the data submitted to Array Express (https://www.ebi.ac.uk/biostudies/arrayexpress/studies/E-MTAB-12052/sdrf?full=true) to find that run accession number and look up the relevant BioSample and ENA Sample accessions (ERS13425945, SAMEA111328380). In doing so I noticed that the "individual" value given in your spreadsheet says "M08" yet in Array Express it says "M22"? Clearly, one of those cannot be correct. As it was honestly the first and only sample I looked at in such depth, it worries me that there maybe other inconsistencies that you will need to check and correct.

May I suggest you have someone in your team take a very careful look at the Samples submitted to Array Express, including the various different accessions that they assign (ENA sample accessions and BioSample accessions) and ensure that all sample have been submitted and have accurate and complete metadata, the geolocation information should be included with all samples. (NB the more metadata you can provide to the archives the more discoverable and reusable your data becomes). Then prepare the Samples spreadsheet from that information and relate it directly to the experiments described in the manuscript at the sample level.

Methods

Are the methods appropriate to the aims of the study, are they well described, and are necessary controls included? Choose an item.

Conclusions

Are the conclusions adequately supported by the data shown? Choose an item.

Reporting Standards

Does the manuscript adhere to the journal's guidelines on [minimum standards of reporting?]() Choose an item.

Choose an item.

Statistics

Are you able to assess all statistics in the manuscript, including the appropriateness of statistical tests used? Choose an item.

Quality of Written English

Please indicate the quality of language in the manuscript: Choose an item.

Declaration of Competing Interests

Please complete a declaration of competing interests, considering the following questions:

- Have you in the past five years received reimbursements, fees, funding, or salary from an organisation that may in any way gain or lose financially from the publication of this manuscript, either now or in the future?
- Do you hold any stocks or shares in an organisation that may in any way gain or lose financially from the publication of this manuscript, either now or in the future?
- Do you hold or are you currently applying for any patents relating to the content of the manuscript?
- Have you received reimbursements, fees, funding, or salary from an organization that holds or has applied for patents relating to the content of the manuscript?
- Do you have any other financial competing interests?
- Do you have any non-financial competing interests in relation to this paper?

If you can answer no to all of the above, write 'I declare that I have no competing interests' below. If your reply is yes to any, please give details below.

I am employed by GigaScience Press as a data curation expert in GigaDB, my review of this manuscript is only looking at data transparency and availability with no review of the scientific conclusions made.

I agree to the open peer review policy of the journal. I understand that my name will be included on my report to the authors and, if the manuscript is accepted for publication, my named report including any attachments I upload will be posted on the website along with the authors' responses. I agree for my report to be made available under an Open Access Creative Commons CC-BY license (http://creativecommons.org/licenses/by/4.0/). I understand that any comments which I do not wish to be included in my named report can be included as confidential comments to the editors, which will not be published.

Choose an item.

To further support our reviewers, we have joined with Publons, where you can gain additional credit to further highlight your hard work (see: https://publons.com/journal/530/gigascience). On publication of this paper, your review will be automatically added to Publons, you can then choose whether or not to claim your Publons credit. I understand this statement.

Yes Choose an item.
