# Supplementary material for: Enhanced bovine genome annotation through integration of transcriptomics and epi-transcriptomics datasets facilitates genomic biology
Source: Gigascience. 2024 Apr 16;13:giae019. doi: 10.1093/gigascience/giae019 (PMC11020238; doi:10.1093/gigascience/giae019)
Supplement: giae019_Reviewer_3_Report_Revision_1 — Christopher Hunter, Ph.D. -- 9/6/2023 Reviewed [file giae019_reviewer_3_report_revision_1.pdf]

Reviewer Report

Title: Enhancing Bovine Genome AnnotationThrough Integration of Transcriptomics and Epi-Transcriptomics Datasets Facilitates Genomic Biology

Version: Revision 1 **Date:** 9/6/2023

Reviewer name: Christopher Hunter, Ph.D.

Reviewer Comments to Author:

Overall the paper has been improved, but it is still far from transparent when trying to marry public data with discussions in the paper, as there is a general lack of consistency in naming conventions between the public data and the manuscript.
Since the manuscript has been drastically re-arranged I will not attempt to deal with each of my previous comments, instead I will deal with issues that I have now found in the new version, which may or may not have been obvious in the previous version.
GigaDB data:
Please ensure the data provided in the private dropbox area of GigaDB (user115) is correct with regards to the revised manuscript.
Abstract:
In the abstract it is stated "A total number of 171,985 unique transcripts (50% protein-coding) representing 35,150 unique genes (64% protein-coding)".
The supplemental_file14 contains lists of all genes and transcripts, however it only includes 34882 and 160820 unique genes and transcripts respectively not the same as stated in the abstract, please clarify which is correct? And ensure other mentions of those numbers in the manuscript are also correct.
Results section:
"The diversity of RNA and miRNA transcript among 50 different bovine tissues and cell types was assessed…" I am still unclear how the number 50 has been reached? Supplemental_file1 includes 51 different names of tissues, however, 5 of those names are actually mammary gland at different time points, so its debatable if they constitute different tissue or cell type?
From a data archiving perspective the Tissue values should all use valid ontology terms as the tissue field is not meant for distinguishing different time points of sampling, there are other metadata fields for that information.
The use of valid ontology terms will enable others to discover and re-use these data appropriately, and is considered good-practice.
Trait similarity network
The section on trait similarity is perplexing me (and this maybe my lack of experience in this area). Many of the traits mentioned in the network are related to phenotypic measurements, e.g. sperm volume. So does that mean you have captured many phenotypic values for all the sampled animals? If so, where are those data?
In the Methods section

Where the bioinformatics analysis steps are mentioned; "The overview of the bioinformatics analysis steps is presented in Supplemental file 2: Fig. S39." The authors should include reference to the annotated script file provided to GigaDB.
In the RNA-seq data analysis and transcriptome assembly section
The statement "…outlier samples were expressed and removed from downstream analysis." requires evidence. All sequence data generated must be submitted to the archives and cited by accession number, especially where you have removed it from further analysis as an outlier. If you do not provide those data you are open to accusations of cherry-picking your data.
Supplemental_file5
The description of the supplemental file 5 in the manuscript differs from the content, please check all supplemental files contain the expected data and are correctly described in the manuscript.
Supplemental_file23
The addition of supplemental_file23.docx has helped clarify some aspects, but it has also drawn attention to some (possibly) missing data;
- The section sub headed "Cell sample collections" describes how some cells were grown, however the main manuscript does not describe these results clearly and I am unable to determine what analysis was actually done with those cells? Were they sequenced? If so, which BioSample accessions do they relate to?
For better clarity, would it be possible to list the unique Animal IDs within each section, e.g. Adult tissue collection change "Eleven cattle (6 males and 5 females) were slaughtered…" to "Eleven cattle (6 males- M08, M09,M10, M11, M130, M22, M23, and 5 females- F05, F06, F07, F12) were slaughtered…"
As you can see above, by looking at the "Samples_meta-data.tsv" provided and filtering for age 420days* it appears there are actually 7 males and 4 females not 6 and 5 as stated in the MS, please clarify which is correct.
*- why use 420 days in the archive but 4 months in the paper? Try to be consistent.
"Mammary gland tissue collection. The 14 animals used in this study… Samples were collected from animals at 4 time points: virgin state before pregnancy between 13 and 15 months of age (virgin), mid-pregnant at day 100 of pregnancy, late pregnant ~2 weeks pre-calving, and early lactation ~2 weeks post-calving."
In the supplemental_file1 table, when I filter for tissue= mammary gland (virgin), mammary gland (late pregnant), mammary gland (early lactating), or mammary gland (mid pregnant); I can only find 10 different Animal IDs; mam-01, mam-02, mam-03, mam-09, mam-10, mam-11, mam-13, mam-14, mam-15, mam-16. Where are the data for the other 4 animals? It appears maybe there is a 5th mammary tissue "mammary gland (adult)" that may account for the other 4 samples, which means the manuscript statement of 4 time points is incorrect.
"RNA-seq library construction. Tissue samples (Supplemental file 1) were collected from live" - supplemental_file1 does not contain a list of tissues, it is a table of all different sequence run experiments.
The section titled "Sequencing the transcriptomes of seven bovine tissues by using the PacBio Iso-Seq and Illumina RNA-Seq technologies" it is unclear to me why it starts by stating previously published data were used and then goes on to describe how you extracted RNA. Is that a description of how those previously published data were created? Or is it describing additional sequencing carried out by

yourselves for this study? If the later, please clarify which NCBI accessions relate to those data. Despite all the above issues, I believe the manuscript is well intended and contains a lot of useful and informative details that are worthy of publication. The authors should spend more time and care over the data and metadata organisation to enable a greater reuse potential by others, and ensure transparency in their findings.

Methods

Are the methods appropriate to the aims of the study, are they well described, and are necessary controls included? Choose an item.

Conclusions

Are the conclusions adequately supported by the data shown? Choose an item.

Reporting Standards

Does the manuscript adhere to the journal's guidelines on minimum standards of reporting? Choose an item.

Choose an item.

Statistics

Are you able to assess all statistics in the manuscript, including the appropriateness of statistical tests used? Choose an item.

Quality of Written English

Please indicate the quality of language in the manuscript: Choose an item.

Declaration of Competing Interests

Please complete a declaration of competing interests, considering the following questions:

- Have you in the past five years received reimbursements, fees, funding, or salary from an organisation that may in any way gain or lose financially from the publication of this manuscript, either now or in the future?
- Do you hold any stocks or shares in an organisation that may in any way gain or lose financially from the publication of this manuscript, either now or in the future?
- Do you hold or are you currently applying for any patents relating to the content of the manuscript?
- Have you received reimbursements, fees, funding, or salary from an organization that holds or has applied for patents relating to the content of the manuscript?

- Do you have any other financial competing interests?
- Do you have any non-financial competing interests in relation to this paper?

If you can answer no to all of the above, write 'I declare that I have no competing interests' below. If your reply is yes to any, please give details below.

I am a GigaScience Press employee, my review is only focused on the data transparency and availability not the scientific content. I declare that my review is unbiased and all comments are my personal opinions, which may or may not be aligned those of my employer.

I agree to the open peer review policy of the journal. I understand that my name will be included on my report to the authors and, if the manuscript is accepted for publication, my named report including any attachments I upload will be posted on the website along with the authors' responses. I agree for my report to be made available under an Open Access Creative Commons CC-BY license (http://creativecommons.org/licenses/by/4.0/). I understand that any comments which I do not wish to be included in my named report can be included as confidential comments to the editors, which will not be published.

Choose an item.

To further support our reviewers, we have joined with Publons, where you can gain additional credit to further highlight your hard work (see: https://publons.com/journal/530/gigascience). On publication of this paper, your review will be automatically added to Publons, you can then choose whether or not to claim your Publons credit. I understand this statement.

Yes Choose an item.
